# Supplementary material for: Protocol for a cluster randomised controlled trial of the DAFNEplus (Dose Adjustment For Normal Eating) intervention compared with 5x1 DAFNE: a lifelong approach to promote effective self-management in adults with type 1 diabetes
Source: BMJ Open. 2021 Jan 18;11(1):e040438. doi: 10.1136/bmjopen-2020-040438 (PMC7813353; doi:10.1136/bmjopen-2020-040438)
Supplement: Supplementary data [file bmjopen-2020-040438supp001.pdf]

Supplementary material 1 – WHO Trial Registration Dataset

Data Category	Information
Primary registry and trial identifying number	http://www.isrctn.com/ISR ISRCTN42908016
Date of registration in primary registry	08/05/2018
Secondary identifying numbers	Sheffield CTRU: J13-003<br>Sponsor ID: STH20111<br>IRAS: 235621<br>Funding ref: RP-PG-0514-20013<br>REC: 18/SW/0100
Source(s) of monetary or material support	National Institute for Health Research (NIHR) (UK)
Primary sponsor	Sheffield Teaching Hospitals NHS Foundation Trust
Secondary sponsor(s)	N/A
Contact for public queries	Trial manager (Elaine Scott) 0114 222 5158 or dafneplus@sheffield.ac.uk
Contact for scientific queries	Trial manager (Elaine Scott) 0114 222 5158 or dafneplus@sheffield.ac.uk
Public title	DAFNE*plus* Cluster RCT
Scientific title	A cluster randomised controlled trial (RCT) of the DAFNE*plus* (Dose Adjustment for Normal Eating) intervention: A lifelong approach to promote effective self-management in adults with type 1 diabetes
Countries of recruitment	England and Scotland
Health condition(s) or problem(s) studied	Type 1 diabetes
Intervention(s)	DAFNE*plus* (Dose Adjustment for Normal Eating) intervention
Key inclusion and exclusion criteria	*Inclusion criteria:*<br>• Adults (≥18 years);<br>• Diagnosis of type 1 diabetes for at least 6 months, or post-honeymoon;<br>• Prepared to undertake multiple daily injection (MDI) therapy;<br>• Prepared to undertake frequent self-monitoring of blood glucose;<br>• Confirms availability to attend all sessions as part of the intervention;

	• Investigator has confidence that the patient is capable of adhering to all the trial protocol requirements.<br><br>*Exclusion criteria:*<br>• Current use of continuous subcutaneous insulin infusion (CSII) pump therapy<br>• HbA1c > 12%/108 mmol/mol (Investigators can use their judgement, informed by standard DAFNE guidelines and in agreement with the trial team, to include participants with HbA1c >12%/108 mmol/mol).<br>• Serious diabetic complications (e.g. blindness, renal dialysis). (Investigators can use their clinical judgement, informed by standard DAFNE guidelines and in agreement with the trial team).<br>• Other serious co-morbidities e.g. psychosis, diagnosed eating disorder (Investigators can use their clinical judgement, informed by standard DAFNE guidelines and in agreement with the trial team).<br>• Previous participation in standard DAFNE course less than 5 years before proposed study enrolment date<br>• Unable to speak/hear/understand/read write in English<br>• Unable to give written informed consent
Study type	Multi-centre cluster randomised controlled trial with process evaluation and economic evaluation, comparing DAFNE*plus* to standard DAFNE for adults with type 1 diabetes.
Date of first enrolment	01/09/2018
Target sample size	662 participants – 47 per centre.<br><br>Fourteen secondary care diabetes centres in the National Health Service in England and Scotland<br><br>In addition, we aim to recruit 20 DAFNE*plus* facilitators to take part in qualitative interviews for the process evaluation.
Recruitment status	Recruiting
Primary outcome(s)	The primary biomedical outcome is glycaemic control, defined as the change in HbA1c at 12 months (using a centralised assay to ensure standardisation), in those entering the trial with HbA1c >7.5% (estimated at 75% of those currently undertaking DAFNE courses based on our research database).
Key secondary outcomes	Secondary biomedical outcome:<br>Number of participants achieving either an HbA1c <7.5% (58 mmol/mol) or a decrease in HbA1c of ≥0.5% (≥5.5 mmol/mol) (using a centralised assay to ensure standardisation). These endpoints will be calculated using data collected at baseline and 12 months after the course.<br><br>Other secondary biomedical outcomes will include:<br>1. Severe hypoglycaemia, as defined by the American Diabetes Association, denotes severe

	cognitive impairment requiring external assistance for recovery, both rates and proportion of<br>those affected, measured at baseline at 12 months after the course<br>2. Diabetic ketoacidosis, both rates and proportion of those affected, collected at baseline and<br>12 months after the course<br>3. Weight, measured at baseline and 12 months after the course<br>4. Body Mass Index, measured at baseline and 12 months after the course<br>5. Blood pressure, measured at baseline and 12 months after the course<br>6. Lipids, measured at baseline and 12 months after the course<br>7. Albumin/ creatinine, measured at baseline and 12 months after the course<br><br>The primary psychological outcome is the measurement at 12 months of the Audit-Dependent Diabetes Quality of Life Questionnaire (ADDQoL-15), a thirty-item measure of diabetes-specific quality of life.<br><br>Psychological outcomes, measured at baseline, course completion, 3, 6 and 12 months:<br>1. Dawn Impact of Diabetes Profile<br>2. Problem Areas in Diabetes Scale<br>3. Diabetes-specific positive well-being<br>4. Hypoglycaemia Fear Survey<br><br>Process measures:<br>5. Diabetes Management Experiences Questionnaire<br>6. Self-Regulation/Behavioural Regulation Questionnaire<br>7. Diabetes Strengths & Resilience Questionnaire<br>8. Confidence in Diabetes Scale assesses beliefs about capabilities (self-efficacy).<br>9. Diabetes Self-Care Behaviours<br>10. Hypoglycaemia Confidence Scale<br>11. Beliefs about consequences of engaging in DAFNE behaviours and weaving diabetes management into everyday routines.<br>12. The System Usability Score<br>13. Use and dose received of the DANFE*plus* programme assessed via logs of attendance at group and individual sessions, and use of the DANFE*plus* website<br><br>Hypoglycaemia Awareness<br>14. Hypoglycaemia awareness assessed via Gold score<br><br>Health economic measures assessed at baseline, course completion, 6 and 12 months using:<br>1. Health status – EQ-5D-5L<br>2. Health and Self-Management in Diabetes HASMID<br>3. Healthcare utilisation using a bespoke questionnaire<br>4. Contact between professionals and course participants will also be recorded at each site using questionnaires and data from the DANFE*plus* website (in the intervention arm)
