# Supplementary material for: Protocol for a cluster randomised controlled trial of the DAFNEplus (Dose Adjustment For Normal Eating) intervention compared with 5x1 DAFNE: a lifelong approach to promote effective self-management in adults with type 1 diabetes
Source: BMJ Open. 2021 Jan 18;11(1):e040438. doi: 10.1136/bmjopen-2020-040438 (PMC7813353; doi:10.1136/bmjopen-2020-040438)
Supplement: Supplementary data [file bmjopen-2020-040438supp004.pdf]

Supplementary material 4 – Development and modification of questionnaires and individual items

Confidence in Diabetes Scale (CIDS)

This scale assesses the extent to which an individual believes they can engage in particular behaviours related to their type 1 diabetes treatment regimen (self-efficacy). The scale was published in 2003 and Dr Cooke contacted Prof Snoek, the senior author on the original validation paper to seek permission to amend item 3 on the scale (*I believe I can perform the prescribed number of daily insulin injections*). He gave his permission for the team to make the following amendment to reflect the more flexible approach to multiple daily insulin treatment regimens, advocated by courses like DAFNE and DAFNE*plus* (*I believe I can perform the number of daily insulin injections I need to*).

Self-Regulation Questionnaire for Type 1 Diabetes (SRQ-T1D)

This questionnaire is an adaptation of the Self-Regulation Questionnaire [1]. Self-regulation is the ability to develop, implement, and flexibly maintain planned behaviour in order to achieve one's goals. The SRQ and our adaptation of this builds on the work of Frederick Kanfer and two researchers who formulated a seven-step model of self-regulation [2,3]. Although this model was developed specifically to study addictive behaviours, the self-regulatory processes it describes are meant to be general principles of behavioural self-control. In this model, people may have problems managing certain behaviours (behavioural self-regulation) because of challenges at any of these seven steps:

1. **Receiving** relevant information
2. **Evaluating** the information and comparing it to norms
3. **Triggering** change
4. **Searching** for options
5. **Formulating** a plan
6. **Implementing** the plan
7. **Assessing** the plan's effectiveness (which recycles to steps 1 and 2)

The original SRQ has demonstrated reliability, concurrent and discriminant validity in community samples[4]. It consists of 63 items which was too long for our team to use in the DAFNE*plus* questionnaire pack, when this is one of several process measures. This measure was reviewed by 3 members from the DAFNE*plus* PPI group and by our process evaluation team consisting of clinicians, behavioural scientists, psychologists and social anthropologists, two of whom also have type 1 diabetes. The PPI group strongly recommended altering the wording of the individual items slightly so that these were all framed to be diabetes-specific in focus, rather than generic. Dr Cooke, in discussion with two members of the PPI group amended the wording of some of these items to ensure that they were clear and made sense. The process evaluation team and PPI group selected their top 2-3 items from each of the seven categories (above), rank ordering them. Dr Cooke then reviewed these to select the items from each

category which the majority had agreed should be included within the final questionnaire.

Beliefs about Consequences of Diabetes Self-Care Behaviours; Diabetes Support and Routines

The DAFNE*plus* revisions to the original DAFNE curriculum were structured around the Theoretical Domains Framework[5] hence it is very important to the process evaluation team to assess the constructs that are being targeted within individuals through the content and delivery of the DAFNE*plus* course; to assess whether participants in the DAFNE*plus* and standard DAFNE groups respond differently on these measures but also whether these constructs explain any differences in outcomes (HbA1c and diabetes-specific quality of life). Three of these constructs are 'social influences', 'beliefs about consequences of diabetes self-care' and 'environmental cues and prompts'. The research team have generated 11 diabetes-specific items to assess these constructs and have piloted them with our PPI group. These are unvalidated but once we have collected data at two timepoints (course completion and 3-months follow-up), if these measures are shown not to be psychometrically robust, we will remove these items from the 9-month follow-up point. Please note that we are only collecting these process measures at 3 timepoints.

References

1 Brown J, Miller W, LewandowskiLA. The Self-Regulation Questionnaire. In: Creek, Van de L, Jackson T, eds. *Innovations in clinical practice: A source book*. Sarasota, Florida: : Professional Resource Press 1999. 281–9.

2 Brown JM. Self-Regulation and the Addictive Behaviors. In: *Treating Addictive Behaviors*. Springer US 1998. 61–73. doi:10.1007/978-1-4899-1934-2_5

3 Miller W, Brown J. Self-Regulation as a Conceptual Basis for the Prevention and Treatment of Addictive Behaviours. In: Heather N, Miller W, Greenley J, eds. *Self-Control and the Addictive Behaviours*. Sydney: : Maxwell MacMillan Publishing Australia 1991. 3–79.

4 Aubrey L, Brown J, Research WM-AC& E, *et al.* Psychometric properties of a selfregulation questionnaire (SRQ). *Alcohol Clin Exp Res* 1994;**18**:429.

5 Atkins L, Francis J, Islam R, *et al.* A guide to using the Theoretical Domains Framework of behaviour change to investigate implementation problems. *Implement Sci* 2017;**12**:77. doi:10.1186/s13012-017-0605-9
