# Supplementary material for: Protocol for a cluster randomised controlled trial of the DAFNEplus (Dose Adjustment For Normal Eating) intervention compared with 5x1 DAFNE: a lifelong approach to promote effective self-management in adults with type 1 diabetes
Source: BMJ Open. 2021 Jan 18;11(1):e040438. doi: 10.1136/bmjopen-2020-040438 (PMC7813353; doi:10.1136/bmjopen-2020-040438)
Supplement: Supplementary data [file bmjopen-2020-040438supp002.pdf]

Supplementary material 2 – Protocol for DAFNEplus Process evaluation

Aims and research questions

Understanding processes is as important as evaluating outcomes; process evaluations are complementary to outcomes evaluations and provide knowledge and information of equal value. Process evaluations aim to understand the functioning of an intervention by examining its implementation, mechanisms of impact and how contextual factors (i.e. factors external to the intervention/individual receiving the intervention) might affect its delivery and receipt [1,2]. Without this knowledge we may be able to establish from an outcome evaluation that an intervention 'works', but we will be presuming that the intended intervention was delivered and is effective, and we will not necessarily know how, or why, the intervention works and, hence, if it would have the same clinical and psychological effect if rolled out from a trial situation into routine clinical practice. With a complex intervention such as DAFNE*plus* it may well also be that some elements are more vital to its success than others; hence it is very important that we understand and explore the mechanisms of change on outcome from the perspectives of those receiving the intervention, as well as unintended consequences arising from the delivery and receipt of the intervention.

Our overarching research questions are:

1. **Does the DAFNE*plus* intervention 'work' in the ways intended? If not, why not?**

2. **What are the implications of the findings of the process evaluation for the rollout of DAFNE*plus* in routine clinical practice?**

To answer these over-arching questions, a series of over-lapping sub-questions will be explored:

a) What mechanisms change impact on glycaemic control? That is, how do the different elements of DAFNE*plus* (knowledge/skills, technological, structured follow-up), individuals' interaction with these elements, and individual psychological differences trigger changes in and maintenance of key diabetes self-management behaviours? The theoretical model underpinning the DAFNEplus programme assumes that diabetes self-management behaviours are among the principal determinants of glycaemic control.

b) What mechanisms of change impact on diabetes-specific quality of life?

c) What are participants' experiences of, and views about, key elements of the DAFNE*plus* intervention[1] and how do these influence and inform changes in, and maintenance of, key diabetes self-management behaviours over time?

[1] As a result of work undertaken in the pilot phase and MRC guidance to focus on key areas of uncertainty of greatest interest to academic and clinical audiences, a decision has been made to focus upon the technological and resilience/self-compassion elements of the programme.

d) To what extent is the intervention delivered as intended and are there variations between sites and individuals as to how the DAFNE*plus* intervention is delivered? What are the reasons for any variations?

e) What impact (practical and emotional) does intervention delivery have on facilitators and their workloads; what resourcing and support would facilitators and their colleagues need to deliver DAFNE*plus* in routine clinical practice?

f) Do any unintended consequences arise from the delivery and receipt of the DAFNE*plus* intervention, for participants and/or facilitators?

The data sources for each of the sub-questions are shown in table 1.

Table 1 – Data sources for the process evaluation

Research Question	Data source(s)
a) What mechanisms of change impact on glycaemic control?	• Questionnaire study<br>• Process outcomes<br>• Fidelity assessment<br>• Qualitative (from DAFNE*plus* pilot study)
b) What mechanisms of change impact on diabetes-specific quality of life	• Questionnaire study<br>• Process outcomes
c) What are participants' experiences of DAFNE*plus*?	Qualitative
d) To what extent is the intervention delivered as intended?	• Fidelity assessment<br>• Qualitative
e) What impact does intervention delivery have on facilitators and their workloads?	Qualitative
f) Do any unintended consequences arise from the delivery and receipt of the DAFNE*plus* intervention?	• Qualitative<br>• Questionnaire study<br>• Process outcomes<br>• Fidelity assessment

The process evaluation is composed of three interlinking components: (1) qualitative, (2) quantitative and (3) assessment of fidelity of delivery.

(1) Qualitative component

1.1 Overview

The qualitative component of the process evaluation will be informed by realist and Normalization Process theory (NPT) [3,4]. These choices arise from our recognition that context (i.e. factors external to the DAFNE*plus* intervention and/or the individual receiving the intervention) may influence how the intervention is delivered in different centres and how it is received by different individuals. It is also recognised that, when

a complex intervention, such as DAFNE*plus*, is implemented it can have unintended consequences, which may need to be investigated and, hence, that a flexible and adaptive study design will be required.

An iterative, inductive approach will be used wherein data analysis will commence as soon as data collection begins [5]. This will allow issues arising during early phases of qualitative data collection to inform questions asked in later phases and possibly also sampling. The qualitative research will also be responsive to other aspects of the process evaluation, including the fidelity work. Hence, while case studies will comprise the main element of the qualitative research (see below), costings have been included to allow, if necessary, one-off interviews to be undertaken with a 'booster' sample of patients, facilitators and/or other individuals in the event that the quantitative/fidelity components of the process evaluation highlight issues which require qualitative explanation. One example might be that, if the fidelity work highlights significant variations between trial sites as to how the DAFNE*plus* intervention is delivered, we may decide to interview additional facilitators to better understand why this might be the case.

1.2 Qualitative study design: case study approach

A case study approach will be used because it permits detailed exploration of if, how and why the intervention works in different contexts [6]. Each case will comprise: (a) participants who will be interviewed before, during and following completion of DAFNE*plus*, (b) their facilitators who will be interviewed after the participant's closeout from the trial, (c) information about the input and care the participant receives as part of DAFNE*plus* and their engagement with DAFNE*plus* technologies/resources. It will be possible to access this information via clinical records, the Glucollector website and information documented in case report forms and stored on PROSPECT (the CTRU database). Where identifiable clinical information needs transferring between NHS and University sites files will be encrypted and nhs.net accounts or Google Drive will be used. As part of the process evaluation, we will also have access to recordings of participants' face-to-face follow-up sessions with facilitators – these data are being collected for the fidelity assessment work, and data on utilisation of DAFNE*plus* technological components and adherence. Researchers from the University of Edinburgh will also sit in some DAFNEplus sessions as observers, to familiarise themselves with the processes and material to inform the case studies.

Participant Sampling

Two or three participants from each of the seven DAFNE*plus* sites will be selected for the qualitative work, and these individuals will be purposively sampled so there is representation of people of different ages, HbA1c levels, diabetes duration, gender, occupation, educational background, personal circumstance (e.g. single, partnered, parent) and place of residence (e.g. urban and rural locations).

Data collection: participant interviews

Selected participants will be interviewed at four time-points: prior to attending their course, following their course, and 3 and 12 months post course. Interviews will be informed by topic guides. Prior to undertaking a follow-up interview, a participant's previous interviews will be reviewed. As well as including more generic questions, follow-up interviews will be tailored to allow for follow-up of specific issues raised by particular individuals. Questions explored in the post course, 3 and 12 month

interviews will also take account of a review of information collected in medical records, via DAFNEplus technology and audio recorded follow-up sessions. Interviews will take place by telephone (unless an individual requests a face-to-face interview) at a time most convenient to the participant. All interviews will be digitally recorded with consent. It is anticipated that each interview will take 60 minutes to complete.

Facilitator interviews
Each participant's facilitators (n=1-2 per participant) will be interviewed following their close-out from the trial. If the participant received care from more than two facilitators as part of DAFNE*plus* we will ask them to nominate the two individuals from whom they felt they had the most input.

Facilitators will be interviewed once following the participant's close-out from the trial. This decision has been made partly for pragmatic reasons (i.e. we do not want to make excessive demands on the health professionals' time) and also because it will be possible to access information about the participant's care and the decisions made from the contact logs, clinical records and recordings of follow-up sessions. It is also recognised that, if facilitators are made aware that the participant is included in the process evaluation, this might influence or bias the care which is given, although participants will not be prohibited from telling their facilitators they are taking part in the qualitative research should they choose to do so.

Facilitator interviews will explore two key areas: (1) their views about, and experiences of, providing care and support to the case study participant; and (2) the facilitator's more general experiences of recruiting into the trial and delivering the DAFNE*plus* intervention.

Data collection: facilitator interviews
The facilitators' interviews will be informed by topic guides, although each individual's interview will also be tailored to explore issues specific to the participant who forms the focus of the case study (being careful to ensure that patient confidentiality is not breached). Interviews will take place by telephone at a time most convenient to the facilitator and will be digitally recorded.

1.3 Data analysis
Each participant's four interviews will be read through repeatedly and cross-compared with particular attention being paid to continuities and changes in their diabetes self-management practices over time, and the reasons for these. To aid comparison and identify where behaviour change has happened and why, 'critical incidents' will be extracted and compared (a 'critical incident' comprises data where a behaviour/decision/experience is described in detail, including the contextual and antecedent factors leading up to it and the consequences arising from it [7,8]). To help identify reasons for behaviour change, maintenance and lapses, data from the facilitator interviews, and recordings of follow-up sessions and case reports will also be used to help interpret and provide context to analysis of participant interviews.

Facilitator interviews will be cross-compared to identify issues and experiences which cut across different accounts [5]. Depending on the findings of the fidelity work, facilitator interviews may also be analysed in clusters (e.g. facilitators belonging to

'adherent' vs 'non-adherent' sites), to better understand reasons for individual/site differences in how the DAFNE*plus* intervention was delivered.

Key objectives of the analysis of the participant interviews are to better understand the mechanisms of impact in order to: (a) inform analysis of the quantitative data collected for the process evaluation; and (b) aid interpretation of quantitative data collected for both the process and outcomes evaluations. Key objectives of the analysis of the facilitator interviews are to offer insights which might: (a) aid interpretation of the participant case study data; (b) help explain findings from the fidelity work; (c) aid interpretation of trial outcome data; and, (d) offer insights relevant to decision-making about the possible rollout of DAFNE*plus* following the trial.

A key area for reflection
It needs to be recognised that, by interviewing participants at four time-points, and because of the kinds of questions which will be asked, the qualitative study could, potentially, have an impact on how this small group of participants understand, engage with, and experience the DAFNE*plus* intervention. Care will be made to emphasise to participants that the qualitative study is separate to DAFNE*plus* and it is our intention to understand their experiences rather than to influence their behaviours. Whilst we may not be able to diminish the impact that this has on these participants, we hope that the overall impact will be minimal due to the small sample size potentially affected.

(2) Quantitative component

2.1 Overview
A longitudinal, questionnaire study design has been adopted to determine the impact of the RCT on: a) our primary psychological outcome (diabetes-specific quality of life), b) secondary psychological outcomes and c) for the quantitative aspect of the process evaluation. That is, to identify the mechanisms of change that predict glycaemic control and diabetes-specific quality of life.

All participants in the intervention (DAFNE*plus*) and control (DAFNE) arms of the RCT will be given questionnaires to complete at baseline (up to 4 weeks prior to commencing the course) and at course completion, 3, 6, 9, 12 months post-course (see section 7 and Table 1). At baseline, the point at which participants will be more motivated to participate (pre-trial), they will be asked to complete all outcome and process questionnaire measures. To reduce participant burden, at course completion, 3- and 9-months they will only be asked to complete process measures. At 6 and 12-months they will be asked to complete the primary and secondary outcome measures only. Participants will be given the option of completing the questionnaire packs online or as a hard copy. Our choice of questionnaires (see section 7), assessing different constructs, have been selected according to existing knowledge about their association with the trial's primary outcome (HbA1c) and diabetes-specific quality of life (primary psychological outcome), the results of the YOURSAY survey (unpublished), our former work with the DAFNE intervention, and based on the theoretical framework that underpins the new intervention development work and possible treatment mechanisms [9–11]. Brevity of the questionnaires and participant burden have also been a key consideration in our rationale for selection.

2.2 Analysis

The use of a repeated measures, longitudinal design will permit analysis of our primary and secondary psychological outcomes, as well as both the short- and long-term predictors and mediators of outcome (HbA1c and ADDQoL-15) using Structural Equation Modelling. SEM combines confirmatory and exploratory purposes. We will test our proposed model of the long-term predictors and mediators of outcome and then, if necessary, re-test this based on changes suggested by SEM modification indices [12]. The model will partially be informed by the qualitative work (described in section 1 above).

(3) Assessment of fidelity of delivery

3.1 Introduction

Behaviour change interventions are susceptible to variation in implementation, and are not always delivered as planned [13]. Intervention fidelity refers to the methodological strategies used to assess, monitor and enhance the integrity, that is, reliability and validity of behaviour change interventions [13]. The extent to which interventions are delivered as planned indicates internal and external validity, and needs to be known if the trial results are to be accurately interpreted and replicated. If fidelity is low, it is uncertain whether a change in outcome variables is due to the intended intervention, or to unknown factors that may have been added or omitted; alternatively, if no positive change is observed, it cannot be determined whether this is due to an inefficient intervention or a lack of intervention fidelity. This means that ineffective treatments risk being implemented and disseminated, and potentially effective treatments prematurely discarded [13].

The development of the content and structure of the DAFNE*plus* programme was informed by the Behaviour Change Wheel (BCW) framework [11]. The intervention's proposed functions are served by behaviour change techniques (BCTs), specified in the hierarchical Behaviour Change Technique Taxonomy v1 (BCTTv1; [14]), which are its 'active ingredients' [15]. The DAFNE*plus* intervention contains manual-specified BCTs as its active ingredients proposed to effect behaviour change (e.g. action planning, goal setting, and information on health consequences of the behaviour), together with principles for delivery specific to the DAFNE*plus* intervention that were identified during an expert consensus process (e.g. focus on the positives, emphasise individual autonomy). The fidelity analysis will involve assessment of the delivery of BCTs.

Fidelity of delivery of BCTs will also be assessed in the control arm of the trial (standard DAFNE) in order to identify any loss of treatment differentiation between the intervention and control arms as originally designed. Potentially loss of differentiation may result from low fidelity of delivery of additional content in the DAFNEplus programme, or additional content being delivered in the standard DAFNE programme, either unintentionally or as a result of contamination.

3.2 Aims and research questions

The aim is to explore the integrity of delivery of the DAFNE*plus* programme trialled in the RCT.

The research questions are:

- To what extent was the DAFNE*plus* programme delivered as specified in the protocols (course curriculum and follow-up scripts)? Specifically:

  - What proportion of manual-specified content (i.e. BCTs) was delivered by facilitators as intended during the programme sessions?
  - What additional, non-specified BCTs were delivered by facilitators?
  - How did the proportion of manual-specified content delivered differ across sessions and sites?
- What is the extent of treatment differentiation between the content of DAFNEplus and control (standard DAFNE) programmes delivered?

3.3 Methods

3.3.1 Design

A quantitative fidelity assessment, involving content analysis of intervention materials and transcripts of audio-recorded intervention sessions and provider self-rated fidelity checklists.

3.3.2 Observed fidelity of delivery assessment

The direct observation of fidelity via coding of session transcripts will provide an in-depth assessment of fidelity of delivery in a sub-sample of DAFNE*plus* and DAFNE courses.

Participants

Facilitators delivering either the DAFNE*plus* (intervention arm) or standard DAFNE (control arm) curriculum in 6 of the 14 participating sites will have their sessions recorded. It is assumed that each of these sites will have at least three facilitators delivering the DAFNE or DAFNE*plus* programmes (i.e. a minimum of 18 participants). Informed consent will be obtained from all participants at the selected sites (facilitators and patients). As part of the wider RCT., their participation in the programme during the course and follow-up sessions will be audio-recorded for training and research purposes.

Materials: Coding framework to assess observed fidelity of delivery

A coding framework will be developed to specify the BCTs to be delivered during the five face-to-face DAFNE*plus* days and the follow-up sessions, and the standard DAFNE course sessions, as specified in the facilitator manual. For each BCT the coding framework will include a definition, examples and criteria for potential operationalisation in the context of the programme.

Sampling and procedure

Six sites (2 control and 4 intervention) will be purposively sampled for audio recording of all sessions. Selection will be informed by variables such as facilitator experience, previous research activity and site activity levels.

Course sessions, and where applicable follow up sessions, delivered face-to-face, will be audio-recorded in both the intervention (i.e. DAFNE*plus*) arm and control arm (i.e. standard DAFNE) [6] at selected sites. Written informed consent for audio-recording sessions will be sought from all participants and facilitators. Participants will be reassured that transcripts of audio-recorded sessions will be fully anonymised to remove any personal or identifiable information. Facilitators will be supplied with a digital audio-recorder and instructions for operating it. Facilitators will audio-record sessions and upload recordings to the University of Sheffield secure server via Google

Drive which will be accessed by the study manager and authorised members of the research team. Transcription will be performed by an external transcription service and a confidentiality agreement will be put in place with the transcribers to protect participant's data.

Each DAFNE*plus* programme comprises circa 40 sessions (one 1:1 pre-course session, 35 group 'course' sessions and four 1:1 follow-up sessions per participant). Each standard DAFNE course comprises circa 35 group course sessions per participant. Sessions will therefore be purposively sampled for transcription and analysis across both arms, selected sites and courses. Courses will be sampled according to key variables, e.g. geographical location, and the timeline for the trial with earlier courses preferentially sampled due to staff resource. Sessions may be sampled for transcription according to theoretical underpinning of the intervention, and evidenced relation to the outcome.

Analysis

Sampled content of sessions in both the intervention and control arms will be first specified by applying the developed coding framework to the sample of selected course transcripts. Two researchers will independently read through the session transcript line-by-line, using the coding framework to identify and categorise BCTs present in the facilitator's speech. Each identified BCT and delivery principle will be rated as fully, partially or not delivered according to the coding framework definition and criteria. Illustrative examples will be extracted into the framework.

To assess and establish inter-coder reliability, the researchers will meet frequently in coding workshops at the outset of coding (e.g. initially after coding every transcript [16]). Approximately twenty percent of transcripts will be double coded. Inter-rater reliability will be assessed by percentage agreement [17]. Reasons for discrepancies will be discussed, and the coding framework developed accordingly. Following Hardeman et al. [16], a minimum level of 75% inter-coder agreement [18], described as 'high' [19,20] will be considered acceptable. After inter-coder reliability has been established, researchers will code the remainder of transcripts independently.

Fidelity of delivery will be assessed following the methods of Hardeman et al. [16] and Lorencatto et al. [17]. Each of the BCTs specified in the DAFNE*plus* programme (intervention arm) or standard DAFNE programme (control arm) curriculum/scripts will be listed in a checklist, together with details such as session number and facilitator participant number. The BCTs specified in the coding framework will be rated as: 1) fully present, 2) partially present, or 3) absent but should be present. The proportion of BCTs delivered as intended will be assessed by dividing the number of fully/partially present BCTs by the total number of intended BCTs. Established criteria will be applied to classify extent of observed fidelity of delivery [6]: if < 50% of intended content is delivered this will be classified as 'low' fidelity; 51-79% as 'moderate' fidelity, and 80-100%' as 'high fidelity'.

Sessions will be grouped into types based on topics where applicable (for example, the four sessions covering action planning would be grouped into one type). An 'intended content' checklist will be produced for each session, and the session transcript will only be compared to the checklist for the corresponding session or session type, rather than comparison against the full curriculum. Variation in fidelity

will be examined according to site and session type. Delivery of any additional content will also be examined by assessing the frequency of delivery of any non-specified BCTs. This will serve to identify adaptations made whilst delivering DAFNE*plus*.

Treatment differentiation will be assessed by comparing the content analyses of transcripts from the intervention (DAFNE*plus*) and control (standard DAFNE) sessions. BCTs that are fully/partially delivered in transcripts from both arms will be compared, and the proportion of BCTs delivered in both arms assessed, with a higher proportion of common BCTs delivered representing less treatment differentiation.

3.3.3 Self-reported fidelity of delivery

Participants
All facilitators delivering either the DAFNE*plus* (intervention arm) or standard DAFNE (control arm) curriculum/scripts in each of the 14 participating sites will provide data for the fidelity of assessment delivery. It is assumed that each site will have at least three facilitators delivering the DAFNE or DAFNE*plus* programmes (i.e. a minimum of at least 42 participants). Informed consent will be obtained from all participants (facilitators and patients) as part of the wider RCT.

Materials: Facilitator self-rated checklists
To obtain a global snapshot of fidelity across all DAFNE*plus* courses, including those that are not transcribed and included in the observed fidelity assessment, self-reported facilitator checklists will also be developed and administered to all sites (intervention and control). The checklist will include provision of key information and BCTs that are intended to be delivered (i.e. as specified in the pre-course session script, course curriculum and follow-up support scripts), and how confident and competent the facilitators felt delivering the session components. Facilitators will also be asked to record reasons for any components not being fully delivered. Different checklists will be developed for each session. Due to the dynamic nature of the intervention and curriculum development it is not possible to provide definitive and finalised versions of these checklists at this time: the checklists will be finalised following the coding of the final version of DAFNEplus[2].

Procedure
Facilitators will be asked to complete the checklist at the end of each session where possible, or by the end of each day. They will forward completed checklists to Sheffield University CTRU by the end of each day. Facilitators will rate the extent to which they feel they delivered the intervention components listed in the checklists, from 0 (not at all), 1 (partially) to 2 (fully delivered).

Analysis
The proportion of intended components rated as partially/fully delivered by the facilitators will be calculated. The same criteria will be applied to classify extent of fidelity as in the observed measurements: if < 50% of intended content is delivered

[2] We are submitting specimen checklists with this revised version of the protocol, these checklists are subject to change as detailed above. The curriculum will be subject to change up until the point of recruitment and even after this point there might be minor changes which would require modification of the fidelity checklists. A requirement to submit these checklists after each change would be a major burden on both the ethics committee and the research team and therefore we seek permission to revise checklists without further approval and will not submit additional checklists unless requested to do so.

this will be classified as 'low' fidelity; 51-79% as 'moderate' fidelity, and 80-100%' as 'high fidelity'. Variation in proportion of fidelity of delivery will be examined across: session types, facilitators, and courses.

There are well documented discrepancies between what healthcare providers report delivering and actually deliver [21]. Therefore, for the DAFNE*plus* courses where session transcripts have also been coded (as described above), self-reported and objectively verified practice will be directly compared in terms of the proportion of BCTs facilitators report delivering, and that which was identified during the content analysis.

References

1 Moore G, Audrey S, Barker M, *et al.* Process evaluation of complex interventions UK Medical Research Council (MRC) guidance. https://www.mrc.ac.uk/documents/pdf/mrc-phsrn-process-evaluation-guidance-final/ (accessed 30 Jan 2018).

2 Moore GF, Audrey S, Barker M, *et al.* Process evaluation of complex interventions: Medical Research Council guidance. *BMJ* 2015;**350**:h1258. doi:10.1136/bmj.h1258

3 May CR, Mair F, Finch T, *et al.* Development of a theory of implementation and integration: Normalization Process Theory. *Implement Sci* 2009;**4**:29. doi:10.1186/1748-5908-4-29

4 May C, Finch T. Implementing, Embedding, and Integrating Practices: An Outline of Normalization Process Theory. *Sociology* 2009;**43**:535–54. doi:10.1177/0038038509103208

5 Strauss AL, Corbin JM. *Basics of qualitative research : grounded theory procedures and techniques*. Sage Publications 1990.

6 Borrelli B. The assessment, monitoring, and enhancement of treatment fidelity in public health clinical trials. *J Public Health Dent* 2011;**71**:S52–63. doi:10.1111/j.1752-7325.2011.00233.x

7 Flanagan JC. Psychological Bulletin THE CRITICAL INCIDENT TECHNIQUE. *JULY* 1954;**51**.https://books.apa.org/pubs/databases/psycinfo/cit-article.pdf (accessed 30 Jan 2018).

8 Butterfield LD, Borgen WA, Amundson NE, *et al.* Fifty years of the critical incident technique: 1954-2004 and beyond. *Qual Res* 2005;**5**:475–97. doi:10.1177/1468794105056924

9 Cooke D, Bond R, Lawton J, *et al.* Modeling predictors of changes in glycemic control and diabetes-specific quality of life amongst adults with type 1 diabetes 1 year after structured education in flexible, intensive insulin therapy. *J Behav Med* 2015;**38**:817–29. doi:10.1007/s10865-015-9649-y

10 Michie S, Johnston M, Abraham C, *et al.* Making psychological theory useful for implementing evidence based practice: a consensus approach. *Qual Saf Heal Care* 2005;**14**:26–33. doi:10.1136/qshc.2004.011155

11 Michie S, van Stralen MM, West R. The behaviour change wheel: a new method for characterising and designing behaviour change interventions. *Implement Sci* 2011;**6**:42. doi:10.1186/1748-5908-6-42

12 Bentler PM. On tests and indices for evaluating structural models. *Pers Individ Dif* 2007;**42**:825–9. doi:10.1016/J.PAID.2006.09.024

13 Bellg AJ, Borrelli B, Resnick B, *et al.* Enhancing Treatment Fidelity in Health Behavior Change Studies: Best Practices and Recommendations From the NIH Behavior Change Consortium. *Heal Psychol* 2004;**23**:443–51. doi:10.1037/0278-6133.23.5.443

14 Michie S, Richardson M, Johnston M, *et al.* The Behavior Change Technique Taxonomy (v1) of 93 Hierarchically Clustered Techniques: Building an International Consensus for the Reporting of Behavior Change Interventions. *Ann Behav Med* 2013;**46**:81–95.

doi:10.1007/s12160-013-9486-6

15 Michie, S, Atkins, L, West R. *The behaviour change wheel: a guide to designing interventions*. London: : Silverback Publishing 2014.

16 Hardeman W, Michie S, Fanshawe T, *et al.* Fidelity of delivery of a physical activity intervention: Predictors and consequences. *Psychol Health* 2008;**23**:11–24. doi:10.1080/08870440701615948

17 Lorencatto F, West R, Bruguera C, *et al.* A method for assessing fidelity of delivery of telephone behavioral support for smoking cessation. *J Consult Clin Psychol* 2014;**82**:482–91. doi:10.1037/a0035149

18 Lombard M, Snyder-Duch J. Content Analysis in Mass Communication Assessment and Reporting of Intercoder Reliability. *Hum Commun Res* 2002;**28**:587–604.http://marketing.univie.ac.at/fileadmin/user_upload/lehrstuhl_marketing/Lehre/Lehrinhalte/09SS/Lombard_et_al__2002_-1.pdf (accessed 30 Jan 2018).

19 Cohen J. Weighted kappa: nominal scale agreement with provision for scaled disagreement or partial credit. *Psychol Bull* 1968;**70**:213–20.http://www.ncbi.nlm.nih.gov/pubmed/19673146 (accessed 30 Jan 2018).

20 Altman DG. *Practical statistics for medical research*. Chapman and Hall 1991.

21 Hogue A, Dauber S, Lichvar E, *et al.* Validity of Therapist Self-Report Ratings of Fidelity to Evidence-Based Practices for Adolescent Behavior Problems: Correspondence between Therapists and Observers. *Adm Policy Ment Heal Ment Heal Serv Res* 2015;**42**:229–43. doi:10.1007/s10488-014-0548-2
