# Supplementary material for: Protocol for a cluster randomised controlled trial of the DAFNEplus (Dose Adjustment For Normal Eating) intervention compared with 5x1 DAFNE: a lifelong approach to promote effective self-management in adults with type 1 diabetes
Source: BMJ Open. 2021 Jan 18;11(1):e040438. doi: 10.1136/bmjopen-2020-040438 (PMC7813353; doi:10.1136/bmjopen-2020-040438)
Supplement: Supplementary data [file bmjopen-2020-040438supp003.pdf]

Supplementary material 3 – Protocol for DAFNE*plus* Economic Evaluation

Aims and perspective

We will complete an economic evaluation as part of the study so that we are able to understand the cost-effectiveness of DAFNE*plus* compared to the standard DAFNE programme. The economic evaluation will follow guidance set by the National Institute for Health and Clinical Excellence for its Technology Appraisal process [1]. The analysis will take an NHS and personal social services perspective, measure health effects in quality adjusted life years (QALYs), discount future outcomes at 3.5% per annum and consider effects and costs over a lifetime time horizon. The primary analysis will be use long-term cost-effectiveness modelling, a secondary analysis will be an economic evaluation alongside the clinical trial (EEACT). The analysis population for all health economic analyses will consist of all participants in the DAFNEplus trial. A full Health Economic and Decision Modelling Analysis Plan (HEDMAP) will be written and circulated to the Trial Management Group and Programme Steering Committee before being signed-off.

Long-term cost-effectiveness modelling

In the long-term modelling exercise, the resulting evidence base will be incorporated into an updated Sheffield T1D Diabetes Policy Model [2]. This model has been used extensively in the evaluation of education and psychological interventions for people with T1D[3–6]. The time horizon of this analysis will be over each simulated individual's lifetime. As such, the long-term modelling will be considered as the primary health economic analysis. Demographic variables and some key resource use data (e.g. insulin use, contacts with NHS professionals) will be obtained from the trial data. The Sheffield T1D Diabetes Policy Model will be updated to use statistical models that estimate the clinical effects of DAFNE*plus* compared to DAFNE on HbA1c, the incidence of severe hypoglycaemia and the incidence of DKA. Two long-term modelling analyses will be conducted, the first will use the data collected by the one-year time point and will be submitted as part of the report to the NIHR on the DAFNE*plus* programme grant. This analysis will be updated after the two-year data collection is complete to incorporate the statistical analysis of the two-year follow up data. These statistical analyses of the clinical effects of DAFNE*plus* compared to DAFNE will be pre-specified in either the statistical analysis plan or the HEDMAP. The reporting of this evaluation will follow the Palmer *et al*[7] checklist for the reporting of model inputs to diabetes health economic studies.

Economic evaluation alongside the clinical trial

For the EEACT, we conduct the analysis in line with Ramsey *et al's* [8] recommendations for cost-effectiveness analysis alongside clinical trials. Specifically, we will collect data alongside the trial on intervention costs, associated healthcare resource use and a preference based utility measure: the EQ-5D-5L measure [9]. The intervention costing process will include training of educators, resource use, and adherence to structured follow up appointments, professional staff time and the technology component. A standard self-reported resource use questionnaire, used

previously in the DAFNE*plus* pilot (as well as the 5x1 DAFNE [10] and the REPOSE trials [11]), will ascertain NHS usage in terms of GP, community, outpatient, A&E and inpatients, as well as occurrence of DKA and hypoglycaemic events by level of severity. Unit costs will be taken from standard sources (NHS Reference Costs, British National Formulary, PSSRU). The standard self-reported resource use questionnaire and the EQ-5D-5L will be collected at baseline, 6 months and 12 months. Course costs (administrative and clinical) will be estimated using a bespoke questionnaire for completion by site staff. Our primary analysis will use the EQ-5D-5L valuation study to generate utility scores at baseline, course completion, 6 months and 12 months for each study participant [12]. There are on-going discussions about the valuation of the EQ-5D-5L, and NICE recently produced a position statement recommending that EQ-5D-5L data should be valued using mapping to the EQ-5D-3L and not the bespoke EQ-5D-5L value set [13,14]. Therefore our primary analysis will follow the most recent NICE guidance at the time of analysis, with the other valuation method been used in a sensitivity analysis. QALYs for each participant will be estimated by calculating the area under the curve defined by EQ-5D utility score, mortality and length of follow-up. The base case analysis will use the complete case data. In a scenario analysis, the missing data will be imputed. The time horizon of this analysis will be limited to the one-year time horizon of the trial. This evaluation will be considered as the secondary health economic analysis for two reasons: 1) The effects and costs of DAFNE*plus* may be incurred beyond the one-year trial time horizon (due to expected differences in the time to onset of diabetes related complications and potential maintenance of treatment effects beyond the trial period); and, 2) the DAFNEplus trial is not powered to detect differences in the incidence of long-term diabetes complications, as such the estimates of differences in the cost and QALYs between the two trial arms may be misleading.

Outcome measures and uncertainty analyses

In both the EEACT and the long term modelling the main outcome of interest will be the comparison of the incremental cost-effectiveness ratio (ICER) of DAFNE*plus* compared to DAFNE. The ICER will be compared to a maximum acceptable ICER of £20,000 per QALY gained, as this is the lower limit of the ICER range used by NICE to determine if an intervention is cost-effective [1]. Uncertainty in the ICER will be determined using: scenario analyses, subgroup analyses (pre-specified with the wider DAFNE*plus* team), probabilistic sensitivity analysis and expected value of information calculations. In particular, uncertainty in the cost-effectiveness of DAFNE*plus* as used in a wider rollout (compared to as utilised in the trial) and in subgroups of participants with a HbA1c less than 7.5% and greater than or equal to 7.5% will be explored in our scenario analyses.

References

1 National Institute for Health and Care Excellence. Guide to the methods of technology appraisal 2013 | Guidance and guidelines | NICE. https://www.nice.org.uk/process/pmg9/chapter/foreword (accessed 30 Jan 2018).

2 Thokala P, Kruger J, Brennan A, *et al.* Assessing the cost-effectiveness of Type 1 diabetes interventions: the Sheffield Type 1 Diabetes Policy Model. *Diabet Med* 2014;**31**:477–86. doi:10.1111/dme.12371

3 Kruger J, Brennan A, Thokala P, *et al.* The cost-effectiveness of the Dose Adjustment for Normal Eating (DAFNE) structured education programme: an update using the Sheffield Type 1 Diabetes Policy Model. *Diabet Med* 2013;**30**:1236–44. doi:10.1111/dme.12270

4 Heller S, Lawton J, Amiel S, *et al.* Improving management of type 1 diabetes in the UK: the Dose Adjustment For Normal Eating (DAFNE) programme as a research test-bed. A mixed-method analysis of the barriers to and facilitators of successful diabetes self-management, a health economic analysis, a cluster randomised controlled trial of different models of delivery of an educational intervention and the potential of insulin pumps and additional educator input to improve outcomes. *Program Grants Appl Res* 2014;**2**:1–188. doi:10.3310/pgfar02050

5 Kruger J, Pollard D, Basarir H, *et al.* Incorporating Psychological Predictors of Treatment Response into Health Economic Simulation Models. *Med Decis Mak* 2015;**35**:872–87. doi:10.1177/0272989X15590143

6 Pollard DJ, Brennan A, Dixon S, *et al.* Cost-effectiveness of insulin pumps compared with multiple daily injections both provided with structured education for adults with type 1 diabetes: A health economic analysis of the Relative Effectiveness of Pumps over Structured Education (REPOSE) randomised controlled trial. *BMJ Open* 2018;**8**:e016766. doi:10.1136/bmjopen-2017-016766

7 Palmer AJ, Si L, Tew M, *et al.* Computer Modeling of Diabetes and Its Transparency: A Report on the Eighth Mount Hood Challenge. *Value Heal* 2018;**21**:724–31. doi:10.1016/j.jval.2018.02.002

8 Ramsey SD, Willke RJ, Glick H, *et al.* Cost-Effectiveness Analysis Alongside Clinical Trials II—An ISPOR Good Research Practices Task Force Report. *Value Heal* 2015;**18**:161–72. doi:10.1016/j.jval.2015.02.001

9 Herdman M, Gudex C, Lloyd A, *et al.* Development and preliminary testing of the new five-level version of EQ-5D (EQ-5D-5L). *Qual Life Res* 2011;**20**:1727–36. doi:10.1007/s11136-011-9903-x

10 Elliott J, Lawton J, Rankin D, *et al.* The 5x1 DAFNE study protocol: a cluster randomised trial comparing a standard 5 day DAFNE course delivered over 1 week against DAFNE training delivered over 1 day a week for 5 consecutive weeks. *BMC Endocr Disord* 2012;**12**:28. doi:10.1186/1472-6823-12-28

11 REPOSE Study Group TRS. Relative effectiveness of insulin pump treatment over multiple daily injections and structured education during flexible intensive insulin treatment for type 1 diabetes: cluster randomised trial (REPOSE). *BMJ* 2017;**356**:j1285. doi:10.1136/BMJ.J1285

12 Devlin NJ, Shah KK, Feng Y, *et al.* Valuing health-related quality of life: An EQ-5D-5L value set for England. *Health Econ* Published Online First: 22 August 2017. doi:10.1002/hec.3564

13 National Institute for Health and Care Excellence. NICE's position statement on EQ-5D-5L NATIONAL INSTITUTE FOR HEALTH AND CARE EXCELLENCE Position statement on use of the EQ-5D-5L valuation set.

https://www.nice.org.uk/Media/Default/About/what-we-do/NICE-guidance/NICE-technology-appraisal-guidance/eq5d5l_nice_position_statement.pdf (accessed 30 Jan 2018).

14 van Hout B, Janssen MF, Feng Y-S, *et al.* Interim Scoring for the EQ-5D-5L: Mapping the EQ-5D-5L to EQ-5D-3L Value Sets. *Value Heal* 2012;**15**:708–15. doi:10.1016/j.jval.2012.02.008
