# Supplementary material for: Protocol for a cluster randomised controlled trial of the DAFNEplus (Dose Adjustment For Normal Eating) intervention compared with 5x1 DAFNE: a lifelong approach to promote effective self-management in adults with type 1 diabetes
Source: BMJ Open. 2021 Jan 18;11(1):e040438. doi: 10.1136/bmjopen-2020-040438 (PMC7813353; doi:10.1136/bmjopen-2020-040438)
Supplement: Supplementary data [file bmjopen-2020-040438supp005.pdf]

Supplementary Material 5 – DAFNE*plus* SAP

Project Title: A Cluster Randomised Controlled Trial (CRCT) of the DAFNE*plus* (Dose for Adjustment for Normal Eating) intervention: A lifelong approach to promote effective self-management in adults with type 1 diabetes

Statistical Analysis Plan

Version 1 17th December 2019

Based on Protocol Version 6.0, dated 9th September 2019

REC: 18/SW/0100 ISRCTN: 42908016 Sheffield CTRU Job no. J13-003

Authored by

___________________________________ ___/___/____

Nikki Totton Date
Statistician, CTRU, University of Sheffield

Approved by

___________________________________ ___/___/_____

Mike Bradburn Date
Senior Statistician, CTRU, University of Sheffield

___________________________________ ___/___/_____

Simon Heller Date
Chief Investigator, Sheffield Teaching Hospitals NHS Foundation Trust

___________________________________ ___/___/_____

Simon Griffin Date
DAFNE*plus* TSC Chair, University of Cambridge

Roles and Responsibilities

This SAP was initially drafted by Henry Nanji (previous DAFNE*plus* trial statistician) and updated by Nikki Totton (DAFNE*plus* trial statistician), with input from Mike Bradburn (senior statistician) and the trial team.

SAP Revision History

Version Number	Revision Date	Timing Within Trial	Description/Justification

Coates E, *et al. BMJ Open* 2021; 11:e040438. doi: 10.1136/bmjopen-2020-040438

List of Abbreviations and Definitions of Terms

**ADDQoL-14**	Audit of Diabetes Dependent Quality of Life (14 items)
**AE**	Adverse Event
**BMI**	Body Mass Index
**CIDS**	Confidence in Diabetes Scale
**CRF**	Case Report Form
**CTRU**	Clinical Trials Research Unit
**DAFNE**	Dose Adjustment For Normal Eating
**DIDP**	DAWN Impact of Diabetes Profile
**DKA**	Diabetic Ketoacidosis
**DME-Q**	Diabetes Management Experience Questionnaire
**DSRQ**	Diabetes Strengths and Resilience Questionnaire
**eGFR**	Estimated Glomerular Filtration Rate
**EQ-5D-5L**	EuroQol - 5 Dimensions - 5 Levels
**GCP**	Good Clinical Practice
**GLM**	Generalised Linear Model
**HASMID**	Health And Self-Management In Diabetes
**HbA1c**	Glycated Haemoglobin (Haemoglobin A1c)
**HCS**	Hypoglycaemia Confidence Scale
**HDL**	High-Density Lipoprotein
**HFS**	Hypoglycaemia Fear Survey
**ICC**	Intra-class Correlation Coefficient
**IQR**	Inter Quartile Range
**IRR**	Incidence Rate Ratio
**ISRCTN**	International Standard Randomised Controlled Trial Number
**ITT**	Intention To Treat
**LDL**	Low-Density Lipoprotein
**LRT**	Likelihood Ratio Test
**MD**	Mean Difference
**MDI**	Multiple Daily Injections
**NHS**	National Health Service
**OR**	Odds Ratio
**PAID**	Problem Areas In Diabetes
**PP**	Per Protocol
**QoL**	Quality of Life
**RCT**	Randomised Controlled Trial
**REC**	Research Ethics Committee
**SAE**	Serious Adverse Event
**SCB-T1D**	Self-Care Behaviours: Type 1 Diabetes
**SD**	Standard Deviation
**SOP**	Standard Operating Procedure
**SRQ-T1D**	Self-Regulation Questionnaire
**SUS**	System Usability Score
**T1D**	Type 1 Diabetes
**TMG**	Trial Management Group
**TSC**	Trial Steering Committee
**VAS**	Visual Analogue Scale
**W-BQ28**	Well-Being Questionnaire 28

DAFNE*plus* Statistical Analysis Plan
Version 1 17th December 2019

Contents

1 Introduction 4
1.1 Background and Rationale 4
1.2 Objectives 4
2 Trial Methods 5
2.1 Trial Design 5
2.2 Randomisation and Blinding 5
2.3 Sample Size 5
2.4 Trial Framework 6
2.5 Trial Monitoring and Management 6
2.6 Interim Analysis and Stopping Rules 6
2.7 Timing of Final Analysis 6
2.8 Timing of Outcome Assessments 7
3 Statistical principles 8
3.1 Confidence Intervals and P Values 8
3.2 Adherence and Protocol Deviations 8
3.3 Analysis Populations 8
4 Screening, recruitment, demographics and withdrawal 9
4.1 Eligibility Criteria 9
4.2 CONSORT 10
4.3 Withdrawal of Participants 11
4.4 Baseline Characteristics 11
5 Outline of Statistical AnalysIs 13
5.1 Outcome Measures 13
5.1.1 Primary Outcome 13
5.1.2 Secondary Outcomes - Biomedical 13
5.1.3 Secondary Outcomes - Psychological 14
5.1.4 Secondary Outcomes - Process Measures 15
5.2 Analysis of Primary Outcome 16
5.2.1 Model Checking 17
5.3 Sensitivity Analyses of the Primary Outcome 17
5.4 Subgroup Analyses 17
5.5 Handling Missing Data 18
5.6 Analysis of Secondary Outcomes - Biomedical 19
5.6.1 HbA1c 19
5.6.2 Weight, BMI, Blood Pressure and Lipids 19
5.6.3 Severe Hypoglycaemia and DKA 19
5.6.4 Model Checking 20
5.7 Analysis of Secondary Outcomes - Psychological 20
5.8 Safety Outcomes 20
5.9 Statistical Software 20
6 References 21
6.1 Documents 21
6.2 Publications 21

Coates E, *et al. BMJ Open* 2021; 11:e040438. doi: 10.1136/bmjopen-2020-040438

1 INTRODUCTION

1.1 Background and Rationale

The successful management of Type 1 Diabetes (T1D) requires those affected (>300,000 adults in the UK) [1] to keep their glucose levels sufficiently close to normal to avoid long-term complications [2]. In this condition, unlike type 2 diabetes, there is an absolute insulin deficiency, and so insulin must be injected subcutaneously, and tablet therapy is not possible. Preventing complications depends upon an individual's ability to prevent hyperglycaemia (high blood glucose levels) by self-managing their condition. This is done by calculating precise insulin doses based on accurate estimations of food intake before every meal using frequent blood glucose measurements, and accounting for fluctuations in physical activity, illness, stress and hormones. Hypoglycaemia (low blood glucose levels), if severe, can result in acute cognitive impairment, confusion, collapse and injury, coma or even death [3].

Dose Adjustment For Normal Eating (DAFNE) is a clinical education programme run within the National Health Service (NHS), designed to teach and improve self-management skills in flexible intensive insulin therapy to improve both glucose control and quality of life in adults with T1D. It is a five-day training course for adults with T1D, delivered in small groups. The DAFNE*plus* programme grant has modified the existing DAFNE curriculum to incorporate techniques for initiating and sustaining behaviour change, structured follow-up support, and digital information communication technology.

1.2 Objectives

The primary objective of the trial is to:

1. Assess the effects of the intervention on glycaemic control as measured by glycated haemoglobin (HbA1c) at 12 months.

The secondary objectives of the trial are to:

1. Assess the medium term effect of the intervention on glycaemic control, as measured by HbA1c, using data at 6 months,
2. Assess the effects of the intervention on the diabetes-specific quality of life,
3. Assess the effects of the intervention on diabetes distress and other biomedical outcomes (severe episodes of hypoglycaemia, diabetic ketoacidosis, weight, body mass index, blood pressure and lipids),
4. Undertake a mixed methods process evaluation to aid understanding of the Randomised Controlled Trial (RCT) findings, and to inform decision making about the implementation of DAFNE*plus* in clinical care post-trial,
5. Assess fidelity of delivery of the DAFNE*plus* intervention,
6. Undertake a health economic analysis to determine the cost-effectiveness of DAFNE*plus* versus standard DAFNE.

Objectives 4, 5 and 6 under secondary objectives will not be considered as part of this SAP and will be dealt with separately to the main trial analysis.

2 TRIAL METHODS

2.1 Trial Design

The trial will use a pragmatic, parallel group, cluster randomised (1:1 allocation) controlled design involving 14 sites. Centre randomisation is required rather than individual since 'contamination' of the control arm may occur if educators are trained in DAFNE*plus* (intervention) and still are required to deliver standard DAFNE (control) [4]. Potential participants are identified by local diabetes clinicians and will use standard criteria for referral to DAFNE.

Participants recruited at control centres will receive treatment as usual and will attend the DAFNE course one day a week, over five consecutive weeks. A bolus calculator will be provided to support the calculation of insulin dose, but there will be no structured follow-up appointment beyond those provided in usual care.

Participants in centres allocated to the intervention will attend the DAFNE*plus* course one day a week, over five consecutive weeks, which includes the use of technology to transmit and display blood glucose data to support pattern recognition and interpretation. A bolus calculator to support insulin dose calculations will be provided and up to five structured follow-up appointments are offered in the 12 months after the course.

Further details on the trial design can be found in the protocol.

2.2 Randomisation and Blinding

Following ethical approval, all participating centres were randomised on a 1:1 basis to control (standard DAFNE courses) or the intervention arm (DAFNE*plus* course). In order to balance the centres within the two arms, a covariate constrained approach [5] was adopted matching the centres on the number of patients, number of educators and the total number of previous DAFNE courses delivered by the centre as stratification variables. Due to the nature of the intervention, The University of Sheffield Clinical Trials Research Unit (CTRU) in-house randomisation system (SCRAM) was not applicable and so in line with Standard Operating Procedure (SOP) ST007, a randomisation guidance document detailing the randomisation procedure has been written. The random allocation was conducted by the trial statistician using STATA [6] and therefore no outcome data which is split by treatment group will be seen by the statistician until the trial is complete to minimise bias. Further details on the randomisation and unblinding can be found in the protocol.

2.3 Sample Size

2.3.1 Original Sample Size

It is expected that there will be 882 patients referred for DAFNE courses within the 15-month recruitment window and of these, it is expected that 75% (662 patients) will be recruited, equivalent to 47 participants at each of the 14 centres. Based on data from current DAFNE courses, a further 25% are expected not to meet the primary analysis population criteria of a baseline HbA1c greater than 7.5%, leaving 497 participants. Finally, we anticipate 15% of participants to be lost to follow-up by the 12-month stage, therefore giving a primary analysis population of 422 patients. Taking into account a

design effect, due to the cluster design of the trial, with an Intra-class Correlation Coefficient (ICC) of 1.5% (from previous DAFNE data) and 30 patients per cluster (422 patients over 14 centres) the design effect is 1.435 leaving the effective total sample size of 294 participants (147 per arm).

Using a two-sample comparison of mean HbA1c at the 12-month follow-up with 2-sided alpha of 5%, a correlation of 0.5 between baseline and final values and a standard deviation of 1.45 (from previous DAFNE data), the trial sample gives 92% power to detect a 0.5% difference in HbA1c between the two treatment groups.

2.3.2 Updated Sample Size

The original calculations (i.e. worked backwards from the expected number of recruits) gave a power of 92.7%. The team discussed different options, but in light of the difficulties in enlisting new centres and therefore now 13 centres not 14, it was agreed by our Trial Steering Committee to continue with the original planned recruitment per centre, with 6 interventions and 7 control. Therefore, reducing the sample size solely in the intervention arm. This results in a power of 90.4% with a small imbalance between the two arms (ratio 1:1.67) with a reduced sample size of 615 (instead of 662). As this trial is cluster randomised and provided through courses, there was always likely to be some imbalance between the two treatment arms which is out of our control.

2.4 Trial Framework

The primary aim of this trial is to conduct a superiority cluster RCT comparing the new DAFNE*plus* intervention to the existing DAFNE to detect a minimum clinically significant difference of 0.5% in HbA1c between the two groups after 12 months.

2.5 Trial Monitoring and Management

In compliance with Sheffield CTRU's SOPs, the following committees will be established to govern the overall conduct and supervision of the trial:

- Trial Steering Committee (TSC)
- Trial Management Group (TMG)

The trial will be supervised on a day to day basis at Sheffield CTRU by the Trial Manager with supervision from the Chief Investigator and a Senior Trial Manager.

2.6 Interim Analysis and Stopping Rules

There are no interim analyses or early stopping planned for this trial, hence no stopping rules are applicable.

2.7 Timing of Final Analysis

The final analysis will take place after the last participants have completed their 12-month follow-up visit. All data will be analysed collectively at this time point. A further study will be completed in the future to complete analysis of data collected at the 24-month follow-up, but this is not included within this SAP and will be outlined separately.

2.8 Timing of Outcome Assessments

Table 1 below shows the biomedical and psychological outcome measures and the different time-points outcomes are measured. A detailed description of the outcome assessment is found under section 5.

Table 1: Outcomes measures within the trial and time points they will be collected at

Outcome Measure	Baseline	6 Months Post Course	12 Months Post Course
**Clinical Outcomes**			
Demographics[1]	x		
HbA1c	x	x	X
Severe Hypoglycaemic Episodes	x	x	x
Diabetic Ketoacidosis (DKA) Episodes	x	x	x
Body Mass Index (BMI)	x	x	x
Blood Pressure	x		x
Lipids (HDL, LDL)[2]	x		x
**Psychological Outcomes**			
ADDQoL-15 (Diabetes-specific quality of life)	x	x	x
DIDP (Diabetes-specific quality of life)	x	x	x
PAID-11 (Diabetes Distress)	x	x	x
W-BQ28 (Diabetes-Specific Wellbeing)	x	x	x
HFS-11 (Fear of Hypoglycaemia)	x	x	x
Gold Score (Hypoglycaemia awareness)	x	x	x
**Health Economic Measures**			
HASMID (Health Status)	x	x	x
EQ-5D-5L (Health Status)	x	x	x
**Process Measures**			
DSRQ	x		
SCB-T1D	x		
Usability Score			
DME-Q	x		
HCS	x		
Beliefs about Consequences	x		
SRQ-T1D	x		

1: Detail description of demographic characteristics are found under section 4.4

Coates E, *et al. BMJ Open* 2021; 11:e040438. doi: 10.1136/bmjopen-2020-040438

2: HDL=High Density Lipoprotein, LDL=Low Density Lipoprotein

3 STATISTICAL PRINCIPLES

This statistical analysis plan (SAP) is written in conjunction with the International Conference of Harmonisation topic E9 [7], applicable Standard Operating Procedures (SOP) from the Sheffield Clinical Trials Research Unit (CTRU) (ST001 and ST006).

3.1 Confidence Intervals and P Values

All statistical tests will be completed at the 5% significance level and estimates of the treatment effect will be reported with their associated 95% confidence intervals. All tests completed will be two-sided. The results of the trial are focussed on the primary endpoint (HbA1c at 12 months) so adjustment for multiple testing and control of the type 1 error rate is required.

3.2 Adherence and Protocol Deviations

Adherence to the standard DAFNE course within the control group is defined as:

- Attending a minimum of four of the five days within the course which must include the first two days.

Adherence to the DAFNE*plus* course within the intervention group is defined as:

- Attending a minimum of four of the five days within the course which must include the first two days *AND*
- Attending at least three of the five follow-up sessions (this can be any three sessions)

Adherence to the courses will be presented as the number and percentage of participants in each arm of those that adhered. Additionally, for the intervention group the number and percentage of participants that adhered to each of the two adherence requirements will be presented separately to show which, if either, of these are more prominent.

In the DAFNE*plus* trial, any intended failure to adhere to the protocol will be classed as protocol violation and may be minor or major while any unintended (non-serious) departures from the protocol would be considered as protocol deviations and all these will be reported.

Attendance will be captured on case report forms (CRFs) when participants attend the course. Participants who failed to meet this criterion will be classed as having a major protocol deviation.

The number (and percentage) of patients with major and minor protocol deviations will be summarised by treatment group with details of type of deviation provided. No formal statistical testing will be undertaken between the two groups.

3.3 Analysis Populations

The primary analysis set will be that defined in Intention To Treat (ITT) on the primary outcome. Additional analysis populations, such as Per Protocol (PP), will be used as sensitivity analyses. Table 2 defines each of the analysis sets.

Table 2 Definition of the analysis set

Analysis Set	Outcomes	Participant Inclusion Criteria
**Primary ITT**	Primary outcome only	All consented participants, analysed according to their centre's randomisation regardless of their adherence to the entry criteria, intervention received, subsequent withdrawal or deviation from the protocol unless they have explicitly requested that their data be removed [7]. In addition, participants must have a baseline HbA1c more than 7.5%.
**Full ITT**	Secondary outcomes and as a sensitivity analysis for the primary outcome	All consented participants, analysed according to their centre's randomisation regardless of their adherence to the entry criteria, intervention received, subsequent withdrawal or deviation from the protocol unless they have explicitly requested that their data be removed [7].
**PP**	Primary outcome only	All consented participants excluding those who didn't adhere to the assigned intervention as defined by section 3.2.

4 SCREENING, RECRUITMENT, DEMOGRAPHICS AND WITHDRAWAL

4.1 Eligibility Criteria

Centre eligibility:

- Adult diabetes centre currently delivering DAFNE
- At least three DAFNE educators trained in delivering the five-week model of DAFNE
- Delivery of sufficient DAFNE courses per year to recruit the trial sample.

Participant eligibility:
Inclusion and exclusion criteria for participants are outlined in Table 3.

Table 3: Patient eligibility criteria

Inclusion Criteria	Exclusion Criteria
Age ≥18 years	HbA1c >12%
Diagnosis of type 1 diabetes for ≥6 months or post-honeymoon	Current use of continuous subcutaneous insulin infusion (CSII) pump therapy
Prepared to undertake multiple daily injections (MDI) therapy and frequent self-monitoring of blood glucose	Serious diabetes-related complications (e.g. blindness, renal dialysis), or other serious co-morbidities (e.g. psychosis, diagnosed eating disorder)
Available to attend all sessions	Unable to hear/speak/understand/read/write in English

Investigator has confidence that the patient is capable of adhering to all the trial protocol requirements	Previous participation in standard DAFNE course less than 5 years before proposed trial enrolment date
	Unable to give informed consent.

4.2 CONSORT

Using guidelines from the CONSORT statement [8], the summaries outlined Table 4 will be calculated in order to construct a CONSORT flowchart. Data will be presented overall and by treatment arm to show if any differences are present due to the sites treatment allocation.

Table 4: CONSORT Summary

**Screening Data**	• Number of participants assessed for eligibility at screening<br>• Number ineligible including reasons<br>• Number eligible but declined to participate including reasons
**Recruitment Data**	• Number of participants consented and recruited<br>• Number and percentage of those who attended all five sessions within either DAFNE/DAFNE*plus* course<br>• Number and percentage of those who completed the primary outcome (HbA1c) at 6 and 12 months follow-up
**Lost to Follow Up/Withdrawal Data**	• Number and percentage of those consented who dropped out and withdrew before the course<br>• Number and percentage of those consented who dropped out and withdrew after completing the course but before the 6-month follow-up<br>• Number and percentage of those consented who dropped out and withdrew after the 6-month follow up but before the 12-month follow-up
**Analysis Population Data**	• Number of those included in primary ITT set at 6 and 12 months follow-up<br>• Number of those included in full ITT set at 6 and 12 months follow-up<br>• Number of those included in PP set at 12 months follow-up

4.3 Withdrawal of Participants

Details of potential reasons for withdrawal are found in the protocol and summaries of these reasons plus any additional reasons found within the trial will be presented overall and split by treatment arm. Withdrawal numbers will also be summarised dependent on if the participant has withdrawn from the intervention, but continue with follow-up, withdrawal from follow-up but allow data collected to date to be used, withdrawal for all data collected to date to be used, or lost to follow up.

4.4 Baseline Characteristics

Baseline characteristics will be summarised at both the centre and participant level to assess the balance between the two treatment arms.

At the centre level, the stratification variables used (number of patients, number of educators and the total number of previous DAFNE courses delivered) within the randomisation will be presented by treatment arm to evaluate the balance between centres.

At the participant level, the variables shown in Table 5 as captured at baseline will be presented overall and by treatment arm. Categorical variables will be presented using counts and percentages, continuous variables will be presented with means and standard deviations or median and inter-quartile ranges as appropriate. No statistical significance testing will be used to test baseline imbalances between the two groups but any noteworthy differences will be descriptively reported.

Table 5: Baseline variables

**Demographics**	
	Age (Years)
	Gender
	Ethnicity
	Highest qualification
**Medical History**	
	Duration of diabetes (Years)
	Previously attended a DAFNE course
	Pregnancy since diabetes
	Current pregnancy, if yes gestation (weeks)
	Use of lipid lowering medication
	Use of antiplatelet agent
	Use of medication for depression
	Smoking status, if yes no of cigarettes per day
	Physical activity levels
	Complications – conditions and events (list as applicable)
	Quick acting insulin (average daily dose, number of injections per day and type)
	Background insulin (average daily dose, number of injections per day and type)
	Pre-mixed insulin (average daily dose, number of injections per day and type)
	Use of ratios
	Presence of Lipohypertrophy
	Number of blood glucose test performed (last 2 weeks)
	Use of CGM, method and length of use (for Libre only – how it is funded and how it's being used)
	Use of apps
	Severe hypoglycaemic episode in the last year
	Number of hypoglycaemic episodes that were unable to be treated themselves
	Number of hypoglycaemic episodes that required paramedic assistance
	Number of hypoglycaemic episodes that required A&E attendance
	Number of hypoglycaemic episodes that required hospital admission
	Blood glucose of hypoglycaemia
	Admissions due to DKA (ever and in last year)
**Labs and Vital Signs**	
	BMI (kg/m2)
	Blood pressure (mmHg)
	HbA1c (mmol/mol)
	Creatinine (µmol/L)
	Albumin-creatinine (mg/mmol)
	Cholesterol (mmol/L)
	Triglycerides (mmol/L)
	High Density Lipoprotein (HDL) cholesterol (mmol/L)

If there are any issues with partial dates in the database, the following approaches will be used to deal with them and therefore still allow derived time variables such as duration of diabetes to be calculated with sufficient precision:

If only year is available ("YYYY"), replace with "01/07/YYYY" or
If only month and year are available ("MM/YYYY"), replace with "15/MM/YYYY".

5 OUTLINE OF STATISTICAL ANALYSIS

Continuous variables will be summarised and presented by treatment group and overall as follows:

a) Mean and standard deviation (SD) for normal distribution
b) Median, Inter-Quartile Range (IQR), minimum and maximum for asymmetrical distribution

Categorical variables will be summarised and presented by treatment groups as the number of observations and proportion in each category and overall.

5.1 Outcome Measures

5.1.1 Primary Outcome

The primary outcome is glycaemic control defined as HbA1c, the primary endpoint refers to this data at 12-months but this will also be collected at baseline and 6-months (secondary endpoint). HbA1c is collected in mmol/mol but will be presented as a percentage. In order to convert between the two, the following calculation will be used [30]:

$$\text{HbA1c (\%)} = \text{HbA1c (mmol/mol)}/10.929 + 2.15$$

5.1.2 Secondary Outcomes - Biomedical

As an extension to the primary outcome, a secondary binary outcome to represent successful glycaemic control [9] will be calculated using the HbA1c data at 6- and 12-months. This outcome identified whether a participant achieved either a:

1) HbA1c $\leq$7.5% (58 mmol/mol) or
2) Reduction in HbA1c of ≥0.5% (≥5.5 mmol/mol).

Patient's BMI will be calculated using height and weight data at baseline, 6- and 12-months. Note – height is only collected once at baseline and used throughout. Weight can be collected either in kilograms (kg) or stones (st) and pounds (lb) and if collected in stone a conversion to kg will be completed using the following formula:

$$\text{Weight (kg)} = \text{Weight (st)}/0.15747 + \text{Weight (lb)}/2.2046$$

Similarly, height can be collected in metres (m) or feet (ft) and inches (in) and the following formula will be used to convert all to m:

$$\text{Height (m)} = \text{Height (ft)}/3.2808 + \text{Height (in)}/0.0254$$

BMI will then be calculated using the following formula:

$$\text{BMI (kg/m}^2\text{)} = \text{Weight (kg)} / \text{Height (m)}^2$$

Episodes of severe hypoglycaemia (as defined by the American Diabetes Association [9]), and incidence of DKA will be collected at baseline, 6-months and 12-months. Both will collect the number since the last visit.

Two different measures of lipids will be presented, high- and low-density lipoprotein which will be measured in mmol/L.

5.1.3 Secondary Outcomes - Psychological

- Audit of Diabetes-Dependent Quality of Life-15 (ADDQoL-15)

ADDQoL-15 is a questionnaire to measure the impact of diabetes and its treatment on a participant's QoL [10], [11]. It contains two overview items and 15 diabetes-specific items that relate to different aspects of life. Each has two parts, an impact score and an importance score. Each impact score is scored from -3 (very much better) to 1 (worse) and each importance score is from 3 (very important) to 0 (not at all important). These are then multiplied together to get a weighted impact score for each domain with -9 representing the maximum negative impact and 3 being the maximum positive impact. The average weighted impact (AWI) is then calculated as a mean of the weighted impacts for each domain.

If either the impact score or the importance score is missing, then the domain score cannot be computed and will not be included within the AWI. In the first instance, an AWI will only be calculated is all domain scores are available.

- DAWN Impact of Diabetes Profile (DIDP)

The participant's diabetes-specific quality of life will be assessed using the DIDP [12]. This consists of seven-items which investigates the impact diabetes has on different aspects of the participant's life. Each item is scored from 1 (very positive impact) to 7 (very negative impact), the composite score is the mean of all available responses, it ranges from 1-7 with lower scores indicating a greater positive impact. The percentage score is the composite score divided by 7, again lower percentages indicate greater positive impact.

- The Problem Areas in Diabetes

The Problem Areas in Diabetes (PAID) [13], [14] is a self-reported questionnaire that describe negative emotions related to diabetes (e.g. fear, anger, frustration) to assess diabetes distress. The short version, which will be used in this trial, consists of 11 items. Each question has five possible answers with a value from 0 to 4, with 0 representing “no problem” and 4 “a serious problem”. The overall score is the sum of all questions and ranges from 0 (best) to 44 (worst). A score of 18+ indicates severe diabetes distress.

- Diabetes-specific Positive Well-being

Diabetes-Specific Positive Well-being will be measured using the specific subscale of the Well Being Questionnaire (W-BQ28) [15]. This questionnaire consist of four subscales each with four possible responses used to measure diabetes-specific well-being. Each item is scored from 0 (not at all) to 3 (all the time). The scores for this subscale are summed to get a total score ranging from 0-12 with higher scores representing more positive well-being.

- Fear of hypoglycaemia (HFS-II Short Form)

This 11-item questionnaire assesses the level of fear amongst people with diabetes [16] and contains five behavioural items and six worry items taken from the original full measure. Each item is scored

on a five-point Likert scale (0=never to 4=almost always) and a total score for the behaviour and worry subscales are calculated by summing the scores.
For missing values on the questionnaire, participant mean score will be imputed for missing values if at least nine of the questions have been completed.

- Health Status.

Health status will be measured using EQ-5D-5L [17] questionnaire which is a self-reported outcome measure which aims to assess the general health-related quality of life of the participant. It consists of five dimensions measure mobility, self-care, usual activity, pain/discomfort and anxiety/depression with each dimension having five possible responses. Participants also rate their overall health on the day of the interview on a 0–100 visual analogue scale with 0 = worst imaginable health state and 100 = best imaginable health state.

Scoring of EQ-5D-5L will be either scored using the relevant value set [18] or by mapping onto the EQ-5D-3L depending on the most up to date method at the time. The EQ-5D-5L health utility will not be calculated if any of the five dimensions are missing.

- Health and Self-Management in Diabetes

Health status will also be assessed using HASMID [19] assessment tool. It consists of ten items each with four possible responses. Responses are scored from zero to three with higher scores indicating little or no impact upon health related QoL. The overall questionnaire is a sum of all question scores and scored from 0 to 30 with higher scores indicating good health related QoL and a lower score indicating poor health-related quality of life. The scores are then used to calculate utility scores, the HASMiD health utility will not be calculated if any of the component ten questions are missing.

- Hypoglycaemia awareness

Awareness of hypoglycaemia will be measured using the Gold score questionnaire [20] which is a 1 item questionnaire consisting of a seven-point scale where one represent ‘always aware of the onset of hypoglycaemia’ and seven ‘never aware of the onset of hypoglycaemia’.

5.1.4 Secondary Outcomes - Process Measures

- Diabetes Strengths and Resilience Questionnaire (DSRQ)

Adaptive behaviours and attitudes associated with overcoming challenges with diabetes management will be measured using the DSRQ [21]. The questionnaire consists of 12 items each scored from 1 (Never) to 5 (Always), the scores are summed to produce the total score which ranges from 12-60. Higher scores indicate perception of having greater T1D strengths.

- Self-Care Behaviours: Type 1 Diabetes (SCB-T1D)

Fifty items from the Self-Care Behaviours: Type 1 Diabetes (SCB-T1D) scale [22]will be used to assess the extent to which participants engaged with diabetes self-care behaviours. If an individual has completed over 50% of the items then the mean of the completed items will be used for the missing items. Otherwise the score will be coded as missing.

- System Usability Score (SUS)

Coates E, *et al. BMJ Open* 2021; 11:e040438. doi: 10.1136/bmjopen-2020-040438

The System Usability Score (SUS) [23] will be used to gather feedback on the DAFNEplus website at follow-up. As recommended by the scale authors the term ‘system’ will be replaced with ‘Glucocollector’ for the DAFNEplus group and ‘bolus calculator’ for the DAFNE (control) group. The SUS consists of 10 questions scored from 1 (strongly disagree) to 5 (strongly agree). Before calculating the total score the individual scores are transformed as follows:

– For each odd question (1,3,5,7 and 9) subtract 1
– For each even question (2,4,6,8 and 10) subtract from 5

The total score is the sum of these transformed values multiplied by 2.5, the score ranges from 1-100 with higher values indicating better usability.

- Diabetes Management Experience Questionnaire (DME-Q)

Satisfaction with diabetes treatment will be measured using the Diabetes Management Experience Questionnaire (DME-Q).

- Confidence in Diabetes Scale (CIDS)

The Confidence in Diabetes Scale (CIDS) [24] is a self-reported questionnaire with 20 items each scored from 1 (No, I am sure I cannot) to 5 (Yes, I am sure I can). The overall score is the sum of the items, minus the lowest possible score (20), divided by the score range (80) and multiplied by 100. This results in a 0-100 scale where higher scores indicate higher self-efficacy.

- Hypoglycaemia Confidence Scale (HCS)

The Hypoglycaemia Confidence Scale (HCS) [25] consists of 9 questions (8 items for participants without a partner) each rated from 1 (Not confident at all) to 4 (Very confident). The total score is calculated as the sum of the items divided by the number of items completed and ranges between 1-4 with higher scores indicating more confidence.

- Beliefs about Consequences

The Beliefs about Consequences questionnaire contains 6 items scored from 1 (not at all helpful) to 5 (extremely helpful), the items are summed to obtain the total score which ranges from 6-30.

- Self-Regulation Questionnaire (SRQ-T1D)

The SRQ-T1D questionnaire is an adaptation of the Self-Regulation Questionnaire [26], individual items are scored from 1 (strongly disagree) to 5 (strongly agree) and some items are reverse scaled (i.e. 1=5, 2=4, 3=3, 4=2, 5=1).

5.2 Analysis of Primary Outcome

The primary analysis will use the primary outcome of HbA1c at 12-months, using the primary ITT analysis population which is all consenting participants that have a baseline HbA1c > 7.5%. Descriptive statistics for baseline, 6- and 12-month HbA1c will be summarised and presented as mean, standard deviation (SD), median, min and max.

The treatment groups will be compared using a multiple linear regression model with coefficients estimated using GEE. The advantage of using GEE is that it is possible to calculate robust standard errors which are consistent even if the correlation structure is specified incorrectly [27], [28]. In this

model, an exchangeable correlation will be used to account for the clustering. In the event of any baseline differences in patient demographic characteristics, these covariates will be included in the model along with course. Adjustments for the stratification variables has not been included as they are at the centre level and therefore will be highly correlated with the course variable included in the model. The adjusted and unadjusted mean difference (MD) between DAFNE and DAFNE*plus* with associated 95% CI and p-value will be reported as well as the ICC from the model.

5.2.1 Model Checking

Given that correlation can lead to loss of information, ignoring the correlation structure can waste information and decrease standard errors when using an inappropriate analytical method. Model assumptions will be assessed graphically using the following methods:

- The linearity of the response variable will be assessed by a plot of the residuals against each explanatory variable in the model (curvilinear relationships). In cases of non-linearity, a transformation of the response variable could be performed, e.g log transformation or for particular fixed effect, a non-linear transformation of the particular fixed effect could be undertaken and included in the model,
- Constant variance will be assessed by plotting the residuals against the fitted values (errors have constant variance),
- Normality checks will be performed using a normal probability plot of the residuals (standardised) or histogram of the residuals,
- Partial residual plot will be used in identifying if quadratic or higher order terms are needed for any of the explanatory variables,
- Cook's distance can be used to indicate those observations that may be having an undue influence on the estimates. In cases of influential points, a sensitivity analyses with and without those points to assess the effects these points have on the regression coefficients will be undertaken.

5.3 Sensitivity Analyses of the Primary Outcome

Matching analyses will be undertaken on the primary outcome at 12-months using the full ITT and PP analysis sets as defined in table 4. These analyses will be completed using the primary analysis model with the only alternation the population set included within the analysis.

Additionally, a multi-level model will be completed using the primary ITT population on the primary outcome. This will contain the variables as mentioned in the original analysis but use course as a random effect to take into account the clustering in this model. These analyses will help to assess the robustness of the main trial result. The results in each case will be presented as adjusted MD of HbA1c at 12 months with associated 95% CI and p-value.

5.4 Subgroup Analyses

The following sub-group analysis will be completed on an ITT basis (Full ITT analysis population). The analysis will be the same model as the primary analysis with the addition of an interaction term between the treatment and subgroup to assess the stability of the result in different populations.

Treatment effect estimates with 95% confidence intervals will be calculated for each sub-group and a test for the overall interaction effect to reduce p-value use within each subgroup.

a) Previously attended a DAFNE course (Yes, No)
b) Use CGM or flash glucose monitoring between baseline and 12-month follow up (Yes, No)
c) Baseline HbA1c (≤7.5%, 7.5% < 8.5%, ≥8.5%)
d) Duration of diabetes (<15 years, ≥15 years)
e) Blood glucose level that symptoms of hypoglycaemia occur (do not feel symptoms, < 3mmol/l, ≥ 3mmol/l)
f) Self-reported use of the bolus advisor over the study duration (never or rarely, sometimes, often or always)
g) Age (≤34, 35-49, ≥50 years)
h) Sex (Male, Female)
i) BMI (Normal (<25), Overweight (25 < 30), Obese (≥30))
j) Socio-economic status (SES) as defined by the ONS Index of Multiple Deprivation (4 groups: above/below median in England, and above/below median in Scotland)
k) Total daily dose of insulin at baseline
l) Experience of lead course educator (Less experienced (6 courses or less within previous 3 years OR completed the DAFNE educator programme within previous year), More experienced (7+ courses within previous 3 years OR had continuous educator status for over 6 years))
m) Pregnant during the trial (Yes, No)
n) Type of basal insulin: (Human, Levemir, Lantus, Degludec, Toujeo)

5.5 Handling Missing Data

Missing observations can occur for numerous reasons (e.g. attrition) which can shrink the sample size, affects the precision of confidence intervals, reduce statistical power and biases parameter estimates [29]. Appropriately dealing with missing observations requires careful examination of data to identify the type and pattern of missingness.

In DAFNE*plus*, we anticipate that missing observations on the primary outcome (HbA1c) at 6 and 12months will occur amongst ITT participants. HbA1c results will be considered missing if the measure is outside +/- six weeks of the expected follow-up date. For the primary endpoint of HbA1c at 12 months, participant characteristics will be compared for those with and without the outcome. The aim of this is to explore any possible predictors of the missing outcome and evaluate the missing at random assumption. Multiple imputation strategies using a sequence of regression models [30] on the primary endpoint will be used where the missing values are filled ten times to generate ten complete data sets while utilising all variables that were included in the primary outcome analysis (section 5.4) as predictors. Any additional variables associated with the missing data will be included in the imputation model. This model will use a conservative approach by excluding treatment allocation.

If the data results in being missing not at random then a sensitivity analysis will be completed to assess the difference this makes on the results.

If weight is not recorded, other time points can be used as follows:

- Baseline weight missing - use 6-month weight,
- 6-month weight missing - if both baseline and 12-month weight are available, use the mean of these two. If only one of baseline or 12-month data are available, the recorded value will be used,
- All other circumstances - weight defined as missing.

5.6 Analysis of Secondary Outcomes - Biomedical

5.6.1 HbA1c

Additional secondary analyses on the primary outcome using the same model described in section 5.4 will be undertaken using HbA1c data collected at 6 months. This will be undertaken for all participants with HbA1C > 7.5% on an ITT basis. The adjusted and unadjusted mean difference (MD) between DAFNE and DAFNE*plus* with associated 95% CI and p-value will be reported as shown in table 7.11. Multiple imputations will be undertaken for missing HbA1c at 6 months using model described in 5.7.

The proportion of patients that have achieved improved glycaemic control will also be assessed. The patient is deemed to have improved glycaemic control if they achieved either: HbA1c $\leq$7.5% (58 mmol/mol) OR decrease in HbA1c ≥0.5% (≥5.5 mmol/mol). To test this, a GLM using a logit link function with treatment group, course and centre as random effects, and any baseline characteristics as used within the primary analysis model will be used (Logistic regression). Summary statistics for counts and percentages at 6 and 12 months follow up will be reported for DAFNE and DAFNE*plus* and overall. Treatment effect will be reported as both unadjusted or adjusted Odds Ratio (OR) with its associated 95% CI and p-value. This analysis will be undertaken using data collected at 6- and 12-month follow-up.

Additionally, it is important to assess the HbA1c trend over time.

5.6.2 Weight, BMI, Blood Pressure and Lipids

Summary statistics will be presented for each of these variables at baseline, and 12-months follow-up and all are treated as continuous variables. Summaries of weight and BMI at 6-months will be presented in the same way. Results will be presented by treatment group and overall and it will include the number, range and either the mean and SD or median and interquartile range depending on the distribution.

5.6.3 Severe Hypoglycaemia and DKA

The total number of episodes of hypoglycaemia will be treated as continuous and summary statistics of episodes since their last visit prior to the 12-month follow-up will be reported for DAFNE, DAFNE*plus* and overall.

To test for the difference between the two groups, the number of episodes of severe hypoglycaemia will be modelled using a negative binomial regression model with treatment group, baseline HbA1c and course (random effect). Treatment effect will be reported as Incidence Rate Ratios (IRR) with its associated 95% CI and p-value.

An additional analysis will be complete for the proportion of participants who experienced at least one episode of severe hypoglycaemia since their last visit prior to the 12 months follow-up. This will be modelled using a random effect logistic regression model. The model will include treatment group, baseline HbA1c and centre. Treatment effect will be reported and presented as Odds Ratio (OR) with its associated 95% CI and p-value.

5.6.4 Model Checking

Frequency graphs and the ratio of the variance to the mean will be used to assess the distribution of severe hypoglycaemia episodes. Failure to properly address existing over dispersion leads to serious underestimation of standard errors and misleading inference for the treatment effect. The Deviance and Likelihood Ratio Test (LRT) will be employed to assess goodness of fit of the Poisson linear regression model against two specific alternatives: a) a zero-inflated Poisson GLM (in the case of overdispersion due to excess zeros, or participants who experienced no episodes), and b) negative binomial regression for more general overdispersion. Further model diagnostics including measures of influence such as Cook's Distance will be undertaken for sensitivity analysis.

Unlike linear regression where graphical diagnostic displays can be very useful, for logistic regression models, the discreteness of binary data makes it difficult to interpret such displays. Three methods will be used for diagnostic checking of logistic regression models. Local mean deviance plots for detecting overall lack of fit, empirical probability plots to point out isolated departures from the fitted model and partial residual plots (smoothed) to identify specific causes of lack of fit.

5.7 Analysis of Secondary Outcomes - Psychological

All psychological outcomes will be analysed by the team at the University of Surrey, led by Debbie Cooke. The planned analysis will be defined by the team in a separate document and will be discussed with the statistical team to ensure consistency between methods. This will be signed off prior to data analysis.

5.8 Safety Outcomes

Adverse Events (AE) will be recorded throughout the trial and are defined as any unwanted medical occurrences which includes any episodes of diabetic ketoacidosis and any increase in frequency of severe hypoglycaemia. Serious Adverse Event (SAE) will also be recorded throughout the trial and are defined as AEs which result in hospitalisation or have a risk to life. A detailed description of AEs and SAEs can be found in the protocol.

Summary measures will be presented by treatment group as the number and percentage of participants reporting an AE/SAE as well as the total number of AE/SAEs reported and will be on an ITT basis. No formal statistical testing will be undertaken.

5.9 Statistical Software

This analysis will be carried out using any suitable packages such as R or STATA.

6 REFERENCES

6.1 Documents

DAFNE*plus* Protocol V2.0, 4th July 2018
Data Management Plan
ST001 The Statistical Analysis Plan
ST006 Undertaking a Statistical Analysis

6.2 Publications

[1] Diabetes UK, "Diabetes UK: Facts and Stats, October 2016," 2016. .
[2] The Diabetes Control and Complications Trial Research Group, "The effect of intensive treatment of diabetes on the development and progression of long-term complications in insulin-dependent diabetes mellitus," *N. Engl. J. Med.*, 1993.
[3] S. Heller, "Hypoglycaemia and diabetes," in *Textbook of Diabetes*, G. Pickup, J, WIlliams, Ed. Oxford: Blackwell Science, 2003, pp. 33.1-33.19.
[4] M. J. Campbell and S. J. Walters, *How to Design, Analyse and Report Cluster Randomised Trials in Medicine and Health Related Research*. Chichester, UK: John Wiley & Sons, Ltd, 2014.
[5] F. Li, Y. Lokhnygina, D. M. Murray, P. J. Heagerty, and E. R. DeLong, "An evaluation of constrained randomization for the design and analysis of group-randomized trials," *Stat. Methods Med. Res.*, vol. 35, no. 10, pp. 1565–1579, 2016.
[6] StataCorp, "Stata Statistical Software: Release 15," *2017*. 2017.
[7] ICH, "ICH Harmonised Tripartite Guideline. Statistical Principles for Clinical Trials. E9.," 1998. [Online]. Available: http://www.ich.org/fileadmin/Public_Web_Site/ICH_Products/Guidelines/Efficacy/E9/Step4/E9_Guideline.pdf.
[8] K. F. Schulz, D. G. Altman, D. Moher, and CONSORT Group, "CONSORT 2010 statement: updated guidelines for reporting parallel group randomised trials.," *BMJ*, vol. 340, no. mar23 1, p. c332, 2010.
[9] International Hypoglycaemia Study Group, "Glucose Concentrations of Less Than 3.0 mmol/L (54 mg/dL) Should Be Reported in Clinical Trials: A Joint Position Statement of the American Diabetes Association and the European Association for the Study of Diabetes: Table 1," *Diabetes Care*, vol. 40, no. 1, pp. 155–157, Jan. 2017.
[10] C. Bradley, C. Todd, T. Gorton, E. Symonds, A. Martin, and R. Plowright, "The development of an individualized questionnaire measure of perceived impact of diabetes on quality of life: the ADDQoL," *Qual. Life Res.*, vol. 8, no. 1/2, pp. 79–91, 1999.
[11] M. C. Rosal, B. Olendzki, G. W. Reed, O. Gumieniak, J. Scavron, and I. Ockene, "Diabetes self-management among low-income Spanish-speaking patients: A pilot study," *Ann. Behav. Med.*, vol. 29, no. 3, pp. 225–235, 2005.
[12] M. Peyrot, R. R. Rubin, T. Lauritzen, F. J. Snoek, D. R. Matthews, and S. E. Skovlund, "Psychosocial problems and barriers to improved diabetes management: results of the Cross-National Diabetes Attitudes, Wishes and Needs (DAWN) Study," *Diabet. Med.*, vol. 22, no. 10, pp. 1379–1385, Oct. 2005.
[13] W. H. Polonsky *et al.*, "Assessment of diabetes-related distress," *Diabetes Care*, 1995.
[14] D. A. W. and N. (DAWN), "Problem Area In Diabetes Questionnaire (PAID)," 1999. [Online]. Available: http://www.dawnstudy.com/content/dam/Dawnstudy/AFFILIATE/www-dawnstudy-com/Home/TOOLSANDRESOURCES/Documents/PAID_problem_areas_in_diabetes_questionnaire.pdf.

[15] C. Speight, J, Barendse, S, Bradley, "The W-BQ28: further development of the well-being Questionnaire to include diabetes-specific as well as generic subscales and new stress subscales," in *British Psychological Society, Division of Health Psychology*, 1999.
[16] J. Grabman *et al.*, "An empirically derived short form of the Hypoglycaemia Fear Survey II," *Diabet. Med.*, vol. 34, no. 4, pp. 500–504, Apr. 2017.
[17] M. Herdman *et al.*, "Development and preliminary testing of the new five-level version of EQ-5D (EQ-5D-5L)," *Qual. Life Res.*, vol. 20, no. 10, pp. 1727–1736, Dec. 2011.
[18] N. J. Devlin, K. K. Shah, Y. Feng, B. Mulhern, and B. van Hout, "Valuing health-related quality of life: An EQ-5D-5L value set for England," *Health Econ.*, Aug. 2017.
[19] J. Carlton *et al.*, "Developing a questionnaire to determine the impact of self-management in diabetes: giving people with diabetes a voice," *Health Qual. Life Outcomes*, vol. 15, no. 1, p. 146, Dec. 2017.
[20] A. E. Gold, K. M. MacLeod, and B. M. Frier, "Frequency of severe hypoglycemia in patients with type I diabetes with impaired awareness of hypoglycemia.," *Diabetes Care*, vol. 17, no. 7, pp. 697–703, Jul. 1994.
[21] M. E. Hilliard, E. Iturralde, J. Weissberg-Benchell, and K. K. Hood, "The Diabetes Strengths and Resilience Measure for Adolescents With Type 1 Diabetes (DSTAR-Teen): Validation of a New, Brief Self-Report Measure," *J. Pediatr. Psychol.*, vol. 42, no. 9, pp. 995–1005, Oct. 2017.
[22] C. D. Mansell P, Grant L, Bond R, Pouwer F, Page N, Davies A, Stenner K, Elliott J, Hopkins D, Jones A, Vallis M, Speight J, Lawton J, Clark M, Heller S, "Assessment of Self-Care Behaviours focused on Glycemic Control in Type 1 Diabetes: Development and Validation of the SCB-T1D Questionnaire." .
[23] J. Brooke, "SUS-A quick and dirty usability scale," *Usability Eval. Ind.*, vol. 189, no. 194, pp. 4–7, 1996.
[24] N. C. W. Van Der Ven *et al.*, "The confidence in diabetes self-care scale: Psychometric properties of a new measure of diabetes-specific self-efficacy in Dutch and U.S. patients with type 1 diabetes," *Diabetes Care*, vol. 26, no. 3, pp. 713–718, Mar. 2003.
[25] W. H. Polonsky, L. Fisher, D. Hessler, and S. V. Edelman, "Investigating Hypoglycemic Confidence in Type 1 and Type 2 Diabetes," *Diabetes Technol. Ther.*, vol. 19, no. 2, pp. 131–136, Feb. 2017.
[26] J. M. Brown, W. R. Miller, and L. A. Lawendowski, "The Self-Regulation Questionnaire.," in *Vandecreek, L. and Jackson, T.L., Eds., Innovations in Clinical Practice: A Sourcebook*, Vol. 17., Professional Resource Press/Professional Resource Exchange, Sarasota, FL, 1999, pp. 281–292.
[27] K.-Y. Liang and S. L. Zeger, "Longitudinal Data Analysis Using Generalized Linear Models," *Biometrika*, 1986.
[28] E. Demidenko and T. A. Stukel, "Influence analysis for linear mixed-effects models," *Stat. Med.*, 2005.
[29] P. D. Allison, "Missing Data," *Quant. Appl. Soc. Sci.*, 2001.
[30] T. E. Raghunathan, J. M. Lepkowski, J. Van Hoewyk, and P. Solenberger, "A multivariate technique for multiply imputing missing values using a sequence of regression models," *Surv. Methodol.*, 2001.
