# Supplementary material for: Protocol for a cluster randomised controlled trial of the DAFNEplus (Dose Adjustment For Normal Eating) intervention compared with 5x1 DAFNE: a lifelong approach to promote effective self-management in adults with type 1 diabetes
Source: BMJ Open. 2021 Jan 18;11(1):e040438. doi: 10.1136/bmjopen-2020-040438 (PMC7813353; doi:10.1136/bmjopen-2020-040438)
Supplement: Author's manuscript [file bmjopen-2020-040438.draft_revisions.pdf]

BMJ Open is committed to open peer review. As part of this commitment we make the peer review history of every article we publish publicly available.

When an article is published we post the peer reviewers' comments and the authors' responses online. We also post the versions of the paper that were used during peer review. These are the versions that the peer review comments apply to.

The versions of the paper that follow are the versions that were submitted during the peer review process. They are not the versions of record or the final published versions. They should not be cited or distributed as the published version of this manuscript.

BMJ Open is an open access journal and the full, final, typeset and author-corrected version of record of the manuscript is available on our site with no access controls, subscription charges or pay-per-view fees (http://bmjopen.bmj.com).

If you have any questions on BMJ Open's open peer review process please email info.bmjopen@bmj.com

BMJ Open

A cluster randomised controlled trial of the DAFNEplus (Dose Adjustment for Normal Eating) intervention compared with 5x1 DAFNE: A lifelong approach to promote effective self-management in adults with type 1 diabetes

Journal:	*BMJ Open*
Manuscript ID	bmjopen-2020-040438
Article Type:	Protocol
Date Submitted by the Author:	15-May-2020
Complete List of Authors:	Coates, Elizabeth; University of Sheffield, ScHARR<br>Amiel, Stephanie; Kings College London School of Medicine, Diabetes, Endocrinology and Internal Medicine<br>Baird, Wendy; University of Sheffield, ScHARR<br>Benaissa, Mohammed; University of Sheffield, Department of Electronic and Electrical Engineering<br>Brennan, Alan; University of Sheffield, School of Health & Related Research<br>Campbell, Michael; University of Sheffield, Health Services Research ScHARR<br>Chadwick, Paul; UCL, Epidemiology and Public Health<br>Chater, Tim; The University of Sheffield, School of Health and Related Research<br>Choudhary, Pratik; King's College London<br>Cooke, Debbie; University of Surrey,<br>Cooper, Cindy; University of Sheffield, School of Health and Related Research<br>Cross, Elizabeth; University of Sheffield, ScHARR<br>De Zoysa, Nicole; King's College Hospital<br>Eissa, Mohammad; University of Sheffield, Department of Electronic and Electrical Engineering<br>Elliott, Jackie; University of Sheffield, Academic Unit of Diabetes, Endocrinology and Metabolism<br>Gianfrancesco, Carla; Sheffield Teaching Hospitals NHS Foundation Trust, Diabetes Centre<br>Good, Tim; University of Sheffield, Department of Electronic and Electrical Engineering<br>Hopkins, David; King's College London, General and Emergency Medicine<br>Hui, Zheng; University of Sheffield, Department of Electronic and Electrical Engineering<br>Lawton, Julia; University of Edinburgh, Centre for Population Health Sciences<br>Lorencatto, Fabiana; UCL<br>Michie, Susan; University College London, Centre for Outcomes Research and Effectivenes<br>Pollard, Daniel ; University of Sheffield, School of Health and Related Research<br>Rankin, David; University of Edinburgh, Centre for Population Health

Sciences
Schutter, Jose; University of Sheffield, ScHARR
Scott, Elaine; University of Sheffield, ScHARR
Speight, Jane; The Australian Centre for Behavioural Research in Diabetes,
Stanton-Fay, Stephanie; UCL, Epidemiology and Public Health
Taylor, Carolin; Sheffield Teaching Hospitals NHS Foundation Trust, Diabetes Centre
Thompson, Gillian; Northumbria Healthcare NHS Foundation Trust
Totton, Nikki; University of Sheffield, School of Health and Related Research
Yardley, Lucy; University of Southampton, Academic Unit of Psychology
Zaitcev, Aleksandr; University of Sheffield, Department of Electronic and Electrical Engineering
Heller, Simon; Unversity of Sheffield, Academic Unit of Diabetes, Endo and Metab

Keywords: DIABETES & ENDOCRINOLOGY, EDUCATION & TRAINING (see Medical Education & Training), HEALTH ECONOMICS, QUALITATIVE RESEARCH

SCHOLARONE™
Manuscripts

I, the Submitting Author has the right to grant and does grant on behalf of all authors of the Work (as defined in the below author licence), an exclusive licence and/or a non-exclusive licence for contributions from authors who are: i) UK Crown employees; ii) where BMJ has agreed a CC-BY licence shall apply, and/or iii) in accordance with the terms applicable for US Federal Government officers or employees acting as part of their official duties; on a worldwide, perpetual, irrevocable, royalty-free basis to BMJ Publishing Group Ltd ("BMJ") its licensees and where the relevant Journal is co-owned by BMJ to the co-owners of the Journal, to publish the Work in this journal and any other BMJ products and to exploit all rights, as set out in our licence.

The Submitting Author accepts and understands that any supply made under these terms is made by BMJ to the Submitting Author unless you are acting as an employee on behalf of your employer or a postgraduate student of an affiliated institution which is paying any applicable article publishing charge ("APC") for Open Access articles. Where the Submitting Author wishes to make the Work available on an Open Access basis (and intends to pay the relevant APC), the terms of reuse of such Open Access shall be governed by a Creative Commons licence – details of these licences and which Creative Commons licence will apply to this Work are set out in our licence referred to above.

Other than as permitted in any relevant BMJ Author's Self Archiving Policies, I confirm this Work has not been accepted for publication elsewhere, is not being considered for publication elsewhere and does not duplicate material already published. I confirm all authors consent to publication of this Work and authorise the granting of this licence.

For peer review only

Title: A cluster randomised controlled trial of the DAFNE*plus* (Dose Adjustment for Normal Eating) intervention compared with 5x1 DAFNE: A lifelong approach to promote effective self-management in adults with type 1 diabetes

Authors: The DAFNE*plus* group (to be listed as)

Elizabeth Coates	University of Sheffield	e.coates@sheffield.ac.uk
Stephanie Amiel	King's College Hospital	stephanie.amiel@kcl.ac.uk
Wendy Baird	University of Sheffield	w.o.baird@sheffield.ac.uk
Mohammed Benaissa	University of Sheffield	m.benaissa@sheffield.ac.uk
Alan Brennan	University of Sheffield	a.brennan@sheffield.ac.uk
Mike Campbell	University of Sheffield	m.j.campbell@sheffield.ac.uk
Paul Chadwick	University College London	p.chadwick@ucl.ac.uk
Tim Chater	University of Sheffield	t.chater@sheffield.ac.uk
Pratik Choudhary	King's College Hospital	pratik.choudhary@kcl.ac.uk
Debbie Cooke	University of Surrey	d.cooke@surrey.ac.uk
Cindy Cooper	University of Sheffield	c.l.cooper@sheffield.ac.uk
Elizabeth Cross	University of Sheffield	e.a.cross@sheffield.ac.uk
Nicole De Zoysa	King's College Hospital	ndezoysa@nhs.net
Mohammad Eissa	University of Sheffield	m.eissa@sheffield.ac.uk
Jackie Elliott	Sheffield Teaching Hospitals NHS Foundation Trust	j.elliott@sheffield.ac.uk
Carla Gianfrancesco	Sheffield Teaching Hospitals NHS Foundation Trust	carla.gianfrancesco@nhs.net
Tim Good	University of Sheffield	tim.good@sheffield.ac.uk
David Hopkins	King's College Hospital	dhopkins3@nhs.net
Zheng Hui	University of Sheffield	zhui2@sheffield.ac.uk
Julia Lawton	University of Edinburgh	j.lawton@ed.ac.uk
Fabi Lorencatto	University College London	f.lorencatto@ucl.ac.uk
Susan Michie	University College London	s.michie@ucl.ac.uk
Daniel Pollard	University of Sheffield	d.j.pollard@sheffield.ac.uk
David Rankin	University of Edinburgh	a.d.rankin@ed.ac.uk
Jose Schutter	University of Sheffield	j.schutter@sheffield.ac.uk
Elaine Scott	University of Sheffield	elaine.scott@sheffield.ac.uk
Jane Speight	The Australian Centre for Behavioural Research in Diabetes: Deakin University & Diabetes Victoria	jspeight@acbrd.org.au
Stephanie Stanton-Fay	University College London	stephanie.stanton-fay@ucl.ac.uk
Carolin Taylor	Sheffield Teaching Hospitals NHS Foundation Trust	carolin.taylor@nhs.net
Gillian Thompson	Northumbria Healthcare NHS	gillian.thompson@northumbria-healthcare.nhs.uk
Nikki Totton	University of Sheffield	n.v.totton@sheffield.ac.uk
Lucy Yardley	University of Southampton	l.yardley@soton.ac.uk

Alexsandr Zaitcev	University of Sheffield	a.zaitcev@sheffield.ac.uk
Simon Heller*	Sheffield Teaching Hospitals NHS Foundation Trust	s.heller@sheffield.ac.uk

* Corresponding author:
Professor Simon Heller, Professor of Clinical Diabetes, Department of Human Metabolism, Sheffield Teaching Hospitals NHS Foundation Trust, Beech Hill Road, Sheffield S10 2RX, Tel: 0114 215 9009, s.heller@sheffield.ac.uk

Version 6.0, 09.09.2019

Word count: 4768

For peer review only

Abstract

Introduction: The successful treatment of type 1 diabetes (T1D) requires those affected to employ insulin therapy to maintain their blood glucose levels as close to normal to avoid complications in the long-term. The Dose Adjustment for Normal Eating (DAFNE) intervention is a group education course designed to help adults with T1D develop and sustain the complex self-management skills needed to adjust insulin in everyday life. It leads to improved glucose levels in the short-term (manifest by falls in HbA1c), reduced rates of hypoglycaemia and sustained improvements in quality of life but overall glucose levels remain well above national targets. The DAFNE*plus* intervention is a development of DAFNE designed to incorporate behaviour change techniques, technology and longer-term structured support from healthcare professionals.

Methods and analysis: A pragmatic cluster randomised controlled trial in adults with T1D, delivered in diabetes centres in NHS secondary care hospitals in the United Kingdom. Centres will be randomised on a 1:1 basis to standard DAFNE or DAFNE*plus.*

Primary clinical outcome is the change in HbA1c (glycated haemoglobin) and the primary endpoint is HbA1c at 12 months, in those entering the trial with HbA1c >7.5% (58 mmol/mol), and HbA1c at 6 months is the secondary endpoint. Sample size is 662 participants (approximately 47 per centre); 92% power to detect a 0.5% difference in the primary outcome of HbA1c between treatment groups. The trial also measures rates of hypoglycaemia, psychological outcomes, an economic evaluation and process evaluation.

Ethics and dissemination: Ethics approval was granted by South West – Exeter Research Ethics Committee (REC ref: 18/SW/0100) on 14th May 2018. The results of the trial will be published in a National Institute for Health Research monograph and relevant high-impact journals.

Trial registration: ISRCTN42908016 – registered on 17th May 2018

For peer review only

Article summary

Strengths and limitations of this study

- Comparison of group therapy against another group therapy will standardise the treatment comparison.
- Cluster randomisation to avoid contamination of the intervention material.
- Number of sites in both England and Scotland representing a wide range of NHS Trusts.
- Use of a covariate constrained methodology to randomise means that sites are matched which can create issues if sites drop out.
- Blinding not possible in trial due to the intervention and design.

Keywords

Diabetes mellitus, Type 1; Self management; Patient education; Randomised controlled trial;

Funding:
This work is funded by the National Institute for Health Research through their Programme Grants for Applied Research Programme (Ref: RP-PG-0514-20013) and NHS England provide funding for Excess Treatment Costs.

Sponsor: Sheffield Teaching Hospitals NHS Foundation Trust
Contact Name Dr Erica Wallis
Clinical Research Office
Sheffield Teaching Hospitals NHS Foundation Trust
Glossop Road
Sheffield S10 2JF
Phone: 0114 2265931
Email: erica.wallis1@nhs.net

Role of study sponsor and funder
Neither the funder nor the sponsor has had any role in study design, data collection or analysis, decision to publish, or preparation of manuscripts.

For peer review only

Chief Investigator: Professor Simon Heller, Professor of Clinical Diabetes, Sheffield Teaching Hospitals NHS Foundation Trust

Trial Co-ordinating Centre: Sheffield CTRU, University of Sheffield
Mrs Elaine Scott, Trial Manager
Mrs Liz Cross, Senior Trial Manager
Miss Jose Schutter, Research Assistant
Mr Tim Chater, Data Manager
Miss Becky Brown, Trials Support Officer
Professor Cindy Cooper, Trials Oversight

Trial Management Group:
Professor Stephanie Amiel, King's College London
Professor Wendy Baird, University of Sheffield
Dr Mohammed Benaissa, University of Sheffield
Professor Alan Brennan, University of Sheffield
Miss Becky Brown, University of Sheffield
Dr Paul Chadwick, University College London
Mr Tim Chater, University of Sheffield
Dr Pratik Choudhary, King's College London
Dr Debbie Cooke, University of Surrey
Professor Cindy Cooper, University of Sheffield
Dr Mohammad Eissa, University of Sheffield
Dr Tim Good, University of Sheffield
Ms Carla Gianfrancesco, Sheffield Teaching Hospitals NHS Foundation Trust
Dr Jackie Elliott, University of Sheffield
Dr David Hopkins, King's College Hospital NHS Foundation Trust
Professor Julia Lawton, University of Edinburgh
Dr Fabiana Lorencatto, University College London
Professor Susan Michie, University College London
Mr Dan Pollard, University of Sheffield
Dr David Rankin, University of Edinburgh
Miss Jose Schutter, University of Sheffield
Mrs Elaine Scott, University of Sheffield
Professor Jane Speight, Deakin University, Australia
Dr Stephanie Stanton-Fay, University College London
Mrs Carolin Taylor, Sheffield Teaching Hospitals NHS Foundation Trust
Mrs Gill Thompson, Northumbria Healthcare NHS Foundation Trust
Ms Nikki Totton, University of Sheffield
Mr Chris Turtle, University of Sheffield
Dr Nicole de Zoysa, King's College Hospital NHS Foundation Trust

Trial Steering Committee membership:
Chair: Professor Simon Griffin, University of Cambridge;
Independent Statistician: Professor Catherine Hewitt, University of York;
Independent Clinical Expert: Professor James Shaw, University of Newcastle
Patient Representative: Mr Arthur Durrant, Ms Lynne Dawson
Sponsor's Representative: Dr Erica Wallis, Sheffield Teaching Hospitals NHS Foundation Trust
Funder's Representative: Mr Ramnath Elaswarapu, National Institute for Health Research

For peer review only

Introduction

Background and Rationale

Type 1 diabetes (T1D) is characterised by absolute insulin deficiency, requiring insulin to be injected subcutaneously several times a day. Successful management requires those affected (>300,000 adults in the UK)[1] to keep their blood glucose levels sufficiently close to recommended targets to avoid long-term complications including blindness, renal failure, amputations and premature death[2]. In addition, exogenous insulin therapy can prevent high blood glucose and acute, life threatening emergencies such as diabetic ketoacidosis, as well as being a tool to prevent long-term complications.

Achieving the blood glucose control to help prevent complications depends upon an individual's ability to self-manage their condition, calculating precise insulin doses based on accurate estimations of food intake before every meal, frequent blood glucose measurements, and account for fluctuations in physical activity, illness and hormones. If people with T1D are unable or unwilling to calculate and administer their insulin doses correctly, their blood glucose either runs high, increasing the risks of complications, or else falls too low leading to hypoglycaemia. Hypoglycaemia, if severe, can result in acute cognitive impairment, confusion, collapse and injury, coma or even death[3]. Thus, people with T1D must acquire complex self-management knowledge and skills, and have the motivation and ability to apply them effectively every day. The responsibility of diabetes healthcare professionals (HCPs) is to ensure that all people with T1D have the opportunity to acquire these skills and are supported in applying them successfully in everyday life.

'Dose Adjustment for Normal Eating' (DAFNE) is a structured education programme run within the National Health Service (NHS), designed to enable adults with T1D to learn or enhance their self-management skills in flexible intensive insulin therapy to improve both glucose control and quality of life. It is a five-day training course, delivered in small groups. DAFNE has been delivered to over 51,000 adults in the UK[4]. The publication of the UK DAFNE randomised controlled trial (RCT) in 2002[5] established the ability of structured education courses to enable people with diabetes to acquire the knowledge and skills to live successfully with this lifelong condition. The subsequent rollout of DAFNE across the UK has enabled many individuals to meet these demands and achieve their goals, but over half of DAFNE graduates still struggle to manage glucose levels consistently. After attending a DAFNE course, people have better quality of life, better control of blood glucose levels and are admitted to hospital less often for diabetes emergencies[6]. Many DAFNE graduates find the course helpful; quality of life improves and rates of severe hypoglycaemia fall. However although HbA1c (glycated haemoglobin) falls and in one trial, this improvement was sustained for 2 years, average HbA1c, the intermediate measure of glucose control that best predicts risk of diabetes complications, remains well above recommended UK targets[7,8]. Many, find it difficult to implement and sustain the skills needed to maintain blood glucose levels and often struggle to obtain suitable support from HCPs[6,9–15].

For peer review only

The DAFNE*plus* intervention has been developed through modifying the existing DAFNE programme by incorporating techniques for initiating and sustaining behaviour change, and supplementing this with structured follow-up support and enhanced information technology. The aim of this trial is to investigate whether the DAFNE*plus* programme will produce improved and sustainable diabetes self-management behaviour and better glucose outcomes than currently achieved with standard DAFNE, without compromising quality of life in the longer term.

Aims and objectives

The primary aim of this study is to conduct a cluster RCT comparing the new DAFNE*plus* intervention to the existing DAFNE programme to answer the following question:

In adults with T1D, will modifying the existing DAFNE programme and developing structured professional input, using learning from our recent research, behaviour change theory and new forms of technological support, produce improved and sustained diabetes self-management behaviours, leading to better glucose control than currently achieved, using the existing DAFNE intervention, without compromising quality of life?

The primary objective is to assess the effects of the intervention on glycaemic control, as measured by HbA1c at 12 months.

The secondary objectives of this trial are:

1. To compare the effects of the intervention (DAFNE*plus*) to standard DAFNE on diabetes-specific quality of life.
2. To compare the medium term effect of the intervention (DAFNE*plus*) to standard DANFE on glycaemic control as measured by HbA1c using data at 6 months.
3. To compare the effects of the intervention (DAFNE*plus*) to standard DAFNE on other biomedical outcomes.
4. To compare the effects of the intervention (DAFNE*plus*) to standard DAFNE on psychological outcomes.
5. To undertake a mixed methods process evaluation to aid understanding of the RCT findings, and to inform decision making about the implementation of DAFNE*plus* in clinical care post-trial.
6. To assess fidelity of delivery of the DAFNE*plus* intervention.
7. To undertake a health economic analysis to determine the cost-effectiveness of DAFNE*plus* versus standard DAFNE.

Methods and analysis

Trial Design

The study will use a pragmatic cluster randomised controlled trial design. This is required since ‘contamination’ of the control arm may occur if DAFNE healthcare professionals, trained in the new programme were to deliver standard DAFNE. Hence the randomisation of DAFNE centres rather than

individuals[16]. Figure 1 shows the flow of participants through the trial (**s**ee supplementary material 1 for WHO Trial Registration Data Set).

[Insert Figure 1 here]

Study setting

The trial will be delivered in adult diabetes centres in secondary care NHS hospitals in the UK. The eligibility criteria for study centres are:

1. Diabetes centre delivering DAFNE to adults with T1D;
2. At least three DAFNE educators trained in delivering the 5-week model of DAFNE;
3. Delivery of sufficient DAFNE courses per year to recruit study sample.

Adults with T1D eligible for or referred to DAFNE courses at participating centres as part of usual care will be eligible to be invited to participate in the RCT, and standard criteria for referral to DAFNE will be utilised.

Eligibility criteria

Inclusion criteria:

1. Adults (≥18 years);
2. Diagnosis of type 1 diabetes for at least 6 months, or post-honeymoon[1];
3. Prepared to undertake multiple daily injection (MDI) therapy;
4. Prepared to undertake frequent self-monitoring of blood glucose;
5. Confirms availability to attend all sessions as part of the intervention;
6. Investigator has confidence that the patient is capable of adhering to all the trial protocol requirements.

Exclusion criteria:

1. Current use of continuous subcutaneous insulin infusion (CSII) pump therapy;
2. HbA1c > 12% (108mmol/mol) (Investigators can use their judgement, informed by standard DAFNE guidelines and in agreement with the trial team, to include participants with HbA1c >12%);
3. Serious diabetic complications (e.g. blindness, renal dialysis). (Investigators can use their clinical judgement, informed by standard DAFNE guidelines and in agreement with the trial team);
4. Other serious co-morbidities e.g. psychosis, diagnosed eating disorder (Investigators can use their clinical judgement, informed by standard DAFNE guidelines and in agreement with the trial team);
5. Previous participation in standard DAFNE course less than 5 years before proposed study enrolment date;
6. Unable to speak/hear/understand/read or write in English;

[1] The honeymoon period refers to the time when, post-diagnosis, people start taking insulin injections, and their insulin producing cells sometimes recover temporarily (generally around 3 -12 months. The dose of insulin needed might reduce during this period, and some people might even need to stop using insulin for a while, but eventually it will be needed again. The criteria for referral to DAFNE at least 6 months after diagnosis is to allow for the honeymoon period to have passed before attendance at the course.

For peer review only

7. Unable to give written informed consent.

Recruitment

Patient participants will be identified from current caseloads of adults with T1D from each participating centre. They will be sent an invitation letter and information sheet before the course. A member of the clinical team in participating centres will then telephone potential participants to discuss whether or not they are interested in principle in taking part. If interested, they will be asked to consent to participate at their baseline visit. In both trial arms, if they do not want to take part in the research they will be offered attendance at a standard DAFNE course that is not part of this trial, if that is their wish. Reasons for non-participation in the trial will be recorded.

In order to maximise recruitment to the courses, a reserve list of eligible patients will be held at participating centres. Eligible patients may also be invited to take part by their HCP during routine face-to-face appointments, or via telephone. Trial information meetings may also be held during the recruitment period at various locations in centres.

Written informed consent will be obtained from all participants. Members of the local study teams will be responsible for taking informed consent from potentially eligible study participants at the DAFNE centres. The process for obtaining participant informed consent will be in accordance with the REC guidance, and Good Clinical Practice (GCP) and any other regulatory requirements that might be introduced.

Written informed consent to contribute to the process evaluation will also be taken from HCPs in participating sites by the central study team.

Interventions

Standard DAFNE (control arm)

DAFNE is a skill-based structured education programme for adults with T1D delivered in the NHS. Two evidence-based models of delivering standard DAFNE are in operation, whereby the five sessions of the course are delivered weekly or daily, as described elsewhere [17]. Each course is delivered to seven participants on average (minimum of four and maximum of eight).

The aim of the course is to train adults with T1D in the skills to manage their condition effectively. It covers numerous topics in a progressive modular based structure. In addition to the five days of the course, participants are asked to attend a baseline appointment before the DAFNE course, and they are also typically invited to attend an optional group follow-up session 6-8 weeks after the course. They may also attend routine appointments every 6-12 months and seek ad-hoc support from local diabetes clinicians post-course.

For the purposes of this study, the control arm will be the 5-week model of standard DAFNE to match the frequency of sessions offered in DAFNE*plus*. All

For peer review only

participants in the control arm will be given access to a stand-alone bolus calculator to assist them with calculating insulin doses. There will be no structured follow-up appointments beyond those provided in usual care. To qualify as adherent for statistical purposes, participants need to have attended the equivalent of four days of the course including days one and two which are mandatory; it will be acceptable to include half days in the total.

DAFNE*plus* (intervention arm)

DAFNE*plus* will be delivered by trained DAFNE educators in the NHS[2]. These will be HCPs including diabetes specialist nurses, dietitians and physicians, all of whom will be using DAFNE principles as an integral part of the management of T1D in adults. DAFNE*plus* is a complex intervention, defined by the Medical Research Council[18] as having 'several interacting components', described in summary below.

The development of the content and structure of the DAFNE*plus* programme was informed by the Behaviour Change Wheel (BCW) framework[19]. The intervention's proposed functions are served by behaviour change techniques (BCTs), specified in the hierarchical Behaviour Change Technique Taxonomy v1 (BCTTv1)[20], deemed its 'active ingredients'[21]. The development of the DAFNE*plus* programme (described in [22]) was informed by expert consensus, integrating data on participant- and clinician-generated barriers and facilitators to sustaining DAFNE with the findings from a synthesis of qualitative evidence about post-DAFNE challenges[22]. Prior to this RCT, the DAFNE*plus* programme was piloted in three NHS Diabetes Centres.

The DAFNE*plus* programme comprises three components:

a) DAFNE*plus* course

The group-based course component of the DAFNE*plus* programme is delivered one day per week, over five consecutive weeks, and is based on a revision of the standard DAFNE five-week curriculum, with a view to strengthening and sustaining self-management behaviours over a longer term to enable them to achieve blood glucose levels closer to target. Participants will attend an individual pre-course appointment approximately two weeks before the course which serves as their introduction to the programme, during which they are given access to and trained in using the DAFNE*plus* technology (see below), as well as a bolus calculator.

New sessions included in the DAFNE*plus* course include technology assisted individual review, emotional aspects of living with diabetes and its management, harnessing social support, and behaviour change – including additional support for action planning and relapse prevention to help participants achieve their self-management goals. The curriculum was revised to be consistent with modern approaches to the recommended language used

[2] In DAFNE*plus*, those delivering the intervention are referred to as 'facilitators', as opposed to 'educators' in standard DAFNE.

in diabetes care[23]. Requirements to qualify as adherent for statistical purposes are defined above.

b) Structured follow-up support

The model of structured follow-up support builds upon the clinical and behavioural skills introduced during the course to enable participants to maximise the efficacy of key DAFNE*plus* principles to improve self-management and achieve/sustain glycaemic targets. As part of the trial, up to five one-to-one consultations (face-to-face, telephone or in some centres, web-based video calling) with a DAFNE facilitator will be offered, delivered at progressively wider spaced intervals during the 12 months after the course. Appointments are supported by paperwork to ‘activate’ both the participant and the facilitator prior to meeting.

The purpose of these individual sessions is to review participants’ progress with managing their diabetes, including progress with their action plans, review blood glucose data on the DAFNE*plus* website, revise course material, address any additional clinical needs, and signpost participants to any relevant sources of support. In addition, ad-hoc support by telephone, email or web-based video calling will be available, as necessary. To qualify as adherent for statistical purposes, participants will need to have attended a minimum of three follow-up sessions.

c) Digital technology

The DAFNE*plus* programme incorporates two forms of digital technology via the DAFNE*plus* website and box. Participants will be given access and training at the pre-course appointment, so that they can use the technology before and throughout the 12 month programme. The DAFNE*plus* box (*Withcare+*) transmits, stores and displays blood glucose (and other) data on a secure-server via the DAFNE*plus* website in formats to help people with T1D and their HCPs recognise and interpret blood glucose patterns. The website also includes an e-learning section to help maintain knowledge of the DAFNE*plus* approach.

Training and supervision

A clinical psychologist who specialises in diabetes and is experienced in training diabetes professionals in behaviour change skills will lead the development and delivery of DAFNE*plus* facilitator training and supervision. The training programme is delivered over a maximum of five days and will build on the existing skill-set of DAFNE facilitators but also draw on additional behavioural science to deliver the revised curriculum.

Throughout the trial, facilitators in each centre will be offered supervision by the clinical psychologist and a DAFNE*plus* facilitator. Supervision will comprise of weekly teleconferences before and during the first DAFNE*plus* course, weekly email supervision (for subsequent courses) and ad-hoc remote support to allow issues that arise to be addressed in a timely manner during the trial.

For peer review only

Criteria for withdrawal from or discontinuation of trial treatment

The decision regarding participation in the study is entirely voluntary, and consent regarding study participation may be withdrawn at any time without affecting the quality or quantity of future medical care. No study-specific interventions will be undertaken before informed consent has been obtained.

A participant will be classed as complete if they have continued in the study until the last protocol defined intervention (final 12 month outcome assessment), although there may be missing data for individual participants.

Random allocation

Upon recruitment of centres and following ethical approval, the participating centres will be randomised on a 1:1 basis to control or the intervention arm of the trial by the trial statistician. As there are numerous stratification variables that have been identified as clinically important and the small number of randomising centres, a covariate constrained methodology[24] will be employed. The centres will be matched on the number of patients within the centre, number of educators within the centre and number of previous DAFNE courses delivered (as a marker of centre experience) to balance centres between the two arms of the trial.

Blinding

Due to the nature of the intervention, it is not possible for members of the study team working directly with participants or the intervention to be blinded. Additionally, the blinding of the statistician is problematic due to the cluster level randomisation. Statisticians are usually involved within TMG discussions and have access to status reports where the potential for unintentional unblinding is a high possibility. It is considered important for the statistician to be included in these aspects of the trial management and so after discussion with senior statisticians at the Clinical Trials Research Unit (CTRU) and the independent statistician on the Trial Steering Committee, it has been deemed acceptable that the statisticians are not blind within this study.

Outcomes

Table 1 shows a breakdown of all outcome measures.

Biomedical outcomes

The primary biomedical outcome is an integrated measure of glucose levels over the previous 4-6 weeks, defined by HbA1c (using a centralised assay to ensure standardisation). The primary endpoint is HbA1c at 12 months, in those entering the trial with HbA1c >7.5% (58 mmol/mol), and HbA1c at 6 months is the secondary endpoint.

For peer review only

Our primary aim is to compare HbA1c between the two arms and we have therefore confined our primary analysis to those with raised A1c values at baseline. We therefore excluded those with an HbA1c below 7.5% (58 mmol/mol) when calculating the primary endpoint as these people have less need to reduce their HbA1c.

However, we have included participants with lower A1c values to ensure we can calculate important secondary outcomes part rates of hypoglycaemia, and other biomedical and psychological outcomes. We have estimated the expected proportion of participants with A1c values above 7.5% at 75% of those currently undertaking DAFNE courses based on a national research database.

Other secondary outcomes are the number of participants achieving either an HbA1c <7.5% (58 mmol/mol) or a decrease in HbA1c of ≥0.5% (≥5.5 mmol/mol) which will be calculated at both 6 and 12 months post course. These cut-off points are recognised throughout the diabetes research community as being clinically relevant[25]. We will also collect and analyse 24-month outcome data (HbA1c and severe hypoglycaemic episodes) and analyse after the main study has closed and been reported based on locally available clinical data which is routinely collected annually in clinical centres.

Other secondary biomedical outcomes will include: Severe hypoglycaemia, as defined by the American Diabetes Association[26], denotes severe cognitive impairment requiring external assistance for recovery, both rates and proportion of those affected; Diabetic ketoacidosis, both rates and proportion of those affected; weight; Body Mass Index; Blood Pressure; Lipids; Albumin/creatinine ratio.

Psychological outcomes and process evaluation

Quantitative outcomes

Psychological outcomes and process measures will be collected via self-completed postal or online questionnaires at baseline, course completion, 3, 6, 9 and 12 months (see Table 1).

The primary psychological outcome is the impact of diabetes on quality of life assessed at 12 months using a 15-domain version of the Audit-Dependent Diabetes Quality of Life Questionnaire (ADDQoL-15)[27].

Additional psychological constructs are assessed with validated questionnaires and study-specific individual items, based upon: existing knowledge about their association with the trial's primary biomedical outcome (HbA1c) and primary psychological outcome (diabetes-specific quality of life), including the findings of the YourSAY survey[28]; previous work with the DAFNE intervention, and the theoretical framework underpinning the DAFNE*plus* intervention development and possible treatment mechanisms[19,29,30].

Qualitative outcomes

Interviews will be undertaken with a subset of participants randomized to the intervention at baseline, course completion, 3 months and 12 months (Figure 1) to explore how key elements of the intervention influence and inform changes

For peer review only

to, and maintenance of, key self-management behaviours over time. Facilitators will be interviewed from across the intervention sites to explore their experiences of intervention delivery and their views about the training, resourcing and support staff would need to deliver DAFNE*plus* in routine care.

Fidelity assessment

We will explore fidelity of delivery using two methods to assess the extent to which the intervention content specified in the DAFNE/DAFNE*plus* manuals is delivered as intended: self-report checklists completed by educators/facilitators, and objectively analysed delivery from session audio recordings. Fidelity of delivery will be assessed in standard DAFNE as well as DAFNE*plus* in order to assess any loss of treatment differentiation and potential contamination between the two arms.

1) Self-report checklists: Facilitators will complete checklists after each session. Each checklist lists the components intended to be delivered in each session (according to the manual). These components correspond to different BCTs. Each component will be rated as fully, partially or not delivered, with space for additional comments. The proportion of intended components rated as partially/fully delivered by educators/facilitators will be calculated, with <50% of intended content delivered classified as low fidelity; 51-79% as moderate fidelity, and 80-100%' as high fidelity[31].

2) Objectively analysed delivery: A subsample of group course sessions in both arms will be audio-recorded and transcribed verbatim. Transcripts will be coded into component BCTs using an established taxonomy[20]. BCTs identified in each session transcript will be compared to corresponding section of the intervention manual that specifies which BCTs are intended to be delivered in that session. Fidelity will be calculated in terms of the percentage of manual-specified BCTs delivered as intended. Additional BCTs delivered that are not specified in the curricula will also be noted.

Detailed plans for the process evaluation are in supplementary material 2.

Health economic outcomes

Table 1 details the health economic data collected in the trial. In addition, data collected from the DAFNE*plus* website will be used to cost the intervention. The analysis population for the health economic analyses will include all trial participants, as it is important that the analysis of health economic data includes all participants who would be eligible to receive DAFNE*plus* (if it were to be implemented). In line with the statistical analysis, we will conduct subgroup analyses in participants with an HbA1c ≤ 7.5% and > 7.5% (58 mmol/mol).

Two health economic analyses will be conducted, a primary long-term analysis using the Sheffield T1D Policy Model and a secondary analysis of the data collected in the trial. All health economic analysis will compare the incremental cost-effectiveness ratio of DAFNE*plus* versus DAFNE to standard NICE thresholds to determine cost-effectiveness[32]. See supplementary material 3 for detailed plans for the economic evaluation.

For peer review only

Safety outcomes

Adverse Events

Study centres are only required to report as adverse events episodes of diabetic ketoacidosis and severe hypoglycaemia which while not requiring admission to hospital have been noted by either the participant or their relative/partner etc. These will be recorded on the data collection form and database.

Reporting

We do not anticipate many SAEs related specifically to DAFNE*plus* or standard DAFNE but will report any which are deemed related to the study intervention and or are unexpected to the Sponsor and the REC in line with best practice.

Sample size

It is expected that there will be 882 patients referred for DAFNE courses within the 15-month recruitment window and of these it is expected 75% (662 patients) will be recruited, equivalent to 47 participants at each of the 14 centres. From current DAFNE data, a further 25% are expected not to meet the primary analysis population criteria of baseline HbA1c greater than 7.5% (58 mmol/mol), leaving 497 participants. Finally, we anticipate 15% of participants to be lost to follow-up by the 12-month stage, therefore giving a primary analysis population of 422 participants. The sample size takes into account the design effect associated with the cluster design of the study. With an ICC of 1.5% (from previous DAFNE data) and 30 participants per cluster (422 participants over 14 centres) the design effect is 1.435 leaving the effective total sample size of n=294 participants (n=147 per arm).

Using a two sample comparison of mean HbA1c at the 12-month follow-up with 2-sided alpha of 5%, a correlation of 0.5 between baseline and final values and a standard deviation of 1.45 (from previous DAFNE data), the trial sample gives 92% power to detect a 0.5% difference in HbA1c between the two treatment groups in the study.

For peer review only

Table 1 – List of outcome and process measures

Concepts	Questionnaire	Baseline: Pre-course appt	Course Completion	Post-course assessments			
				3m*	6m*	9m*	12m*
**Demographic / Clinical**							
Glycaemic Control (HbA1c)	N/A	✓			✓		✓
Lipids	N/A	✓					✓
Body mass index (height/weight)	N/A	✓			✓		✓
Blood Pressure	N/A	✓			✓		✓
Episodes of severe Hypoglycaemia	N/A	✓			✓		✓
Episodes of Ketoacidosis	N/A	✓			✓		✓
Demographics	Individual items	✓			✓		✓
Hypoglycaemia awareness	Gold score[33] and DAFNE hypo awareness measure	✓			✓		✓
**Primary Psychological Outcomes**							
Diabetes-specific quality of life	ADDQoL-15[27]	✓			✓		✓
**Secondary Psychological Outcomes**							
Diabetes distress	Problem Areas In Diabetes (PAID-11) (short-form)[34]	✓			✓		✓
Diabetes-specific quality of life	Dawn Impact of Diabetes Profile (DIDP)[35]	✓			✓		✓
Diabetes-specific positive well-being	4-item sub-scale of the Well Being Questionnaire (W-BQ28)[36]	✓			✓		✓
Fear of hypoglycaemia	Hypoglycaemia Fear Survey-11 (HFS-11) short-form)[37]	✓			✓		✓
Health status	Health and Self-Management in Diabetes (HASMID)[38]	✓			✓		✓
Health status	EQ-5D-5L[39]	✓			✓		✓
Healthcare utilisation	Individual items	✓			✓		✓
Resource allocation	Individual items		✓				
**Process Measures**							

For peer review only

Concepts	Questionnaire	Baseline: Pre-course appt	Course Completion	Post-course assessments			
				3m*	6m*	9m*	12m*
Diabetes Management Experiences (satisfaction)	Diabetes Management Experiences Questionnaire (DME-Q)[40]	✓	✓	✓		✓	
Self-regulatory skills/behavioural regulation	Self-Regulation Questionnaire (SRQ-T1D)*[41]	✓	✓	✓		✓	
Diabetes strengths and resilience	Diabetes Strengths and Resilience Questionnaire (DSRQ)[42]	✓	✓	✓		✓	
Beliefs about capabilities: diabetes self-care	Confidence in Diabetes Scale (CIDS)*[43]	✓	✓	✓		✓	
Beliefs about capabilities: hypoglycaemia confidence	Hypoglycaemia Confidence Scale (HCS)[44]	✓	✓	✓		✓	
Diabetes-specific self-care behaviours	Diabetes Self-Care Behaviours (SCB-T1D)[45]	✓	✓	✓		✓	
Beliefs about consequences of engaging in DAFNE behaviours and weaving diabetes management into everyday routines	Individual items*	✓	✓	✓		✓	
Evaluation of technology (DAFNE*plus* website in intervention group and bolus calculator in control group)	System Usability Scale[46]		✓	✓		✓	

*Description about the development and modifications of these questionnaires and individual items are detailed in supplementary material 4

For peer review only

Statistical analysis

The primary analysis population will be participants that had an HbA1c greater than 7.5% (58 mmol/mol) at baseline and the analysis will be completed on an intention to treat (ITT) basis. This primary analysis is to assess the difference between the two treatment groups on the participant's HbA1c at the 12-month follow-up appointment which will be completed using a multiple linear regression model with coefficients estimated using generalised estimating equations (GEE) to account for the clustering design. A 95% confidence interval for the difference between the two treatment groups will be presented. Appropriate covariates will be included in the model, along with the participant's baseline HbA1c, to adjust the treatment effect accordingly.

The secondary analysis population is all consenting participants in the trial and analysis will again be completed on an ITT basis. This population will also be used to assess the difference in psychological outcomes between the two treatment groups using the same model as for the primary analysis.

A full statistical analysis plan has been written and was circulated to the Trial Management Group and Trial Steering Committee before being signed-off. This is available in supplementary material 5. All analysis results will be reported according to the revised CONSORT 2010 statement for cluster RCTs[47].

Data collection and management

Case report forms will be completed by DAFNE facilitators/educators at each study visit. Follow-up questionnaires will be self-completed by participants at each follow-up point. Participants will be allocated a unique identification number to identify them throughout the trial.

Plans to promote retention and follow-up of all trial participants include research appointments being scheduled and followed up by their clinical teams at 6 and 12-months. Overdue questionnaires are followed-up with an email reminder and then telephone call from CTRU. All participants received email newsletters to update them on trial progress.

Data will be entered onto the DAFNE*plus* database on CTRU's secure online system, hosted on University of Sheffield servers. Access is restricted such that users can enter and view only information required to perform their role.

Identifiable data will be shared with CTRU and the supporting study team and DAFNE*plus* website teams. Consent will be obtained from the participant for this to occur. Data will be stored securely on access-restricted network drive folders in accordance with CTRU standard operating procedures (SOPs).

All consent forms and questionnaires will be kept in a locked filing cabinet in a secured area and will be retained for a minimum of 5 years after study completion, in accordance with the sponsor's archiving requirements. Sheffield CTRU may request consent forms to be sent from the research site to the CTRU via post or email as part of remote monitoring procedures.

For peer review only

The nature, frequency and intensity of trial monitoring will be outlined in the site monitoring plan, which will be devised in accordance with CTRU SOPs.

Trial oversight committees

Two oversight committees have been established to oversee the conduct of this trial – Trial Steering Committee (TSC) and Trial Management Group (TMG), the composition of each is listed at the beginning of this paper. A Data Monitoring and Ethics Committee has not been convened, on the grounds that the study is low risk, in line with CTRU SOP GOV003. This has been approved by the Sponsor and TSC.

Research ethics approval

The RCT was not initiated until the protocol, informed consent forms and participant information sheets received approval from the Research Ethics Committee, the Health Research Authority and local Capacity and Capability is confirmed by the respective National Health Service Research & Development departments. MHRA approval was not required for this study.

The RCT is being conducted in accordance with the ethical principles that have their origin in the Declaration of Helsinki[48]; the principles of Good Clinical Practice, and the UK Framework for Health and Social Care Research[49].

Patient and Public Involvement

In addition to the patient representation on the TSC and TMG, this trial is supported by a Patient Advisory Group who have and will continue to meet regularly during the conduct of the trial (and the wider programme grant). Patient input has been sought throughout on the trial and intervention design, the informational material to support trial conduct and patient burden.

Dissemination Policy

Outputs from the trial will be generated in accordance with the communication and dissemination strategy. A number of academic outputs will be produced as the data are analysed from the trial. Journals will be selected based on the highest possible impact. Other stakeholder specific outputs in relevant formats will also be produced for commissioners, third sector, and user advocacy organisations.

For peer review only

Acknowledgements

The authors would like to thank all of the patients who are taking part in this trial, and all the participating clinical sites. The authors would also like to thank Becky Brown for administrative support to the trial, and Chris Turtle for data management.

Conflicts of interest

SA reports Advisory Board membership for Novonordisk, Medtronic, Roche and Abbott. MC reports personal fees from University of Sheffield. DP reports non-financial support from Novo Nordisk, Eli Lilly and Company Limited, Abbott Diabetes Care, Sanofi-Aventis and Medtronic. SH reports Advisory board membership and consultancy for Sanofi-Aventis, Boeringher-Ingelheim, Eli-Lilly, Novo-Nordisk, Springer Medical, Zealand Pharma and UN-EEG. Personal fees for speaker panels for Novo-Nordisk. JS reports Personal fees, non-financial support and other (advisory board membership/support to attend educational meetings) from Medtronic, Roche Diabetes Care and Sanofi Diabetes, grants and personal fees from Abbott Diabetes Care, grants and personal fees from AstraZeneca.

Data statement

No data from this protocol are available. Only approved personnel may access the data. Data and statistical code will be available on request.

Author contributions

ECo, SH, JE, JL, NdZ, NT, DP, SSF, SA, CG, DC, JS, TC, FL, DR contributed to the design of the protocol and drafted or critically revised the manuscript. CLC, ECr, PC and DH also critically revised the manuscript. ECo prepared the first draft of the manuscript and edited this with input from other authors. All authors reviewed and approved the final manuscript.

For peer review only

References

1 Diabetes UK. Diabetes UK: Facts and Stats, October 2016. 2016.
2 Group TDC and CTR. The Effect of Intensive Treatment of Diabetes on the Development and Progression of Long-Term Complications in Insulin-Dependent Diabetes Mellitus. *N Engl J Med* 1993;**329**:977–86. doi:10.1056/NEJM199309303291401
3 Heller S. Hypoglycaemia and diabetes. In: Pickup, J, WIlliams G, ed. *Textbook of Diabetes*. Oxford: : Blackwell Science 2003. 33.1-33.19.
4 DAFNE. DAFNE Home. http://www.dafne.uk.com/ (accessed 26 Feb 2020).
5 DAFNE Study Group DS. Training in flexible, intensive insulin management to enable dietary freedom in people with type 1 diabetes: dose adjustment for normal eating (DAFNE) randomised controlled trial. *BMJ* 2002;**325**:746.http://www.ncbi.nlm.nih.gov/pubmed/12364302 (accessed 30 Jan 2018).
6 Heller S, Lawton J, Amiel S, *et al.* Improving management of type 1 diabetes in the UK: the Dose Adjustment For Normal Eating (DAFNE) programme as a research test-bed. A mixed-method analysis of the barriers to and facilitators of successful diabetes self-management, a health economic analysis, a cluster randomised controlled trial of different models of delivery of an educational intervention and the potential of insulin pumps and additional educator input to improve outcomes. *Program Grants Appl Res* 2014;**2**:1–188. doi:10.3310/pgfar02050
7 National Institute for Health and Care Excellence. Type 1 diabetes in adults: diagnosis and management | Guidance and guidelines | NICE. NICE https://www.nice.org.uk/guidance/ng17 (accessed 2 Feb 2018).
8 McKnight JA, Wild SH, Lamb MJE, *et al.* Glycaemic control of Type 1 diabetes in clinical practice early in the 21st century: an international comparison. *Diabet Med* 2015;**32**:1036–50. doi:10.1111/dme.12676
9 Rankin D, Barnard K, Elliott J, *et al.* Type 1 diabetes patients' experiences of, and need for, social support after attending a structured education programme: A qualitative longitudinal investigation. *J Clin Nurs* 2014;**23**:2919–27. doi:10.1111/jocn.12539
10 Lawton J, Rankin D, Cooke DD, *et al.* Self-treating hypoglycaemia: A longitudinal qualitative investigation of the experiences and views of people with Type 1 diabetes. *Diabet Med* 2013;**30**:209–15. doi:10.1111/dme.12007
11 Lawton J, Rankin D, Cooke D, *et al.* Patients' experiences of adjusting insulin doses when implementing flexible intensive insulin therapy: A longitudinal, qualitative investigation. *Diabetes Res Clin Pract* 2012;**98**:236–42. doi:10.1016/j.diabres.2012.09.024
12 Rankin D, Cooke DD, Elliott J, *et al.* Supporting self-management after attending a structured education programme: A qualitative longitudinal investigation of type 1 diabetes patients experiences and views. *BMC Public Health* 2012;**12**:652. doi:10.1186/1471-2458-12-652
13 Rankin D, Cooke DD, Heller S, *et al.* Experiences of using blood glucose targets when following an intensive insulin regimen: A qualitative longitudinal investigation involving patients with Type1 diabetes. *Diabet Med* 2012;**29**:1079–84. doi:10.1111/j.1464-5491.2012.03670.x
14 Lawton J, Rankin D, Cooke DD, *et al.* Dose Adjustment for Normal Eating: A qualitative longitudinal exploration of the food and eating practices of type 1 diabetes patients converted to flexible intensive insulin therapy in the UK. *Diabetes Res Clin Pract* 2011;**91**:87–93. doi:10.1016/j.diabres.2010.11.007
15 Rankin D, Cooke DD, Clark M, *et al.* How and why do patients with Type1 diabetes sustain their use of flexible intensive insulin therapy? A qualitative

For peer review only

longitudinal investigation of patients' self-management practices following attendance at a Dose Adjustment for Normal Eating (DAFNE) course. *Diabet Med* 2011;**28**:532–8. doi:10.1111/j.1464-5491.2011.03243.x
16 Campbell MJ, Walters SJ. *How to Design, Analyse and Report Cluster Randomised Trials in Medicine and Health Related Research*. Chichester, UK: : John Wiley & Sons, Ltd 2014. doi:10.1002/9781118763452
17 Elliott J, Lawton J, Rankin D, *et al.* The 5x1 DAFNE study protocol: a cluster randomised trial comparing a standard 5 day DAFNE course delivered over 1 week against DAFNE training delivered over 1 day a week for 5 consecutive weeks. *BMC Endocr Disord* 2012;**12**:28. doi:10.1186/1472-6823-12-28
18 Craig P, Dieppe P, Macintyre S, *et al.* Developing and evaluating complex interventions: the new Medical Research Council guidance. *BMJ* 2008;**337**:a1655. doi:10.1136/BMJ.A1655
19 Michie S, van Stralen MM, West R. The behaviour change wheel: A new method for characterising and designing behaviour change interventions. *Implement Sci* 2011;**6**:42. doi:10.1186/1748-5908-6-42
20 Michie S, Richardson M, Johnston M, *et al.* The Behavior Change Technique Taxonomy (v1) of 93 Hierarchically Clustered Techniques: Building an International Consensus for the Reporting of Behavior Change Interventions. *Ann Behav Med* 2013;**46**:81–95. doi:10.1007/s12160-013-9486-6
21 Michie, S, Atkins, L, West R. *The behaviour change wheel: a guide to designing interventions*. London: : Silverback Publishing 2014.
22 Campbell F, Lawton J, Rankin D, *et al.* Follow-Up Support for Effective type 1 Diabetes self-management (The FUSED Model): A systematic review and meta-ethnography of the barriers, facilitators and recommendations for sustaining self-management skills after attending a structured education programme. *BMC Health Serv Res* 2018;**18**:898. doi:10.1186/s12913-018-3655-z
23 Speight J, Conn J, Dunning T, *et al.* Diabetes Australia position statement. A new language for diabetes: Improving communications with and about people with diabetes. *Diabetes Res Clin Pract* 2012;**97**:425–31. doi:10.1016/j.diabres.2012.03.015
24 Li F, Lokhnygina Y, Murray DM, *et al.* An evaluation of constrained randomization for the design and analysis of group-randomized trials. *Stat Med* 2016;**35**:1565–79. doi:10.1002/sim.6813
25 REPOSE Study Group TRS. Relative effectiveness of insulin pump treatment over multiple daily injections and structured education during flexible intensive insulin treatment for type 1 diabetes: cluster randomised trial (REPOSE). *BMJ* 2017;**356**:j1285. doi:10.1136/BMJ.J1285
26 International Hypoglycaemia Study Group. Glucose Concentrations of Less Than 3.0 mmol/L (54 mg/dL) Should Be Reported in Clinical Trials: A Joint Position Statement of the American Diabetes Association and the European Association for the Study of Diabetes: Table 1. *Diabetes Care* 2017;**40**:155–7. doi:10.2337/dc16-2215
27 Bradley C, Todd C, Gorton T, *et al.* The development of an individualized questionnaire measure of perceived impact of diabetes on quality of life: The ADDQoL. *Qual Life Res* 1999;**8**:79–91. doi:10.1023/A:1026485130100
28 SPEIGHT J, HOLMES-TRUSCOTT E, HENDRIECKX C, *et al.* 809-P: Comparison of the Acceptability and Psychometric Properties of Scales Assessing the Impact of Type 1 Diabetes on Quality of Life: Results of the Your SAY: QoL Study. *Diabetes* 2019;**68**:809-P. doi:10.2337/db19-809-p
29 Cooke D, Bond R, Lawton J, *et al.* Modeling predictors of changes in glycemic control and diabetes-specific quality of life amongst adults with type 1 diabetes 1 year after structured education in flexible, intensive insulin therapy. *J Behav Med* 2015;**38**:817–29. doi:10.1007/s10865-015-9649-y

30 Michie S, Johnston M, Abraham C, *et al.* Making psychological theory useful for implementing evidence based practice: a consensus approach. *Qual Saf Heal Care* 2005;**14**:26–33. doi:10.1136/qshc.2004.011155

31 Borrelli B. The assessment, monitoring, and enhancement of treatment fidelity in public health clinical trials. *J Public Health Dent* 2011;**71**:S52–63. doi:10.1111/j.1752-7325.2011.00233.x

32 National Institute for Health and Care Excellence. Guide to the methods of technology appraisal 2013 | Guidance and guidelines | NICE. https://www.nice.org.uk/process/pmg9/chapter/foreword (accessed 30 Jan 2018).

33 Gold AE, MacLeod KM, Frier BM. Frequency of severe hypoglycemia in patients with type I diabetes with impaired awareness of hypoglycemia. *Diabetes Care* 1994;**17**:697–703.http://www.ncbi.nlm.nih.gov/pubmed/7924780 (accessed 30 Jan 2018).

34 Polonsky WH, Anderson BJ, Lohrer PA, *et al.* Assessment of diabetes-related distress. *Diabetes Care* 1995;**18**:754–60.http://www.ncbi.nlm.nih.gov/pubmed/7555499 (accessed 30 Jan 2018).

35 Peyrot M, Rubin RR, Lauritzen T, *et al.* Psychosocial problems and barriers to improved diabetes management: results of the Cross-National Diabetes Attitudes, Wishes and Needs (DAWN) Study. *Diabet Med* 2005;**22**:1379–85. doi:10.1111/j.1464-5491.2005.01644.x

36 Speight, J, Barendse, S, Bradley C. The W-BQ28: further development of the well-being Questionnaire to include diabetes-specific as well as generic subscales and new stress subscales. In: *British Psychological Society, Division of Health Psychology*. Leeds: 1999.

37 Cox DJ, Irvine A, Gonder-Frederick L, *et al.* Fear of hypoglycemia: Quantification, validation, and utilization. *Diabetes Care* 1987;**10**:617–21. doi:10.2337/diacare.10.5.617

38 Carlton J, Elliott J, Rowen D, *et al.* Developing a questionnaire to determine the impact of self-management in diabetes: giving people with diabetes a voice. *Health Qual Life Outcomes* 2017;**15**:146. doi:10.1186/s12955-017-0719-4

39 Herdman M, Gudex C, Lloyd A, *et al.* Development and preliminary testing of the new five-level version of EQ-5D (EQ-5D-5L). *Qual Life Res* 2011;**20**:1727–36. doi:10.1007/s11136-011-9903-x

40 Speight J, Holmes-Truscott E, Little SA, *et al.* Satisfaction with the Use of Different Technologies for Insulin Delivery and Glucose Monitoring Among Adults with Long-Standing Type 1 Diabetes and Problematic Hypoglycemia: 2-Year Follow-Up in the HypoCOMPaSS Randomized Clinical Trial. *Diabetes Technol Ther* 2019;**21**:619–26. doi:10.1089/dia.2019.0152

41 Brown J, Miller W, LewandowskiLA. The Self-Regulation Questionnaire. In: Creek, Van de L, Jackson T, eds. *Innovations in clinical practice: A source book*. Sarasota, Florida: : Professional Resource Press 1999. 281–9.

42 Hilliard ME, Iturralde E, Weissberg-Benchell J, *et al.* The Diabetes Strengths and Resilience Measure for Adolescents With Type 1 Diabetes (DSTAR-Teen): Validation of a New, Brief Self-Report Measure. *J Pediatr Psychol* 2017;**42**:995–1005. doi:10.1093/jpepsy/jsx086

43 Van Der Ven NCW, Weinger K, Yi J, *et al.* The confidence in diabetes self-care scale: Psychometric properties of a new measure of diabetes-specific self-efficacy in Dutch and U.S. patients with type 1 diabetes. *Diabetes Care* 2003;**26**:713–8. doi:10.2337/diacare.26.3.713

44 Polonsky WH, Fisher L, Hessler D, *et al.* Investigating Hypoglycemic Confidence in Type 1 and Type 2 Diabetes. doi:10.1089/dia.2016.0366

45 Mansell P, Grant L, Bond R, Pouwer F, Page N, Davies A, Stenner K, Elliott J, Hopkins D, Jones A, Vallis M, Speight J, Lawton J, Clark M, Heller S CD.

For peer review only

Assessment of Self-Care Behaviours focused on Glycemic Control in Type 1 Diabetes: Development and Validation of the SCB-T1D Questionnaire.
46 Brooke J. SUS-A quick and dirty usability scale. *Usability Eval Ind* 1996;**189**:4–7.
47 Campbell MK, Piaggio G, Elbourne DR, *et al.* Consort 2010 statement: extension to cluster randomised trials. *BMJ* 2012;**345**:e5661. doi:10.1136/BMJ.E5661
48 World Medical Association. World Medical Association Declaration of Helsinki: Recommendations guiding physicians in biomedical research involving human subjects. 1996.
49 Department of Health. UK Framework for Health and Social Care Research. 2017.

For peer review only

Figure 1 – RCT flow diagram

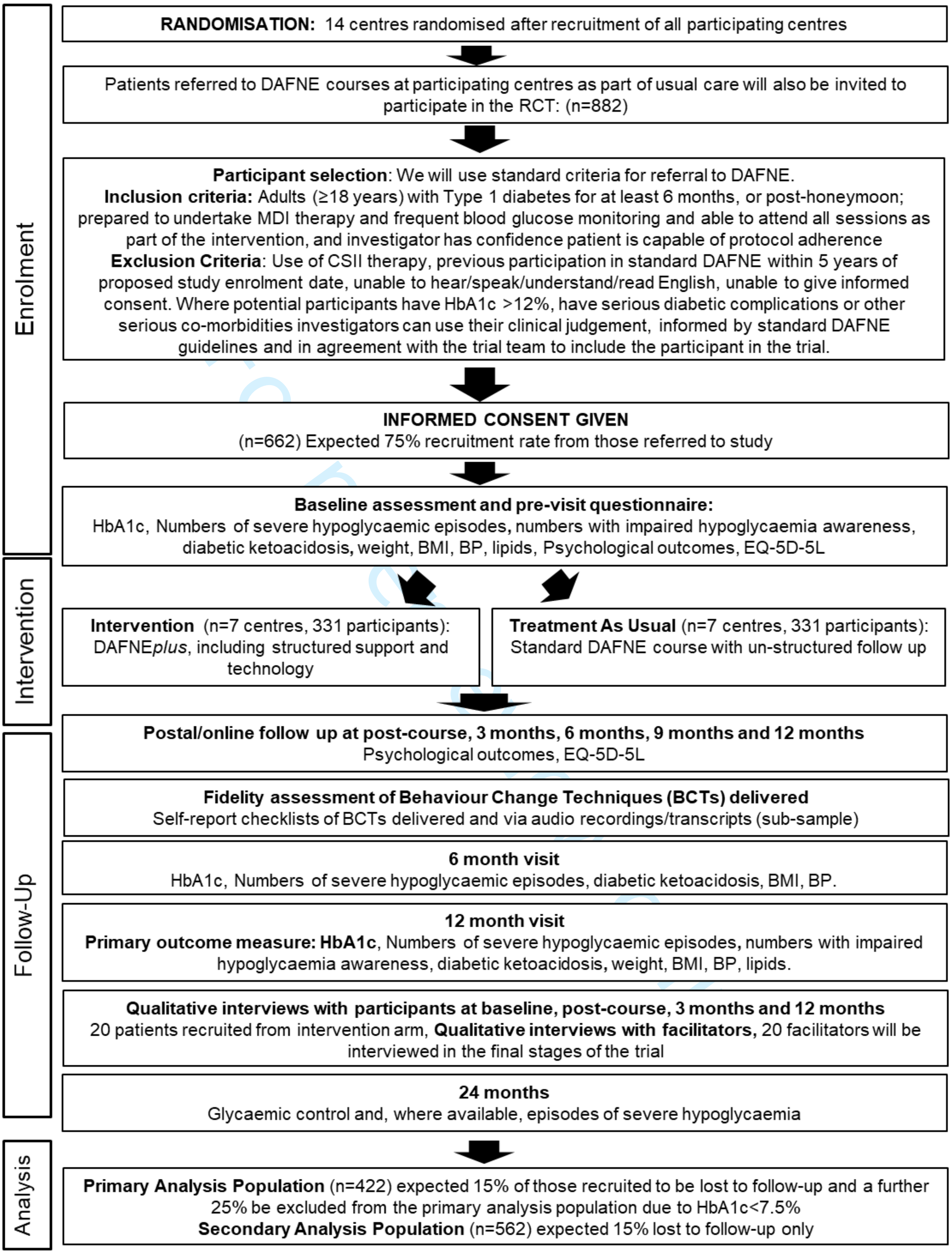

Supplementary material 1 – WHO Trial Registration Dataset

Data Category	Information
Primary registry and trial identifying number	http://www.isrctn.com/ISR ISRCTN42908016
Date of registration in primary registry	08/05/2018
Secondary identifying numbers	Sheffield CTRU: J13-003<br>Sponsor ID: STH20111<br>IRAS: 235621<br>Funding ref: RP-PG-0514-20013<br>REC: 18/SW/0100
Source(s) of monetary or material support	National Institute for Health Research (NIHR) (UK)
Primary sponsor	Sheffield Teaching Hospitals NHS Foundation Trust
Secondary sponsor(s)	N/A
Contact for public queries	Trial manager (Elaine Scott) 0114 222 5158 or dafneplus@sheffield.ac.uk
Contact for scientific queries	Trial manager (Elaine Scott) 0114 222 5158 or dafneplus@sheffield.ac.uk
Public title	DAFNE*plus* Cluster RCT
Scientific title	A cluster randomised controlled trial (RCT) of the DAFNE*plus* (Dose Adjustment for Normal Eating) intervention: A lifelong approach to promote effective self-management in adults with type 1 diabetes
Countries of recruitment	England and Scotland
Health condition(s) or problem(s) studied	Type 1 diabetes
Intervention(s)	DAFNE*plus* (Dose Adjustment for Normal Eating) intervention
Key inclusion and exclusion criteria	*Inclusion criteria:*<br>• Adults (≥18 years);<br>• Diagnosis of type 1 diabetes for at least 6 months, or post-honeymoon;<br>• Prepared to undertake multiple daily injection (MDI) therapy;<br>• Prepared to undertake frequent self-monitoring of blood glucose;<br>• Confirms availability to attend all sessions as part of the intervention;

	• Investigator has confidence that the patient is capable of adhering to all the trial protocol requirements.<br><br>*Exclusion criteria:*<br>• Current use of continuous subcutaneous insulin infusion (CSII) pump therapy<br>• HbA1c > 12%/108 mmol/mol (Investigators can use their judgement, informed by standard DAFNE guidelines and in agreement with the trial team, to include participants with HbA1c >12%/108 mmol/mol).<br>• Serious diabetic complications (e.g. blindness, renal dialysis). (Investigators can use their clinical judgement, informed by standard DAFNE guidelines and in agreement with the trial team).<br>• Other serious co-morbidities e.g. psychosis, diagnosed eating disorder (Investigators can use their clinical judgement, informed by standard DAFNE guidelines and in agreement with the trial team).<br>• Previous participation in standard DAFNE course less than 5 years before proposed study enrolment date<br>• Unable to speak/hear/understand/read write in English<br>• Unable to give written informed consent
Study type	Multi-centre cluster randomised controlled trial with process evaluation and economic evaluation, comparing DAFNE*plus* to standard DAFNE for adults with type 1 diabetes.
Date of first enrolment	01/09/2018
Target sample size	662 participants – 47 per centre.<br><br>Fourteen secondary care diabetes centres in the National Health Service in England and Scotland<br><br>In addition, we aim to recruit 20 DAFNE*plus* facilitators to take part in qualitative interviews for the process evaluation.
Recruitment status	Recruiting
Primary outcome(s)	The primary biomedical outcome is glycaemic control, defined as the change in HbA1c at 12 months (using a centralised assay to ensure standardisation), in those entering the trial with HbA1c >7.5% (estimated at 75% of those currently undertaking DAFNE courses based on our research database).
Key secondary outcomes	Secondary biomedical outcome:<br>Number of participants achieving either an HbA1c <7.5% (58 mmol/mol) or a decrease in HbA1c of ≥0.5% (≥5.5 mmol/mol) (using a centralised assay to ensure standardisation). These endpoints will be calculated using data collected at baseline and 12 months after the course.<br><br>Other secondary biomedical outcomes will include:<br>1. Severe hypoglycaemia, as defined by the American Diabetes Association, denotes severe

For peer review only

	cognitive impairment requiring external assistance for recovery, both rates and proportion of those affected, measured at baseline at 12 months after the course<br>2. Diabetic ketoacidosis, both rates and proportion of those affected, collected at baseline and 12 months after the course<br>3. Weight, measured at baseline and 12 months after the course<br>4. Body Mass Index, measured at baseline and 12 months after the course<br>5. Blood pressure, measured at baseline and 12 months after the course<br>6. Lipids, measured at baseline and 12 months after the course<br>7. Albumin/ creatinine, measured at baseline and 12 months after the course<br><br>The primary psychological outcome is the measurement at 12 months of the Audit-Dependent Diabetes Quality of Life Questionnaire (ADDQoL-15), a thirty-item measure of diabetes-specific quality of life.<br><br>Psychological outcomes, measured at baseline, course completion, 3, 6 and 12 months:<br>1. Dawn Impact of Diabetes Profile<br>2. Problem Areas in Diabetes Scale<br>3. Diabetes-specific positive well-being<br>4. Hypoglycaemia Fear Survey<br><br>Process measures:<br>5. Diabetes Management Experiences Questionnaire<br>6. Self-Regulation/Behavioural Regulation Questionnaire<br>7. Diabetes Strengths & Resilience Questionnaire<br>8. Confidence in Diabetes Scale assesses beliefs about capabilities (self-efficacy).<br>9. Diabetes Self-Care Behaviours<br>10. Hypoglycaemia Confidence Scale<br>11. Beliefs about consequences of engaging in DAFNE behaviours and weaving diabetes management into everyday routines.<br>12. The System Usability Score<br>13. Use and dose received of the DANFE*plus* programme assessed via logs of attendance at group and individual sessions, and use of the DANFE*plus* website<br><br>Hypoglycaemia Awareness<br>14. Hypoglycaemia awareness assessed via Gold score<br><br>Health economic measures assessed at baseline, course completion, 6 and 12 months using:<br>1. Health status – EQ-5D-5L<br>2. Health and Self-Management in Diabetes HASMID<br>3. Healthcare utilisation using a bespoke questionnaire<br>4. Contact between professionals and course participants will also be recorded at each site using questionnaires and data from the DANFE*plus* website (in the intervention arm)

For peer review only

Supplementary material 2 – Protocol for DAFNEplus Process evaluation

Aims and research questions

Understanding processes is as important as evaluating outcomes; process evaluations are complementary to outcomes evaluations and provide knowledge and information of equal value. Process evaluations aim to understand the functioning of an intervention by examining its implementation, mechanisms of impact and how contextual factors (i.e. factors external to the intervention/individual receiving the intervention) might affect its delivery and receipt [1,2]. Without this knowledge we may be able to establish from an outcome evaluation that an intervention 'works', but we will be presuming that the intended intervention was delivered and is effective, and we will not necessarily know how, or why, the intervention works and, hence, if it would have the same clinical and psychological effect if rolled out from a trial situation into routine clinical practice. With a complex intervention such as DAFNE*plus* it may well also be that some elements are more vital to its success than others; hence it is very important that we understand and explore the mechanisms of change on outcome from the perspectives of those receiving the intervention, as well as unintended consequences arising from the delivery and receipt of the intervention.

Our overarching research questions are:

1. **Does the DAFNE*plus* intervention 'work' in the ways intended? If not, why not?**

2. **What are the implications of the findings of the process evaluation for the rollout of DAFNE*plus* in routine clinical practice?**

To answer these over-arching questions, a series of over-lapping sub-questions will be explored:

a) What mechanisms change impact on glycaemic control? That is, how do the different elements of DAFNE*plus* (knowledge/skills, technological, structured follow-up), individuals' interaction with these elements, and individual psychological differences trigger <u>changes in</u> and <u>maintenance</u> <u>of</u> key diabetes self-management behaviours? The theoretical model underpinning the DAFNEplus programme assumes that diabetes self-management behaviours are among the principal determinants of glycaemic control.

b) What mechanisms of change impact on diabetes-specific quality of life?

c) What are participants' experiences of, and views about, key elements of the DAFNE*plus* intervention[1] and how do these influence and inform changes in, and maintenance of, key diabetes self-management behaviours over time?

---

[1] As a result of work undertaken in the pilot phase and MRC guidance to focus on key areas of uncertainty of greatest interest to academic and clinical audiences, a decision has been made to focus upon the technological and resilience/self-compassion elements of the programme.

For peer review only - http://bmjopen.bmj.com/site/about/guidelines.xhtml

d) To what extent is the intervention delivered as intended and are there variations between sites and individuals as to how the DAFNE*plus* intervention is delivered? What are the reasons for any variations?

e) What impact (practical and emotional) does intervention delivery have on facilitators and their workloads; what resourcing and support would facilitators and their colleagues need to deliver DAFNE*plus* in routine clinical practice?

f) Do any unintended consequences arise from the delivery and receipt of the DAFNE*plus* intervention, for participants and/or facilitators?

The data sources for each of the sub-questions are shown in table 1.

Table 1 – Data sources for the process evaluation

Research Question	Data source(s)
a) What mechanisms of change impact on glycaemic control?	• Questionnaire study<br>• Process outcomes<br>• Fidelity assessment<br>• Qualitative (from DAFNE*plus* pilot study)
b) What mechanisms of change impact on diabetes-specific quality of life	• Questionnaire study<br>• Process outcomes
c) What are participants' experiences of DAFNE*plus*?	Qualitative
d) To what extent is the intervention delivered as intended?	• Fidelity assessment<br>• Qualitative
e) What impact does intervention delivery have on facilitators and their workloads?	Qualitative
f) Do any unintended consequences arise from the delivery and receipt of the DAFNE*plus* intervention?	• Qualitative<br>• Questionnaire study<br>• Process outcomes<br>• Fidelity assessment

The process evaluation is composed of three interlinking components: (1) qualitative, (2) quantitative and (3) assessment of fidelity of delivery.

(1) Qualitative component

1.1 Overview

The qualitative component of the process evaluation will be informed by realist and Normalization Process theory (NPT) [3,4]. These choices arise from our recognition that context (i.e. factors external to the DAFNE*plus* intervention and/or the individual receiving the intervention) may influence how the intervention is delivered in different centres and how it is received by different individuals. It is also recognised that, when

For peer review only

a complex intervention, such as DAFNE*plus*, is implemented it can have unintended consequences, which may need to be investigated and, hence, that a flexible and adaptive study design will be required.

An iterative, inductive approach will be used wherein data analysis will commence as soon as data collection begins [5]. This will allow issues arising during early phases of qualitative data collection to inform questions asked in later phases and possibly also sampling. The qualitative research will also be responsive to other aspects of the process evaluation, including the fidelity work. Hence, while case studies will comprise the main element of the qualitative research (see below), costings have been included to allow, if necessary, one-off interviews to be undertaken with a 'booster' sample of patients, facilitators and/or other individuals in the event that the quantitative/fidelity components of the process evaluation highlight issues which require qualitative explanation. One example might be that, if the fidelity work highlights significant variations between trial sites as to how the DAFNE*plus* intervention is delivered, we may decide to interview additional facilitators to better understand why this might be the case.

1.2 Qualitative study design: case study approach

A case study approach will be used because it permits detailed exploration of if, how and why the intervention works in different contexts [6]. Each case will comprise: (a) participants who will be interviewed before, during and following completion of DAFNE*plus*, (b) their facilitators who will be interviewed after the participant's closeout from the trial, (c) information about the input and care the participant receives as part of DAFNE*plus* and their engagement with DAFNE*plus* technologies/resources. It will be possible to access this information via clinical records, the Glucollector website and information documented in case report forms and stored on PROSPECT (the CTRU database). Where identifiable clinical information needs transferring between NHS and University sites files will be encrypted and nhs.net accounts or Google Drive will be used. As part of the process evaluation, we will also have access to recordings of participants' face-to-face follow-up sessions with facilitators – these data are being collected for the fidelity assessment work, and data on utilisation of DAFNE*plus* technological components and adherence. Researchers from the University of Edinburgh will also sit in some DAFNEplus sessions as observers, to familiarise themselves with the processes and material to inform the case studies.

Participant Sampling

Two or three participants from each of the seven DAFNE*plus* sites will be selected for the qualitative work, and these individuals will be purposively sampled so there is representation of people of different ages, HbA1c levels, diabetes duration, gender, occupation, educational background, personal circumstance (e.g. single, partnered, parent) and place of residence (e.g. urban and rural locations).

Data collection: participant interviews

Selected participants will be interviewed at four time-points: prior to attending their course, following their course, and 3 and 12 months post course. Interviews will be informed by topic guides. Prior to undertaking a follow-up interview, a participant's previous interviews will be reviewed. As well as including more generic questions, follow-up interviews will be tailored to allow for follow-up of specific issues raised by particular individuals. Questions explored in the post course, 3 and 12 month

For peer review only

interviews will also take account of a review of information collected in medical records, via DAFNEplus technology and audio recorded follow-up sessions. Interviews will take place by telephone (unless an individual requests a face-to-face interview) at a time most convenient to the participant. All interviews will be digitally recorded with consent. It is anticipated that each interview will take 60 minutes to complete.

Facilitator interviews

Each participant's facilitators (n=1-2 per participant) will be interviewed following their close-out from the trial. If the participant received care from more than two facilitators as part of DAFNE*plus* we will ask them to nominate the two individuals from whom they felt they had the most input.

Facilitators will be interviewed once following the participant's close-out from the trial. This decision has been made partly for pragmatic reasons (i.e. we do not want to make excessive demands on the health professionals' time) and also because it will be possible to access information about the participant's care and the decisions made from the contact logs, clinical records and recordings of follow-up sessions. It is also recognised that, if facilitators are made aware that the participant is included in the process evaluation, this might influence or bias the care which is given, although participants will not be prohibited from telling their facilitators they are taking part in the qualitative research should they choose to do so.

Facilitator interviews will explore two key areas: (1) their views about, and experiences of, providing care and support to the case study participant; and (2) the facilitator's more general experiences of recruiting into the trial and delivering the DAFNE*plus* intervention.

Data collection: facilitator interviews

The facilitators' interviews will be informed by topic guides, although each individual's interview will also be tailored to explore issues specific to the participant who forms the focus of the case study (being careful to ensure that patient confidentiality is not breached). Interviews will take place by telephone at a time most convenient to the facilitator and will be digitally recorded.

1.3 Data analysis

Each participant's four interviews will be read through repeatedly and cross-compared with particular attention being paid to continuities and changes in their diabetes self-management practices over time, and the reasons for these. To aid comparison and identify where behaviour change has happened and why, 'critical incidents' will be extracted and compared (a 'critical incident' comprises data where a behaviour/decision/experience is described in detail, including the contextual and antecedent factors leading up to it and the consequences arising from it [7,8]). To help identify reasons for behaviour change, maintenance and lapses, data from the facilitator interviews, and recordings of follow-up sessions and case reports will also be used to help interpret and provide context to analysis of participant interviews.

Facilitator interviews will be cross-compared to identify issues and experiences which cut across different accounts [5]. Depending on the findings of the fidelity work, facilitator interviews may also be analysed in clusters (e.g. facilitators belonging to

For peer review only

‘adherent’ vs ‘non-adherent’ sites), to better understand reasons for individual/site differences in how the DAFNE*plus* intervention was delivered.

Key objectives of the analysis of the participant interviews are to better understand the mechanisms of impact in order to: (a) inform analysis of the quantitative data collected for the process evaluation; and (b) aid interpretation of quantitative data collected for both the process and outcomes evaluations. Key objectives of the analysis of the facilitator interviews are to offer insights which might: (a) aid interpretation of the participant case study data; (b) help explain findings from the fidelity work; (c) aid interpretation of trial outcome data; and, (d) offer insights relevant to decision-making about the possible rollout of DAFNE*plus* following the trial.

A key area for reflection

It needs to be recognised that, by interviewing participants at four time-points, and because of the kinds of questions which will be asked, the qualitative study could, potentially, have an impact on how this small group of participants understand, engage with, and experience the DAFNE*plus* intervention. Care will be made to emphasise to participants that the qualitative study is separate to DAFNE*plus* and it is our intention to understand their experiences rather than to influence their behaviours. Whilst we may not be able to diminish the impact that this has on these participants, we hope that the overall impact will be minimal due to the small sample size potentially affected.

(2) Quantitative component

2.1 Overview

A longitudinal, questionnaire study design has been adopted to determine the impact of the RCT on: a) our primary psychological outcome (diabetes-specific quality of life), b) secondary psychological outcomes and c) for the quantitative aspect of the process evaluation. That is, to identify the mechanisms of change that predict glycaemic control and diabetes-specific quality of life.

All participants in the intervention (DAFNE*plus*) and control (DAFNE) arms of the RCT will be given questionnaires to complete at baseline (up to 4 weeks prior to commencing the course) and at course completion, 3, 6, 9, 12 months post-course (see section 7 and Table 1). At baseline, the point at which participants will be more motivated to participate (pre-trial), they will be asked to complete all outcome and process questionnaire measures. To reduce participant burden, at course completion, 3- and 9-months they will only be asked to complete process measures. At 6 and 12-months they will be asked to complete the primary and secondary outcome measures only. Participants will be given the option of completing the questionnaire packs online or as a hard copy. Our choice of questionnaires (see section 7), assessing different constructs, have been selected according to existing knowledge about their association with the trial’s primary outcome (HbA1c) and diabetes-specific quality of life (primary psychological outcome), the results of the YOURSAY survey (unpublished), our former work with the DAFNE intervention, and based on the theoretical framework that underpins the new intervention development work and possible treatment mechanisms [9–11]. Brevity of the questionnaires and participant burden have also been a key consideration in our rationale for selection.

2.2 Analysis

For peer review only

The use of a repeated measures, longitudinal design will permit analysis of our primary and secondary psychological outcomes, as well as both the short- and long-term predictors and mediators of outcome (HbA1c and ADDQoL-15) using Structural Equation Modelling. SEM combines confirmatory and exploratory purposes. We will test our proposed model of the long-term predictors and mediators of outcome and then, if necessary, re-test this based on changes suggested by SEM modification indices [12]. The model will partially be informed by the qualitative work (described in section 1 above).

(3) Assessment of fidelity of delivery

3.1 Introduction

Behaviour change interventions are susceptible to variation in implementation, and are not always delivered as planned [13]. Intervention fidelity refers to the methodological strategies used to assess, monitor and enhance the integrity, that is, reliability and validity of behaviour change interventions [13]. The extent to which interventions are delivered as planned indicates internal and external validity, and needs to be known if the trial results are to be accurately interpreted and replicated. If fidelity is low, it is uncertain whether a change in outcome variables is due to the intended intervention, or to unknown factors that may have been added or omitted; alternatively, if no positive change is observed, it cannot be determined whether this is due to an inefficient intervention or a lack of intervention fidelity. This means that ineffective treatments risk being implemented and disseminated, and potentially effective treatments prematurely discarded [13].

The development of the content and structure of the DAFNE*plus* programme was informed by the Behaviour Change Wheel (BCW) framework [11]. The intervention's proposed functions are served by behaviour change techniques (BCTs), specified in the hierarchical Behaviour Change Technique Taxonomy v1 (BCTTv1; [14]), which are its 'active ingredients' [15]. The DAFNE*plus* intervention contains manual-specified BCTs as its active ingredients proposed to effect behaviour change (e.g. action planning, goal setting, and information on health consequences of the behaviour), together with principles for delivery specific to the DAFNE*plus* intervention that were identified during an expert consensus process (e.g. focus on the positives, emphasise individual autonomy). The fidelity analysis will involve assessment of the delivery of BCTs.

Fidelity of delivery of BCTs will also be assessed in the control arm of the trial (standard DAFNE) in order to identify any loss of treatment differentiation between the intervention and control arms as originally designed. Potentially loss of differentiation may result from low fidelity of delivery of additional content in the DAFNEplus programme, or additional content being delivered in the standard DAFNE programme, either unintentionally or as a result of contamination.

3.2 Aims and research questions

The aim is to explore the integrity of delivery of the DAFNE*plus* programme trialled in the RCT.

The research questions are:

- To what extent was the DAFNE*plus* programme delivered as specified in the protocols (course curriculum and follow-up scripts)? Specifically:

  - What proportion of manual-specified content (i.e. BCTs) was delivered by facilitators as intended during the programme sessions?
  - What additional, non-specified BCTs were delivered by facilitators?
  - How did the proportion of manual-specified content delivered differ across sessions and sites?
- What is the extent of treatment differentiation between the content of DAFNEplus and control (standard DAFNE) programmes delivered?

3.3 Methods

3.3.1 Design

A quantitative fidelity assessment, involving content analysis of intervention materials and transcripts of audio-recorded intervention sessions and provider self-rated fidelity checklists.

3.3.2 Observed fidelity of delivery assessment

The direct observation of fidelity via coding of session transcripts will provide an in-depth assessment of fidelity of delivery in a sub-sample of DAFNE*plus* and DAFNE courses.

Participants

Facilitators delivering either the DAFNE*plus* (intervention arm) or standard DAFNE (control arm) curriculum in 6 of the 14 participating sites will have their sessions recorded. It is assumed that each of these sites will have at least three facilitators delivering the DAFNE or DAFNE*plus* programmes (i.e. a minimum of 18 participants). Informed consent will be obtained from all participants at the selected sites (facilitators and patients). As part of the wider RCT., their participation in the programme during the course and follow-up sessions will be audio-recorded for training and research purposes.

Materials: Coding framework to assess observed fidelity of delivery

A coding framework will be developed to specify the BCTs to be delivered during the five face-to-face DAFNE*plus* days and the ~~four~~? follow-up sessions, and the standard DAFNE course sessions, as specified in the facilitator manual. For each BCT the coding framework will include a definition, examples and criteria for potential operationalisation in the context of the programme.

Sampling and procedure

Six sites (2 control and 4 intervention) will be purposively sampled for audio recording of all sessions. Selection will be informed by variables such as facilitator experience, previous research activity and site activity levels.

Course sessions, and where applicable follow up sessions, delivered face-to-face, will be audio-recorded in both the intervention (i.e. DAFNE*plus*) arm and control arm (i.e. standard DAFNE) [6] at selected sites. Written informed consent for audio-recording sessions will be sought from all participants and facilitators. Participants will be reassured that transcripts of audio-recorded sessions will be fully anonymised to remove any personal or identifiable information. Facilitators will be supplied with a digital audio-recorder and instructions for operating it. Facilitators will audio-record sessions and upload recordings to the University of Sheffield secure server via Google

For peer review only

Drive which will be accessed by the study manager and authorised members of the research team. Transcription will be performed by an external transcription service and a confidentiality agreement will be put in place with the transcribers to protect participant's data.

Each DAFNE*plus* programme comprises circa 40 sessions (one 1:1 pre-course session, 35 group 'course' sessions and four 1:1 follow-up sessions per participant). Each standard DAFNE course comprises circa 35 group course sessions per participant. Sessions will therefore be purposively sampled for transcription and analysis across both arms, selected sites and courses. Courses will be sampled according to key variables, e.g. geographical location, and the timeline for the trial with earlier courses preferentially sampled due to staff resource. Sessions may be sampled for transcription according to theoretical underpinning of the intervention, and evidenced relation to the outcome.

Analysis

Sampled content of sessions in both the intervention and control arms will be first specified by applying the developed coding framework to the sample of selected course transcripts. Two researchers will independently read through the session transcript line-by-line, using the coding framework to identify and categorise BCTs present in the facilitator's speech. Each identified BCT and delivery principle will be rated as fully, partially or not delivered according to the coding framework definition and criteria. Illustrative examples will be extracted into the framework.

To assess and establish inter-coder reliability, the researchers will meet frequently in coding workshops at the outset of coding (e.g. initially after coding every transcript [16]). Approximately twenty percent of transcripts will be double coded. Inter-rater reliability will be assessed by percentage agreement [17]. Reasons for discrepancies will be discussed, and the coding framework developed accordingly. Following Hardeman et al. [16], a minimum level of 75% inter-coder agreement [18], described as 'high' [19,20] will be considered acceptable. After inter-coder reliability has been established, researchers will code the remainder of transcripts independently.

Fidelity of delivery will be assessed following the methods of Hardeman et al. [16] and Lorencatto et al. [17]. Each of the BCTs specified in the DAFNE*plus* programme (intervention arm) or standard DAFNE programme (control arm) curriculum/scripts will be listed in a checklist, together with details such as session number and facilitator participant number. The BCTs specified in the coding framework will be rated as: 1) fully present, 2) partially present, or 3) absent but should be present. The proportion of BCTs delivered as intended will be assessed by dividing the number of fully/partially present BCTs by the total number of intended BCTs. Established criteria will be applied to classify extent of observed fidelity of delivery [6]: if < 50% of intended content is delivered this will be classified as 'low' fidelity; 51-79% as 'moderate' fidelity, and 80-100%' as 'high fidelity'.

Sessions will be grouped into types based on topics where applicable (for example, the four sessions covering action planning would be grouped into one type). An 'intended content' checklist will be produced for each session, and the session transcript will only be compared to the checklist for the corresponding session or session type, rather than comparison against the full curriculum. Variation in fidelity

For peer review only

will be examined according to site and session type. Delivery of any additional content will also be examined by assessing the frequency of delivery of any non-specified BCTs. This will serve to identify adaptations made whilst delivering DAFNE*plus*.

Treatment differentiation will be assessed by comparing the content analyses of transcripts from the intervention (DAFNE*plus*) and control (standard DAFNE) sessions. BCTs that are fully/partially delivered in transcripts from both arms will be compared, and the proportion of BCTs delivered in both arms assessed, with a higher proportion of common BCTs delivered representing less treatment differentiation.

3.3.3 Self-reported fidelity of delivery

Participants

All facilitators delivering either the DAFNE*plus* (intervention arm) or standard DAFNE (control arm) curriculum/scripts in each of the 14 participating sites will provide data for the fidelity of assessment delivery. It is assumed that each site will have at least three facilitators delivering the DAFNE or DAFNE*plus* programmes (i.e. a minimum of at least 42 participants). Informed consent will be obtained from all participants (facilitators and patients) as part of the wider RCT.

Materials: Facilitator self-rated checklists

To obtain a global snapshot of fidelity across all DAFNE*plus* courses, including those that are not transcribed and included in the observed fidelity assessment, self-reported facilitator checklists will also be developed and administered to all sites (intervention and control). The checklist will include provision of key information and BCTs that are intended to be delivered (i.e. as specified in the pre-course session script, course curriculum and follow-up support scripts), and how confident and competent the facilitators felt delivering the session components. Facilitators will also be asked to record reasons for any components not being fully delivered. Different checklists will be developed for each session. Due to the dynamic nature of the intervention and curriculum development it is not possible to provide definitive and finalised versions of these checklists at this time: the checklists will be finalised following the coding of the final version of DAFNEplus[2].

Procedure

Facilitators will be asked to complete the checklist at the end of each session where possible, or by the end of each day. They will forward completed checklists to Sheffield University CTRU by the end of each day. Facilitators will rate the extent to which they feel they delivered the intervention components listed in the checklists, from 0 (not at all), 1 (partially) to 2 (fully delivered).

Analysis

The proportion of intended components rated as partially/fully delivered by the facilitators will be calculated. The same criteria will be applied to classify extent of fidelity as in the observed measurements: if < 50% of intended content is delivered

[2] We are submitting specimen checklists with this revised version of the protocol, these checklists are subject to change as detailed above. The curriculum will be subject to change up until the point of recruitment and even after this point there might be minor changes which would require modification of the fidelity checklists. A requirement to submit these checklists after each change would be a major burden on both the ethics committee and the research team and therefore we seek permission to revise checklists without further approval and will not submit additional checklists unless requested to do so.

For peer review only - http://bmjopen.bmj.com/site/about/guidelines.xhtml

this will be classified as ‘low’ fidelity; 51-79% as ‘moderate’ fidelity, and 80-100%’ as ‘high fidelity’. Variation in proportion of fidelity of delivery will be examined across: session types, facilitators, and courses.

There are well documented discrepancies between what healthcare providers report delivering and actually deliver [21]. Therefore, for the DAFNE*plus* courses where session transcripts have also been coded (as described above), self-reported and objectively verified practice will be directly compared in terms of the proportion of BCTs facilitators report delivering, and that which was identified during the content analysis.

References

For peer review only

1 Moore G, Audrey S, Barker M, *et al.* Process evaluation of complex interventions UK Medical Research Council (MRC) guidance. https://www.mrc.ac.uk/documents/pdf/mrc-phsrn-process-evaluation-guidance-final/ (accessed 30 Jan 2018).

2 Moore GF, Audrey S, Barker M, *et al.* Process evaluation of complex interventions: Medical Research Council guidance. *BMJ* 2015;**350**:h1258. doi:10.1136/bmj.h1258

3 May CR, Mair F, Finch T, *et al.* Development of a theory of implementation and integration: Normalization Process Theory. *Implement Sci* 2009;**4**:29. doi:10.1186/1748-5908-4-29

4 May C, Finch T. Implementing, Embedding, and Integrating Practices: An Outline of Normalization Process Theory. *Sociology* 2009;**43**:535–54. doi:10.1177/0038038509103208

5 Strauss AL, Corbin JM. *Basics of qualitative research : grounded theory procedures and techniques*. Sage Publications 1990.

6 Borrelli B. The assessment, monitoring, and enhancement of treatment fidelity in public health clinical trials. *J Public Health Dent* 2011;**71**:S52–63. doi:10.1111/j.1752-7325.2011.00233.x

7 Flanagan JC. Psychological Bulletin THE CRITICAL INCIDENT TECHNIQUE. *JULY* 1954;**51.**https://books.apa.org/pubs/databases/psycinfo/cit-article.pdf (accessed 30 Jan 2018).

8 Butterfield LD, Borgen WA, Amundson NE, *et al.* Fifty years of the critical incident technique: 1954-2004 and beyond. *Qual Res* 2005;**5**:475–97. doi:10.1177/1468794105056924

9 Cooke D, Bond R, Lawton J, *et al.* Modeling predictors of changes in glycemic control and diabetes-specific quality of life amongst adults with type 1 diabetes 1 year after structured education in flexible, intensive insulin therapy. *J Behav Med* 2015;**38**:817–29. doi:10.1007/s10865-015-9649-y

10 Michie S, Johnston M, Abraham C, *et al.* Making psychological theory useful for implementing evidence based practice: a consensus approach. *Qual Saf Heal Care* 2005;**14**:26–33. doi:10.1136/qshc.2004.011155

11 Michie S, van Stralen MM, West R. The behaviour change wheel: a new method for characterising and designing behaviour change interventions. *Implement Sci* 2011;**6**:42. doi:10.1186/1748-5908-6-42

12 Bentler PM. On tests and indices for evaluating structural models. *Pers Individ Dif* 2007;**42**:825–9. doi:10.1016/J.PAID.2006.09.024

13 Bellg AJ, Borrelli B, Resnick B, *et al.* Enhancing Treatment Fidelity in Health Behavior Change Studies: Best Practices and Recommendations From the NIH Behavior Change Consortium. *Heal Psychol* 2004;**23**:443–51. doi:10.1037/0278-6133.23.5.443

14 Michie S, Richardson M, Johnston M, *et al.* The Behavior Change Technique Taxonomy (v1) of 93 Hierarchically Clustered Techniques: Building an International Consensus for the Reporting of Behavior Change Interventions. *Ann Behav Med* 2013;**46**:81–95.

doi:10.1007/s12160-013-9486-6

15 Michie, S, Atkins, L, West R. *The behaviour change wheel: a guide to designing interventions*. London: : Silverback Publishing 2014.

16 Hardeman W, Michie S, Fanshawe T, *et al.* Fidelity of delivery of a physical activity intervention: Predictors and consequences. *Psychol Health* 2008;**23**:11–24. doi:10.1080/08870440701615948

17 Lorencatto F, West R, Bruguera C, *et al.* A method for assessing fidelity of delivery of telephone behavioral support for smoking cessation. *J Consult Clin Psychol* 2014;**82**:482–91. doi:10.1037/a0035149

18 Lombard M, Snyder-Duch J. Content Analysis in Mass Communication Assessment and Reporting of Intercoder Reliability. *Hum Commun Res* 2002;**28**:587–604.http://marketing.univie.ac.at/fileadmin/user_upload/lehrstuhl_marketing/Lehre/Lehrinhalte/09SS/Lombard_et_al__2002_-1.pdf (accessed 30 Jan 2018).

19 Cohen J. Weighted kappa: nominal scale agreement with provision for scaled disagreement or partial credit. *Psychol Bull* 1968;**70**:213–20.http://www.ncbi.nlm.nih.gov/pubmed/19673146 (accessed 30 Jan 2018).

20 Altman DG. *Practical statistics for medical research*. Chapman and Hall 1991.

21 Hogue A, Dauber S, Lichvar E, *et al.* Validity of Therapist Self-Report Ratings of Fidelity to Evidence-Based Practices for Adolescent Behavior Problems: Correspondence between Therapists and Observers. *Adm Policy Ment Heal Ment Heal Serv Res* 2015;**42**:229–43. doi:10.1007/s10488-014-0548-2

For peer review only

For peer review only

Supplementary material 3 – Protocol for DAFNE*plus* Economic Evaluation

Aims and perspective

We will complete an economic evaluation as part of the study so that we are able to understand the cost-effectiveness of DAFNE*plus* compared to the standard DAFNE programme. The economic evaluation will follow guidance set by the National Institute for Health and Clinical Excellence for its Technology Appraisal process [1]. The analysis will take an NHS and personal social services perspective, measure health effects in quality adjusted life years (QALYs), discount future outcomes at 3.5% per annum and consider effects and costs over a lifetime time horizon. The primary analysis will be use long-term cost-effectiveness modelling, a secondary analysis will be an economic evaluation alongside the clinical trial (EEACT). The analysis population for all health economic analyses will consist of all participants in the DAFNEplus trial. A full Health Economic and Decision Modelling Analysis Plan (HEDMAP) will be written and circulated to the Trial Management Group and Programme Steering Committee before being signed-off.

Long-term cost-effectiveness modelling

In the long-term modelling exercise, the resulting evidence base will be incorporated into an updated Sheffield T1D Diabetes Policy Model [2]. This model has been used extensively in the evaluation of education and psychological interventions for people with T1D[3–6]. The time horizon of this analysis will be over each simulated individual's lifetime. As such, the long-term modelling will be considered as the primary health economic analysis. Demographic variables and some key resource use data (e.g. insulin use, contacts with NHS professionals) will be obtained from the trial data. The Sheffield T1D Diabetes Policy Model will be updated to use statistical models that estimate the clinical effects of DAFNE*plus* compared to DAFNE on HbA1c, the incidence of severe hypoglycaemia and the incidence of DKA. Two long-term modelling analyses will be conducted, the first will use the data collected by the one-year time point and will be submitted as part of the report to the NIHR on the DAFNE*plus* programme grant. This analysis will be updated after the two-year data collection is complete to incorporate the statistical analysis of the two-year follow up data. These statistical analyses of the clinical effects of DAFNE*plus* compared to DAFNE will be pre-specified in either the statistical analysis plan or the HEDMAP. The reporting of this evaluation will follow the Palmer *et al*[7] checklist for the reporting of model inputs to diabetes health economic studies.

Economic evaluation alongside the clinical trial

For the EEACT, we conduct the analysis in line with Ramsey *et al's* [8] recommendations for cost-effectiveness analysis alongside clinical trials. Specifically, we will collect data alongside the trial on intervention costs, associated healthcare resource use and a preference based utility measure: the EQ-5D-5L measure [9]. The intervention costing process will include training of educators, resource use, and adherence to structured follow up appointments, professional staff time and the technology component. A standard self-reported resource use questionnaire, used

For peer review only

previously in the DAFNE*plus* pilot (as well as the 5x1 DAFNE [10] and the REPOSE trials [11]), will ascertain NHS usage in terms of GP, community, outpatient, A&E and inpatients, as well as occurrence of DKA and hypoglycaemic events by level of severity. Unit costs will be taken from standard sources (NHS Reference Costs, British National Formulary, PSSRU). The standard self-reported resource use questionnaire and the EQ-5D-5L will be collected at baseline, 6 months and 12 months. Course costs (administrative and clinical) will be estimated using a bespoke questionnaire for completion by site staff. Our primary analysis will use the EQ-5D-5L valuation study to generate utility scores at baseline, course completion, 6 months and 12 months for each study participant [12]. There are on-going discussions about the valuation of the EQ-5D-5L, and NICE recently produced a position statement recommending that EQ-5D-5L data should be valued using mapping to the EQ-5D-3L and not the bespoke EQ-5D-5L value set [13,14]. Therefore our primary analysis will follow the most recent NICE guidance at the time of analysis, with the other valuation method been used in a sensitivity analysis. QALYs for each participant will be estimated by calculating the area under the curve defined by EQ-5D utility score, mortality and length of follow-up. The base case analysis will use the complete case data. In a scenario analysis, the missing data will be imputed. The time horizon of this analysis will be limited to the one-year time horizon of the trial. This evaluation will be considered as the secondary health economic analysis for two reasons: 1) The effects and costs of DAFNE*plus* may be incurred beyond the one-year trial time horizon (due to expected differences in the time to onset of diabetes related complications and potential maintenance of treatment effects beyond the trial period); and, 2) the DAFNEplus trial is not powered to detect differences in the incidence of long-term diabetes complications, as such the estimates of differences in the cost and QALYs between the two trial arms may be misleading.

Outcome measures and uncertainty analyses

In both the EEACT and the long term modelling the main outcome of interest will be the comparison of the incremental cost-effectiveness ratio (ICER) of DAFNE*plus* compared to DAFNE. The ICER will be compared to a maximum acceptable ICER of £20,000 per QALY gained, as this is the lower limit of the ICER range used by NICE to determine if an intervention is cost-effective [1]. Uncertainty in the ICER will be determined using: scenario analyses, subgroup analyses (pre-specified with the wider DAFNE*plus* team), probabilistic sensitivity analysis and expected value of information calculations. In particular, uncertainty in the cost-effectiveness of DAFNE*plus* as used in a wider rollout (compared to as utilised in the trial) and in subgroups of participants with a HbA1c less than 7.5% and greater than or equal to 7.5% will be explored in our scenario analyses.

References

1 National Institute for Health and Care Excellence. Guide to the methods of technology appraisal 2013 | Guidance and guidelines | NICE. https://www.nice.org.uk/process/pmg9/chapter/foreword (accessed 30 Jan 2018).

2 Thokala P, Kruger J, Brennan A, *et al.* Assessing the cost-effectiveness of Type 1 diabetes interventions: the Sheffield Type 1 Diabetes Policy Model. *Diabet Med* 2014;**31**:477–86. doi:10.1111/dme.12371

For peer review only - http://bmjopen.bmj.com/site/about/guidelines.xhtml

3 Kruger J, Brennan A, Thokala P, *et al.* The cost-effectiveness of the Dose Adjustment for Normal Eating (DAFNE) structured education programme: an update using the Sheffield Type 1 Diabetes Policy Model. *Diabet Med* 2013;**30**:1236–44. doi:10.1111/dme.12270

4 Heller S, Lawton J, Amiel S, *et al.* Improving management of type 1 diabetes in the UK: the Dose Adjustment For Normal Eating (DAFNE) programme as a research test-bed. A mixed-method analysis of the barriers to and facilitators of successful diabetes self-management, a health economic analysis, a cluster randomised controlled trial of different models of delivery of an educational intervention and the potential of insulin pumps and additional educator input to improve outcomes. *Program Grants Appl Res* 2014;**2**:1–188. doi:10.3310/pgfar02050

5 Kruger J, Pollard D, Basarir H, *et al.* Incorporating Psychological Predictors of Treatment Response into Health Economic Simulation Models. *Med Decis Mak* 2015;**35**:872–87. doi:10.1177/0272989X15590143

6 Pollard DJ, Brennan A, Dixon S, *et al.* Cost-effectiveness of insulin pumps compared with multiple daily injections both provided with structured education for adults with type 1 diabetes: A health economic analysis of the Relative Effectiveness of Pumps over Structured Education (REPOSE) randomised controlled trial. *BMJ Open* 2018;**8**:e016766. doi:10.1136/bmjopen-2017-016766

7 Palmer AJ, Si L, Tew M, *et al.* Computer Modeling of Diabetes and Its Transparency: A Report on the Eighth Mount Hood Challenge. *Value Heal* 2018;**21**:724–31. doi:10.1016/j.jval.2018.02.002

8 Ramsey SD, Willke RJ, Glick H, *et al.* Cost-Effectiveness Analysis Alongside Clinical Trials II—An ISPOR Good Research Practices Task Force Report. *Value Heal* 2015;**18**:161–72. doi:10.1016/j.jval.2015.02.001

9 Herdman M, Gudex C, Lloyd A, *et al.* Development and preliminary testing of the new five-level version of EQ-5D (EQ-5D-5L). *Qual Life Res* 2011;**20**:1727–36. doi:10.1007/s11136-011-9903-x

10 Elliott J, Lawton J, Rankin D, *et al.* The 5x1 DAFNE study protocol: a cluster randomised trial comparing a standard 5 day DAFNE course delivered over 1 week against DAFNE training delivered over 1 day a week for 5 consecutive weeks. *BMC Endocr Disord* 2012;**12**:28. doi:10.1186/1472-6823-12-28

11 REPOSE Study Group TRS. Relative effectiveness of insulin pump treatment over multiple daily injections and structured education during flexible intensive insulin treatment for type 1 diabetes: cluster randomised trial (REPOSE). *BMJ* 2017;**356**:j1285. doi:10.1136/BMJ.J1285

12 Devlin NJ, Shah KK, Feng Y, *et al.* Valuing health-related quality of life: An EQ-5D-5L value set for England. *Health Econ* Published Online First: 22 August 2017. doi:10.1002/hec.3564

13 National Institute for Health and Care Excellence. NICE's position statement on EQ-5D-5L NATIONAL INSTITUTE FOR HEALTH AND CARE EXCELLENCE Position statement on use of the EQ-5D-5L valuation set.

For peer review only

https://www.nice.org.uk/Media/Default/About/what-we-do/NICE-guidance/NICE-technology-appraisal-guidance/eq5d5l_nice_position_statement.pdf (accessed 30 Jan 2018).

14 van Hout B, Janssen MF, Feng Y-S, *et al.* Interim Scoring for the EQ-5D-5L: Mapping the EQ-5D-5L to EQ-5D-3L Value Sets. *Value Heal* 2012;**15**:708–15. doi:10.1016/j.jval.2012.02.008

For peer review only

Supplementary material 4 – Development and modification of questionnaires and individual items

Confidence in Diabetes Scale (CIDS)

This scale assesses the extent to which an individual believes they can engage in particular behaviours related to their type 1 diabetes treatment regimen (self-efficacy). The scale was published in 2003 and Dr Cooke contacted Prof Snoek, the senior author on the original validation paper to seek permission to amend item 3 on the scale (*I believe I can perform the prescribed number of daily insulin injections*). He gave his permission for the team to make the following amendment to reflect the more flexible approach to multiple daily insulin treatment regimens, advocated by courses like DAFNE and DAFNE*plus* (*I believe I can perform the number of daily insulin injections I need to*).

Self-Regulation Questionnaire for Type 1 Diabetes (SRQ-T1D)

This questionnaire is an adaptation of the Self-Regulation Questionnaire [1]. Self-regulation is the ability to develop, implement, and flexibly maintain planned behaviour in order to achieve one's goals. The SRQ and our adaptation of this builds on the work of Frederick Kanfer and two researchers who formulated a seven-step model of self-regulation [2,3]. Although this model was developed specifically to study addictive behaviours, the self-regulatory processes it describes are meant to be general principles of behavioural self-control. In this model, people may have problems managing certain behaviours (behavioural self-regulation) because of challenges at any of these seven steps:

1. **Receiving** relevant information
2. **Evaluating** the information and comparing it to norms
3. **Triggering** change
4. **Searching** for options
5. **Formulating** a plan
6. **Implementing** the plan
7. **Assessing** the plan's effectiveness (which recycles to steps 1 and 2)

The original SRQ has demonstrated reliability, concurrent and discriminant validity in community samples[4]. It consists of 63 items which was too long for our team to use in the DAFNE*plus* questionnaire pack, when this is one of several process measures. This measure was reviewed by 3 members from the DAFNE*plus* PPI group and by our process evaluation team consisting of clinicians, behavioural scientists, psychologists and social anthropologists, two of whom also have type 1 diabetes. The PPI group strongly recommended altering the wording of the individual items slightly so that these were all framed to be diabetes-specific in focus, rather than generic. Dr Cooke, in discussion with two members of the PPI group amended the wording of some of these items to ensure that they were clear and made sense. The process evaluation team and PPI group selected their top 2-3 items from each of the seven categories (above), rank ordering them. Dr Cooke then reviewed these to select the items from each

For peer review only

category which the majority had agreed should be included within the final questionnaire.

Beliefs about Consequences of Diabetes Self-Care Behaviours; Diabetes Support and Routines

The DAFNE*plus* revisions to the original DAFNE curriculum were structured around the Theoretical Domains Framework[5] hence it is very important to the process evaluation team to assess the constructs that are being targeted within individuals through the content and delivery of the DAFNE*plus* course; to assess whether participants in the DAFNE*plus* and standard DAFNE groups respond differently on these measures but also whether these constructs explain any differences in outcomes (HbA1c and diabetes-specific quality of life). Three of these constructs are 'social influences', 'beliefs about consequences of diabetes self-care' and 'environmental cues and prompts'. The research team have generated 11 diabetes-specific items to assess these constructs and have piloted them with our PPI group. These are unvalidated but once we have collected data at two timepoints (course completion and 3-months follow-up), if these measures are shown not to be psychometrically robust, we will remove these items from the 9-month follow-up point. Please note that we are only collecting these process measures at 3 timepoints.

References

1 Brown J, Miller W, LewandowskiLA. The Self-Regulation Questionnaire. In: Creek, Van de L, Jackson T, eds. *Innovations in clinical practice: A source book*. Sarasota, Florida: : Professional Resource Press 1999. 281–9.

2 Brown JM. Self-Regulation and the Addictive Behaviors. In: *Treating Addictive Behaviors*. Springer US 1998. 61–73. doi:10.1007/978-1-4899-1934-2_5

3 Miller W, Brown J. Self-Regulation as a Conceptual Basis for the Prevention and Treatment of Addictive Behaviours. In: Heather N, Miller W, Greenley J, eds. *Self-Control and the Addictive Behaviours*. Sydney: : Maxwell MacMillan Publishing Australia 1991. 3–79.

4 Aubrey L, Brown J, Research WM-AC& E, *et al.* Psychometric properties of a selfregulation questionnaire (SRQ). *Alcohol Clin Exp Res* 1994;**18**:429.

5 Atkins L, Francis J, Islam R, *et al.* A guide to using the Theoretical Domains Framework of behaviour change to investigate implementation problems. *Implement Sci* 2017;**12**:77. doi:10.1186/s13012-017-0605-9

For peer review only

Supplementary Material 5 – DAFNE*plus* SAP

Project Title: A Cluster Randomised Controlled Trial (CRCT) of the DAFNE*plus* (Dose for Adjustment for Normal Eating) intervention: A lifelong approach to promote effective self-management in adults with type 1 diabetes

Statistical Analysis Plan

Version 1 17th December 2019

Based on Protocol Version 6.0, dated 9th September 2019

REC: 18/SW/0100 ISRCTN: 42908016 Sheffield CTRU Job no. J13-003

Authored by

________________________________ ___/___/____
Nikki Totton Date
Statistician, CTRU, University of Sheffield

Approved by

________________________________ ___/___/_____
Mike Bradburn Date
Senior Statistician, CTRU, University of Sheffield

________________________________ ___/___/_____
Simon Heller Date
Chief Investigator, Sheffield Teaching Hospitals
NHS Foundation Trust

________________________________ ___/___/_____
Simon Griffin Date
DAFNE*plus* TSC Chair, University of Cambridge

Roles and Responsibilities

This SAP was initially drafted by Henry Nanji (previous DAFNE*plus* trial statistician) and updated by Nikki Totton (DAFNE*plus* trial statistician), with input from Mike Bradburn (senior statistician) and the trial team.

SAP Revision History

Version Number	Revision Date	Timing Within Trial	Description/Justification

For peer review only - http://bmjopen.bmj.com/site/about/guidelines.xhtml

List of Abbreviations and Definitions of Terms

**ADDQoL-14**	Audit of Diabetes Dependent Quality of Life (14 items)
**AE**	Adverse Event
**BMI**	Body Mass Index
**CIDS**	Confidence in Diabetes Scale
**CRF**	Case Report Form
**CTRU**	Clinical Trials Research Unit
**DAFNE**	Dose Adjustment For Normal Eating
**DIDP**	DAWN Impact of Diabetes Profile
**DKA**	Diabetic Ketoacidosis
**DME-Q**	Diabetes Management Experience Questionnaire
**DSRQ**	Diabetes Strengths and Resilience Questionnaire
**eGFR**	Estimated Glomerular Filtration Rate
**EQ-5D-5L**	EuroQol - 5 Dimensions - 5 Levels
**GCP**	Good Clinical Practice
**GLM**	Generalised Linear Model
**HASMID**	Health And Self-Management In Diabetes
**HbA1c**	Glycated Haemoglobin (Haemoglobin A1c)
**HCS**	Hypoglycaemia Confidence Scale
**HDL**	High-Density Lipoprotein
**HFS**	Hypoglycaemia Fear Survey
**ICC**	Intra-class Correlation Coefficient
**IQR**	Inter Quartile Range
**IRR**	Incidence Rate Ratio
**ISRCTN**	International Standard Randomised Controlled Trial Number
**ITT**	Intention To Treat
**LDL**	Low-Density Lipoprotein
**LRT**	Likelihood Ratio Test
**MD**	Mean Difference
**MDI**	Multiple Daily Injections
**NHS**	National Health Service
**OR**	Odds Ratio
**PAID**	Problem Areas In Diabetes
**PP**	Per Protocol
**QoL**	Quality of Life
**RCT**	Randomised Controlled Trial
**REC**	Research Ethics Committee
**SAE**	Serious Adverse Event
**SCB-T1D**	Self-Care Behaviours: Type 1 Diabetes
**SD**	Standard Deviation
**SOP**	Standard Operating Procedure
**SRQ-T1D**	Self-Regulation Questionnaire
**SUS**	System Usability Score
**T1D**	Type 1 Diabetes
**TMG**	Trial Management Group
**TSC**	Trial Steering Committee
**VAS**	Visual Analogue Scale
**W-BQ28**	Well-Being Questionnaire 28

For peer review only - http://bmjopen.bmj.com/site/about/guidelines.xhtml

Contents

1 Introduction 4
1.1 Background and Rationale 4
1.2 Objectives 4
2 Trial Methods 5
2.1 Trial Design 5
2.2 Randomisation and Blinding 5
2.3 Sample Size 5
2.4 Trial Framework 6
2.5 Trial Monitoring and Management 6
2.6 Interim Analysis and Stopping Rules 6
2.7 Timing of Final Analysis 6
2.8 Timing of Outcome Assessments 7
3 Statistical principles 8
3.1 Confidence Intervals and P Values 8
3.2 Adherence and Protocol Deviations 8
3.3 Analysis Populations 8
4 Screening, recruitment, demographics and withdrawal 9
4.1 Eligibility Criteria 9
4.2 CONSORT 10
4.3 Withdrawal of Participants 11
4.4 Baseline Characteristics 11
5 Outline of Statistical AnalysIs 13
5.1 Outcome Measures 13
5.1.1 Primary Outcome 13
5.1.2 Secondary Outcomes - Biomedical 13
5.1.3 Secondary Outcomes - Psychological 14
5.1.4 Secondary Outcomes - Process Measures 15
5.2 Analysis of Primary Outcome 16
5.2.1 Model Checking 17
5.3 Sensitivity Analyses of the Primary Outcome 17
5.4 Subgroup Analyses 17
5.5 Handling Missing Data 18
5.6 Analysis of Secondary Outcomes - Biomedical 19
5.6.1 HbA1c 19
5.6.2 Weight, BMI, Blood Pressure and Lipids 19
5.6.3 Severe Hypoglycaemia and DKA 19
5.6.4 Model Checking 20
5.7 Analysis of Secondary Outcomes - Psychological 20
5.8 Safety Outcomes 20
5.9 Statistical Software 20
6 References 21
6.1 Documents 21
6.2 Publications 21

For peer review only - http://bmjopen.bmj.com/site/about/guidelines.xhtml

1 INTRODUCTION

1.1 Background and Rationale

The successful management of Type 1 Diabetes (T1D) requires those affected (>300,000 adults in the UK) [1] to keep their glucose levels sufficiently close to normal to avoid long-term complications [2]. In this condition, unlike type 2 diabetes, there is an absolute insulin deficiency, and so insulin must be injected subcutaneously, and tablet therapy is not possible. Preventing complications depends upon an individual's ability to prevent hyperglycaemia (high blood glucose levels) by self-managing their condition. This is done by calculating precise insulin doses based on accurate estimations of food intake before every meal using frequent blood glucose measurements, and accounting for fluctuations in physical activity, illness, stress and hormones. Hypoglycaemia (low blood glucose levels), if severe, can result in acute cognitive impairment, confusion, collapse and injury, coma or even death [3].

Dose Adjustment For Normal Eating (DAFNE) is a clinical education programme run within the National Health Service (NHS), designed to teach and improve self-management skills in flexible intensive insulin therapy to improve both glucose control and quality of life in adults with T1D. It is a five-day training course for adults with T1D, delivered in small groups. The DAFNE*plus* programme grant has modified the existing DAFNE curriculum to incorporate techniques for initiating and sustaining behaviour change, structured follow-up support, and digital information communication technology.

1.2 Objectives

The primary objective of the trial is to:

1. Assess the effects of the intervention on glycaemic control as measured by glycated haemoglobin (HbA1c) at 12 months.

The secondary objectives of the trial are to:

1. Assess the medium term effect of the intervention on glycaemic control, as measured by HbA1c, using data at 6 months,
2. Assess the effects of the intervention on the diabetes-specific quality of life,
3. Assess the effects of the intervention on diabetes distress and other biomedical outcomes (severe episodes of hypoglycaemia, diabetic ketoacidosis, weight, body mass index, blood pressure and lipids),
4. Undertake a mixed methods process evaluation to aid understanding of the Randomised Controlled Trial (RCT) findings, and to inform decision making about the implementation of DAFNE*plus* in clinical care post-trial,
5. Assess fidelity of delivery of the DAFNE*plus* intervention,
6. Undertake a health economic analysis to determine the cost-effectiveness of DAFNE*plus* versus standard DAFNE.

Objectives 4, 5 and 6 under secondary objectives will not be considered as part of this SAP and will be dealt with separately to the main trial analysis.

For peer review only - http://bmjopen.bmj.com/site/about/guidelines.xhtml

2 TRIAL METHODS

2.1 Trial Design

The trial will use a pragmatic, parallel group, cluster randomised (1:1 allocation) controlled design involving 14 sites. Centre randomisation is required rather than individual since 'contamination' of the control arm may occur if educators are trained in DAFNE*plus* (intervention) and still are required to deliver standard DAFNE (control) [4]. Potential participants are identified by local diabetes clinicians and will use standard criteria for referral to DAFNE.

Participants recruited at control centres will receive treatment as usual and will attend the DAFNE course one day a week, over five consecutive weeks. A bolus calculator will be provided to support the calculation of insulin dose, but there will be no structured follow-up appointment beyond those provided in usual care.

Participants in centres allocated to the intervention will attend the DAFNE*plus* course one day a week, over five consecutive weeks, which includes the use of technology to transmit and display blood glucose data to support pattern recognition and interpretation. A bolus calculator to support insulin dose calculations will be provided and up to five structured follow-up appointments are offered in the 12 months after the course.

Further details on the trial design can be found in the protocol.

2.2 Randomisation and Blinding

Following ethical approval, all participating centres were randomised on a 1:1 basis to control (standard DAFNE courses) or the intervention arm (DAFNE*plus* course). In order to balance the centres within the two arms, a covariate constrained approach [5] was adopted matching the centres on the number of patients, number of educators and the total number of previous DAFNE courses delivered by the centre as stratification variables. Due to the nature of the intervention, The University of Sheffield Clinical Trials Research Unit (CTRU) in-house randomisation system (SCRAM) was not applicable and so in line with Standard Operating Procedure (SOP) ST007, a randomisation guidance document detailing the randomisation procedure has been written. The random allocation was conducted by the trial statistician using STATA [6] and therefore no outcome data which is split by treatment group will be seen by the statistician until the trial is complete to minimise bias. Further details on the randomisation and unblinding can be found in the protocol.

2.3 Sample Size

2.3.1 Original Sample Size

It is expected that there will be 882 patients referred for DAFNE courses within the 15-month recruitment window and of these, it is expected that 75% (662 patients) will be recruited, equivalent to 47 participants at each of the 14 centres. Based on data from current DAFNE courses, a further 25% are expected not to meet the primary analysis population criteria of a baseline HbA1c greater than 7.5%, leaving 497 participants. Finally, we anticipate 15% of participants to be lost to follow-up by the 12-month stage, therefore giving a primary analysis population of 422 patients. Taking into account a

For peer review only - http://bmjopen.bmj.com/site/about/guidelines.xhtml

design effect, due to the cluster design of the trial, with an Intra-class Correlation Coefficient (ICC) of 1.5% (from previous DAFNE data) and 30 patients per cluster (422 patients over 14 centres) the design effect is 1.435 leaving the effective total sample size of 294 participants (147 per arm).

Using a two-sample comparison of mean HbA1c at the 12-month follow-up with 2-sided alpha of 5%, a correlation of 0.5 between baseline and final values and a standard deviation of 1.45 (from previous DAFNE data), the trial sample gives 92% power to detect a 0.5% difference in HbA1c between the two treatment groups.

2.3.2 Updated Sample Size

The original calculations (i.e. worked backwards from the expected number of recruits) gave a power of 92.7%. The team discussed different options, but in light of the difficulties in enlisting new centres and therefore now 13 centres not 14, it was agreed by our Trial Steering Committee to continue with the original planned recruitment per centre, with 6 interventions and 7 control. Therefore, reducing the sample size solely in the intervention arm. This results in a power of 90.4% with a small imbalance between the two arms (ratio 1:1.67) with a reduced sample size of 615 (instead of 662). As this trial is cluster randomised and provided through courses, there was always likely to be some imbalance between the two treatment arms which is out of our control.

2.4 Trial Framework

The primary aim of this trial is to conduct a superiority cluster RCT comparing the new DAFNE*plus* intervention to the existing DAFNE to detect a minimum clinically significant difference of 0.5% in HbA1c between the two groups after 12 months.

2.5 Trial Monitoring and Management

In compliance with Sheffield CTRU's SOPs, the following committees will be established to govern the overall conduct and supervision of the trial:

- Trial Steering Committee (TSC)
- Trial Management Group (TMG)

The trial will be supervised on a day to day basis at Sheffield CTRU by the Trial Manager with supervision from the Chief Investigator and a Senior Trial Manager.

2.6 Interim Analysis and Stopping Rules

There are no interim analyses or early stopping planned for this trial, hence no stopping rules are applicable.

2.7 Timing of Final Analysis

The final analysis will take place after the last participants have completed their 12-month follow-up visit. All data will be analysed collectively at this time point. A further study will be completed in the future to complete analysis of data collected at the 24-month follow-up, but this is not included within this SAP and will be outlined separately.

For peer review only - http://bmjopen.bmj.com/site/about/guidelines.xhtml

2.8 Timing of Outcome Assessments

Table 1 below shows the biomedical and psychological outcome measures and the different time-points outcomes are measured. A detailed description of the outcome assessment is found under section 5.

Table 1: Outcomes measures within the trial and time points they will be collected at

Outcome Measure	Baseline	6 Months Post Course	12 Months Post Course
**Clinical Outcomes**			
Demographics[1]	x		
HbA1c	x	x	X
Severe Hypoglycaemic Episodes	x	x	x
Diabetic Ketoacidosis (DKA) Episodes	x	x	x
Body Mass Index (BMI)	x	x	x
Blood Pressure	x		x
Lipids (HDL, LDL)[2]	x		x
**Psychological Outcomes**			
ADDQoL-15 (Diabetes-specific quality of life)	x	x	x
DIDP (Diabetes-specific quality of life)	x	x	x
PAID-11 (Diabetes Distress)	x	x	x
W-BQ28 (Diabetes-Specific Wellbeing)	x	x	x
HFS-11 (Fear of Hypoglycaemia)	x	x	x
Gold Score (Hypoglycaemia awareness)	x	x	x
**Health Economic Measures**			
HASMID (Health Status)	x	x	x
EQ-5D-5L (Health Status)	x	x	x
**Process Measures**			
DSRQ	x		
SCB-T1D	x		
Usability Score			
DME-Q	x		
HCS	x		
Beliefs about Consequences	x		
SRQ-T1D	x		

1: Detail description of demographic characteristics are found under section 4.4

For peer review only - http://bmjopen.bmj.com/site/about/guidelines.xhtml

2: HDL=High Density Lipoprotein, LDL=Low Density Lipoprotein

3 STATISTICAL PRINCIPLES

This statistical analysis plan (SAP) is written in conjunction with the International Conference of Harmonisation topic E9 [7], applicable Standard Operating Procedures (SOP) from the Sheffield Clinical Trials Research Unit (CTRU) (ST001 and ST006).

3.1 Confidence Intervals and P Values

All statistical tests will be completed at the 5% significance level and estimates of the treatment effect will be reported with their associated 95% confidence intervals. All tests completed will be two-sided. The results of the trial are focussed on the primary endpoint (HbA1c at 12 months) so adjustment for multiple testing and control of the type 1 error rate is required.

3.2 Adherence and Protocol Deviations

Adherence to the standard DAFNE course within the control group is defined as:

- Attending a minimum of four of the five days within the course which must include the first two days.

Adherence to the DAFNE*plus* course within the intervention group is defined as:

- Attending a minimum of four of the five days within the course which must include the first two days *AND*
- Attending at least three of the five follow-up sessions (this can be any three sessions)

Adherence to the courses will be presented as the number and percentage of participants in each arm of those that adhered. Additionally, for the intervention group the number and percentage of participants that adhered to each of the two adherence requirements will be presented separately to show which, if either, of these are more prominent.

In the DAFNE*plus* trial, any intended failure to adhere to the protocol will be classed as protocol violation and may be minor or major while any unintended (non-serious) departures from the protocol would be considered as protocol deviations and all these will be reported.

Attendance will be captured on case report forms (CRFs) when participants attend the course. Participants who failed to meet this criterion will be classed as having a major protocol deviation.

The number (and percentage) of patients with major and minor protocol deviations will be summarised by treatment group with details of type of deviation provided. No formal statistical testing will be undertaken between the two groups.

3.3 Analysis Populations

The primary analysis set will be that defined in Intention To Treat (ITT) on the primary outcome. Additional analysis populations, such as Per Protocol (PP), will be used as sensitivity analyses. Table 2 defines each of the analysis sets.

For peer review only - http://bmjopen.bmj.com/site/about/guidelines.xhtml

Table 2 Definition of the analysis set

Analysis Set	Outcomes	Participant Inclusion Criteria
**Primary ITT**	Primary outcome only	All consented participants, analysed according to their centre's randomisation regardless of their adherence to the entry criteria, intervention received, subsequent withdrawal or deviation from the protocol unless they have explicitly requested that their data be removed [7]. In addition, participants must have a baseline HbA1c more than 7.5%.
**Full ITT**	Secondary outcomes and as a sensitivity analysis for the primary outcome	All consented participants, analysed according to their centre's randomisation regardless of their adherence to the entry criteria, intervention received, subsequent withdrawal or deviation from the protocol unless they have explicitly requested that their data be removed [7].
**PP**	Primary outcome only	All consented participants excluding those who didn't adhere to the assigned intervention as defined by section 3.2.

4 SCREENING, RECRUITMENT, DEMOGRAPHICS AND WITHDRAWAL

4.1 Eligibility Criteria

Centre eligibility:

- Adult diabetes centre currently delivering DAFNE
- At least three DAFNE educators trained in delivering the five-week model of DAFNE
- Delivery of sufficient DAFNE courses per year to recruit the trial sample.

Participant eligibility:
Inclusion and exclusion criteria for participants are outlined in Table 3.

Table 3: Patient eligibility criteria

Inclusion Criteria	Exclusion Criteria
Age ≥18 years	HbA1c >12%
Diagnosis of type 1 diabetes for ≥6 months or post-honeymoon	Current use of continuous subcutaneous insulin infusion (CSII) pump therapy
Prepared to undertake multiple daily injections (MDI) therapy and frequent self-monitoring of blood glucose	Serious diabetes-related complications (e.g. blindness, renal dialysis), or other serious co-morbidities (e.g. psychosis, diagnosed eating disorder)
Available to attend all sessions	Unable to hear/speak/understand/read/write in English

For peer review only - http://bmjopen.bmj.com/site/about/guidelines.xhtml

Investigator has confidence that the patient is capable of adhering to all the trial protocol requirements	Previous participation in standard DAFNE course less than 5 years before proposed trial enrolment date
	Unable to give informed consent.

4.2 CONSORT

Using guidelines from the CONSORT statement [8], the summaries outlined Table 4 will be calculated in order to construct a CONSORT flowchart. Data will be presented overall and by treatment arm to show if any differences are present due to the sites treatment allocation.

For peer review only

For peer review only - http://bmjopen.bmj.com/site/about/guidelines.xhtml

Table 4: CONSORT Summary

**Screening Data**	• Number of participants assessed for eligibility at screening<br>• Number ineligible including reasons<br>• Number eligible but declined to participate including reasons
**Recruitment Data**	• Number of participants consented and recruited<br>• Number and percentage of those who attended all five sessions within either DAFNE/DAFNE*plus* course<br>• Number and percentage of those who completed the primary outcome (HbA1c) at 6 and 12 months follow-up
**Lost to Follow Up/Withdrawal Data**	• Number and percentage of those consented who dropped out and withdrew before the course<br>• Number and percentage of those consented who dropped out and withdrew after completing the course but before the 6-month follow-up<br>• Number and percentage of those consented who dropped out and withdrew after the 6-month follow up but before the 12-month follow-up
**Analysis Population Data**	• Number of those included in primary ITT set at 6 and 12 months follow-up<br>• Number of those included in full ITT set at 6 and 12 months follow-up<br>• Number of those included in PP set at 12 months follow-up

4.3 Withdrawal of Participants

Details of potential reasons for withdrawal are found in the protocol and summaries of these reasons plus any additional reasons found within the trial will be presented overall and split by treatment arm. Withdrawal numbers will also be summarised dependent on if the participant has withdrawn from the intervention, but continue with follow-up, withdrawal from follow-up but allow data collected to date to be used, withdrawal for all data collected to date to be used, or lost to follow up.

4.4 Baseline Characteristics

Baseline characteristics will be summarised at both the centre and participant level to assess the balance between the two treatment arms.

At the centre level, the stratification variables used (number of patients, number of educators and the total number of previous DAFNE courses delivered) within the randomisation will be presented by treatment arm to evaluate the balance between centres.

At the participant level, the variables shown in Table 5 as captured at baseline will be presented overall and by treatment arm. Categorical variables will be presented using counts and percentages, continuous variables will be presented with means and standard deviations or median and inter-quartile ranges as appropriate. No statistical significance testing will be used to test baseline imbalances between the two groups but any noteworthy differences will be descriptively reported.

Table 5: Baseline variables

For peer review only

**Demographics**
Age (Years)
Gender
Ethnicity
Highest qualification
**Medical History**
Duration of diabetes (Years)
Previously attended a DAFNE course
Pregnancy since diabetes
Current pregnancy, if yes gestation (weeks)
Use of lipid lowering medication
Use of antiplatelet agent
Use of medication for depression
Smoking status, if yes no of cigarettes per day
Physical activity levels
Complications – conditions and events (list as applicable)
Quick acting insulin (average daily dose, number of injections per day and type)
Background insulin (average daily dose, number of injections per day and type)
Pre-mixed insulin (average daily dose, number of injections per day and type)
Use of ratios
Presence of Lipohypertrophy
Number of blood glucose test performed (last 2 weeks)
Use of CGM, method and length of use (for Libre only – how it is funded and how it's being used)
Use of apps
Severe hypoglycaemic episode in the last year
Number of hypoglycaemic episodes that were unable to be treated themselves
Number of hypoglycaemic episodes that required paramedic assistance
Number of hypoglycaemic episodes that required A&E attendance
Number of hypoglycaemic episodes that required hospital admission
Blood glucose of hypoglycaemia
Admissions due to DKA (ever and in last year)
**Labs and Vital Signs**
BMI (kg/m2)
Blood pressure (mmHg)
HbA1c (mmol/mol)
Creatinine (µmol/L)
Albumin-creatinine (mg/mmol)
Cholesterol (mmol/L)
Triglycerides (mmol/L)
High Density Lipoprotein (HDL) cholesterol (mmol/L)

For peer review only - http://bmjopen.bmj.com/site/about/guidelines.xhtml

If there are any issues with partial dates in the database, the following approaches will be used to deal with them and therefore still allow derived time variables such as duration of diabetes to be calculated with sufficient precision:

If only year is available ("YYYY"), replace with "01/07/YYYY" or
If only month and year are available ("MM/YYYY"), replace with "15/MM/YYYY".

5 OUTLINE OF STATISTICAL ANALYSIS

Continuous variables will be summarised and presented by treatment group and overall as follows:

a) Mean and standard deviation (SD) for normal distribution
b) Median, Inter-Quartile Range (IQR), minimum and maximum for asymmetrical distribution

Categorical variables will be summarised and presented by treatment groups as the number of observations and proportion in each category and overall.

5.1 Outcome Measures

5.1.1 Primary Outcome

The primary outcome is glycaemic control defined as HbA1c, the primary endpoint refers to this data at 12-months but this will also be collected at baseline and 6-months (secondary endpoint). HbA1c is collected in mmol/mol but will be presented as a percentage. In order to convert between the two, the following calculation will be used [30]:

$$\text{HbA1c (\%)} = \text{HbA1c (mmol/mol)}/10.929 + 2.15$$

5.1.2 Secondary Outcomes - Biomedical

As an extension to the primary outcome, a secondary binary outcome to represent successful glycaemic control [9] will be calculated using the HbA1c data at 6- and 12-months. This outcome identified whether a participant achieved either a:

1) HbA1c ≤7.5% (58 mmol/mol) or
2) Reduction in HbA1c of ≥0.5% (≥5.5 mmol/mol).

Patient's BMI will be calculated using height and weight data at baseline, 6- and 12-months. Note – height is only collected once at baseline and used throughout. Weight can be collected either in kilograms (kg) or stones (st) and pounds (lb) and if collected in stone a conversion to kg will be completed using the following formula:

$$\text{Weight (kg)} = \text{Weight (st)}/0.15747 + \text{Weight (lb)}/2.2046$$

Similarly, height can be collected in metres (m) or feet (ft) and inches (in) and the following formula will be used to convert all to m:

$$\text{Height (m)} = \text{Height (ft)}/3.2808 + \text{Height (in)}/0.0254$$

BMI will then be calculated using the following formula:

$$\text{BMI (kg/m}^2\text{)} = \text{Weight (kg)} / \text{Height (m)}^2$$

Episodes of severe hypoglycaemia (as defined by the American Diabetes Association [9]), and incidence of DKA will be collected at baseline, 6-months and 12-months. Both will collect the number since the last visit.

For peer review only - http://bmjopen.bmj.com/site/about/guidelines.xhtml

Two different measures of lipids will be presented, high- and low-density lipoprotein which will be measured in mmol/L.

5.1.3 Secondary Outcomes - Psychological

- Audit of Diabetes-Dependent Quality of Life-15 (ADDQoL-15)

ADDQoL-15 is a questionnaire to measure the impact of diabetes and its treatment on a participant's QoL [10], [11]. It contains two overview items and 15 diabetes-specific items that relate to different aspects of life. Each has two parts, an impact score and an importance score. Each impact score is scored from -3 (very much better) to 1 (worse) and each importance score is from 3 (very important) to 0 (not at all important). These are then multiplied together to get a weighted impact score for each domain with -9 representing the maximum negative impact and 3 being the maximum positive impact. The average weighted impact (AWI) is then calculated as a mean of the weighted impacts for each domain.

If either the impact score or the importance score is missing, then the domain score cannot be computed and will not be included within the AWI. In the first instance, an AWI will only be calculated is all domain scores are available.

- DAWN Impact of Diabetes Profile (DIDP)

The participant's diabetes-specific quality of life will be assessed using the DIDP [12]. This consists of seven-items which investigates the impact diabetes has on different aspects of the participant's life. Each item is scored from 1 (very positive impact) to 7 (very negative impact), the composite score is the mean of all available responses, it ranges from 1-7 with lower scores indicating a greater positive impact. The percentage score is the composite score divided by 7, again lower percentages indicate greater positive impact.

- The Problem Areas in Diabetes

The Problem Areas in Diabetes (PAID) [13], [14] is a self-reported questionnaire that describe negative emotions related to diabetes (e.g. fear, anger, frustration) to assess diabetes distress. The short version, which will be used in this trial, consists of 11 items. Each question has five possible answers with a value from 0 to 4, with 0 representing "no problem" and 4 "a serious problem". The overall score is the sum of all questions and ranges from 0 (best) to 44 (worst). A score of 18+ indicates severe diabetes distress.

- Diabetes-specific Positive Well-being

Diabetes-Specific Positive Well-being will be measured using the specific subscale of the Well Being Questionnaire (W-BQ28) [15]. This questionnaire consist of four subscales each with four possible responses used to measure diabetes-specific well-being. Each item is scored from 0 (not at all) to 3 (all the time). The scores for this subscale are summed to get a total score ranging from 0-12 with higher scores representing more positive well-being.

- Fear of hypoglycaemia (HFS-II Short Form)

This 11-item questionnaire assesses the level of fear amongst people with diabetes [16] and contains five behavioural items and six worry items taken from the original full measure. Each item is scored

For peer review only - http://bmjopen.bmj.com/site/about/guidelines.xhtml

on a five-point Likert scale (0=never to 4=almost always) and a total score for the behaviour and worry subscales are calculated by summing the scores.
For missing values on the questionnaire, participant mean score will be imputed for missing values if at least nine of the questions have been completed.

- Health Status.

Health status will be measured using EQ-5D-5L [17] questionnaire which is a self-reported outcome measure which aims to assess the general health-related quality of life of the participant. It consists of five dimensions measure mobility, self-care, usual activity, pain/discomfort and anxiety/depression with each dimension having five possible responses. Participants also rate their overall health on the day of the interview on a 0–100 visual analogue scale with 0 = worst imaginable health state and 100 = best imaginable health state.

Scoring of EQ-5D-5L will be either scored using the relevant value set [18] or by mapping onto the EQ-5D-3L depending on the most up to date method at the time. The EQ-5D-5L health utility will not be calculated if any of the five dimensions are missing.

- Health and Self-Management in Diabetes

Health status will also be assessed using HASMID [19] assessment tool. It consists of ten items each with four possible responses. Responses are scored from zero to three with higher scores indicating little or no impact upon health related QoL. The overall questionnaire is a sum of all question scores and scored from 0 to 30 with higher scores indicating good health related QoL and a lower score indicating poor health-related quality of life. The scores are then used to calculate utility scores, the HASMiD health utility will not be calculated if any of the component ten questions are missing.

- Hypoglycaemia awareness

Awareness of hypoglycaemia will be measured using the Gold score questionnaire [20] which is a 1 item questionnaire consisting of a seven-point scale where one represent 'always aware of the onset of hypoglycaemia' and seven 'never aware of the onset of hypoglycaemia'.

5.1.4 Secondary Outcomes - Process Measures

- Diabetes Strengths and Resilience Questionnaire (DSRQ)

Adaptive behaviours and attitudes associated with overcoming challenges with diabetes management will be measured using the DSRQ [21]. The questionnaire consists of 12 items each scored from 1 (Never) to 5 (Always), the scores are summed to produce the total score which ranges from 12-60. Higher scores indicate perception of having greater T1D strengths.

- Self-Care Behaviours: Type 1 Diabetes (SCB-T1D)

Fifty items from the Self-Care Behaviours: Type 1 Diabetes (SCB-T1D) scale [22]will be used to assess the extent to which participants engaged with diabetes self-care behaviours. If an individual has completed over 50% of the items then the mean of the completed items will be used for the missing items. Otherwise the score will be coded as missing.

- System Usability Score (SUS)

For peer review only - http://bmjopen.bmj.com/site/about/guidelines.xhtml

The System Usability Score (SUS) [23] will be used to gather feedback on the DAFNEplus website at follow-up. As recommended by the scale authors the term 'system' will be replaced with 'Glucocollector' for the DAFNEplus group and 'bolus calculator' for the DAFNE (control) group. The SUS consists of 10 questions scored from 1 (strongly disagree) to 5 (strongly agree). Before calculating the total score the individual scores are transformed as follows:

- For each odd question (1,3,5,7 and 9) subtract 1
- For each even question (2,4,6,8 and 10) subtract from 5

The total score is the sum of these transformed values multiplied by 2.5, the score ranges from 1-100 with higher values indicating better usability.

- Diabetes Management Experience Questionnaire (DME-Q)

Satisfaction with diabetes treatment will be measured using the Diabetes Management Experience Questionnaire (DME-Q).

- Confidence in Diabetes Scale (CIDS)

The Confidence in Diabetes Scale (CIDS) [24] is a self-reported questionnaire with 20 items each scored from 1 (No, I am sure I cannot) to 5 (Yes, I am sure I can). The overall score is the sum of the items, minus the lowest possible score (20), divided by the score range (80) and multiplied by 100. This results in a 0-100 scale where higher scores indicate higher self-efficacy.

- Hypoglycaemia Confidence Scale (HCS)

The Hypoglycaemia Confidence Scale (HCS) [25] consists of 9 questions (8 items for participants without a partner) each rated from 1 (Not confident at all) to 4 (Very confident). The total score is calculated as the sum of the items divided by the number of items completed and ranges between 1-4 with higher scores indicating more confidence.

- Beliefs about Consequences

The Beliefs about Consequences questionnaire contains 6 items scored from 1 (not at all helpful) to 5 (extremely helpful), the items are summed to obtain the total score which ranges from 6-30.

- Self-Regulation Questionnaire (SRQ-T1D)

The SRQ-T1D questionnaire is an adaptation of the Self-Regulation Questionnaire [26], individual items are scored from 1 (strongly disagree) to 5 (strongly agree) and some items are reverse scaled (i.e. 1=5, 2=4, 3=3, 4=2, 5=1).

5.2 Analysis of Primary Outcome

The primary analysis will use the primary outcome of HbA1c at 12-months, using the primary ITT analysis population which is all consenting participants that have a baseline HbA1c > 7.5%. Descriptive statistics for baseline, 6- and 12-month HbA1c will be summarised and presented as mean, standard deviation (SD), median, min and max.

The treatment groups will be compared using a multiple linear regression model with coefficients estimated using GEE. The advantage of using GEE is that it is possible to calculate robust standard errors which are consistent even if the correlation structure is specified incorrectly [27], [28]. In this

For peer review only - http://bmjopen.bmj.com/site/about/guidelines.xhtml

model, an exchangeable correlation will be used to account for the clustering. In the event of any baseline differences in patient demographic characteristics, these covariates will be included in the model along with course. Adjustments for the stratification variables has not been included as they are at the centre level and therefore will be highly correlated with the course variable included in the model. The adjusted and unadjusted mean difference (MD) between DAFNE and DAFNE*plus* with associated 95% CI and p-value will be reported as well as the ICC from the model.

5.2.1 Model Checking

Given that correlation can lead to loss of information, ignoring the correlation structure can waste information and decrease standard errors when using an inappropriate analytical method. Model assumptions will be assessed graphically using the following methods:

- The linearity of the response variable will be assessed by a plot of the residuals against each explanatory variable in the model (curvilinear relationships). In cases of non-linearity, a transformation of the response variable could be performed, e.g log transformation or for particular fixed effect, a non-linear transformation of the particular fixed effect could be undertaken and included in the model,
- Constant variance will be assessed by plotting the residuals against the fitted values (errors have constant variance),
- Normality checks will be performed using a normal probability plot of the residuals (standardised) or histogram of the residuals,
- Partial residual plot will be used in identifying if quadratic or higher order terms are needed for any of the explanatory variables,
- Cook's distance can be used to indicate those observations that may be having an undue influence on the estimates. In cases of influential points, a sensitivity analyses with and without those points to assess the effects these points have on the regression coefficients will be undertaken.

5.3 Sensitivity Analyses of the Primary Outcome

Matching analyses will be undertaken on the primary outcome at 12-months using the full ITT and PP analysis sets as defined in table 4. These analyses will be completed using the primary analysis model with the only alternation the population set included within the analysis.

Additionally, a multi-level model will be completed using the primary ITT population on the primary outcome. This will contain the variables as mentioned in the original analysis but use course as a random effect to take into account the clustering in this model. These analyses will help to assess the robustness of the main trial result. The results in each case will be presented as adjusted MD of HbA1c at 12 months with associated 95% CI and p-value.

5.4 Subgroup Analyses

The following sub-group analysis will be completed on an ITT basis (Full ITT analysis population). The analysis will be the same model as the primary analysis with the addition of an interaction term between the treatment and subgroup to assess the stability of the result in different populations.

For peer review only - http://bmjopen.bmj.com/site/about/guidelines.xhtml

Treatment effect estimates with 95% confidence intervals will be calculated for each sub-group and a test for the overall interaction effect to reduce p-value use within each subgroup.

a) Previously attended a DAFNE course (Yes, No)
b) Use CGM or flash glucose monitoring between baseline and 12-month follow up (Yes, No)
c) Baseline HbA1c (≤7.5%, 7.5% < 8.5%, ≥8.5%)
d) Duration of diabetes (<15 years, ≥15 years)
e) Blood glucose level that symptoms of hypoglycaemia occur (do not feel symptoms, < 3mmol/l, ≥ 3mmol/l)
f) Self-reported use of the bolus advisor over the study duration (never or rarely, sometimes, often or always)
g) Age (≤34, 35-49, ≥50 years)
h) Sex (Male, Female)
i) BMI (Normal (<25), Overweight (25 < 30), Obese (≥30))
j) Socio-economic status (SES) as defined by the ONS Index of Multiple Deprivation (4 groups: above/below median in England, and above/below median in Scotland)
k) Total daily dose of insulin at baseline
l) Experience of lead course educator (Less experienced (6 courses or less within previous 3 years OR completed the DAFNE educator programme within previous year), More experienced (7+ courses within previous 3 years OR had continuous educator status for over 6 years))
m) Pregnant during the trial (Yes, No)
n) Type of basal insulin: (Human, Levemir, Lantus, Degludec, Toujeo)

5.5 Handling Missing Data

Missing observations can occur for numerous reasons (e.g. attrition) which can shrink the sample size, affects the precision of confidence intervals, reduce statistical power and biases parameter estimates [29]. Appropriately dealing with missing observations requires careful examination of data to identify the type and pattern of missingness.

In DAFNE*plus*, we anticipate that missing observations on the primary outcome (HbA1c) at 6 and 12months will occur amongst ITT participants. HbA1c results will be considered missing if the measure is outside +/- six weeks of the expected follow-up date. For the primary endpoint of HbA1c at 12 months, participant characteristics will be compared for those with and without the outcome. The aim of this is to explore any possible predictors of the missing outcome and evaluate the missing at random assumption. Multiple imputation strategies using a sequence of regression models [30] on the primary endpoint will be used where the missing values are filled ten times to generate ten complete data sets while utilising all variables that were included in the primary outcome analysis (section 5.4) as predictors. Any additional variables associated with the missing data will be included in the imputation model. This model will use a conservative approach by excluding treatment allocation.

If the data results in being missing not at random then a sensitivity analysis will be completed to assess the difference this makes on the results.

If weight is not recorded, other time points can be used as follows:

For peer review only - http://bmjopen.bmj.com/site/about/guidelines.xhtml

- Baseline weight missing - use 6-month weight,
- 6-month weight missing - if both baseline and 12-month weight are available, use the mean of these two. If only one of baseline or 12-month data are available, the recorded value will be used,
- All other circumstances - weight defined as missing.

5.6 Analysis of Secondary Outcomes - Biomedical

5.6.1 HbA1c

Additional secondary analyses on the primary outcome using the same model described in section 5.4 will be undertaken using HbA1c data collected at 6 months. This will be undertaken for all participants with HbA1C > 7.5% on an ITT basis. The adjusted and unadjusted mean difference (MD) between DAFNE and DAFNE*plus* with associated 95% CI and p-value will be reported as shown in table 7.11. Multiple imputations will be undertaken for missing HbA1c at 6 months using model described in 5.7.

The proportion of patients that have achieved improved glycaemic control will also be assessed. The patient is deemed to have improved glycaemic control if they achieved either: HbA1c ≤7.5% (58 mmol/mol) OR decrease in HbA1c ≥0.5% (≥5.5 mmol/mol). To test this, a GLM using a logit link function with treatment group, course and centre as random effects, and any baseline characteristics as used within the primary analysis model will be used (Logistic regression). Summary statistics for counts and percentages at 6 and 12 months follow up will be reported for DAFNE and DAFNE*plus* and overall. Treatment effect will be reported as both unadjusted or adjusted Odds Ratio (OR) with its associated 95% CI and p-value. This analysis will be undertaken using data collected at 6- and 12-month follow-up.

Additionally, it is important to assess the HbA1c trend over time.

5.6.2 Weight, BMI, Blood Pressure and Lipids

Summary statistics will be presented for each of these variables at baseline, and 12-months follow-up and all are treated as continuous variables. Summaries of weight and BMI at 6-months will be presented in the same way. Results will be presented by treatment group and overall and it will include the number, range and either the mean and SD or median and interquartile range depending on the distribution.

5.6.3 Severe Hypoglycaemia and DKA

The total number of episodes of hypoglycaemia will be treated as continuous and summary statistics of episodes since their last visit prior to the 12-month follow-up will be reported for DAFNE, DAFNE*plus* and overall.

To test for the difference between the two groups, the number of episodes of severe hypoglycaemia will be modelled using a negative binomial regression model with treatment group, baseline HbA1c and course (random effect). Treatment effect will be reported as Incidence Rate Ratios (IRR) with its associated 95% CI and p-value.

For peer review only - http://bmjopen.bmj.com/site/about/guidelines.xhtml

An additional analysis will be complete for the proportion of participants who experienced at least one episode of severe hypoglycaemia since their last visit prior to the 12 months follow-up. This will be modelled using a random effect logistic regression model. The model will include treatment group, baseline HbA1c and centre. Treatment effect will be reported and presented as Odds Ratio (OR) with its associated 95% CI and p-value.

5.6.4 Model Checking

Frequency graphs and the ratio of the variance to the mean will be used to assess the distribution of severe hypoglycaemia episodes. Failure to properly address existing over dispersion leads to serious underestimation of standard errors and misleading inference for the treatment effect. The Deviance and Likelihood Ratio Test (LRT) will be employed to assess goodness of fit of the Poisson linear regression model against two specific alternatives: a) a zero-inflated Poisson GLM (in the case of overdispersion due to excess zeros, or participants who experienced no episodes), and b) negative binomial regression for more general overdispersion. Further model diagnostics including measures of influence such as Cook's Distance will be undertaken for sensitivity analysis.

Unlike linear regression where graphical diagnostic displays can be very useful, for logistic regression models, the discreteness of binary data makes it difficult to interpret such displays. Three methods will be used for diagnostic checking of logistic regression models. Local mean deviance plots for detecting overall lack of fit, empirical probability plots to point out isolated departures from the fitted model and partial residual plots (smoothed) to identify specific causes of lack of fit.

5.7 Analysis of Secondary Outcomes - Psychological

All psychological outcomes will be analysed by the team at the University of Surrey, led by Debbie Cooke. The planned analysis will be defined by the team in a separate document and will be discussed with the statistical team to ensure consistency between methods. This will be signed off prior to data analysis.

5.8 Safety Outcomes

Adverse Events (AE) will be recorded throughout the trial and are defined as any unwanted medical occurrences which includes any episodes of diabetic ketoacidosis and any increase in frequency of severe hypoglycaemia. Serious Adverse Event (SAE) will also be recorded throughout the trial and are defined as AEs which result in hospitalisation or have a risk to life. A detailed description of AEs and SAEs can be found in the protocol.

Summary measures will be presented by treatment group as the number and percentage of participants reporting an AE/SAE as well as the total number of AE/SAEs reported and will be on an ITT basis. No formal statistical testing will be undertaken.

5.9 Statistical Software

This analysis will be carried out using any suitable packages such as R or STATA.

For peer review only - http://bmjopen.bmj.com/site/about/guidelines.xhtml

6 REFERENCES

6.1 Documents

DAFNE*plus* Protocol V2.0, 4th July 2018
Data Management Plan
ST001 The Statistical Analysis Plan
ST006 Undertaking a Statistical Analysis

6.2 Publications

[1] Diabetes UK, "Diabetes UK: Facts and Stats, October 2016," 2016. .

[2] The Diabetes Control and Complications Trial Research Group, "The effect of intensive treatment of diabetes on the development and progression of long-term complications in insulin-dependent diabetes mellitus," *N. Engl. J. Med.*, 1993.

[3] S. Heller, "Hypoglycaemia and diabetes," in *Textbook of Diabetes*, G. Pickup, J, WIlliams, Ed. Oxford: Blackwell Science, 2003, pp. 33.1-33.19.

[4] M. J. Campbell and S. J. Walters, *How to Design, Analyse and Report Cluster Randomised Trials in Medicine and Health Related Research*. Chichester, UK: John Wiley & Sons, Ltd, 2014.

[5] F. Li, Y. Lokhnygina, D. M. Murray, P. J. Heagerty, and E. R. DeLong, "An evaluation of constrained randomization for the design and analysis of group-randomized trials," *Stat. Methods Med. Res.*, vol. 35, no. 10, pp. 1565–1579, 2016.

[6] StataCorp, "Stata Statistical Software: Release 15," *2017*. 2017.

[7] ICH, "ICH Harmonised Tripartite Guideline. Statistical Principles for Clinical Trials. E9.," 1998. [Online]. Available: http://www.ich.org/fileadmin/Public_Web_Site/ICH_Products/Guidelines/Efficacy/E9/Step4/E9_Guideline.pdf.

[8] K. F. Schulz, D. G. Altman, D. Moher, and CONSORT Group, "CONSORT 2010 statement: updated guidelines for reporting parallel group randomised trials.," *BMJ*, vol. 340, no. mar23 1, p. c332, 2010.

[9] International Hypoglycaemia Study Group, "Glucose Concentrations of Less Than 3.0 mmol/L (54 mg/dL) Should Be Reported in Clinical Trials: A Joint Position Statement of the American Diabetes Association and the European Association for the Study of Diabetes: Table 1," *Diabetes Care*, vol. 40, no. 1, pp. 155–157, Jan. 2017.

[10] C. Bradley, C. Todd, T. Gorton, E. Symonds, A. Martin, and R. Plowright, "The development of an individualized questionnaire measure of perceived impact of diabetes on quality of life: the ADDQoL," *Qual. Life Res.*, vol. 8, no. 1/2, pp. 79–91, 1999.

[11] M. C. Rosal, B. Olendzki, G. W. Reed, O. Gumieniak, J. Scavron, and I. Ockene, "Diabetes self-management among low-income Spanish-speaking patients: A pilot study," *Ann. Behav. Med.*, vol. 29, no. 3, pp. 225–235, 2005.

[12] M. Peyrot, R. R. Rubin, T. Lauritzen, F. J. Snoek, D. R. Matthews, and S. E. Skovlund, "Psychosocial problems and barriers to improved diabetes management: results of the Cross-National Diabetes Attitudes, Wishes and Needs (DAWN) Study," *Diabet. Med.*, vol. 22, no. 10, pp. 1379–1385, Oct. 2005.

[13] W. H. Polonsky *et al.*, "Assessment of diabetes-related distress," *Diabetes Care*, 1995.

[14] D. A. W. and N. (DAWN), "Problem Area In Diabetes Questionnaire (PAID)," 1999. [Online]. Available: http://www.dawnstudy.com/content/dam/Dawnstudy/AFFILIATE/www-dawnstudy-com/Home/TOOLSANDRESOURCES/Documents/PAID_problem_areas_in_diabetes_questionnaire.pdf.

For peer review only - http://bmjopen.bmj.com/site/about/guidelines.xhtml

[15] C. Speight, J, Barendse, S, Bradley, "The W-BQ28: further development of the well-being Questionnaire to include diabetes-specific as well as generic subscales and new stress subscales," in *British Psychological Society, Division of Health Psychology*, 1999.
[16] J. Grabman *et al.*, "An empirically derived short form of the Hypoglycaemia Fear Survey II," *Diabet. Med.*, vol. 34, no. 4, pp. 500–504, Apr. 2017.
[17] M. Herdman *et al.*, "Development and preliminary testing of the new five-level version of EQ-5D (EQ-5D-5L)," *Qual. Life Res.*, vol. 20, no. 10, pp. 1727–1736, Dec. 2011.
[18] N. J. Devlin, K. K. Shah, Y. Feng, B. Mulhern, and B. van Hout, "Valuing health-related quality of life: An EQ-5D-5L value set for England," *Health Econ.*, Aug. 2017.
[19] J. Carlton *et al.*, "Developing a questionnaire to determine the impact of self-management in diabetes: giving people with diabetes a voice," *Health Qual. Life Outcomes*, vol. 15, no. 1, p. 146, Dec. 2017.
[20] A. E. Gold, K. M. MacLeod, and B. M. Frier, "Frequency of severe hypoglycemia in patients with type I diabetes with impaired awareness of hypoglycemia.," *Diabetes Care*, vol. 17, no. 7, pp. 697–703, Jul. 1994.
[21] M. E. Hilliard, E. Iturralde, J. Weissberg-Benchell, and K. K. Hood, "The Diabetes Strengths and Resilience Measure for Adolescents With Type 1 Diabetes (DSTAR-Teen): Validation of a New, Brief Self-Report Measure," *J. Pediatr. Psychol.*, vol. 42, no. 9, pp. 995–1005, Oct. 2017.
[22] C. D. Mansell P, Grant L, Bond R, Pouwer F, Page N, Davies A, Stenner K, Elliott J, Hopkins D, Jones A, Vallis M, Speight J, Lawton J, Clark M, Heller S, "Assessment of Self-Care Behaviours focused on Glycemic Control in Type 1 Diabetes: Development and Validation of the SCB-T1D Questionnaire." .
[23] J. Brooke, "SUS-A quick and dirty usability scale," *Usability Eval. Ind.*, vol. 189, no. 194, pp. 4–7, 1996.
[24] N. C. W. Van Der Ven *et al.*, "The confidence in diabetes self-care scale: Psychometric properties of a new measure of diabetes-specific self-efficacy in Dutch and U.S. patients with type 1 diabetes," *Diabetes Care*, vol. 26, no. 3, pp. 713–718, Mar. 2003.
[25] W. H. Polonsky, L. Fisher, D. Hessler, and S. V. Edelman, "Investigating Hypoglycemic Confidence in Type 1 and Type 2 Diabetes," *Diabetes Technol. Ther.*, vol. 19, no. 2, pp. 131–136, Feb. 2017.
[26] J. M. Brown, W. R. Miller, and L. A. Lawendowski, "The Self-Regulation Questionnaire.," in *Vandecreek, L. and Jackson, T.L., Eds., Innovations in Clinical Practice: A Sourcebook*, Vol. 17., Professional Resource Press/Professional Resource Exchange, Sarasota, FL, 1999, pp. 281–292.
[27] K.-Y. Liang and S. L. Zeger, "Longitudinal Data Analysis Using Generalized Linear Models," *Biometrika*, 1986.
[28] E. Demidenko and T. A. Stukel, "Influence analysis for linear mixed-effects models," *Stat. Med.*, 2005.
[29] P. D. Allison, "Missing Data," *Quant. Appl. Soc. Sci.*, 2001.
[30] T. E. Raghunathan, J. M. Lepkowski, J. Van Hoewyk, and P. Solenberger, "A multivariate technique for multiply imputing missing values using a sequence of regression models," *Surv. Methodol.*, 2001.

For peer review only

For peer review only - http://bmjopen.bmj.com/site/about/guidelines.xhtml

SPIRIT

STANDARD PROTOCOL ITEMS: RECOMMENDATIONS FOR INTERVENTIONAL TRIALS

SPIRIT 2013 checklist: Recommended items to address in a clinical trial protocol and related documents

Completed for DAFNE*plus* protocol submitted to BMJ Open

Section/item	ItemNo	Description	Included? (page number)
**Administrative information**			
Title	1	Descriptive title identifying the study design, population, interventions, and, if applicable, trial acronym	Yes<br>All elements included (p1)
Trial registration	2a	Trial identifier and registry name. If not yet registered, name of intended registry	Yes<br>Registered with ISRCTN42908016 (p3)
	2b	All items from the World Health Organization Trial Registration Data Set	Yes<br>See supplementary material 1
Protocol version	3	Date and version identifier	Yes (p2)
Funding	4	Sources and types of financial, material, and other support	Yes<br>Funded by the NIHR (p4)
Roles and responsibilities	5a	Names, affiliations, and roles of protocol contributors	Yes<br>Protocol contributors to be listed under Corporate 'DAFNEplus Group' Authorship are provided (p1)
	5b	Name and contact information for the trial sponsor	Yes<br>Sponsored by STH NHS FT (p4)
	5c	Role of study sponsor and funders, if any, in study design; collection, management, analysis, and interpretation of data; writing of the report; and the decision to submit the report for publication, including whether they will have ultimate authority over any of these activities	Yes (p4)

For peer review only

	5d	Composition, roles, and responsibilities of the coordinating centre, steering committee, endpoint adjudication committee, data management team, and other individuals or groups overseeing the trial, if applicable (see Item 21a for data monitoring committee)	Yes<br>Membership of trial co-ordinating centre, Trial Management Group and Trial Steering Committee listed (p5)
**Introduction**			
Background and rationale	6a	Description of research question and justification for undertaking the trial, including summary of relevant studies (published and unpublished) examining benefits and harms for each intervention	Yes<br>See ‘Background and rationale’ section (pp6-7)
	6b	Explanation for choice of comparators	Yes<br>See ‘Standard DAFNE (control arm)’ section, paragraph 3 (pp9-10)
Objectives	7	Specific objectives or hypotheses	Yes<br>See ‘Aims and objectives’ section (p7)
Trial design	8	Description of trial design including type of trial (eg, parallel group, crossover, factorial, single group), allocation ratio, and framework (eg, superiority, equivalence, noninferiority, exploratory)	Yes<br>See ‘Trial design’ section (p7-8)
**Methods: Participants, interventions, and outcomes**			
Study setting	9	Description of study settings (eg, community clinic, academic hospital) and list of countries where data will be collected. Reference to where list of study sites can be obtained	Yes<br>See ‘Study setting’ section (p8)
Eligibility criteria	10	Inclusion and exclusion criteria for participants. If applicable, eligibility criteria for study centres and individuals who will perform the interventions (eg, surgeons, psychotherapists)	Yes<br>See ‘Eligibility criteria’ section (p8)
Interventions	11a	Interventions for each group with sufficient detail to allow replication, including how and when they will be administered	Yes<br>See ‘Interventions’ section (pp9-11)

For peer review only

	11b	Criteria for discontinuing or modifying allocated interventions for a given trial participant (eg, drug dose change in response to harms, participant request, or improving/worsening disease)	Yes<br>See 'Criteria for withdrawal' section (p12)
	11c	Strategies to improve adherence to intervention protocols, and any procedures for monitoring adherence (eg, drug tablet return, laboratory tests)	Yes<br>Supervision of interventionists (p11) and fidelity assessment (pp14)
	11d	Relevant concomitant care and interventions that are permitted or prohibited during the trial	No<br>No restrictions on concomitant care other than use of CSII pump therapy (exclusion criteria 1)
Outcomes	12	Primary, secondary, and other outcomes, including the specific measurement variable (eg, systolic blood pressure), analysis metric (eg, change from baseline, final value, time to event), method of aggregation (eg, median, proportion), and time point for each outcome. Explanation of the clinical relevance of chosen efficacy and harm outcomes is strongly recommended	Yes<br>Outcomes listed in table 1 (pp16-17) and described in 'Outcomes' section (pp12-15)
Participant timeline	13	Time schedule of enrolment, interventions (including any run-ins and washouts), assessments, and visits for participants. A schematic diagram is highly recommended (see Figure)	Yes<br>Participant timeline shown in figure 1
Sample size	14	Estimated number of participants needed to achieve study objectives and how it was determined, including clinical and statistical assumptions supporting any sample size calculations	Yes<br>See 'sample size' section (p15)
Recruitment	15	Strategies for achieving adequate participant enrolment to reach target sample size	Yes<br>See 'recruitment' section (p9)
**Methods: Assignment of interventions (for controlled trials)**			
Allocation:			

For peer review only

Sequence generation	16a	Method of generating the allocation sequence (eg, computer-generated random numbers), and list of any factors for stratification. To reduce predictability of a random sequence, details of any planned restriction (eg, blocking) should be provided in a separate document that is unavailable to those who enrol participants or assign interventions	Yes<br>See 'random allocation' section (p12)
Allocation concealment mechanism	16b	Mechanism of implementing the allocation sequence (eg, central telephone; sequentially numbered, opaque, sealed envelopes), describing any steps to conceal the sequence until interventions are assigned	N/A – cluster RCT whereby trial centres randomised post ethical approval (p12)
Implementation	16c	Who will generate the allocation sequence, who will enrol participants, and who will assign participants to interventions	Yes<br>See 'random allocation' (p12)
Blinding (masking)	17a	Who will be blinded after assignment to interventions (eg, trial participants, care providers, outcome assessors, data analysts), and how	Yes<br>Blinding not possible during this trial – see 'blinding' section (p12)
	17b	If blinded, circumstances under which unblinding is permissible, and procedure for revealing a participant's allocated intervention during the trial	N/A
**Methods: Data collection, management, and analysis**			
Data collection methods	18a	Plans for assessment and collection of outcome, baseline, and other trial data, including any related processes to promote data quality (eg, duplicate measurements, training of assessors) and a description of study instruments (eg, questionnaires, laboratory tests) along with their reliability and validity, if known. Reference to where data collection forms can be found, if not in the protocol	Yes<br>Collection of outcome data described in 'Outcomes' section (pp12-15) and paragraph 1 of 'Data collection and management' (pp18-19)
	18b	Plans to promote participant retention and complete follow-up, including list of any outcome data to be collected for participants who discontinue or deviate from intervention protocols	Yes<br>See 'Data collection and management' section (pp18-19)

For peer review only

Data management	19	Plans for data entry, coding, security, and storage, including any related processes to promote data quality (eg, double data entry; range checks for data values). Reference to where details of data management procedures can be found, if not in the protocol	Yes<br>See ‘Data collection and management’ section (p18-19)
Statistical methods	20a	Statistical methods for analysing primary and secondary outcomes. Reference to where other details of the statistical analysis plan can be found, if not in the protocol	Yes<br>See ‘Statistical analysis’ section (p22) and SAP is provided in supplementary material
	20b	Methods for any additional analyses (eg, subgroup and adjusted analyses)	Yes<br>See ‘Statistical analysis’ section (p18) and SAP is provided in supplementary material
	20c	Definition of analysis population relating to protocol non-adherence (eg, as randomised analysis), and any statistical methods to handle missing data (eg, multiple imputation)	Yes<br>See ‘Statistical analysis’ section (p18) and SAP is provided in supplementary material
**Methods: Monitoring**			
Data monitoring	21a	Composition of data monitoring committee (DMC); summary of its role and reporting structure; statement of whether it is independent from the sponsor and competing interests; and reference to where further details about its charter can be found, if not in the protocol. Alternatively, an explanation of why a DMC is not needed	N/A<br>As described in ‘Trial oversight committees’, a DMEC has not been convened for this trial on the grounds that this is low risk. This has been approved by both Sponsor and TSC.
	21b	Description of any interim analyses and stopping guidelines, including who will have access to these interim results and make the final decision to terminate the trial	N/A
Harms	22	Plans for collecting, assessing, reporting, and managing solicited and spontaneously reported adverse events and other unintended effects of trial interventions or trial conduct	Yes<br>See ‘Safety outcomes’ (p15)

For peer review only

Auditing	23	Frequency and procedures for auditing trial conduct, if any, and whether the process will be independent from investigators and the sponsor	Yes<br>See final paragraph of 'Data collection and management' (p19) section for summary of plans for monitoring.
**Ethics and dissemination**			
Research ethics approval	24	Plans for seeking research ethics committee/institutional review board (REC/IRB) approval	Yes<br>See 'Research ethics approval section' (p19)
Protocol amendments	25	Plans for communicating important protocol modifications (eg, changes to eligibility criteria, outcomes, analyses) to relevant parties (eg, investigators, REC/IRBs, trial participants, trial registries, journals, regulators)	No<br>This has not been included due to word count restrictions.
Consent or assent	26a	Who will obtain informed consent or assent from potential trial participants or authorised surrogates, and how (see Item 32)	Yes<br>See paragraph 3 of 'Recruitment' section (p9)
	26b	Additional consent provisions for collection and use of participant data and biological specimens in ancillary studies, if applicable	N/A
Confidentiality	27	How personal information about potential and enrolled participants will be collected, shared, and maintained in order to protect confidentiality before, during, and after the trial	Yes<br>See 'Data collection and management' (pp18-19)
Declaration of interests	28	Financial and other competing interests for principal investigators for the overall trial and each study site	Yes<br>See declarations of interest submitted to journal
Access to data	29	Statement of who will have access to the final trial dataset, and disclosure of contractual agreements that limit such access for investigators	No but documented in Data Management Plan for trial
Ancillary and post-trial care	30	Provisions, if any, for ancillary and post-trial care, and for compensation to those who suffer harm from trial participation	No but provisions addressed in sponsor contracts
Dissemination policy	31a	Plans for investigators and sponsor to communicate trial results to participants, healthcare professionals, the	Yes

For peer review only

		public, and other relevant groups (eg, via publication, reporting in results databases, or other data sharing arrangements), including any publication restrictions	See 'Dissemination Policy' section (p19)
	31b	Authorship eligibility guidelines and any intended use of professional writers	No<br>This is not documented in protocol due to word count restrictions but addressed in dissemination strategy for trial. ICJME criteria used to assess authorship eligibility.
	31c	Plans, if any, for granting public access to the full protocol, participant-level dataset, and statistical code	No but documented in Data Management Plan for trial
**Appendices**			
Informed consent materials	32	Model consent form and other related documentation given to participants and authorised surrogates	No<br>Available if required for supplementary
Biological specimens	33	Plans for collection, laboratory evaluation, and storage of biological specimens for genetic or molecular analysis in the current trial and for future use in ancillary studies, if applicable	N/A

For peer review only

BMJ Open

A protocol for a cluster randomised controlled trial of the DAFNEplus (Dose Adjustment for Normal Eating) intervention compared with 5x1 DAFNE: A lifelong approach to promote effective self-management in adults with type 1 diabetes

Journal:	*BMJ Open*
Manuscript ID	bmjopen-2020-040438.R1
Article Type:	Protocol
Date Submitted by the Author:	23-Nov-2020

Complete List of Authors:	Coates, Elizabeth; The University of Sheffield School of Health and Related Research,<br>Amiel, Stephanie; Kings College London School of Medicine, Diabetes, Endocrinology and Internal Medicine<br>Baird, Wendy; University of Sheffield, ScHARR<br>Benaissa, Mohammed; University of Sheffield, Department of Electronic and Electrical Engineering<br>Brennan, Alan; University of Sheffield, School of Health & Related Research<br>Campbell, Michael; University of Sheffield, Health Services Research ScHARR<br>Chadwick, Paul; UCL, Epidemiology and Public Health<br>Chater, Tim; The University of Sheffield, School of Health and Related Research<br>Choudhary, Pratik; King's College London Faculty of Life Sciences and Medicine, Department of Diabetes<br>Cooke, Debbie; University of Surrey,<br>Cooper, Cindy; University of Sheffield, School of Health and Related Research<br>Cross, Elizabeth; University of Sheffield, ScHARR<br>De Zoysa, Nicole; King's College Hospital<br>Eissa, Mohammad; University of Sheffield, Department of Electronic and Electrical Engineering<br>Elliott, Jackie; University of Sheffield, Academic Unit of Diabetes, Endocrinology and Metabolism<br>Gianfrancesco, Carla; Sheffield Teaching Hospitals NHS Foundation Trust, Diabetes Centre<br>Good, Tim; University of Sheffield, Department of Electronic and Electrical Engineering<br>Hopkins, David; King's College London, General and Emergency Medicine<br>Hui, Zheng; University of Sheffield, Department of Electronic and Electrical Engineering<br>Lawton, Julia; University of Edinburgh, Centre for Population Health Sciences<br>Lorencatto, Fabiana; UCL,<br>Michie, Susan; University College London, Centre for Outcomes Research and Effectivenes

Pollard, Daniel ; University of Sheffield, School of Health and Related Research
Rankin, David; University of Edinburgh, Centre for Population Health Sciences
Schutter, Jose; University of Sheffield, ScHARR
Scott, Elaine; University of Sheffield, ScHARR
Speight, Jane; The Australian Centre for Behavioural Research in Diabetes,
Stanton-Fay, Stephanie; UCL, Epidemiology and Public Health
Taylor, Carolin; Sheffield Teaching Hospitals NHS Foundation Trust, Diabetes Centre
Thompson, Gillian; Northumbria Healthcare NHS Foundation Trust
Totton, Nikki; University of Sheffield, School of Health and Related Research
Yardley, Lucy; University of Southampton, Academic Unit of Psychology
Zaitcev, Aleksandr; University of Sheffield, Department of Electronic and Electrical Engineering
Heller, Simon; The University of Sheffield Department of Oncology and Metabolism,

**Primary Subject Heading**:	Diabetes and endocrinology
Secondary Subject Heading:	Health services research, Health economics, Qualitative research
Keywords:	DIABETES & ENDOCRINOLOGY, EDUCATION & TRAINING (see Medical Education & Training), HEALTH ECONOMICS, QUALITATIVE RESEARCH

SCHOLARONE™
Manuscripts

I, the Submitting Author has the right to grant and does grant on behalf of all authors of the Work (as defined in the below author licence), an exclusive licence and/or a non-exclusive licence for contributions from authors who are: i) UK Crown employees; ii) where BMJ has agreed a CC-BY licence shall apply, and/or iii) in accordance with the terms applicable for US Federal Government officers or employees acting as part of their official duties; on a worldwide, perpetual, irrevocable, royalty-free basis to BMJ Publishing Group Ltd ("BMJ") its licensees and where the relevant Journal is co-owned by BMJ to the co-owners of the Journal, to publish the Work in this journal and any other BMJ products and to exploit all rights, as set out in our licence.

The Submitting Author accepts and understands that any supply made under these terms is made by BMJ to the Submitting Author unless you are acting as an employee on behalf of your employer or a postgraduate student of an affiliated institution which is paying any applicable article publishing charge ("APC") for Open Access articles. Where the Submitting Author wishes to make the Work available on an Open Access basis (and intends to pay the relevant APC), the terms of reuse of such Open Access shall be governed by a Creative Commons licence – details of these licences and which Creative Commons licence will apply to this Work are set out in our licence referred to above.

Other than as permitted in any relevant BMJ Author's Self Archiving Policies, I confirm this Work has not been accepted for publication elsewhere, is not being considered for publication elsewhere and does not duplicate material already published. I confirm all authors consent to publication of this Work and authorise the granting of this licence.

For peer review only

Title: A protocol for a cluster randomised controlled trial of the DAFNE*plus* (Dose Adjustment for Normal Eating) intervention compared with 5x1 DAFNE: A lifelong approach to promote effective self-management in adults with type 1 diabetes

Authors: The DAFNE*plus* group (to be listed as)

Elizabeth Coates	University of Sheffield	e.coates@sheffield.ac.uk
Stephanie Amiel	King's College London	stephanie.amiel@kcl.ac.uk
Wendy Baird	University of Sheffield	w.o.baird@sheffield.ac.uk
Mohammed Benaissa	University of Sheffield	m.benaissa@sheffield.ac.uk
Alan Brennan	University of Sheffield	a.brennan@sheffield.ac.uk
Michael Joseph Campbell	University of Sheffield	m.j.campbell@sheffield.ac.uk
Paul Chadwick	University College London	p.chadwick@ucl.ac.uk
Tim Chater	University of Sheffield	t.chater@sheffield.ac.uk
Pratik Choudhary	King's College London	pratik.choudhary@kcl.ac.uk
Debbie Cooke	University of Surrey	d.cooke@surrey.ac.uk
Cindy Cooper	University of Sheffield	c.l.cooper@sheffield.ac.uk
Elizabeth Cross	University of Sheffield	e.a.cross@sheffield.ac.uk
Nicole De Zoysa	King's College Hospital	ndezoysa@nhs.net
Mohammad Eissa	University of Sheffield	m.eissa@sheffield.ac.uk
Jackie Elliott	Sheffield Teaching Hospitals NHS Foundation Trust	j.elliott@sheffield.ac.uk
Carla Gianfrancesco	Sheffield Teaching Hospitals NHS Foundation Trust	carla.gianfrancesco@nhs.net
Tim Good	University of Sheffield	tim.good@sheffield.ac.uk
David Hopkins	King's College Hospital	dhopkins3@nhs.net
Zheng Hui	University of Sheffield	zhui2@sheffield.ac.uk
Julia Lawton	University of Edinburgh	j.lawton@ed.ac.uk
Fabiana Lorencatto	University College London	f.lorencatto@ucl.ac.uk
Susan Michie	University College London	s.michie@ucl.ac.uk
Daniel John Pollard	University of Sheffield	d.j.pollard@sheffield.ac.uk
David Rankin	University of Edinburgh	a.d.rankin@ed.ac.uk
Jose Schutter	University of Sheffield	j.schutter@sheffield.ac.uk
Elaine Scott	University of Sheffield	elaine.scott@sheffield.ac.uk
Jane Speight	The Australian Centre for Behavioural Research in Diabetes: Deakin University & Diabetes Victoria	jspeight@acbrd.org.au
Stephanie Stanton-Fay	University College London	stephanie.stanton-fay@ucl.ac.uk
Carolin Taylor	Sheffield Teaching Hospitals NHS Foundation Trust	carolin.taylor@nhs.net
Gillian Thompson	Northumbria Healthcare NHS	gillian.thompson@northumbria-healthcare.nhs.uk

For peer review only - http://bmjopen.bmj.com/site/about/guidelines.xhtml

Nikki Totton	University of Sheffield	n.v.totton@sheffield.ac.uk
Lucy Yardley	University of Southampton	l.yardley@soton.ac.uk
Aleksandr Zaitcev	University of Sheffield	a.zaitcev@sheffield.ac.uk
Simon Heller*	University of Sheffield	s.heller@sheffield.ac.uk

* Corresponding author:
Professor Simon Heller, Professor of Clinical Diabetes, Department of Oncology and Metabolism, University of Sheffield, Beech Hill Road, Sheffield S10 2RX, Tel: 0114 215 9009, s.heller@sheffield.ac.uk

Version 6.0, 09.09.2019

Word count: 4804

For peer review only

Abstract

Introduction: The successful treatment of type 1 diabetes (T1D) requires those affected to employ insulin therapy to maintain their blood glucose levels as close to normal to avoid complications in the long-term. The Dose Adjustment for Normal Eating (DAFNE) intervention is a group education course designed to help adults with T1D develop and sustain the complex self-management skills needed to adjust insulin in everyday life. It leads to improved glucose levels in the short-term (manifest by falls in HbA1c), reduced rates of hypoglycaemia and sustained improvements in quality of life but overall glucose levels remain well above national targets. The DAFNE*plus* intervention is a development of DAFNE designed to incorporate behaviour change techniques, technology and longer-term structured support from healthcare professionals.

Methods and analysis: A pragmatic cluster randomised controlled trial in adults with T1D, delivered in diabetes centres in NHS secondary care hospitals in the United Kingdom. Centres will be randomised on a 1:1 basis to standard DAFNE or DAFNE*plus.*

Primary clinical outcome is the change in HbA1c (glycated haemoglobin) and the primary endpoint is HbA1c at 12 months, in those entering the trial with HbA1c >7.5% (58 mmol/mol), and HbA1c at 6 months is the secondary endpoint. Sample size is 662 participants (approximately 47 per centre); 92% power to detect a 0.5% difference in the primary outcome of HbA1c between treatment groups. The trial also measures rates of hypoglycaemia, psychological outcomes, an economic evaluation and process evaluation.

Ethics and dissemination: Ethics approval was granted by South West – Exeter Research Ethics Committee (REC ref: 18/SW/0100) on 14th May 2018. The results of the trial will be published in a National Institute for Health Research monograph and relevant high-impact journals.

Trial registration: ISRCTN42908016 – registered on 17th May 2018

Article summary
Strengths and limitations of this study

- Comparison of group therapy against another group therapy will standardise the treatment comparison.
- Cluster randomisation to avoid contamination of the intervention material.
- Number of sites in both England and Scotland representing a wide range of NHS Trusts.
- Use of a covariate constrained methodology to randomise means that sites are matched which can create issues if sites drop out.
- Blinding not possible in trial due to the intervention and design.

Keywords
Diabetes mellitus, Type 1; Self management; Patient education; Randomised controlled trial;

For peer review only

Sponsor: Sheffield Teaching Hospitals NHS Foundation Trust
Contact Name Dr Erica Wallis
Clinical Research Office
Sheffield Teaching Hospitals NHS Foundation Trust
Glossop Road
Sheffield S10 2JF
Phone: 0114 2265931
Email: erica.wallis1@nhs.net

Role of study sponsor and funder

Neither the funder nor the sponsor has had any role in study design, data collection or analysis, decision to publish, or preparation of manuscripts.

For peer review only

Chief Investigator: Professor Simon Heller, Professor of Clinical Diabetes, Sheffield Teaching Hospitals NHS Foundation Trust

Trial Co-ordinating Centre: Sheffield CTRU, University of Sheffield
Mrs Elaine Scott, Trial Manager
Mrs Liz Cross, Senior Trial Manager
Miss Jose Schutter, Research Assistant
Mr Tim Chater, Data Manager
Miss Becky Brown, Trials Support Officer
Professor Cindy Cooper, Trials Oversight

Trial Management Group:
Professor Stephanie Amiel, King's College London
Professor Wendy Baird, University of Sheffield
Dr Mohammed Benaissa, University of Sheffield
Professor Alan Brennan, University of Sheffield
Miss Becky Brown, University of Sheffield
Dr Paul Chadwick, University College London
Mr Tim Chater, University of Sheffield
Dr Pratik Choudhary, King's College London
Dr Debbie Cooke, University of Surrey
Professor Cindy Cooper, University of Sheffield
Dr Mohammad Eissa, University of Sheffield
Dr Tim Good, University of Sheffield
Ms Carla Gianfrancesco, Sheffield Teaching Hospitals NHS Foundation Trust
Dr Jackie Elliott, University of Sheffield
Dr David Hopkins, King's College Hospital NHS Foundation Trust
Professor Julia Lawton, University of Edinburgh
Dr Fabiana Lorencatto, University College London
Professor Susan Michie, University College London
Mr Dan Pollard, University of Sheffield
Dr David Rankin, University of Edinburgh
Miss Jose Schutter, University of Sheffield
Mrs Elaine Scott, University of Sheffield
Professor Jane Speight, Deakin University, Australia
Dr Stephanie Stanton-Fay, University College London
Mrs Carolin Taylor, Sheffield Teaching Hospitals NHS Foundation Trust
Mrs Gill Thompson, Northumbria Healthcare NHS Foundation Trust
Ms Nikki Totton, University of Sheffield
Mr Chris Turtle, University of Sheffield
Dr Nicole de Zoysa, King's College Hospital NHS Foundation Trust

Trial Steering Committee membership:
Chair: Professor Simon Griffin, University of Cambridge;
Independent Statistician: Professor Catherine Hewitt, University of York;
Independent Clinical Expert: Professor James Shaw, University of Newcastle
Patient Representative: Mr Arthur Durrant, Ms Lynne Dawson
Sponsor's Representative: Dr Erica Wallis, Sheffield Teaching Hospitals NHS Foundation Trust
Funder's Representative: Mr Ramnath Elaswarapu, National Institute for Health Research

For peer review only

Introduction

Background and Rationale

Type 1 diabetes (T1D) is characterised by absolute insulin deficiency, requiring insulin to be injected subcutaneously several times a day. Successful management requires those affected (>300,000 adults in the UK)[1] to keep their blood glucose levels sufficiently close to recommended targets to avoid long-term complications including blindness, renal failure, amputations and premature death[2]. In addition, exogenous insulin therapy can prevent high blood glucose and acute, life threatening emergencies such as diabetic ketoacidosis, as well as being a tool to prevent long-term complications.

Achieving the blood glucose control to help prevent complications depends upon an individual's ability to self-manage their condition, calculating precise insulin doses based on accurate estimations of food intake before every meal, frequent blood glucose measurements, and account for fluctuations in physical activity, illness and hormones. If people with T1D are unable or unwilling to calculate and administer their insulin doses correctly, their blood glucose either runs high, increasing the risks of complications, or else falls too low leading to hypoglycaemia. Hypoglycaemia, if severe, can result in acute cognitive impairment, confusion, collapse and injury, coma or even death[3]. Thus, people with T1D must acquire complex self-management knowledge and skills, and have the motivation and ability to apply them effectively every day. The responsibility of diabetes healthcare professionals (HCPs) is to ensure that all people with T1D have the opportunity to acquire these skills and are supported in applying them successfully in everyday life.

'Dose Adjustment for Normal Eating' (DAFNE) is a structured education programme run within the National Health Service (NHS), designed to enable adults with T1D to learn or enhance their self-management skills in flexible intensive insulin therapy to improve both glucose control and quality of life. It is a five-day training course, delivered in small groups. DAFNE has been delivered to over 51,000 adults in the UK[4]. The publication of the UK DAFNE randomised controlled trial (RCT) in 2002[5] established the ability of structured education courses to enable people with diabetes to acquire the knowledge and skills to live successfully with this lifelong condition. The subsequent rollout of DAFNE across the UK has enabled many individuals to meet these demands and achieve their goals, but over half of DAFNE graduates still struggle to manage glucose levels consistently. After attending a DAFNE course, people have better quality of life, better control of blood glucose levels and are admitted to hospital less often for diabetes emergencies[6]. Many DAFNE graduates find the course helpful; quality of life improves and rates of severe hypoglycaemia fall. However although HbA1c (glycated haemoglobin) falls and in one trial, this improvement was sustained for 2 years, average HbA1c, the intermediate measure of glucose control that best predicts risk of diabetes complications, remains well above recommended UK targets[7,8]. Many, find it difficult to implement and sustain the skills needed to maintain blood glucose levels and often struggle to obtain suitable support from HCPs[6,9–15].

For peer review only

The DAFNE*plus* intervention has been developed through modifying the existing DAFNE programme by incorporating techniques for initiating and sustaining behaviour change, and supplementing this with structured follow-up support and enhanced information technology. The aim of this trial is to investigate whether the DAFNE*plus* programme will produce improved and sustainable diabetes self-management behaviour and better glucose outcomes than currently achieved with standard DAFNE, without compromising quality of life in the longer term.

Aims and objectives

The primary aim of this study is to conduct a cluster RCT comparing the new DAFNE*plus* intervention to the existing DAFNE programme to answer the following question:

In adults with T1D, will modifying the existing DAFNE programme and developing structured professional input, using learning from our recent research, behaviour change theory and new forms of technological support, produce improved and sustained diabetes self-management behaviours, leading to better glucose control than currently achieved, using the existing DAFNE intervention, without compromising quality of life?

The primary objective is to assess the effects of the intervention on glycaemic control, as measured by HbA1c at 12 months.

The secondary objectives of this trial are:

1. To compare the effects of the intervention (DAFNE*plus*) to standard DAFNE on diabetes-specific quality of life.
2. To compare the medium term effect of the intervention (DAFNE*plus*) to standard DANFE on glycaemic control as measured by HbA1c using data at 6 months.
3. To compare the effects of the intervention (DAFNE*plus*) to standard DAFNE on other biomedical outcomes.
4. To compare the effects of the intervention (DAFNE*plus*) to standard DAFNE on psychological outcomes.
5. To undertake a mixed methods process evaluation to aid understanding of the RCT findings, and to inform decision making about the implementation of DAFNE*plus* in clinical care post-trial.
6. To assess fidelity of delivery of the DAFNE*plus* intervention.
7. To undertake a health economic analysis to determine the cost-effectiveness of DAFNE*plus* versus standard DAFNE.

Methods and analysis

Trial Design

The study will use a pragmatic cluster randomised controlled trial design. This is required since ‘contamination’ of the control arm may occur if DAFNE healthcare professionals, trained in the new programme were to deliver standard DAFNE. Hence the randomisation of DAFNE centres rather than

For peer review only

individuals[16]. Figure 1 shows the flow of participants through the trial (**s**ee supplementary material 1 for WHO Trial Registration Data Set).

[Insert Figure 1 here]

Study setting

The trial will be delivered in adult diabetes centres in secondary care NHS hospitals in the UK. The eligibility criteria for study centres are:

1. Diabetes centre delivering DAFNE to adults with T1D;
2. At least three DAFNE educators trained in delivering the 5-week model of DAFNE;
3. Delivery of sufficient DAFNE courses per year to recruit study sample.

Adults with T1D eligible for or referred to DAFNE courses at participating centres as part of usual care will be eligible to be invited to participate in the RCT, and standard criteria for referral to DAFNE will be utilised.

Eligibility criteria

Inclusion criteria:

1. Adults (≥18 years);
2. Diagnosis of type 1 diabetes for at least 6 months, or post-honeymoon[1];
3. Prepared to undertake multiple daily injection (MDI) therapy;
4. Prepared to undertake frequent self-monitoring of blood glucose;
5. Confirms availability to attend all sessions as part of the intervention;
6. Investigator has confidence that the patient is capable of adhering to all the trial protocol requirements.

Exclusion criteria:

1. Current use of continuous subcutaneous insulin infusion (CSII) pump therapy;
2. HbA1c > 12% (108mmol/mol) (Investigators can use their judgement, informed by standard DAFNE guidelines and in agreement with the trial team, to include participants with HbA1c >12%);
3. Serious diabetic complications (e.g. blindness, renal dialysis). (Investigators can use their clinical judgement, informed by standard DAFNE guidelines and in agreement with the trial team);
4. Other serious co-morbidities e.g. psychosis, diagnosed eating disorder (Investigators can use their clinical judgement, informed by standard DAFNE guidelines and in agreement with the trial team);
5. Previous participation in standard DAFNE course less than 5 years before proposed study enrolment date;
6. Unable to speak/hear/understand/read or write in English;

---

[1] The honeymoon period refers to the time when, post-diagnosis, people start taking insulin injections, and their insulin producing cells sometimes recover temporarily (generally around 3 -12 months. The dose of insulin needed might reduce during this period, and some people might even need to stop using insulin for a while, but eventually it will be needed again. The criteria for referral to DAFNE at least 6 months after diagnosis is to allow for the honeymoon period to have passed before attendance at the course.

For peer review only

7. Unable to give written informed consent.

Recruitment

Patient participants will be identified from current caseloads of adults with T1D from each participating centre. They will be sent an invitation letter and information sheet before the course. A member of the clinical team in participating centres will then telephone potential participants to discuss whether or not they are interested in principle in taking part. If interested, they will be asked to consent to participate at their baseline visit. In both trial arms, if they do not want to take part in the research they will be offered attendance at a standard DAFNE course that is not part of this trial, if that is their wish. Reasons for non-participation in the trial will be recorded.

In order to maximise recruitment to the courses, a reserve list of eligible patients will be held at participating centres. Eligible patients may also be invited to take part by their HCP during routine face-to-face appointments, or via telephone. Trial information meetings may also be held during the recruitment period at various locations in centres.

Written informed consent will be obtained from all participants. Members of the local study teams will be responsible for taking informed consent from potentially eligible study participants at the DAFNE centres. The process for obtaining participant informed consent will be in accordance with the REC guidance, and Good Clinical Practice (GCP) and any other regulatory requirements that might be introduced.

Written informed consent to contribute to the process evaluation will also be taken from HCPs in participating sites by the central study team.

Interventions

Standard DAFNE (control arm)

DAFNE is a skill-based structured education programme for adults with T1D delivered in the NHS. Two evidence-based models of delivering standard DAFNE are in operation, whereby the five sessions of the course are delivered weekly or daily, as described elsewhere [17]. Each course is delivered to seven participants on average (minimum of four and maximum of eight). Standard DAFNE will be delivered, as usual care, by trained DAFNE educators in the NHS, including diabetes specialist nurses, dietitians and physicians.

The aim of the course is to train adults with T1D in the skills to manage their condition effectively. It covers numerous topics in a progressive modular based structure. In addition to the five days of the course, participants are asked to attend a baseline appointment before the DAFNE course, and they are also typically invited to attend an optional group follow-up session 6-8 weeks after the course. They may also attend routine appointments every 6-12 months and seek ad-hoc support from local diabetes clinicians post-course.

For peer review only

For the purposes of this study, the control arm will be the 5-week model of standard DAFNE to match the frequency of sessions offered in DAFNE*plus*. All participants in the control arm will be given access to a stand-alone bolus calculator to assist them with calculating insulin doses. There will be no structured follow-up appointments beyond those provided in usual care. To qualify as adherent for statistical purposes, participants need to have attended the equivalent of four days of the course including days one and two which are mandatory; it will be acceptable to include half days in the total.

DAFNE*plus* (intervention arm)

DAFNE*plus* will be delivered by trained DAFNE educators in the NHS[2]. These will be HCPs including diabetes specialist nurses, dietitians and physicians, all of whom will be using DAFNE principles as an integral part of the management of T1D in adults. DAFNE*plus* is a complex intervention, defined by the Medical Research Council[18] as having 'several interacting components', described in summary below.

The development of the content and structure of the DAFNE*plus* programme was informed by the Behaviour Change Wheel (BCW) framework[19]. The intervention's proposed functions are served by behaviour change techniques (BCTs), specified in the hierarchical Behaviour Change Technique Taxonomy v1 (BCTTv1)[20], deemed its 'active ingredients'[21]. The development of the DAFNE*plus* programme (described in [22]) was informed by expert consensus, integrating data on participant- and clinician-generated barriers and facilitators to sustaining DAFNE with the findings from a synthesis of qualitative evidence about post-DAFNE challenges[22]. Prior to this RCT, the DAFNE*plus* programme was piloted in three NHS Diabetes Centres.

The DAFNE*plus* programme comprises three components:

a) **DAFNE*plus* course**

The group-based course component of the DAFNE*plus* programme is delivered one day per week, over five consecutive weeks, and is based on a revision of the standard DAFNE five-week curriculum, with a view to strengthening and sustaining self-management behaviours over a longer term to enable them to achieve blood glucose levels closer to target. Participants will attend an individual pre-course appointment approximately two weeks before the course which serves as their introduction to the programme, during which they are given access to and trained in using the DAFNE*plus* technology (see below), as well as a bolus calculator.

New sessions included in the DAFNE*plus* course include technology assisted individual review, emotional aspects of living with diabetes and its management, harnessing social support, and behaviour change – including additional support for action planning and relapse prevention to help participants achieve their self-management goals. The curriculum was revised

[2] In DAFNE*plus*, those delivering the intervention are referred to as 'facilitators', as opposed to 'educators' in standard DAFNE.

to be consistent with modern approaches to the recommended language used in diabetes care[23]. Requirements to qualify as adherent for statistical purposes are defined above.

b) **Structured follow-up support**

The model of structured follow-up support builds upon the clinical and behavioural skills introduced during the course to enable participants to maximise the efficacy of key DAFNE*plus* principles to improve self-management and achieve/sustain glycaemic targets. As part of the trial, up to five one-to-one consultations (face-to-face, telephone or in some centres, web-based video calling) with a DAFNE facilitator will be offered, delivered at progressively wider spaced intervals during the 12 months after the course. Appointments are supported by paperwork to 'activate' both the participant and the facilitator prior to meeting.

The purpose of these individual sessions is to review participants' progress with managing their diabetes, including progress with their action plans, review blood glucose data on the DAFNE*plus* website, revise course material, address any additional clinical needs, and signpost participants to any relevant sources of support. In addition, ad-hoc support by telephone, email or web-based video calling will be available, as necessary. To qualify as adherent for statistical purposes, participants will need to have attended a minimum of three follow-up sessions.

c) **Digital technology**

The DAFNE*plus* programme incorporates two forms of digital technology via the DAFNE*plus* website and box. Participants will be given access and training at the pre-course appointment, so that they can use the technology before and throughout the 12 month programme. The DAFNE*plus* box (*Withcare*+) transmits, stores and displays blood glucose (and other) data on a secure-server via the DAFNE*plus* website in formats to help people with T1D and their HCPs recognise and interpret blood glucose patterns. The website also includes an e-learning section to help maintain knowledge of the DAFNE*plus* approach.

Training and supervision

A clinical psychologist who specialises in diabetes and is experienced in training diabetes professionals in behaviour change skills will lead the development and delivery of DAFNE*plus* facilitator training and supervision. The training programme is delivered over a maximum of five days and will build on the existing skill-set of DAFNE facilitators but also draw on additional behavioural science to deliver the revised curriculum.

Throughout the trial, facilitators in each centre will be offered supervision by the clinical psychologist and a DAFNE*plus* facilitator. Supervision will comprise of weekly teleconferences before and during the first DAFNE*plus* course, weekly email supervision (for subsequent courses) and ad-hoc remote support to allow issues that arise to be addressed in a timely manner during the trial.

For peer review only

Criteria for withdrawal from or discontinuation of trial treatment

The decision regarding participation in the study is entirely voluntary, and consent regarding study participation may be withdrawn at any time without affecting the quality or quantity of future medical care. No study-specific interventions will be undertaken before informed consent has been obtained.

A participant will be classed as complete if they have continued in the study until the last protocol defined intervention (final 12 month outcome assessment), although there may be missing data for individual participants.

Random allocation

Upon recruitment of centres and following ethical approval, the participating centres will be randomised on a 1:1 basis to control or the intervention arm of the trial by the trial statistician. As there are numerous stratification variables that have been identified as clinically important and the small number of randomising centres, a covariate constrained methodology[24] will be employed. The centres will be matched on the number of patients within the centre, number of educators within the centre and number of previous DAFNE courses delivered (as a marker of centre experience) to balance centres between the two arms of the trial.

Blinding

Due to the nature of the intervention, it is not possible for members of the study team working directly with participants or the intervention to be blinded. Additionally, the blinding of the statistician is problematic due to the cluster level randomisation. Statisticians are usually involved within TMG discussions and have access to status reports where the potential for unintentional unblinding is a high possibility. It is considered important for the statistician to be included in these aspects of the trial management and so after discussion with senior statisticians at the Clinical Trials Research Unit (CTRU) and the independent statistician on the Trial Steering Committee, it has been deemed acceptable that the statisticians are not blind within this study.

Outcomes

Table 1 shows a breakdown of all outcome measures.

Biomedical outcomes

The primary biomedical outcome is an integrated measure of glucose levels over the previous 4-6 weeks, defined by HbA1c (using a centralised assay to ensure standardisation). The primary endpoint is HbA1c at 12 months, in those entering the trial with HbA1c >7.5% (58 mmol/mol), and HbA1c at 6 months is the secondary endpoint.

For peer review only

Our primary aim is to compare HbA1c between the two arms and we have therefore confined our primary analysis to those with raised A1c values at baseline. We therefore excluded those with an HbA1c below 7.5% (58 mmol/mol) when calculating the primary endpoint as these people have less need to reduce their HbA1c.

However, we have included participants with lower A1c values to ensure we can calculate important secondary outcomes part rates of hypoglycaemia, and other biomedical and psychological outcomes. We have estimated the expected proportion of participants with A1c values above 7.5% at 75% of those currently undertaking DAFNE courses based on a national research database.

Other secondary outcomes are the number of participants achieving either an HbA1c <7.5% (58 mmol/mol) or a decrease in HbA1c of ≥0.5% (≥5.5 mmol/mol) which will be calculated at both 6 and 12 months post course. These cut-off points are recognised throughout the diabetes research community as being clinically relevant[25]. We will also collect and analyse 24-month outcome data (HbA1c and severe hypoglycaemic episodes) and analyse after the main study has closed and been reported based on locally available clinical data which is routinely collected annually in clinical centres.

Other secondary biomedical outcomes will include: Severe hypoglycaemia, as defined by the American Diabetes Association[26], denotes severe cognitive impairment requiring external assistance for recovery, both rates and proportion of those affected; Diabetic ketoacidosis, both rates and proportion of those affected; weight; Body Mass Index; Blood Pressure; Lipids; Albumin/creatinine ratio.

Psychological outcomes and process evaluation

Quantitative outcomes

Psychological outcomes and process measures will be collected via self-completed postal or online questionnaires at baseline, course completion, 3, 6, 9 and 12 months (see Table 1).

The primary psychological outcome is the impact of diabetes on quality of life assessed at 12 months using a 15-domain version of the Audit-Dependent Diabetes Quality of Life Questionnaire (ADDQoL-15)[27].

Additional psychological constructs are assessed with validated questionnaires and study-specific individual items, based upon: existing knowledge about their association with the trial's primary biomedical outcome (HbA1c) and primary psychological outcome (diabetes-specific quality of life), including the findings of the YourSAY survey[28]; previous work with the DAFNE intervention, and the theoretical framework underpinning the DAFNE*plus* intervention development and possible treatment mechanisms[19,29,30].

Qualitative outcomes

For peer review only

Interviews will be undertaken with a subset of participants randomized to the intervention at baseline, course completion, 3 months and 12 months (Figure 1) to explore how key elements of the intervention influence and inform changes to, and maintenance of, key self-management behaviours over time. Facilitators will be interviewed from across the intervention sites to explore their experiences of intervention delivery and their views about the training, resourcing and support staff would need to deliver DAFNE*plus* in routine care.

Fidelity assessment

We will explore fidelity of delivery using two methods to assess the extent to which the intervention content specified in the DAFNE/DAFNE*plus* manuals is delivered as intended: self-report checklists completed by educators/facilitators, and objectively analysed delivery from session audio recordings. Fidelity of delivery will be assessed in standard DAFNE as well as DAFNE*plus* in order to assess any loss of treatment differentiation and potential contamination between the two arms.

1) Self-report checklists: Facilitators will complete checklists after each session. Each checklist lists the components intended to be delivered in each session (according to the manual). These components correspond to different behaviour change techniques (BCTs). Each component will be rated as fully, partially or not delivered, with space for additional comments. The proportion of intended components rated as partially/fully delivered by educators/facilitators will be calculated, with <50% of intended content delivered classified as low fidelity; 51-79% as moderate fidelity, and 80-100%' as high fidelity[31].

2) Objectively analysed delivery: A subsample of group course sessions in both arms will be audio-recorded and transcribed verbatim. Transcripts will be coded into component BCTs using an established taxonomy[20]. BCTs identified in each session transcript will be compared to corresponding section of the intervention manual that specifies which BCTs are intended to be delivered in that session. Fidelity will be calculated in terms of the percentage of manual-specified BCTs delivered as intended. Additional BCTs delivered that are not specified in the curricula will also be noted.

Detailed plans for the process evaluation are in supplementary material 2.

Health economic outcomes

Table 1 details the health economic data collected in the trial. In addition, data collected from the DAFNE*plus* website will be used to cost the intervention. The analysis population for the health economic analyses will include all trial participants, as it is important that the analysis of health economic data includes all participants who would be eligible to receive DAFNE*plus* (if it were to be implemented). In line with the statistical analysis, we will conduct subgroup analyses in participants with an HbA1c ≤ 7.5% and > 7.5% (58 mmol/mol).

Two health economic analyses will be conducted, a primary long-term analysis using the Sheffield T1D Policy Model and a secondary analysis of the data collected in the trial. All health economic analysis will compare the incremental cost-effectiveness ratio of DAFNE*plus* versus DAFNE to standard NICE

thresholds to determine cost-effectiveness[32]. See supplementary material 3 for detailed plans for the economic evaluation.

Safety outcomes

Adverse Events

Study centres are only required to report as adverse events episodes of diabetic ketoacidosis and severe hypoglycaemia which while not requiring admission to hospital have been noted by either the participant or their relative/partner etc. These will be recorded on the data collection form and database.

Reporting

We do not anticipate many SAEs related specifically to DAFNE*plus* or standard DAFNE but will report any which are deemed related to the study intervention and or are unexpected to the Sponsor and the REC in line with best practice.

Sample size

It is expected that there will be 882 patients referred for DAFNE courses within the 15-month recruitment window and of these it is expected 75% (662 patients) will be recruited, equivalent to 47 participants at each of the 14 centres. From current DAFNE data, a further 25% are expected not to meet the primary analysis population criteria of baseline HbA1c greater than 7.5% (58 mmol/mol), leaving 497 participants. Finally, we anticipate 15% of participants to be lost to follow-up by the 12-month stage, therefore giving a primary analysis population of 422 participants. The sample size takes into account the design effect associated with the cluster design of the study. With an ICC of 1.5% (from previous DAFNE data) and 30 participants per cluster (422 participants over 14 centres) the design effect is 1.435 leaving the effective total sample size of n=294 participants (n=147 per arm).

Using a two sample comparison of mean HbA1c at the 12-month follow-up with 2-sided alpha of 5%, a correlation of 0.5 between baseline and final values and a standard deviation of 1.45 (from previous DAFNE data), the trial sample gives 92% power to detect a 0.5% difference in HbA1c (the minimum clinically important difference) between the two treatment groups in the study.

For peer review only

Table 1 – List of outcome and process measures

Concepts	Questionnaire	Baseline: Pre-course appt	Course Completion	Post-course assessments			
				3m*	6m*	9m*	12m*
**Demographic / Clinical**							
Glycaemic Control (HbA1c)	N/A	✓			✓		✓
Lipids	N/A	✓					✓
Body mass index (height/weight)	N/A	✓			✓		✓
Blood Pressure	N/A	✓			✓		✓
Episodes of severe Hypoglycaemia	N/A	✓			✓		✓
Episodes of Ketoacidosis	N/A	✓			✓		✓
Demographics	Individual items	✓			✓		✓
Hypoglycaemia awareness	Gold score[33] and DAFNE hypo awareness measure	✓			✓		✓
**Primary Psychological Outcomes**							
Diabetes-specific quality of life	ADDQoL-15[27]	✓			✓		✓
**Secondary Psychological Outcomes**							
Diabetes distress	Problem Areas In Diabetes (PAID-11) (short-form)[34]	✓			✓		✓
Diabetes-specific quality of life	Dawn Impact of Diabetes Profile (DIDP)[35]	✓			✓		✓
Diabetes-specific positive well-being	4-item sub-scale of the Well Being Questionnaire (W-BQ28)[36]	✓			✓		✓
Fear of hypoglycaemia	Hypoglycaemia Fear Survey-11 (HFS-11) short-form)[37]	✓			✓		✓
Health status	Health and Self-Management in Diabetes (HASMID)[38]	✓			✓		✓
Health status	EQ-5D-5L[39]	✓			✓		✓
Healthcare utilisation	Individual items	✓			✓		✓
Resource allocation	Individual items		✓				
**Process Measures**							

For peer review only

Concepts	Questionnaire	Baseline: Pre-course appt	Course Completion	Post-course assessments			
				3m*	6m*	9m*	12m*
Diabetes Management Experiences (satisfaction)	Diabetes Management Experiences Questionnaire (DME-Q)[40]	✓	✓	✓		✓	
Self-regulatory skills/behavioural regulation	Self-Regulation Questionnaire (SRQ-T1D)*[41]	✓	✓	✓		✓	
Diabetes strengths and resilience	Diabetes Strengths and Resilience Questionnaire (DSRQ)[42]	✓	✓	✓		✓	
Beliefs about capabilities: diabetes self-care	Confidence in Diabetes Scale (CIDS)*[43]	✓	✓	✓		✓	
Beliefs about capabilities: hypoglycaemia confidence	Hypoglycaemia Confidence Scale (HCS)[44]	✓	✓	✓		✓	
Diabetes-specific self-care behaviours	Diabetes Self-Care Behaviours (SCB-T1D)[45]	✓	✓	✓		✓	
Beliefs about consequences of engaging in DAFNE behaviours and weaving diabetes management into everyday routines	Individual items*	✓	✓	✓		✓	
Evaluation of technology (DAFNE*plus* website in intervention group and bolus calculator in control group)	System Usability Scale[46]		✓	✓		✓	

*Description about the development and modifications of these questionnaires and individual items are detailed in supplementary material 4

For peer review only

Statistical analysis

The primary analysis population will be participants that had an HbA1c greater than 7.5% (58 mmol/mol) at baseline and the analysis will be completed on an intention to treat (ITT) basis. This primary analysis is to assess the difference between the two treatment groups on the mean HbA1c at 12 months which will be completed using a multiple linear regression model with coefficients estimated using generalised estimating equations (GEE) to account for the clustering design. A 95% confidence interval for the difference between the two treatment groups will be presented. Appropriate covariates will be included in the model, along with the participant's baseline HbA1c, to adjust the treatment effect accordingly.

The secondary analysis population is all consenting participants in the trial and analysis will again be completed on an ITT basis. This population will also be used to assess the difference in psychological outcomes between the two treatment groups using the same model as for the primary analysis.

A full statistical analysis plan has been written and was circulated to the Trial Management Group and Trial Steering Committee before being signed-off. This is available in supplementary material 5. All analysis results will be reported according to the revised CONSORT 2010 statement for cluster RCTs[47].

Data collection and management

Case report forms will be completed by DAFNE facilitators/educators at each study visit. Follow-up questionnaires will be self-completed by participants at each follow-up point. Participants will be allocated a unique identification number to identify them throughout the trial.

Plans to promote retention and follow-up of all trial participants include research appointments being scheduled and followed up by their clinical teams at 6 and 12-months. Overdue questionnaires are followed-up with an email reminder and then telephone call from CTRU. All participants received email newsletters to update them on trial progress.

Data will be entered onto the DAFNE*plus* database on CTRU's secure online system, hosted on University of Sheffield servers. Access is restricted such that users can enter and view only information required to perform their role.

Identifiable data will be shared with CTRU and the supporting study team and DAFNE*plus* website teams. Consent will be obtained from the participant for this to occur. Data will be stored securely on access-restricted network drive folders in accordance with CTRU standard operating procedures (SOPs).

All consent forms and questionnaires will be kept in a locked filing cabinet in a secured area and will be retained for a minimum of 5 years after study completion, in accordance with the sponsor's archiving requirements. Sheffield CTRU may request consent forms to be sent from the research site to the CTRU via post or email as part of remote monitoring procedures.

For peer review only

The nature, frequency and intensity of trial monitoring will be outlined in the site monitoring plan, which will be devised in accordance with CTRU SOPs.

Patient and Public Involvement

In addition to the patient representation on the trial oversight committees, this trial is supported by a Patient Advisory Group who have and will continue to meet regularly during the conduct of the trial (and the wider programme grant). Patient input has been sought throughout on the trial and intervention design, the informational material to support trial conduct and patient burden.

Trial oversight committees

Two oversight committees have been established to oversee the conduct of this trial – Trial Steering Committee (TSC) and Trial Management Group, the composition of each is listed at the beginning of this paper. A Data Monitoring and Ethics Committee has not been convened, on the grounds that the study is low risk, in line with CTRU SOP GOV003. This has been approved by the Sponsor and TSC.

Ethics and dissemination

The RCT was not initiated until the protocol, informed consent forms and participant information sheets received approval from the Research Ethics Committee, the Health Research Authority and local Capacity and Capability is confirmed by the respective National Health Service Research & Development departments. MHRA approval was not required for this study.

The RCT is being conducted in accordance with the ethical principles that have their origin in the Declaration of Helsinki[48]; the principles of Good Clinical Practice, and the UK Framework for Health and Social Care Research[49].

Outputs from the trial will be generated in accordance with the communication and dissemination strategy. A number of academic outputs will be produced as the data are analysed from the trial. Journals will be selected based on the highest possible impact. Other stakeholder specific outputs in relevant formats will also be produced for commissioners, third sector, and user advocacy organisations.

For peer review only

Acknowledgements
The authors would like to thank all of the patients who are taking part in this trial, and all the participating clinical sites. The authors would also like to thank Becky Brown for administrative support to the trial, and Chris Turtle for data management.

Conflicts of interest
SA reports Advisory Board membership for Novonordisk, Medtronic, Roche and Abbott. MC reports personal fees from University of Sheffield. DP reports non-financial support from Novo Nordisk, Eli Lilly and Company Limited, Abbott Diabetes Care, Sanofi-Aventis and Medtronic. SH reports Advisory board membership and consultancy for Sanofi-Aventis, Boeringher-Ingelheim, Eli-Lilly, Novo-Nordisk, Springer Medical, Zealand Pharma and UN-EEG. Personal fees for speaker panels for Novo-Nordisk. JS reports Personal fees, non-financial support and other (advisory board membership/support to attend educational meetings) from Medtronic, Roche Diabetes Care and Sanofi Diabetes, grants and personal fees from Abbott Diabetes Care, grants and personal fees from AstraZeneca.

Data statement
No data from this protocol are available. Only approved personnel may access the data. Data and statistical code will be available on request.

Author contributions
ES, TC, ECr, and JSc have responsibility for running the trial and acquisition of the trial data, under the leadership of SH as Chief Investigator. All other authors (ECo, SA, WB, MB, AB, MJC, PC, PCh, DC, CC, NdZ, ME, JE, CG, TG, DH, ZH, JL, FL, SM, DJP, DR, JS, SSF, CT, GT, NT, LY, AZ, SH) made substantial contributions to the conception or design of the work, whether as co-investigators and/or as members of the Trial Management Group. ECo prepared the first draft of the manuscript and edited this with input from other authors. All authors provided critical review and approval of the final manuscript.

Funding:
This work is funded by the National Institute for Health Research through their Programme Grants for Applied Research Programme (Ref: RP-PG-0514-20013) and NHS England provide funding for Excess Treatment Costs.

For peer review only

References

1 Diabetes UK. Diabetes UK: Facts and Stats, October 2016. 2016.

2 Group TDC and CTR. The Effect of Intensive Treatment of Diabetes on the Development and Progression of Long-Term Complications in Insulin-Dependent Diabetes Mellitus. *N Engl J Med* 1993;**329**:977–86. doi:10.1056/NEJM199309303291401

3 Heller S. Hypoglycaemia and diabetes. In: Pickup, J, WIlliams G, ed. *Textbook of Diabetes*. Oxford: : Blackwell Science 2003. 33.1-33.19.

4 DAFNE. DAFNE Home. http://www.dafne.uk.com/ (accessed 26 Feb 2020).

5 DAFNE Study Group DS. Training in flexible, intensive insulin management to enable dietary freedom in people with type 1 diabetes: dose adjustment for normal eating (DAFNE) randomised controlled trial. *BMJ* 2002;**325**:746.http://www.ncbi.nlm.nih.gov/pubmed/12364302 (accessed 30 Jan 2018).

6 Heller S, Lawton J, Amiel S, *et al.* Improving management of type 1 diabetes in the UK: the Dose Adjustment For Normal Eating (DAFNE) programme as a research test-bed. A mixed-method analysis of the barriers to and facilitators of successful diabetes self-management, a health economic analysis, a cluster randomised controlled trial of different models of delivery of an educational intervention and the potential of insulin pumps and additional educator input to improve outcomes. *Program Grants Appl Res* 2014;**2**:1–188. doi:10.3310/pgfar02050

7 National Institute for Health and Care Excellence. Type 1 diabetes in adults: diagnosis and management | Guidance and guidelines | NICE. NICE https://www.nice.org.uk/guidance/ng17 (accessed 2 Feb 2018).

8 McKnight JA, Wild SH, Lamb MJE, *et al.* Glycaemic control of Type 1 diabetes in clinical practice early in the 21st century: an international comparison. *Diabet Med* 2015;**32**:1036–50. doi:10.1111/dme.12676

9 Rankin D, Barnard K, Elliott J, *et al.* Type 1 diabetes patients' experiences of, and need for, social support after attending a structured education programme: A qualitative longitudinal investigation. *J Clin Nurs* 2014;**23**:2919–27. doi:10.1111/jocn.12539

10 Lawton J, Rankin D, Cooke DD, *et al.* Self-treating hypoglycaemia: A longitudinal qualitative investigation of the experiences and views of people with Type 1 diabetes. *Diabet Med* 2013;**30**:209–15. doi:10.1111/dme.12007

11 Lawton J, Rankin D, Cooke D, *et al.* Patients' experiences of adjusting insulin doses when implementing flexible intensive insulin therapy: A longitudinal, qualitative investigation. *Diabetes Res Clin Pract* 2012;**98**:236–42. doi:10.1016/j.diabres.2012.09.024

12 Rankin D, Cooke DD, Elliott J, *et al.* Supporting self-management after attending a structured education programme: A qualitative longitudinal investigation of type 1 diabetes patients experiences and views. *BMC Public Health* 2012;**12**:652. doi:10.1186/1471-2458-12-652

13 Rankin D, Cooke DD, Heller S, *et al.* Experiences of using blood glucose targets when following an intensive insulin regimen: A qualitative longitudinal investigation involving patients with Type1 diabetes. *Diabet Med* 2012;**29**:1079–84. doi:10.1111/j.1464-5491.2012.03670.x

14 Lawton J, Rankin D, Cooke DD, *et al.* Dose Adjustment for Normal Eating: A qualitative longitudinal exploration of the food and eating practices of type 1 diabetes patients converted to flexible intensive insulin therapy in the UK. *Diabetes Res Clin Pract* 2011;**91**:87–93. doi:10.1016/j.diabres.2010.11.007

15 Rankin D, Cooke DD, Clark M, *et al.* How and why do patients with Type1 diabetes sustain their use of flexible intensive insulin therapy? A qualitative

For peer review only

longitudinal investigation of patients' self-management practices following attendance at a Dose Adjustment for Normal Eating (DAFNE) course. *Diabet Med* 2011;**28**:532–8. doi:10.1111/j.1464-5491.2011.03243.x
16 Campbell MJ, Walters SJ. *How to Design, Analyse and Report Cluster Randomised Trials in Medicine and Health Related Research*. Chichester, UK: : John Wiley & Sons, Ltd 2014. doi:10.1002/9781118763452
17 Elliott J, Lawton J, Rankin D, *et al.* The 5x1 DAFNE study protocol: a cluster randomised trial comparing a standard 5 day DAFNE course delivered over 1 week against DAFNE training delivered over 1 day a week for 5 consecutive weeks. *BMC Endocr Disord* 2012;**12**:28. doi:10.1186/1472-6823-12-28
18 Craig P, Dieppe P, Macintyre S, *et al.* Developing and evaluating complex interventions: the new Medical Research Council guidance. *BMJ* 2008;**337**:a1655. doi:10.1136/BMJ.A1655
19 Michie S, van Stralen MM, West R. The behaviour change wheel: A new method for characterising and designing behaviour change interventions. *Implement Sci* 2011;**6**:42. doi:10.1186/1748-5908-6-42
20 Michie S, Richardson M, Johnston M, *et al.* The Behavior Change Technique Taxonomy (v1) of 93 Hierarchically Clustered Techniques: Building an International Consensus for the Reporting of Behavior Change Interventions. *Ann Behav Med* 2013;**46**:81–95. doi:10.1007/s12160-013-9486-6
21 Michie, S, Atkins, L, West R. *The behaviour change wheel: a guide to designing interventions*. London: : Silverback Publishing 2014.
22 Campbell F, Lawton J, Rankin D, *et al.* Follow-Up Support for Effective type 1 Diabetes self-management (The FUSED Model): A systematic review and meta-ethnography of the barriers, facilitators and recommendations for sustaining self-management skills after attending a structured education programme. *BMC Health Serv Res* 2018;**18**:898. doi:10.1186/s12913-018-3655-z
23 Speight J, Conn J, Dunning T, *et al.* Diabetes Australia position statement. A new language for diabetes: Improving communications with and about people with diabetes. *Diabetes Res Clin Pract* 2012;**97**:425–31. doi:10.1016/j.diabres.2012.03.015
24 Li F, Lokhnygina Y, Murray DM, *et al.* An evaluation of constrained randomization for the design and analysis of group-randomized trials. *Stat Med* 2016;**35**:1565–79. doi:10.1002/sim.6813
25 REPOSE Study Group TRS. Relative effectiveness of insulin pump treatment over multiple daily injections and structured education during flexible intensive insulin treatment for type 1 diabetes: cluster randomised trial (REPOSE). *BMJ* 2017;**356**:j1285. doi:10.1136/BMJ.J1285
26 International Hypoglycaemia Study Group. Glucose Concentrations of Less Than 3.0 mmol/L (54 mg/dL) Should Be Reported in Clinical Trials: A Joint Position Statement of the American Diabetes Association and the European Association for the Study of Diabetes: Table 1. *Diabetes Care* 2017;**40**:155–7. doi:10.2337/dc16-2215
27 Bradley C, Todd C, Gorton T, *et al.* The development of an individualized questionnaire measure of perceived impact of diabetes on quality of life: The ADDQoL. *Qual Life Res* 1999;**8**:79–91. doi:10.1023/A:1026485130100
28 SPEIGHT J, HOLMES-TRUSCOTT E, HENDRIECKX C, *et al.* 809-P: Comparison of the Acceptability and Psychometric Properties of Scales Assessing the Impact of Type 1 Diabetes on Quality of Life: Results of the Your SAY: QoL Study. *Diabetes* 2019;**68**:809-P. doi:10.2337/db19-809-p
29 Cooke D, Bond R, Lawton J, *et al.* Modeling predictors of changes in glycemic control and diabetes-specific quality of life amongst adults with type 1 diabetes 1 year after structured education in flexible, intensive insulin therapy. *J Behav Med* 2015;**38**:817–29. doi:10.1007/s10865-015-9649-y

For peer review only

30 Michie S, Johnston M, Abraham C, *et al.* Making psychological theory useful for implementing evidence based practice: a consensus approach. *Qual Saf Heal Care* 2005;**14**:26–33. doi:10.1136/qshc.2004.011155
31 Borrelli B. The assessment, monitoring, and enhancement of treatment fidelity in public health clinical trials. *J Public Health Dent* 2011;**71**:S52–63. doi:10.1111/j.1752-7325.2011.00233.x
32 National Institute for Health and Care Excellence. Guide to the methods of technology appraisal 2013 | Guidance and guidelines | NICE. https://www.nice.org.uk/process/pmg9/chapter/foreword (accessed 30 Jan 2018).
33 Gold AE, MacLeod KM, Frier BM. Frequency of severe hypoglycemia in patients with type I diabetes with impaired awareness of hypoglycemia. *Diabetes Care* 1994;**17**:697–703.http://www.ncbi.nlm.nih.gov/pubmed/7924780 (accessed 30 Jan 2018).
34 Polonsky WH, Anderson BJ, Lohrer PA, *et al.* Assessment of diabetes-related distress. *Diabetes Care* 1995;**18**:754–60.http://www.ncbi.nlm.nih.gov/pubmed/7555499 (accessed 30 Jan 2018).
35 Peyrot M, Rubin RR, Lauritzen T, *et al.* Psychosocial problems and barriers to improved diabetes management: results of the Cross-National Diabetes Attitudes, Wishes and Needs (DAWN) Study. *Diabet Med* 2005;**22**:1379–85. doi:10.1111/j.1464-5491.2005.01644.x
36 Speight, J, Barendse, S, Bradley C. The W-BQ28: further development of the well-being Questionnaire to include diabetes-specific as well as generic subscales and new stress subscales. In: *British Psychological Society, Division of Health Psychology*. Leeds: 1999.
37 Cox DJ, Irvine A, Gonder-Frederick L, *et al.* Fear of hypoglycemia: Quantification, validation, and utilization. *Diabetes Care* 1987;**10**:617–21. doi:10.2337/diacare.10.5.617
38 Carlton J, Elliott J, Rowen D, *et al.* Developing a questionnaire to determine the impact of self-management in diabetes: giving people with diabetes a voice. *Health Qual Life Outcomes* 2017;**15**:146. doi:10.1186/s12955-017-0719-4
39 Herdman M, Gudex C, Lloyd A, *et al.* Development and preliminary testing of the new five-level version of EQ-5D (EQ-5D-5L). *Qual Life Res* 2011;**20**:1727–36. doi:10.1007/s11136-011-9903-x
40 Speight J, Holmes-Truscott E, Little SA, *et al.* Satisfaction with the Use of Different Technologies for Insulin Delivery and Glucose Monitoring Among Adults with Long-Standing Type 1 Diabetes and Problematic Hypoglycemia: 2-Year Follow-Up in the HypoCOMPaSS Randomized Clinical Trial. *Diabetes Technol Ther* 2019;**21**:619–26. doi:10.1089/dia.2019.0152
41 Brown J, Miller W, LewandowskiLA. The Self-Regulation Questionnaire. In: Creek, Van de L, Jackson T, eds. *Innovations in clinical practice: A source book*. Sarasota, Florida: : Professional Resource Press 1999. 281–9.
42 Hilliard ME, Iturralde E, Weissberg-Benchell J, *et al.* The Diabetes Strengths and Resilience Measure for Adolescents With Type 1 Diabetes (DSTAR-Teen): Validation of a New, Brief Self-Report Measure. *J Pediatr Psychol* 2017;**42**:995–1005. doi:10.1093/jpepsy/jsx086
43 Van Der Ven NCW, Weinger K, Yi J, *et al.* The confidence in diabetes self-care scale: Psychometric properties of a new measure of diabetes-specific self-efficacy in Dutch and U.S. patients with type 1 diabetes. *Diabetes Care* 2003;**26**:713–8. doi:10.2337/diacare.26.3.713
44 Polonsky WH, Fisher L, Hessler D, *et al.* Investigating Hypoglycemic Confidence in Type 1 and Type 2 Diabetes. doi:10.1089/dia.2016.0366
45 Mansell P, Grant L, Bond R, Pouwer F, Page N, Davies A, Stenner K, Elliott J, Hopkins D, Jones A, Vallis M, Speight J, Lawton J, Clark M, Heller S CD.

For peer review only

Assessment of Self-Care Behaviours focused on Glycemic Control in Type 1 Diabetes: Development and Validation of the SCB-T1D Questionnaire.
46 Brooke J. SUS-A quick and dirty usability scale. *Usability Eval Ind* 1996;**189**:4–7.
47 Campbell MK, Piaggio G, Elbourne DR, *et al.* Consort 2010 statement: extension to cluster randomised trials. *BMJ* 2012;**345**:e5661. doi:10.1136/BMJ.E5661
48 World Medical Association. World Medical Association Declaration of Helsinki: Recommendations guiding physicians in biomedical research involving human subjects. 1996.
49 Department of Health. UK Framework for Health and Social Care Research. 2017.

For peer review only

Figure 1 – RCT flow diagram

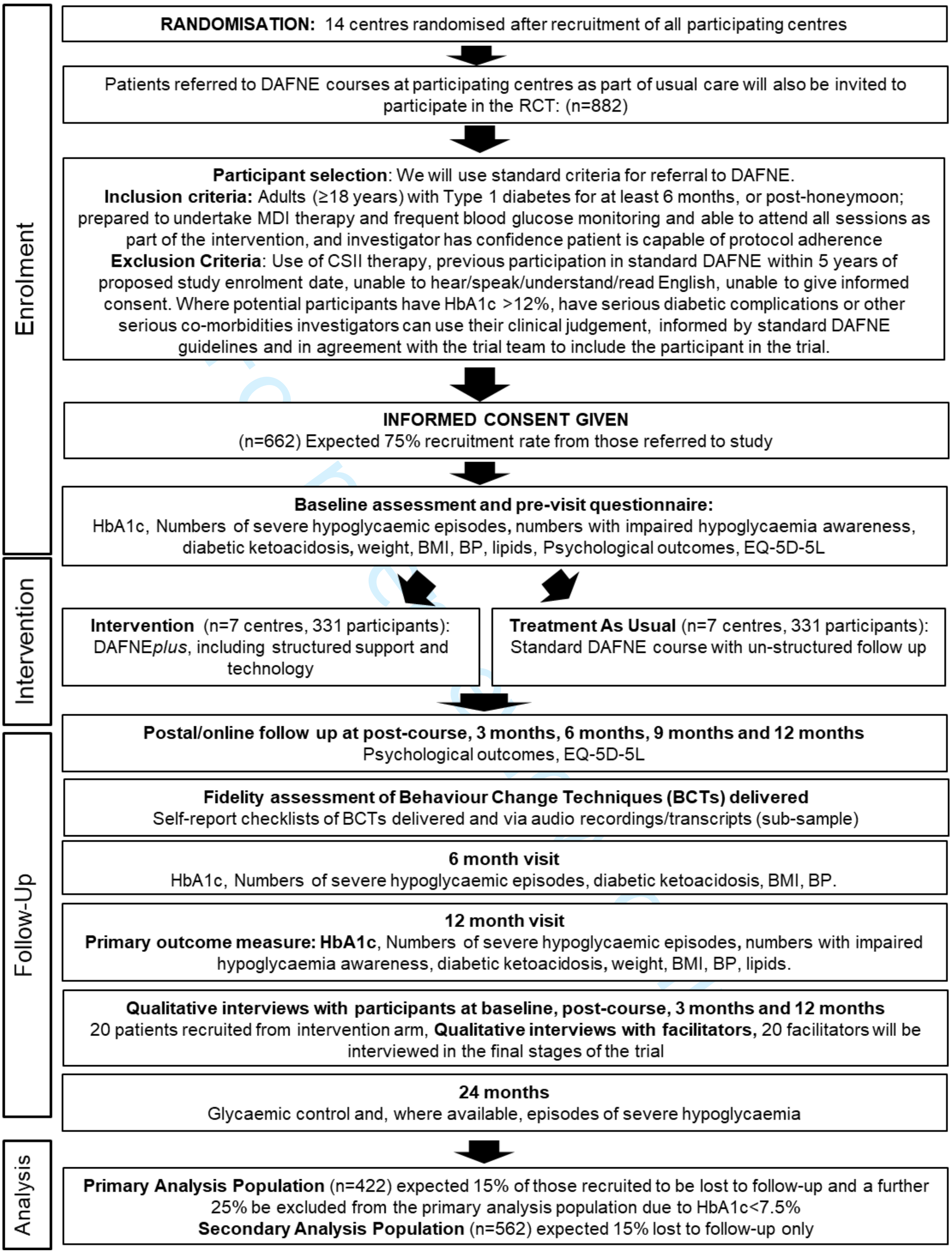

Supplementary material 1 – WHO Trial Registration Dataset

Data Category	Information
Primary registry and trial identifying number	http://www.isrctn.com/ISR ISRCTN42908016
Date of registration in primary registry	08/05/2018
Secondary identifying numbers	Sheffield CTRU: J13-003<br>Sponsor ID: STH20111<br>IRAS: 235621<br>Funding ref: RP-PG-0514-20013<br>REC: 18/SW/0100
Source(s) of monetary or material support	National Institute for Health Research (NIHR) (UK)
Primary sponsor	Sheffield Teaching Hospitals NHS Foundation Trust
Secondary sponsor(s)	N/A
Contact for public queries	Trial manager (Elaine Scott) 0114 222 5158 or dafneplus@sheffield.ac.uk
Contact for scientific queries	Trial manager (Elaine Scott) 0114 222 5158 or dafneplus@sheffield.ac.uk
Public title	DAFNE*plus* Cluster RCT
Scientific title	A cluster randomised controlled trial (RCT) of the DAFNE*plus* (Dose Adjustment for Normal Eating) intervention: A lifelong approach to promote effective self-management in adults with type 1 diabetes
Countries of recruitment	England and Scotland
Health condition(s) or problem(s) studied	Type 1 diabetes
Intervention(s)	DAFNE*plus* (Dose Adjustment for Normal Eating) intervention
Key inclusion and exclusion criteria	*Inclusion criteria:*<br>• Adults (≥18 years);<br>• Diagnosis of type 1 diabetes for at least 6 months, or post-honeymoon;<br>• Prepared to undertake multiple daily injection (MDI) therapy;<br>• Prepared to undertake frequent self-monitoring of blood glucose;<br>• Confirms availability to attend all sessions as part of the intervention;

	• Investigator has confidence that the patient is capable of adhering to all the trial protocol requirements.<br><br>*Exclusion criteria:*<br>• Current use of continuous subcutaneous insulin infusion (CSII) pump therapy<br>• HbA1c > 12%/108 mmol/mol (Investigators can use their judgement, informed by standard DAFNE guidelines and in agreement with the trial team, to include participants with HbA1c >12%/108 mmol/mol).<br>• Serious diabetic complications (e.g. blindness, renal dialysis). (Investigators can use their clinical judgement, informed by standard DAFNE guidelines and in agreement with the trial team).<br>• Other serious co-morbidities e.g. psychosis, diagnosed eating disorder (Investigators can use their clinical judgement, informed by standard DAFNE guidelines and in agreement with the trial team).<br>• Previous participation in standard DAFNE course less than 5 years before proposed study enrolment date<br>• Unable to speak/hear/understand/read write in English<br>• Unable to give written informed consent
Study type	Multi-centre cluster randomised controlled trial with process evaluation and economic evaluation, comparing DAFNE*plus* to standard DAFNE for adults with type 1 diabetes.
Date of first enrolment	01/09/2018
Target sample size	662 participants – 47 per centre.<br><br>Fourteen secondary care diabetes centres in the National Health Service in England and Scotland<br><br>In addition, we aim to recruit 20 DAFNE*plus* facilitators to take part in qualitative interviews for the process evaluation.
Recruitment status	Recruiting
Primary outcome(s)	The primary biomedical outcome is glycaemic control, defined as the change in HbA1c at 12 months (using a centralised assay to ensure standardisation), in those entering the trial with HbA1c >7.5% (estimated at 75% of those currently undertaking DAFNE courses based on our research database).
Key secondary outcomes	Secondary biomedical outcome:<br>Number of participants achieving either an HbA1c <7.5% (58 mmol/mol) or a decrease in HbA1c of ≥0.5% (≥5.5 mmol/mol) (using a centralised assay to ensure standardisation). These endpoints will be calculated using data collected at baseline and 12 months after the course.<br><br>Other secondary biomedical outcomes will include:<br>1. Severe hypoglycaemia, as defined by the American Diabetes Association, denotes severe

For peer review only

	cognitive impairment requiring external assistance for recovery, both rates and proportion of those affected, measured at baseline at 12 months after the course<br>2. Diabetic ketoacidosis, both rates and proportion of those affected, collected at baseline and 12 months after the course<br>3. Weight, measured at baseline and 12 months after the course<br>4. Body Mass Index, measured at baseline and 12 months after the course<br>5. Blood pressure, measured at baseline and 12 months after the course<br>6. Lipids, measured at baseline and 12 months after the course<br>7. Albumin/ creatinine, measured at baseline and 12 months after the course<br><br>The primary psychological outcome is the measurement at 12 months of the Audit-Dependent Diabetes Quality of Life Questionnaire (ADDQoL-15), a thirty-item measure of diabetes-specific quality of life.<br><br>Psychological outcomes, measured at baseline, course completion, 3, 6 and 12 months:<br>1. Dawn Impact of Diabetes Profile<br>2. Problem Areas in Diabetes Scale<br>3. Diabetes-specific positive well-being<br>4. Hypoglycaemia Fear Survey<br><br>Process measures:<br>5. Diabetes Management Experiences Questionnaire<br>6. Self-Regulation/Behavioural Regulation Questionnaire<br>7. Diabetes Strengths & Resilience Questionnaire<br>8. Confidence in Diabetes Scale assesses beliefs about capabilities (self-efficacy).<br>9. Diabetes Self-Care Behaviours<br>10. Hypoglycaemia Confidence Scale<br>11. Beliefs about consequences of engaging in DAFNE behaviours and weaving diabetes management into everyday routines.<br>12. The System Usability Score<br>13. Use and dose received of the DANFE*plus* programme assessed via logs of attendance at group and individual sessions, and use of the DANFE*plus* website<br><br>Hypoglycaemia Awareness<br>14. Hypoglycaemia awareness assessed via Gold score<br><br>Health economic measures assessed at baseline, course completion, 6 and 12 months using:<br>1. Health status – EQ-5D-5L<br>2. Health and Self-Management in Diabetes HASMID<br>3. Healthcare utilisation using a bespoke questionnaire<br>4. Contact between professionals and course participants will also be recorded at each site using questionnaires and data from the DANFE*plus* website (in the intervention arm)

For peer review only

Supplementary material 2 – Protocol for DAFNEplus Process evaluation

Aims and research questions

Understanding processes is as important as evaluating outcomes; process evaluations are complementary to outcomes evaluations and provide knowledge and information of equal value. Process evaluations aim to understand the functioning of an intervention by examining its implementation, mechanisms of impact and how contextual factors (i.e. factors external to the intervention/individual receiving the intervention) might affect its delivery and receipt [1,2]. Without this knowledge we may be able to establish from an outcome evaluation that an intervention 'works', but we will be presuming that the intended intervention was delivered and is effective, and we will not necessarily know how, or why, the intervention works and, hence, if it would have the same clinical and psychological effect if rolled out from a trial situation into routine clinical practice. With a complex intervention such as DAFNE*plus* it may well also be that some elements are more vital to its success than others; hence it is very important that we understand and explore the mechanisms of change on outcome from the perspectives of those receiving the intervention, as well as unintended consequences arising from the delivery and receipt of the intervention.

Our overarching research questions are:

1. **Does the DAFNE*plus* intervention 'work' in the ways intended? If not, why not?**

2. **What are the implications of the findings of the process evaluation for the rollout of DAFNE*plus* in routine clinical practice?**

To answer these over-arching questions, a series of over-lapping sub-questions will be explored:

a) What mechanisms change impact on glycaemic control? That is, how do the different elements of DAFNE*plus* (knowledge/skills, technological, structured follow-up), individuals' interaction with these elements, and individual psychological differences trigger changes in and maintenance of key diabetes self-management behaviours? The theoretical model underpinning the DAFNEplus programme assumes that diabetes self-management behaviours are among the principal determinants of glycaemic control.

b) What mechanisms of change impact on diabetes-specific quality of life?

c) What are participants' experiences of, and views about, key elements of the DAFNE*plus* intervention[1] and how do these influence and inform changes in, and maintenance of, key diabetes self-management behaviours over time?

---

[1] As a result of work undertaken in the pilot phase and MRC guidance to focus on key areas of uncertainty of greatest interest to academic and clinical audiences, a decision has been made to focus upon the technological and resilience/self-compassion elements of the programme.

For peer review only - http://bmjopen.bmj.com/site/about/guidelines.xhtml

d) To what extent is the intervention delivered as intended and are there variations between sites and individuals as to how the DAFNE*plus* intervention is delivered? What are the reasons for any variations?

e) What impact (practical and emotional) does intervention delivery have on facilitators and their workloads; what resourcing and support would facilitators and their colleagues need to deliver DAFNE*plus* in routine clinical practice?

f) Do any unintended consequences arise from the delivery and receipt of the DAFNE*plus* intervention, for participants and/or facilitators?

The data sources for each of the sub-questions are shown in table 1.

Table 1 – Data sources for the process evaluation

Research Question	Data source(s)
a) What mechanisms of change impact on glycaemic control?	• Questionnaire study<br>• Process outcomes<br>• Fidelity assessment<br>• Qualitative (from DAFNE*plus* pilot study)
b) What mechanisms of change impact on diabetes-specific quality of life	• Questionnaire study<br>• Process outcomes
c) What are participants' experiences of DAFNE*plus*?	Qualitative
d) To what extent is the intervention delivered as intended?	• Fidelity assessment<br>• Qualitative
e) What impact does intervention delivery have on facilitators and their workloads?	Qualitative
f) Do any unintended consequences arise from the delivery and receipt of the DAFNE*plus* intervention?	• Qualitative<br>• Questionnaire study<br>• Process outcomes<br>• Fidelity assessment

The process evaluation is composed of three interlinking components: (1) qualitative, (2) quantitative and (3) assessment of fidelity of delivery.

(1) Qualitative component

1.1 Overview

The qualitative component of the process evaluation will be informed by realist and Normalization Process theory (NPT) [3,4]. These choices arise from our recognition that context (i.e. factors external to the DAFNE*plus* intervention and/or the individual receiving the intervention) may influence how the intervention is delivered in different centres and how it is received by different individuals. It is also recognised that, when

For peer review only

a complex intervention, such as DAFNE*plus*, is implemented it can have unintended consequences, which may need to be investigated and, hence, that a flexible and adaptive study design will be required.

An iterative, inductive approach will be used wherein data analysis will commence as soon as data collection begins [5]. This will allow issues arising during early phases of qualitative data collection to inform questions asked in later phases and possibly also sampling. The qualitative research will also be responsive to other aspects of the process evaluation, including the fidelity work. Hence, while case studies will comprise the main element of the qualitative research (see below), costings have been included to allow, if necessary, one-off interviews to be undertaken with a 'booster' sample of patients, facilitators and/or other individuals in the event that the quantitative/fidelity components of the process evaluation highlight issues which require qualitative explanation. One example might be that, if the fidelity work highlights significant variations between trial sites as to how the DAFNE*plus* intervention is delivered, we may decide to interview additional facilitators to better understand why this might be the case.

1.2 Qualitative study design: case study approach

A case study approach will be used because it permits detailed exploration of if, how and why the intervention works in different contexts [6]. Each case will comprise: (a) participants who will be interviewed before, during and following completion of DAFNE*plus*, (b) their facilitators who will be interviewed after the participant's closeout from the trial, (c) information about the input and care the participant receives as part of DAFNE*plus* and their engagement with DAFNE*plus* technologies/resources. It will be possible to access this information via clinical records, the Glucollector website and information documented in case report forms and stored on PROSPECT (the CTRU database). Where identifiable clinical information needs transferring between NHS and University sites files will be encrypted and nhs.net accounts or Google Drive will be used. As part of the process evaluation, we will also have access to recordings of participants' face-to-face follow-up sessions with facilitators – these data are being collected for the fidelity assessment work, and data on utilisation of DAFNE*plus* technological components and adherence. Researchers from the University of Edinburgh will also sit in some DAFNEplus sessions as observers, to familiarise themselves with the processes and material to inform the case studies.

Participant Sampling

Two or three participants from each of the seven DAFNE*plus* sites will be selected for the qualitative work, and these individuals will be purposively sampled so there is representation of people of different ages, HbA1c levels, diabetes duration, gender, occupation, educational background, personal circumstance (e.g. single, partnered, parent) and place of residence (e.g. urban and rural locations).

Data collection: participant interviews

Selected participants will be interviewed at four time-points: prior to attending their course, following their course, and 3 and 12 months post course. Interviews will be informed by topic guides. Prior to undertaking a follow-up interview, a participant's previous interviews will be reviewed. As well as including more generic questions, follow-up interviews will be tailored to allow for follow-up of specific issues raised by particular individuals. Questions explored in the post course, 3 and 12 month

For peer review only

interviews will also take account of a review of information collected in medical records, via DAFNEplus technology and audio recorded follow-up sessions. Interviews will take place by telephone (unless an individual requests a face-to-face interview) at a time most convenient to the participant. All interviews will be digitally recorded with consent. It is anticipated that each interview will take 60 minutes to complete.

Facilitator interviews
Each participant's facilitators (n=1-2 per participant) will be interviewed following their close-out from the trial. If the participant received care from more than two facilitators as part of DAFNE*plus* we will ask them to nominate the two individuals from whom they felt they had the most input.

Facilitators will be interviewed once following the participant's close-out from the trial. This decision has been made partly for pragmatic reasons (i.e. we do not want to make excessive demands on the health professionals' time) and also because it will be possible to access information about the participant's care and the decisions made from the contact logs, clinical records and recordings of follow-up sessions. It is also recognised that, if facilitators are made aware that the participant is included in the process evaluation, this might influence or bias the care which is given, although participants will not be prohibited from telling their facilitators they are taking part in the qualitative research should they choose to do so.

Facilitator interviews will explore two key areas: (1) their views about, and experiences of, providing care and support to the case study participant; and (2) the facilitator's more general experiences of recruiting into the trial and delivering the DAFNE*plus* intervention.

Data collection: facilitator interviews
The facilitators' interviews will be informed by topic guides, although each individual's interview will also be tailored to explore issues specific to the participant who forms the focus of the case study (being careful to ensure that patient confidentiality is not breached). Interviews will take place by telephone at a time most convenient to the facilitator and will be digitally recorded.

1.3 Data analysis
Each participant's four interviews will be read through repeatedly and cross-compared with particular attention being paid to continuities and changes in their diabetes self-management practices over time, and the reasons for these. To aid comparison and identify where behaviour change has happened and why, 'critical incidents' will be extracted and compared (a 'critical incident' comprises data where a behaviour/decision/experience is described in detail, including the contextual and antecedent factors leading up to it and the consequences arising from it [7,8]). To help identify reasons for behaviour change, maintenance and lapses, data from the facilitator interviews, and recordings of follow-up sessions and case reports will also be used to help interpret and provide context to analysis of participant interviews.

Facilitator interviews will be cross-compared to identify issues and experiences which cut across different accounts [5]. Depending on the findings of the fidelity work, facilitator interviews may also be analysed in clusters (e.g. facilitators belonging to

For peer review only

'adherent' vs 'non-adherent' sites), to better understand reasons for individual/site differences in how the DAFNE*plus* intervention was delivered.

Key objectives of the analysis of the participant interviews are to better understand the mechanisms of impact in order to: (a) inform analysis of the quantitative data collected for the process evaluation; and (b) aid interpretation of quantitative data collected for both the process and outcomes evaluations. Key objectives of the analysis of the facilitator interviews are to offer insights which might: (a) aid interpretation of the participant case study data; (b) help explain findings from the fidelity work; (c) aid interpretation of trial outcome data; and, (d) offer insights relevant to decision-making about the possible rollout of DAFNE*plus* following the trial.

A key area for reflection

It needs to be recognised that, by interviewing participants at four time-points, and because of the kinds of questions which will be asked, the qualitative study could, potentially, have an impact on how this small group of participants understand, engage with, and experience the DAFNE*plus* intervention. Care will be made to emphasise to participants that the qualitative study is separate to DAFNE*plus* and it is our intention to understand their experiences rather than to influence their behaviours. Whilst we may not be able to diminish the impact that this has on these participants, we hope that the overall impact will be minimal due to the small sample size potentially affected.

(2) Quantitative component

2.1 Overview

A longitudinal, questionnaire study design has been adopted to determine the impact of the RCT on: a) our primary psychological outcome (diabetes-specific quality of life), b) secondary psychological outcomes and c) for the quantitative aspect of the process evaluation. That is, to identify the mechanisms of change that predict glycaemic control and diabetes-specific quality of life.

All participants in the intervention (DAFNE*plus*) and control (DAFNE) arms of the RCT will be given questionnaires to complete at baseline (up to 4 weeks prior to commencing the course) and at course completion, 3, 6, 9, 12 months post-course (see section 7 and Table 1). At baseline, the point at which participants will be more motivated to participate (pre-trial), they will be asked to complete all outcome and process questionnaire measures. To reduce participant burden, at course completion, 3- and 9-months they will only be asked to complete process measures. At 6 and 12-months they will be asked to complete the primary and secondary outcome measures only. Participants will be given the option of completing the questionnaire packs online or as a hard copy. Our choice of questionnaires (see section 7), assessing different constructs, have been selected according to existing knowledge about their association with the trial's primary outcome (HbA1c) and diabetes-specific quality of life (primary psychological outcome), the results of the YOURSAY survey (unpublished), our former work with the DAFNE intervention, and based on the theoretical framework that underpins the new intervention development work and possible treatment mechanisms [9–11]. Brevity of the questionnaires and participant burden have also been a key consideration in our rationale for selection.

2.2 Analysis

For peer review only

The use of a repeated measures, longitudinal design will permit analysis of our primary and secondary psychological outcomes, as well as both the short- and long-term predictors and mediators of outcome (HbA1c and ADDQoL-15) using Structural Equation Modelling. SEM combines confirmatory and exploratory purposes. We will test our proposed model of the long-term predictors and mediators of outcome and then, if necessary, re-test this based on changes suggested by SEM modification indices [12]. The model will partially be informed by the qualitative work (described in section 1 above).

(3) Assessment of fidelity of delivery

3.1 Introduction

Behaviour change interventions are susceptible to variation in implementation, and are not always delivered as planned [13]. Intervention fidelity refers to the methodological strategies used to assess, monitor and enhance the integrity, that is, reliability and validity of behaviour change interventions [13]. The extent to which interventions are delivered as planned indicates internal and external validity, and needs to be known if the trial results are to be accurately interpreted and replicated. If fidelity is low, it is uncertain whether a change in outcome variables is due to the intended intervention, or to unknown factors that may have been added or omitted; alternatively, if no positive change is observed, it cannot be determined whether this is due to an inefficient intervention or a lack of intervention fidelity. This means that ineffective treatments risk being implemented and disseminated, and potentially effective treatments prematurely discarded [13].

The development of the content and structure of the DAFNE*plus* programme was informed by the Behaviour Change Wheel (BCW) framework [11]. The intervention's proposed functions are served by behaviour change techniques (BCTs), specified in the hierarchical Behaviour Change Technique Taxonomy v1 (BCTTv1; [14]), which are its 'active ingredients' [15]. The DAFNE*plus* intervention contains manual-specified BCTs as its active ingredients proposed to effect behaviour change (e.g. action planning, goal setting, and information on health consequences of the behaviour), together with principles for delivery specific to the DAFNE*plus* intervention that were identified during an expert consensus process (e.g. focus on the positives, emphasise individual autonomy). The fidelity analysis will involve assessment of the delivery of BCTs.

Fidelity of delivery of BCTs will also be assessed in the control arm of the trial (standard DAFNE) in order to identify any loss of treatment differentiation between the intervention and control arms as originally designed. Potentially loss of differentiation may result from low fidelity of delivery of additional content in the DAFNEplus programme, or additional content being delivered in the standard DAFNE programme, either unintentionally or as a result of contamination.

3.2 Aims and research questions

The aim is to explore the integrity of delivery of the DAFNE*plus* programme trialled in the RCT.

The research questions are:

- To what extent was the DAFNE*plus* programme delivered as specified in the protocols (course curriculum and follow-up scripts)? Specifically:

For peer review only

- What proportion of manual-specified content (i.e. BCTs) was delivered by facilitators as intended during the programme sessions?
- What additional, non-specified BCTs were delivered by facilitators?
- How did the proportion of manual-specified content delivered differ across sessions and sites?

- What is the extent of treatment differentiation between the content of DAFNEplus and control (standard DAFNE) programmes delivered?

3.3 Methods

3.3.1 Design

A quantitative fidelity assessment, involving content analysis of intervention materials and transcripts of audio-recorded intervention sessions and provider self-rated fidelity checklists.

3.3.2 Observed fidelity of delivery assessment

The direct observation of fidelity via coding of session transcripts will provide an in-depth assessment of fidelity of delivery in a sub-sample of DAFNE*plus* and DAFNE courses.

Participants

Facilitators delivering either the DAFNE*plus* (intervention arm) or standard DAFNE (control arm) curriculum in 6 of the 14 participating sites will have their sessions recorded. It is assumed that each of these sites will have at least three facilitators delivering the DAFNE or DAFNE*plus* programmes (i.e. a minimum of 18 participants). Informed consent will be obtained from all participants at the selected sites (facilitators and patients). As part of the wider RCT., their participation in the programme during the course and follow-up sessions will be audio-recorded for training and research purposes.

Materials: Coding framework to assess observed fidelity of delivery

A coding framework will be developed to specify the BCTs to be delivered during the five face-to-face DAFNE*plus* days and the follow-up sessions, and the standard DAFNE course sessions, as specified in the facilitator manual. For each BCT the coding framework will include a definition, examples and criteria for potential operationalisation in the context of the programme.

Sampling and procedure

Six sites (2 control and 4 intervention) will be purposively sampled for audio recording of all sessions. Selection will be informed by variables such as facilitator experience, previous research activity and site activity levels.

Course sessions, and where applicable follow up sessions, delivered face-to-face, will be audio-recorded in both the intervention (i.e. DAFNE*plus*) arm and control arm (i.e. standard DAFNE) [6] at selected sites. Written informed consent for audio-recording sessions will be sought from all participants and facilitators. Participants will be reassured that transcripts of audio-recorded sessions will be fully anonymised to remove any personal or identifiable information. Facilitators will be supplied with a digital audio-recorder and instructions for operating it. Facilitators will audio-record sessions and upload recordings to the University of Sheffield secure server via Google

For peer review only

Drive which will be accessed by the study manager and authorised members of the research team. Transcription will be performed by an external transcription service and a confidentiality agreement will be put in place with the transcribers to protect participant's data.

Each DAFNE*plus* programme comprises circa 40 sessions (one 1:1 pre-course session, 35 group 'course' sessions and four 1:1 follow-up sessions per participant). Each standard DAFNE course comprises circa 35 group course sessions per participant. Sessions will therefore be purposively sampled for transcription and analysis across both arms, selected sites and courses. Courses will be sampled according to key variables, e.g. geographical location, and the timeline for the trial with earlier courses preferentially sampled due to staff resource. Sessions may be sampled for transcription according to theoretical underpinning of the intervention, and evidenced relation to the outcome.

Analysis

Sampled content of sessions in both the intervention and control arms will be first specified by applying the developed coding framework to the sample of selected course transcripts. Two researchers will independently read through the session transcript line-by-line, using the coding framework to identify and categorise BCTs present in the facilitator's speech. Each identified BCT and delivery principle will be rated as fully, partially or not delivered according to the coding framework definition and criteria. Illustrative examples will be extracted into the framework.

To assess and establish inter-coder reliability, the researchers will meet frequently in coding workshops at the outset of coding (e.g. initially after coding every transcript [16]). Approximately twenty percent of transcripts will be double coded. Inter-rater reliability will be assessed by percentage agreement [17]. Reasons for discrepancies will be discussed, and the coding framework developed accordingly. Following Hardeman et al. [16], a minimum level of 75% inter-coder agreement [18], described as 'high' [19,20] will be considered acceptable. After inter-coder reliability has been established, researchers will code the remainder of transcripts independently.

Fidelity of delivery will be assessed following the methods of Hardeman et al. [16] and Lorencatto et al. [17]. Each of the BCTs specified in the DAFNE*plus* programme (intervention arm) or standard DAFNE programme (control arm) curriculum/scripts will be listed in a checklist, together with details such as session number and facilitator participant number. The BCTs specified in the coding framework will be rated as: 1) fully present, 2) partially present, or 3) absent but should be present. The proportion of BCTs delivered as intended will be assessed by dividing the number of fully/partially present BCTs by the total number of intended BCTs. Established criteria will be applied to classify extent of observed fidelity of delivery [6]: if < 50% of intended content is delivered this will be classified as 'low' fidelity; 51-79% as 'moderate' fidelity, and 80-100%' as 'high fidelity'.

Sessions will be grouped into types based on topics where applicable (for example, the four sessions covering action planning would be grouped into one type). An 'intended content' checklist will be produced for each session, and the session transcript will only be compared to the checklist for the corresponding session or session type, rather than comparison against the full curriculum. Variation in fidelity

For peer review only

will be examined according to site and session type. Delivery of any additional content will also be examined by assessing the frequency of delivery of any non-specified BCTs. This will serve to identify adaptations made whilst delivering DAFNE*plus*.

Treatment differentiation will be assessed by comparing the content analyses of transcripts from the intervention (DAFNE*plus*) and control (standard DAFNE) sessions. BCTs that are fully/partially delivered in transcripts from both arms will be compared, and the proportion of BCTs delivered in both arms assessed, with a higher proportion of common BCTs delivered representing less treatment differentiation.

3.3.3 Self-reported fidelity of delivery

Participants

All facilitators delivering either the DAFNE*plus* (intervention arm) or standard DAFNE (control arm) curriculum/scripts in each of the 14 participating sites will provide data for the fidelity of assessment delivery. It is assumed that each site will have at least three facilitators delivering the DAFNE or DAFNE*plus* programmes (i.e. a minimum of at least 42 participants). Informed consent will be obtained from all participants (facilitators and patients) as part of the wider RCT.

Materials: Facilitator self-rated checklists

To obtain a global snapshot of fidelity across all DAFNE*plus* courses, including those that are not transcribed and included in the observed fidelity assessment, self-reported facilitator checklists will also be developed and administered to all sites (intervention and control). The checklist will include provision of key information and BCTs that are intended to be delivered (i.e. as specified in the pre-course session script, course curriculum and follow-up support scripts), and how confident and competent the facilitators felt delivering the session components. Facilitators will also be asked to record reasons for any components not being fully delivered. Different checklists will be developed for each session. Due to the dynamic nature of the intervention and curriculum development it is not possible to provide definitive and finalised versions of these checklists at this time: the checklists will be finalised following the coding of the final version of DAFNEplus[2].

Procedure

Facilitators will be asked to complete the checklist at the end of each session where possible, or by the end of each day. They will forward completed checklists to Sheffield University CTRU by the end of each day. Facilitators will rate the extent to which they feel they delivered the intervention components listed in the checklists, from 0 (not at all), 1 (partially) to 2 (fully delivered).

Analysis

The proportion of intended components rated as partially/fully delivered by the facilitators will be calculated. The same criteria will be applied to classify extent of fidelity as in the observed measurements: if < 50% of intended content is delivered

[2] We are submitting specimen checklists with this revised version of the protocol, these checklists are subject to change as detailed above. The curriculum will be subject to change up until the point of recruitment and even after this point there might be minor changes which would require modification of the fidelity checklists. A requirement to submit these checklists after each change would be a major burden on both the ethics committee and the research team and therefore we seek permission to revise checklists without further approval and will not submit additional checklists unless requested to do so.

For peer review only

this will be classified as 'low' fidelity; 51-79% as 'moderate' fidelity, and 80-100%' as 'high fidelity'. Variation in proportion of fidelity of delivery will be examined across: session types, facilitators, and courses.

There are well documented discrepancies between what healthcare providers report delivering and actually deliver [21]. Therefore, for the DAFNE*plus* courses where session transcripts have also been coded (as described above), self-reported and objectively verified practice will be directly compared in terms of the proportion of BCTs facilitators report delivering, and that which was identified during the content analysis.

References

1 Moore G, Audrey S, Barker M, *et al.* Process evaluation of complex interventions UK Medical Research Council (MRC) guidance. https://www.mrc.ac.uk/documents/pdf/mrc-phsrn-process-evaluation-guidance-final/ (accessed 30 Jan 2018).

2 Moore GF, Audrey S, Barker M, *et al.* Process evaluation of complex interventions: Medical Research Council guidance. *BMJ* 2015;**350**:h1258. doi:10.1136/bmj.h1258

3 May CR, Mair F, Finch T, *et al.* Development of a theory of implementation and integration: Normalization Process Theory. *Implement Sci* 2009;**4**:29. doi:10.1186/1748-5908-4-29

4 May C, Finch T. Implementing, Embedding, and Integrating Practices: An Outline of Normalization Process Theory. *Sociology* 2009;**43**:535–54. doi:10.1177/0038038509103208

5 Strauss AL, Corbin JM. *Basics of qualitative research : grounded theory procedures and techniques*. Sage Publications 1990.

6 Borrelli B. The assessment, monitoring, and enhancement of treatment fidelity in public health clinical trials. *J Public Health Dent* 2011;**71**:S52–63. doi:10.1111/j.1752-7325.2011.00233.x

7 Flanagan JC. Psychological Bulletin THE CRITICAL INCIDENT TECHNIQUE. *JULY* 1954;**51**.https://books.apa.org/pubs/databases/psycinfo/cit-article.pdf (accessed 30 Jan 2018).

8 Butterfield LD, Borgen WA, Amundson NE, *et al.* Fifty years of the critical incident technique: 1954-2004 and beyond. *Qual Res* 2005;**5**:475–97. doi:10.1177/1468794105056924

9 Cooke D, Bond R, Lawton J, *et al.* Modeling predictors of changes in glycemic control and diabetes-specific quality of life amongst adults with type 1 diabetes 1 year after structured education in flexible, intensive insulin therapy. *J Behav Med* 2015;**38**:817–29. doi:10.1007/s10865-015-9649-y

10 Michie S, Johnston M, Abraham C, *et al.* Making psychological theory useful for implementing evidence based practice: a consensus approach. *Qual Saf Heal Care* 2005;**14**:26–33. doi:10.1136/qshc.2004.011155

11 Michie S, van Stralen MM, West R. The behaviour change wheel: a new method for characterising and designing behaviour change interventions. *Implement Sci* 2011;**6**:42. doi:10.1186/1748-5908-6-42

12 Bentler PM. On tests and indices for evaluating structural models. *Pers Individ Dif* 2007;**42**:825–9. doi:10.1016/J.PAID.2006.09.024

13 Bellg AJ, Borrelli B, Resnick B, *et al.* Enhancing Treatment Fidelity in Health Behavior Change Studies: Best Practices and Recommendations From the NIH Behavior Change Consortium. *Heal Psychol* 2004;**23**:443–51. doi:10.1037/0278-6133.23.5.443

14 Michie S, Richardson M, Johnston M, *et al.* The Behavior Change Technique Taxonomy (v1) of 93 Hierarchically Clustered Techniques: Building an International Consensus for the Reporting of Behavior Change Interventions. *Ann Behav Med* 2013;**46**:81–95.

For peer review only

doi:10.1007/s12160-013-9486-6

15 Michie, S, Atkins, L, West R. *The behaviour change wheel: a guide to designing interventions*. London: : Silverback Publishing 2014.

16 Hardeman W, Michie S, Fanshawe T, *et al.* Fidelity of delivery of a physical activity intervention: Predictors and consequences. *Psychol Health* 2008;**23**:11–24. doi:10.1080/08870440701615948

17 Lorencatto F, West R, Bruguera C, *et al.* A method for assessing fidelity of delivery of telephone behavioral support for smoking cessation. *J Consult Clin Psychol* 2014;**82**:482–91. doi:10.1037/a0035149

18 Lombard M, Snyder-Duch J. Content Analysis in Mass Communication Assessment and Reporting of Intercoder Reliability. *Hum Commun Res* 2002;**28**:587–604.http://marketing.univie.ac.at/fileadmin/user_upload/lehrstuhl_marketing/Lehre/Lehrinhalte/09SS/Lombard_et_al__2002_-1.pdf (accessed 30 Jan 2018).

19 Cohen J. Weighted kappa: nominal scale agreement with provision for scaled disagreement or partial credit. *Psychol Bull* 1968;**70**:213–20.http://www.ncbi.nlm.nih.gov/pubmed/19673146 (accessed 30 Jan 2018).

20 Altman DG. *Practical statistics for medical research*. Chapman and Hall 1991.

21 Hogue A, Dauber S, Lichvar E, *et al.* Validity of Therapist Self-Report Ratings of Fidelity to Evidence-Based Practices for Adolescent Behavior Problems: Correspondence between Therapists and Observers. *Adm Policy Ment Heal Ment Heal Serv Res* 2015;**42**:229–43. doi:10.1007/s10488-014-0548-2

For peer review only

For peer review only

Supplementary material 3 – Protocol for DAFNE*plus* Economic Evaluation

Aims and perspective

We will complete an economic evaluation as part of the study so that we are able to understand the cost-effectiveness of DAFNE*plus* compared to the standard DAFNE programme. The economic evaluation will follow guidance set by the National Institute for Health and Clinical Excellence for its Technology Appraisal process [1]. The analysis will take an NHS and personal social services perspective, measure health effects in quality adjusted life years (QALYs), discount future outcomes at 3.5% per annum and consider effects and costs over a lifetime time horizon. The primary analysis will be use long-term cost-effectiveness modelling, a secondary analysis will be an economic evaluation alongside the clinical trial (EEACT). The analysis population for all health economic analyses will consist of all participants in the DAFNEplus trial. A full Health Economic and Decision Modelling Analysis Plan (HEDMAP) will be written and circulated to the Trial Management Group and Programme Steering Committee before being signed-off.

Long-term cost-effectiveness modelling

In the long-term modelling exercise, the resulting evidence base will be incorporated into an updated Sheffield T1D Diabetes Policy Model [2]. This model has been used extensively in the evaluation of education and psychological interventions for people with T1D[3–6]. The time horizon of this analysis will be over each simulated individual's lifetime. As such, the long-term modelling will be considered as the primary health economic analysis. Demographic variables and some key resource use data (e.g. insulin use, contacts with NHS professionals) will be obtained from the trial data. The Sheffield T1D Diabetes Policy Model will be updated to use statistical models that estimate the clinical effects of DAFNE*plus* compared to DAFNE on HbA1c, the incidence of severe hypoglycaemia and the incidence of DKA. Two long-term modelling analyses will be conducted, the first will use the data collected by the one-year time point and will be submitted as part of the report to the NIHR on the DAFNE*plus* programme grant. This analysis will be updated after the two-year data collection is complete to incorporate the statistical analysis of the two-year follow up data. These statistical analyses of the clinical effects of DAFNE*plus* compared to DAFNE will be pre-specified in either the statistical analysis plan or the HEDMAP. The reporting of this evaluation will follow the Palmer *et al*[7] checklist for the reporting of model inputs to diabetes health economic studies.

Economic evaluation alongside the clinical trial

For the EEACT, we conduct the analysis in line with Ramsey *et al's* [8] recommendations for cost-effectiveness analysis alongside clinical trials. Specifically, we will collect data alongside the trial on intervention costs, associated healthcare resource use and a preference based utility measure: the EQ-5D-5L measure [9]. The intervention costing process will include training of educators, resource use, and adherence to structured follow up appointments, professional staff time and the technology component. A standard self-reported resource use questionnaire, used

previously in the DAFNE*plus* pilot (as well as the 5x1 DAFNE [10] and the REPOSE trials [11]), will ascertain NHS usage in terms of GP, community, outpatient, A&E and inpatients, as well as occurrence of DKA and hypoglycaemic events by level of severity. Unit costs will be taken from standard sources (NHS Reference Costs, British National Formulary, PSSRU). The standard self-reported resource use questionnaire and the EQ-5D-5L will be collected at baseline, 6 months and 12 months. Course costs (administrative and clinical) will be estimated using a bespoke questionnaire for completion by site staff. Our primary analysis will use the EQ-5D-5L valuation study to generate utility scores at baseline, course completion, 6 months and 12 months for each study participant [12]. There are on-going discussions about the valuation of the EQ-5D-5L, and NICE recently produced a position statement recommending that EQ-5D-5L data should be valued using mapping to the EQ-5D-3L and not the bespoke EQ-5D-5L value set [13,14]. Therefore our primary analysis will follow the most recent NICE guidance at the time of analysis, with the other valuation method been used in a sensitivity analysis. QALYs for each participant will be estimated by calculating the area under the curve defined by EQ-5D utility score, mortality and length of follow-up. The base case analysis will use the complete case data. In a scenario analysis, the missing data will be imputed. The time horizon of this analysis will be limited to the one-year time horizon of the trial. This evaluation will be considered as the secondary health economic analysis for two reasons: 1) The effects and costs of DAFNE*plus* may be incurred beyond the one-year trial time horizon (due to expected differences in the time to onset of diabetes related complications and potential maintenance of treatment effects beyond the trial period); and, 2) the DAFNEplus trial is not powered to detect differences in the incidence of long-term diabetes complications, as such the estimates of differences in the cost and QALYs between the two trial arms may be misleading.

Outcome measures and uncertainty analyses

In both the EEACT and the long term modelling the main outcome of interest will be the comparison of the incremental cost-effectiveness ratio (ICER) of DAFNE*plus* compared to DAFNE. The ICER will be compared to a maximum acceptable ICER of £20,000 per QALY gained, as this is the lower limit of the ICER range used by NICE to determine if an intervention is cost-effective [1]. Uncertainty in the ICER will be determined using: scenario analyses, subgroup analyses (pre-specified with the wider DAFNE*plus* team), probabilistic sensitivity analysis and expected value of information calculations. In particular, uncertainty in the cost-effectiveness of DAFNE*plus* as used in a wider rollout (compared to as utilised in the trial) and in subgroups of participants with a HbA1c less than 7.5% and greater than or equal to 7.5% will be explored in our scenario analyses.

References

1 National Institute for Health and Care Excellence. Guide to the methods of technology appraisal 2013 | Guidance and guidelines | NICE. https://www.nice.org.uk/process/pmg9/chapter/foreword (accessed 30 Jan 2018).

2 Thokala P, Kruger J, Brennan A, *et al.* Assessing the cost-effectiveness of Type 1 diabetes interventions: the Sheffield Type 1 Diabetes Policy Model. *Diabet Med* 2014;**31**:477–86. doi:10.1111/dme.12371

For peer review only - http://bmjopen.bmj.com/site/about/guidelines.xhtml

3 Kruger J, Brennan A, Thokala P, *et al.* The cost-effectiveness of the Dose Adjustment for Normal Eating (DAFNE) structured education programme: an update using the Sheffield Type 1 Diabetes Policy Model. *Diabet Med* 2013;**30**:1236–44. doi:10.1111/dme.12270

4 Heller S, Lawton J, Amiel S, *et al.* Improving management of type 1 diabetes in the UK: the Dose Adjustment For Normal Eating (DAFNE) programme as a research test-bed. A mixed-method analysis of the barriers to and facilitators of successful diabetes self-management, a health economic analysis, a cluster randomised controlled trial of different models of delivery of an educational intervention and the potential of insulin pumps and additional educator input to improve outcomes. *Program Grants Appl Res* 2014;**2**:1–188. doi:10.3310/pgfar02050

5 Kruger J, Pollard D, Basarir H, *et al.* Incorporating Psychological Predictors of Treatment Response into Health Economic Simulation Models. *Med Decis Mak* 2015;**35**:872–87. doi:10.1177/0272989X15590143

6 Pollard DJ, Brennan A, Dixon S, *et al.* Cost-effectiveness of insulin pumps compared with multiple daily injections both provided with structured education for adults with type 1 diabetes: A health economic analysis of the Relative Effectiveness of Pumps over Structured Education (REPOSE) randomised controlled trial. *BMJ Open* 2018;**8**:e016766. doi:10.1136/bmjopen-2017-016766

7 Palmer AJ, Si L, Tew M, *et al.* Computer Modeling of Diabetes and Its Transparency: A Report on the Eighth Mount Hood Challenge. *Value Heal* 2018;**21**:724–31. doi:10.1016/j.jval.2018.02.002

8 Ramsey SD, Willke RJ, Glick H, *et al.* Cost-Effectiveness Analysis Alongside Clinical Trials II—An ISPOR Good Research Practices Task Force Report. *Value Heal* 2015;**18**:161–72. doi:10.1016/j.jval.2015.02.001

9 Herdman M, Gudex C, Lloyd A, *et al.* Development and preliminary testing of the new five-level version of EQ-5D (EQ-5D-5L). *Qual Life Res* 2011;**20**:1727–36. doi:10.1007/s11136-011-9903-x

10 Elliott J, Lawton J, Rankin D, *et al.* The 5x1 DAFNE study protocol: a cluster randomised trial comparing a standard 5 day DAFNE course delivered over 1 week against DAFNE training delivered over 1 day a week for 5 consecutive weeks. *BMC Endocr Disord* 2012;**12**:28. doi:10.1186/1472-6823-12-28

11 REPOSE Study Group TRS. Relative effectiveness of insulin pump treatment over multiple daily injections and structured education during flexible intensive insulin treatment for type 1 diabetes: cluster randomised trial (REPOSE). *BMJ* 2017;**356**:j1285. doi:10.1136/BMJ.J1285

12 Devlin NJ, Shah KK, Feng Y, *et al.* Valuing health-related quality of life: An EQ-5D-5L value set for England. *Health Econ* Published Online First: 22 August 2017. doi:10.1002/hec.3564

13 National Institute for Health and Care Excellence. NICE's position statement on EQ-5D-5L NATIONAL INSTITUTE FOR HEALTH AND CARE EXCELLENCE Position statement on use of the EQ-5D-5L valuation set.

For peer review only

https://www.nice.org.uk/Media/Default/About/what-we-do/NICE-guidance/NICE-technology-appraisal-guidance/eq5d5l_nice_position_statement.pdf (accessed 30 Jan 2018).

14 van Hout B, Janssen MF, Feng Y-S, *et al.* Interim Scoring for the EQ-5D-5L: Mapping the EQ-5D-5L to EQ-5D-3L Value Sets. *Value Heal* 2012;**15**:708–15. doi:10.1016/j.jval.2012.02.008

For peer review only

Supplementary material 4 – Development and modification of questionnaires and individual items

Confidence in Diabetes Scale (CIDS)

This scale assesses the extent to which an individual believes they can engage in particular behaviours related to their type 1 diabetes treatment regimen (self-efficacy). The scale was published in 2003 and Dr Cooke contacted Prof Snoek, the senior author on the original validation paper to seek permission to amend item 3 on the scale (*I believe I can perform the prescribed number of daily insulin injections*). He gave his permission for the team to make the following amendment to reflect the more flexible approach to multiple daily insulin treatment regimens, advocated by courses like DAFNE and DAFNE*plus* (*I believe I can perform the number of daily insulin injections I need to*).

Self-Regulation Questionnaire for Type 1 Diabetes (SRQ-T1D)

This questionnaire is an adaptation of the Self-Regulation Questionnaire [1]. Self-regulation is the ability to develop, implement, and flexibly maintain planned behaviour in order to achieve one's goals. The SRQ and our adaptation of this builds on the work of Frederick Kanfer and two researchers who formulated a seven-step model of self-regulation [2,3]. Although this model was developed specifically to study addictive behaviours, the self-regulatory processes it describes are meant to be general principles of behavioural self-control. In this model, people may have problems managing certain behaviours (behavioural self-regulation) because of challenges at any of these seven steps:

1. **Receiving** relevant information
2. **Evaluating** the information and comparing it to norms
3. **Triggering** change
4. **Searching** for options
5. **Formulating** a plan
6. **Implementing** the plan
7. **Assessing** the plan's effectiveness (which recycles to steps 1 and 2)

The original SRQ has demonstrated reliability, concurrent and discriminant validity in community samples[4]. It consists of 63 items which was too long for our team to use in the DAFNE*plus* questionnaire pack, when this is one of several process measures. This measure was reviewed by 3 members from the DAFNE*plus* PPI group and by our process evaluation team consisting of clinicians, behavioural scientists, psychologists and social anthropologists, two of whom also have type 1 diabetes. The PPI group strongly recommended altering the wording of the individual items slightly so that these were all framed to be diabetes-specific in focus, rather than generic. Dr Cooke, in discussion with two members of the PPI group amended the wording of some of these items to ensure that they were clear and made sense. The process evaluation team and PPI group selected their top 2-3 items from each of the seven categories (above), rank ordering them. Dr Cooke then reviewed these to select the items from each

For peer review only

category which the majority had agreed should be included within the final questionnaire.

Beliefs about Consequences of Diabetes Self-Care Behaviours; Diabetes Support and Routines

The DAFNE*plus* revisions to the original DAFNE curriculum were structured around the Theoretical Domains Framework[5] hence it is very important to the process evaluation team to assess the constructs that are being targeted within individuals through the content and delivery of the DAFNE*plus* course; to assess whether participants in the DAFNE*plus* and standard DAFNE groups respond differently on these measures but also whether these constructs explain any differences in outcomes (HbA1c and diabetes-specific quality of life). Three of these constructs are 'social influences', 'beliefs about consequences of diabetes self-care' and 'environmental cues and prompts'. The research team have generated 11 diabetes-specific items to assess these constructs and have piloted them with our PPI group. These are unvalidated but once we have collected data at two timepoints (course completion and 3-months follow-up), if these measures are shown not to be psychometrically robust, we will remove these items from the 9-month follow-up point. Please note that we are only collecting these process measures at 3 timepoints.

References

1 Brown J, Miller W, LewandowskiLA. The Self-Regulation Questionnaire. In: Creek, Van de L, Jackson T, eds. *Innovations in clinical practice: A source book*. Sarasota, Florida: : Professional Resource Press 1999. 281–9.
2 Brown JM. Self-Regulation and the Addictive Behaviors. In: *Treating Addictive Behaviors*. Springer US 1998. 61–73. doi:10.1007/978-1-4899-1934-2_5
3 Miller W, Brown J. Self-Regulation as a Conceptual Basis for the Prevention and Treatment of Addictive Behaviours. In: Heather N, Miller W, Greenley J, eds. *Self-Control and the Addictive Behaviours*. Sydney: : Maxwell MacMillan Publishing Australia 1991. 3–79.
4 Aubrey L, Brown J, Research WM-AC& E, *et al.* Psychometric properties of a selfregulation questionnaire (SRQ). *Alcohol Clin Exp Res* 1994;**18**:429.
5 Atkins L, Francis J, Islam R, *et al.* A guide to using the Theoretical Domains Framework of behaviour change to investigate implementation problems. *Implement Sci* 2017;**12**:77. doi:10.1186/s13012-017-0605-9

For peer review only

Supplementary Material 5 – DAFNE*plus* SAP

Project Title: A Cluster Randomised Controlled Trial (CRCT) of the DAFNE*plus* (Dose for Adjustment for Normal Eating) intervention: A lifelong approach to promote effective self-management in adults with type 1 diabetes

Statistical Analysis Plan

Version 1 17th December 2019

Based on Protocol Version 6.0, dated 9th September 2019

REC: 18/SW/0100 ISRCTN: 42908016 Sheffield CTRU Job no. J13-003

Authored by

________________________________ ___/___/____

Nikki Totton Date

Statistician, CTRU, University of Sheffield

Approved by

________________________________ ___/___/_____

Mike Bradburn Date

Senior Statistician, CTRU, University of Sheffield

________________________________ ___/___/_____

Simon Heller Date

Chief Investigator, Sheffield Teaching Hospitals NHS Foundation Trust

________________________________ ___/___/_____

Simon Griffin Date

DAFNE*plus* TSC Chair, University of Cambridge

Roles and Responsibilities

This SAP was initially drafted by Henry Nanji (previous DAFNE*plus* trial statistician) and updated by Nikki Totton (DAFNE*plus* trial statistician), with input from Mike Bradburn (senior statistician) and the trial team.

SAP Revision History

Version Number	Revision Date	Timing Within Trial	Description/Justification

For peer review only

For peer review only - http://bmjopen.bmj.com/site/about/guidelines.xhtml

List of Abbreviations and Definitions of Terms

**ADDQoL-14**	Audit of Diabetes Dependent Quality of Life (14 items)
**AE**	Adverse Event
**BMI**	Body Mass Index
**CIDS**	Confidence in Diabetes Scale
**CRF**	Case Report Form
**CTRU**	Clinical Trials Research Unit
**DAFNE**	Dose Adjustment For Normal Eating
**DIDP**	DAWN Impact of Diabetes Profile
**DKA**	Diabetic Ketoacidosis
**DME-Q**	Diabetes Management Experience Questionnaire
**DSRQ**	Diabetes Strengths and Resilience Questionnaire
**eGFR**	Estimated Glomerular Filtration Rate
**EQ-5D-5L**	EuroQol - 5 Dimensions - 5 Levels
**GCP**	Good Clinical Practice
**GLM**	Generalised Linear Model
**HASMID**	Health And Self-Management In Diabetes
**HbA1c**	Glycated Haemoglobin (Haemoglobin A1c)
**HCS**	Hypoglycaemia Confidence Scale
**HDL**	High-Density Lipoprotein
**HFS**	Hypoglycaemia Fear Survey
**ICC**	Intra-class Correlation Coefficient
**IQR**	Inter Quartile Range
**IRR**	Incidence Rate Ratio
**ISRCTN**	International Standard Randomised Controlled Trial Number
**ITT**	Intention To Treat
**LDL**	Low-Density Lipoprotein
**LRT**	Likelihood Ratio Test
**MD**	Mean Difference
**MDI**	Multiple Daily Injections
**NHS**	National Health Service
**OR**	Odds Ratio
**PAID**	Problem Areas In Diabetes
**PP**	Per Protocol
**QoL**	Quality of Life
**RCT**	Randomised Controlled Trial
**REC**	Research Ethics Committee
**SAE**	Serious Adverse Event
**SCB-T1D**	Self-Care Behaviours: Type 1 Diabetes
**SD**	Standard Deviation
**SOP**	Standard Operating Procedure
**SRQ-T1D**	Self-Regulation Questionnaire
**SUS**	System Usability Score
**T1D**	Type 1 Diabetes
**TMG**	Trial Management Group
**TSC**	Trial Steering Committee
**VAS**	Visual Analogue Scale
**W-BQ28**	Well-Being Questionnaire 28

For peer review only - http://bmjopen.bmj.com/site/about/guidelines.xhtml

Contents

1 Introduction ... 4
1.1 Background and Rationale ... 4
1.2 Objectives ... 4
2 Trial Methods ... 5
2.1 Trial Design ... 5
2.2 Randomisation and Blinding ... 5
2.3 Sample Size ... 5
2.4 Trial Framework ... 6
2.5 Trial Monitoring and Management ... 6
2.6 Interim Analysis and Stopping Rules ... 6
2.7 Timing of Final Analysis ... 6
2.8 Timing of Outcome Assessments ... 7
3 Statistical principles ... 8
3.1 Confidence Intervals and P Values ... 8
3.2 Adherence and Protocol Deviations ... 8
3.3 Analysis Populations ... 8
4 Screening, recruitment, demographics and withdrawal ... 9
4.1 Eligibility Criteria ... 9
4.2 CONSORT ... 10
4.3 Withdrawal of Participants ... 11
4.4 Baseline Characteristics ... 11
5 Outline of Statistical AnalysIs ... 13
5.1 Outcome Measures ... 13
5.1.1 Primary Outcome ... 13
5.1.2 Secondary Outcomes - Biomedical ... 13
5.1.3 Secondary Outcomes - Psychological ... 14
5.1.4 Secondary Outcomes - Process Measures ... 15
5.2 Analysis of Primary Outcome ... 16
5.2.1 Model Checking ... 17
5.3 Sensitivity Analyses of the Primary Outcome ... 17
5.4 Subgroup Analyses ... 17
5.5 Handling Missing Data ... 18
5.6 Analysis of Secondary Outcomes - Biomedical ... 19
5.6.1 HbA1c ... 19
5.6.2 Weight, BMI, Blood Pressure and Lipids ... 19
5.6.3 Severe Hypoglycaemia and DKA ... 19
5.6.4 Model Checking ... 20
5.7 Analysis of Secondary Outcomes - Psychological ... 20
5.8 Safety Outcomes ... 20
5.9 Statistical Software ... 20
6 References ... 21
6.1 Documents ... 21
6.2 Publications ... 21

For peer review only - http://bmjopen.bmj.com/site/about/guidelines.xhtml

1 INTRODUCTION

1.1 Background and Rationale

The successful management of Type 1 Diabetes (T1D) requires those affected (>300,000 adults in the UK) [1] to keep their glucose levels sufficiently close to normal to avoid long-term complications [2]. In this condition, unlike type 2 diabetes, there is an absolute insulin deficiency, and so insulin must be injected subcutaneously, and tablet therapy is not possible. Preventing complications depends upon an individual's ability to prevent hyperglycaemia (high blood glucose levels) by self-managing their condition. This is done by calculating precise insulin doses based on accurate estimations of food intake before every meal using frequent blood glucose measurements, and accounting for fluctuations in physical activity, illness, stress and hormones. Hypoglycaemia (low blood glucose levels), if severe, can result in acute cognitive impairment, confusion, collapse and injury, coma or even death [3].

Dose Adjustment For Normal Eating (DAFNE) is a clinical education programme run within the National Health Service (NHS), designed to teach and improve self-management skills in flexible intensive insulin therapy to improve both glucose control and quality of life in adults with T1D. It is a five-day training course for adults with T1D, delivered in small groups. The DAFNE*plus* programme grant has modified the existing DAFNE curriculum to incorporate techniques for initiating and sustaining behaviour change, structured follow-up support, and digital information communication technology.

1.2 Objectives

The primary objective of the trial is to:

1. Assess the effects of the intervention on glycaemic control as measured by glycated haemoglobin (HbA1c) at 12 months.

The secondary objectives of the trial are to:

1. Assess the medium term effect of the intervention on glycaemic control, as measured by HbA1c, using data at 6 months,
2. Assess the effects of the intervention on the diabetes-specific quality of life,
3. Assess the effects of the intervention on diabetes distress and other biomedical outcomes (severe episodes of hypoglycaemia, diabetic ketoacidosis, weight, body mass index, blood pressure and lipids),
4. Undertake a mixed methods process evaluation to aid understanding of the Randomised Controlled Trial (RCT) findings, and to inform decision making about the implementation of DAFNE*plus* in clinical care post-trial,
5. Assess fidelity of delivery of the DAFNE*plus* intervention,
6. Undertake a health economic analysis to determine the cost-effectiveness of DAFNE*plus* versus standard DAFNE.

Objectives 4, 5 and 6 under secondary objectives will not be considered as part of this SAP and will be dealt with separately to the main trial analysis.

For peer review only - http://bmjopen.bmj.com/site/about/guidelines.xhtml

2 TRIAL METHODS

2.1 Trial Design

The trial will use a pragmatic, parallel group, cluster randomised (1:1 allocation) controlled design involving 14 sites. Centre randomisation is required rather than individual since 'contamination' of the control arm may occur if educators are trained in DAFNE*plus* (intervention) and still are required to deliver standard DAFNE (control) [4]. Potential participants are identified by local diabetes clinicians and will use standard criteria for referral to DAFNE.

Participants recruited at control centres will receive treatment as usual and will attend the DAFNE course one day a week, over five consecutive weeks. A bolus calculator will be provided to support the calculation of insulin dose, but there will be no structured follow-up appointment beyond those provided in usual care.

Participants in centres allocated to the intervention will attend the DAFNE*plus* course one day a week, over five consecutive weeks, which includes the use of technology to transmit and display blood glucose data to support pattern recognition and interpretation. A bolus calculator to support insulin dose calculations will be provided and up to five structured follow-up appointments are offered in the 12 months after the course.

Further details on the trial design can be found in the protocol.

2.2 Randomisation and Blinding

Following ethical approval, all participating centres were randomised on a 1:1 basis to control (standard DAFNE courses) or the intervention arm (DAFNE*plus* course). In order to balance the centres within the two arms, a covariate constrained approach [5] was adopted matching the centres on the number of patients, number of educators and the total number of previous DAFNE courses delivered by the centre as stratification variables. Due to the nature of the intervention, The University of Sheffield Clinical Trials Research Unit (CTRU) in-house randomisation system (SCRAM) was not applicable and so in line with Standard Operating Procedure (SOP) ST007, a randomisation guidance document detailing the randomisation procedure has been written. The random allocation was conducted by the trial statistician using STATA [6] and therefore no outcome data which is split by treatment group will be seen by the statistician until the trial is complete to minimise bias. Further details on the randomisation and unblinding can be found in the protocol.

2.3 Sample Size

2.3.1 Original Sample Size

It is expected that there will be 882 patients referred for DAFNE courses within the 15-month recruitment window and of these, it is expected that 75% (662 patients) will be recruited, equivalent to 47 participants at each of the 14 centres. Based on data from current DAFNE courses, a further 25% are expected not to meet the primary analysis population criteria of a baseline HbA1c greater than 7.5%, leaving 497 participants. Finally, we anticipate 15% of participants to be lost to follow-up by the 12-month stage, therefore giving a primary analysis population of 422 patients. Taking into account a

For peer review only - http://bmjopen.bmj.com/site/about/guidelines.xhtml

design effect, due to the cluster design of the trial, with an Intra-class Correlation Coefficient (ICC) of 1.5% (from previous DAFNE data) and 30 patients per cluster (422 patients over 14 centres) the design effect is 1.435 leaving the effective total sample size of 294 participants (147 per arm).

Using a two-sample comparison of mean HbA1c at the 12-month follow-up with 2-sided alpha of 5%, a correlation of 0.5 between baseline and final values and a standard deviation of 1.45 (from previous DAFNE data), the trial sample gives 92% power to detect a 0.5% difference in HbA1c between the two treatment groups.

2.3.2 Updated Sample Size

The original calculations (i.e. worked backwards from the expected number of recruits) gave a power of 92.7%. The team discussed different options, but in light of the difficulties in enlisting new centres and therefore now 13 centres not 14, it was agreed by our Trial Steering Committee to continue with the original planned recruitment per centre, with 6 interventions and 7 control. Therefore, reducing the sample size solely in the intervention arm. This results in a power of 90.4% with a small imbalance between the two arms (ratio 1:1.67) with a reduced sample size of 615 (instead of 662). As this trial is cluster randomised and provided through courses, there was always likely to be some imbalance between the two treatment arms which is out of our control.

2.4 Trial Framework

The primary aim of this trial is to conduct a superiority cluster RCT comparing the new DAFNE*plus* intervention to the existing DAFNE to detect a minimum clinically significant difference of 0.5% in HbA1c between the two groups after 12 months.

2.5 Trial Monitoring and Management

In compliance with Sheffield CTRU's SOPs, the following committees will be established to govern the overall conduct and supervision of the trial:

- Trial Steering Committee (TSC)
- Trial Management Group (TMG)

The trial will be supervised on a day to day basis at Sheffield CTRU by the Trial Manager with supervision from the Chief Investigator and a Senior Trial Manager.

2.6 Interim Analysis and Stopping Rules

There are no interim analyses or early stopping planned for this trial, hence no stopping rules are applicable.

2.7 Timing of Final Analysis

The final analysis will take place after the last participants have completed their 12-month follow-up visit. All data will be analysed collectively at this time point. A further study will be completed in the future to complete analysis of data collected at the 24-month follow-up, but this is not included within this SAP and will be outlined separately.

For peer review only - http://bmjopen.bmj.com/site/about/guidelines.xhtml

2.8 Timing of Outcome Assessments

Table 1 below shows the biomedical and psychological outcome measures and the different time-points outcomes are measured. A detailed description of the outcome assessment is found under section 5.

Table 1: Outcomes measures within the trial and time points they will be collected at

Outcome Measure	Baseline	6 Months Post Course	12 Months Post Course
**Clinical Outcomes**			
Demographics[1]	x		
HbA1c	x	x	X
Severe Hypoglycaemic Episodes	x	x	x
Diabetic Ketoacidosis (DKA) Episodes	x	x	x
Body Mass Index (BMI)	x	x	x
Blood Pressure	x		x
Lipids (HDL, LDL)[2]	x		x
**Psychological Outcomes**			
ADDQoL-15 (Diabetes-specific quality of life)	x	x	x
DIDP (Diabetes-specific quality of life)	x	x	x
PAID-11 (Diabetes Distress)	x	x	x
W-BQ28 (Diabetes-Specific Wellbeing)	x	x	x
HFS-11 (Fear of Hypoglycaemia)	x	x	x
Gold Score (Hypoglycaemia awareness)	x	x	x
**Health Economic Measures**			
HASMID (Health Status)	x	x	x
EQ-5D-5L (Health Status)	x	x	x
**Process Measures**			
DSRQ	x		
SCB-T1D	x		
Usability Score			
DME-Q	x		
HCS	x		
Beliefs about Consequences	x		
SRQ-T1D	x		

1: Detail description of demographic characteristics are found under section 4.4

For peer review only - http://bmjopen.bmj.com/site/about/guidelines.xhtml

2: HDL=High Density Lipoprotein, LDL=Low Density Lipoprotein

3 STATISTICAL PRINCIPLES

This statistical analysis plan (SAP) is written in conjunction with the International Conference of Harmonisation topic E9 [7], applicable Standard Operating Procedures (SOP) from the Sheffield Clinical Trials Research Unit (CTRU) (ST001 and ST006).

3.1 Confidence Intervals and P Values

All statistical tests will be completed at the 5% significance level and estimates of the treatment effect will be reported with their associated 95% confidence intervals. All tests completed will be two-sided. The results of the trial are focussed on the primary endpoint (HbA1c at 12 months) so adjustment for multiple testing and control of the type 1 error rate is required.

3.2 Adherence and Protocol Deviations

Adherence to the standard DAFNE course within the control group is defined as:

- Attending a minimum of four of the five days within the course which must include the first two days.

Adherence to the DAFNE*plus* course within the intervention group is defined as:

- Attending a minimum of four of the five days within the course which must include the first two days *AND*
- Attending at least three of the five follow-up sessions (this can be any three sessions)

Adherence to the courses will be presented as the number and percentage of participants in each arm of those that adhered. Additionally, for the intervention group the number and percentage of participants that adhered to each of the two adherence requirements will be presented separately to show which, if either, of these are more prominent.

In the DAFNE*plus* trial, any intended failure to adhere to the protocol will be classed as protocol violation and may be minor or major while any unintended (non-serious) departures from the protocol would be considered as protocol deviations and all these will be reported.

Attendance will be captured on case report forms (CRFs) when participants attend the course. Participants who failed to meet this criterion will be classed as having a major protocol deviation.

The number (and percentage) of patients with major and minor protocol deviations will be summarised by treatment group with details of type of deviation provided. No formal statistical testing will be undertaken between the two groups.

3.3 Analysis Populations

The primary analysis set will be that defined in Intention To Treat (ITT) on the primary outcome. Additional analysis populations, such as Per Protocol (PP), will be used as sensitivity analyses. Table 2 defines each of the analysis sets.

For peer review only - http://bmjopen.bmj.com/site/about/guidelines.xhtml

Table 2 Definition of the analysis set

Analysis Set	Outcomes	Participant Inclusion Criteria
**Primary ITT**	Primary outcome only	All consented participants, analysed according to their centre's randomisation regardless of their adherence to the entry criteria, intervention received, subsequent withdrawal or deviation from the protocol unless they have explicitly requested that their data be removed [7]. In addition, participants must have a baseline HbA1c more than 7.5%.
**Full ITT**	Secondary outcomes and as a sensitivity analysis for the primary outcome	All consented participants, analysed according to their centre's randomisation regardless of their adherence to the entry criteria, intervention received, subsequent withdrawal or deviation from the protocol unless they have explicitly requested that their data be removed [7].
**PP**	Primary outcome only	All consented participants excluding those who didn't adhere to the assigned intervention as defined by section 3.2.

4 SCREENING, RECRUITMENT, DEMOGRAPHICS AND WITHDRAWAL

4.1 Eligibility Criteria

Centre eligibility:

- Adult diabetes centre currently delivering DAFNE
- At least three DAFNE educators trained in delivering the five-week model of DAFNE
- Delivery of sufficient DAFNE courses per year to recruit the trial sample.

Participant eligibility:
Inclusion and exclusion criteria for participants are outlined in Table 3.

Table 3: Patient eligibility criteria

Inclusion Criteria	Exclusion Criteria
Age ≥18 years	HbA1c >12%
Diagnosis of type 1 diabetes for ≥6 months or post-honeymoon	Current use of continuous subcutaneous insulin infusion (CSII) pump therapy
Prepared to undertake multiple daily injections (MDI) therapy and frequent self-monitoring of blood glucose	Serious diabetes-related complications (e.g. blindness, renal dialysis), or other serious co-morbidities (e.g. psychosis, diagnosed eating disorder)
Available to attend all sessions	Unable to hear/speak/understand/read/write in English

For peer review only - http://bmjopen.bmj.com/site/about/guidelines.xhtml

Investigator has confidence that the patient is capable of adhering to all the trial protocol requirements	Previous participation in standard DAFNE course less than 5 years before proposed trial enrolment date
	Unable to give informed consent.

4.2 CONSORT

Using guidelines from the CONSORT statement [8], the summaries outlined Table 4 will be calculated in order to construct a CONSORT flowchart. Data will be presented overall and by treatment arm to show if any differences are present due to the sites treatment allocation.

For peer review only

For peer review only - http://bmjopen.bmj.com/site/about/guidelines.xhtml

Table 4: CONSORT Summary

**Screening Data**	• Number of participants assessed for eligibility at screening<br>• Number ineligible including reasons<br>• Number eligible but declined to participate including reasons
**Recruitment Data**	• Number of participants consented and recruited<br>• Number and percentage of those who attended all five sessions within either DAFNE/DAFNE*plus* course<br>• Number and percentage of those who completed the primary outcome (HbA1c) at 6 and 12 months follow-up
**Lost to Follow Up/Withdrawal Data**	• Number and percentage of those consented who dropped out and withdrew before the course<br>• Number and percentage of those consented who dropped out and withdrew after completing the course but before the 6-month follow-up<br>• Number and percentage of those consented who dropped out and withdrew after the 6-month follow up but before the 12-month follow-up
**Analysis Population Data**	• Number of those included in primary ITT set at 6 and 12 months follow-up<br>• Number of those included in full ITT set at 6 and 12 months follow-up<br>• Number of those included in PP set at 12 months follow-up

4.3 Withdrawal of Participants

Details of potential reasons for withdrawal are found in the protocol and summaries of these reasons plus any additional reasons found within the trial will be presented overall and split by treatment arm. Withdrawal numbers will also be summarised dependent on if the participant has withdrawn from the intervention, but continue with follow-up, withdrawal from follow-up but allow data collected to date to be used, withdrawal for all data collected to date to be used, or lost to follow up.

4.4 Baseline Characteristics

Baseline characteristics will be summarised at both the centre and participant level to assess the balance between the two treatment arms.

At the centre level, the stratification variables used (number of patients, number of educators and the total number of previous DAFNE courses delivered) within the randomisation will be presented by treatment arm to evaluate the balance between centres.

At the participant level, the variables shown in Table 5 as captured at baseline will be presented overall and by treatment arm. Categorical variables will be presented using counts and percentages, continuous variables will be presented with means and standard deviations or median and inter-quartile ranges as appropriate. No statistical significance testing will be used to test baseline imbalances between the two groups but any noteworthy differences will be descriptively reported.

Table 5: Baseline variables

For peer review only - http://bmjopen.bmj.com/site/about/guidelines.xhtml

Demographics
Age (Years)
Gender
Ethnicity
Highest qualification
**Medical History**
Duration of diabetes (Years)
Previously attended a DAFNE course
Pregnancy since diabetes
Current pregnancy, if yes gestation (weeks)
Use of lipid lowering medication
Use of antiplatelet agent
Use of medication for depression
Smoking status, if yes no of cigarettes per day
Physical activity levels
Complications – conditions and events (list as applicable)
Quick acting insulin (average daily dose, number of injections per day and type)
Background insulin (average daily dose, number of injections per day and type)
Pre-mixed insulin (average daily dose, number of injections per day and type)
Use of ratios
Presence of Lipohypertrophy
Number of blood glucose test performed (last 2 weeks)
Use of CGM, method and length of use (for Libre only – how it is funded and how it's being used)
Use of apps
Severe hypoglycaemic episode in the last year
Number of hypoglycaemic episodes that were unable to be treated themselves
Number of hypoglycaemic episodes that required paramedic assistance
Number of hypoglycaemic episodes that required A&E attendance
Number of hypoglycaemic episodes that required hospital admission
Blood glucose of hypoglycaemia
Admissions due to DKA (ever and in last year)
**Labs and Vital Signs**
BMI (kg/m2)
Blood pressure (mmHg)
HbA1c (mmol/mol)
Creatinine (µmol/L)
Albumin-creatinine (mg/mmol)
Cholesterol (mmol/L)
Triglycerides (mmol/L)
High Density Lipoprotein (HDL) cholesterol (mmol/L)

For peer review only - http://bmjopen.bmj.com/site/about/guidelines.xhtml

If there are any issues with partial dates in the database, the following approaches will be used to deal with them and therefore still allow derived time variables such as duration of diabetes to be calculated with sufficient precision:

If only year is available ("YYYY"), replace with "01/07/YYYY" or
If only month and year are available ("MM/YYYY"), replace with "15/MM/YYYY".

5 OUTLINE OF STATISTICAL ANALYSIS

Continuous variables will be summarised and presented by treatment group and overall as follows:

a) Mean and standard deviation (SD) for normal distribution
b) Median, Inter-Quartile Range (IQR), minimum and maximum for asymmetrical distribution

Categorical variables will be summarised and presented by treatment groups as the number of observations and proportion in each category and overall.

5.1 Outcome Measures

5.1.1 Primary Outcome

The primary outcome is glycaemic control defined as HbA1c, the primary endpoint refers to this data at 12-months but this will also be collected at baseline and 6-months (secondary endpoint). HbA1c is collected in mmol/mol but will be presented as a percentage. In order to convert between the two, the following calculation will be used [30]:

$$\text{HbA1c (\%)} = \text{HbA1c (mmol/mol)}/10.929 + 2.15$$

5.1.2 Secondary Outcomes - Biomedical

As an extension to the primary outcome, a secondary binary outcome to represent successful glycaemic control [9] will be calculated using the HbA1c data at 6- and 12-months. This outcome identified whether a participant achieved either a:

1) HbA1c $\leq$7.5% (58 mmol/mol) or
2) Reduction in HbA1c of ≥0.5% (≥5.5 mmol/mol).

Patient's BMI will be calculated using height and weight data at baseline, 6- and 12-months. Note – height is only collected once at baseline and used throughout. Weight can be collected either in kilograms (kg) or stones (st) and pounds (lb) and if collected in stone a conversion to kg will be completed using the following formula:

$$\text{Weight (kg)} = \text{Weight (st)}/0.15747 + \text{Weight (lb)}/2.2046$$

Similarly, height can be collected in metres (m) or feet (ft) and inches (in) and the following formula will be used to convert all to m:

$$\text{Height (m)} = \text{Height (ft)}/3.2808 + \text{Height (in)}/0.0254$$

BMI will then be calculated using the following formula:

$$\text{BMI (kg/m}^2) = \text{Weight (kg)} / \text{Height (m)}^2$$

Episodes of severe hypoglycaemia (as defined by the American Diabetes Association [9]), and incidence of DKA will be collected at baseline, 6-months and 12-months. Both will collect the number since the last visit.

For peer review only - http://bmjopen.bmj.com/site/about/guidelines.xhtml

Two different measures of lipids will be presented, high- and low-density lipoprotein which will be measured in mmol/L.

5.1.3 Secondary Outcomes - Psychological

- Audit of Diabetes-Dependent Quality of Life-15 (ADDQoL-15)

ADDQoL-15 is a questionnaire to measure the impact of diabetes and its treatment on a participant's QoL [10], [11]. It contains two overview items and 15 diabetes-specific items that relate to different aspects of life. Each has two parts, an impact score and an importance score. Each impact score is scored from -3 (very much better) to 1 (worse) and each importance score is from 3 (very important) to 0 (not at all important). These are then multiplied together to get a weighted impact score for each domain with -9 representing the maximum negative impact and 3 being the maximum positive impact. The average weighted impact (AWI) is then calculated as a mean of the weighted impacts for each domain.

If either the impact score or the importance score is missing, then the domain score cannot be computed and will not be included within the AWI. In the first instance, an AWI will only be calculated is all domain scores are available.

- DAWN Impact of Diabetes Profile (DIDP)

The participant's diabetes-specific quality of life will be assessed using the DIDP [12]. This consists of seven-items which investigates the impact diabetes has on different aspects of the participant's life. Each item is scored from 1 (very positive impact) to 7 (very negative impact), the composite score is the mean of all available responses, it ranges from 1-7 with lower scores indicating a greater positive impact. The percentage score is the composite score divided by 7, again lower percentages indicate greater positive impact.

- The Problem Areas in Diabetes

The Problem Areas in Diabetes (PAID) [13], [14] is a self-reported questionnaire that describe negative emotions related to diabetes (e.g. fear, anger, frustration) to assess diabetes distress. The short version, which will be used in this trial, consists of 11 items. Each question has five possible answers with a value from 0 to 4, with 0 representing "no problem" and 4 "a serious problem". The overall score is the sum of all questions and ranges from 0 (best) to 44 (worst). A score of 18+ indicates severe diabetes distress.

- Diabetes-specific Positive Well-being

Diabetes-Specific Positive Well-being will be measured using the specific subscale of the Well Being Questionnaire (W-BQ28) [15]. This questionnaire consist of four subscales each with four possible responses used to measure diabetes-specific well-being. Each item is scored from 0 (not at all) to 3 (all the time). The scores for this subscale are summed to get a total score ranging from 0-12 with higher scores representing more positive well-being.

- Fear of hypoglycaemia (HFS-II Short Form)

This 11-item questionnaire assesses the level of fear amongst people with diabetes [16] and contains five behavioural items and six worry items taken from the original full measure. Each item is scored

For peer review only - http://bmjopen.bmj.com/site/about/guidelines.xhtml

on a five-point Likert scale (0=never to 4=almost always) and a total score for the behaviour and worry subscales are calculated by summing the scores.
For missing values on the questionnaire, participant mean score will be imputed for missing values if at least nine of the questions have been completed.

- Health Status.

Health status will be measured using EQ-5D-5L [17] questionnaire which is a self-reported outcome measure which aims to assess the general health-related quality of life of the participant. It consists of five dimensions measure mobility, self-care, usual activity, pain/discomfort and anxiety/depression with each dimension having five possible responses. Participants also rate their overall health on the day of the interview on a 0–100 visual analogue scale with 0 = worst imaginable health state and 100 = best imaginable health state.

Scoring of EQ-5D-5L will be either scored using the relevant value set [18] or by mapping onto the EQ-5D-3L depending on the most up to date method at the time. The EQ-5D-5L health utility will not be calculated if any of the five dimensions are missing.

- Health and Self-Management in Diabetes

Health status will also be assessed using HASMID [19] assessment tool. It consists of ten items each with four possible responses. Responses are scored from zero to three with higher scores indicating little or no impact upon health related QoL. The overall questionnaire is a sum of all question scores and scored from 0 to 30 with higher scores indicating good health related QoL and a lower score indicating poor health-related quality of life. The scores are then used to calculate utility scores, the HASMiD health utility will not be calculated if any of the component ten questions are missing.

- Hypoglycaemia awareness

Awareness of hypoglycaemia will be measured using the Gold score questionnaire [20] which is a 1 item questionnaire consisting of a seven-point scale where one represent 'always aware of the onset of hypoglycaemia' and seven 'never aware of the onset of hypoglycaemia'.

5.1.4 Secondary Outcomes - Process Measures

- Diabetes Strengths and Resilience Questionnaire (DSRQ)

Adaptive behaviours and attitudes associated with overcoming challenges with diabetes management will be measured using the DSRQ [21]. The questionnaire consists of 12 items each scored from 1 (Never) to 5 (Always), the scores are summed to produce the total score which ranges from 12-60. Higher scores indicate perception of having greater T1D strengths.

- Self-Care Behaviours: Type 1 Diabetes (SCB-T1D)

Fifty items from the Self-Care Behaviours: Type 1 Diabetes (SCB-T1D) scale [22]will be used to assess the extent to which participants engaged with diabetes self-care behaviours. If an individual has completed over 50% of the items then the mean of the completed items will be used for the missing items. Otherwise the score will be coded as missing.

- System Usability Score (SUS)

For peer review only - http://bmjopen.bmj.com/site/about/guidelines.xhtml

The System Usability Score (SUS) [23] will be used to gather feedback on the DAFNEplus website at follow-up. As recommended by the scale authors the term 'system' will be replaced with 'Glucocollector' for the DAFNEplus group and 'bolus calculator' for the DAFNE (control) group. The SUS consists of 10 questions scored from 1 (strongly disagree) to 5 (strongly agree). Before calculating the total score the individual scores are transformed as follows:

- For each odd question (1,3,5,7 and 9) subtract 1
- For each even question (2,4,6,8 and 10) subtract from 5

The total score is the sum of these transformed values multiplied by 2.5, the score ranges from 1-100 with higher values indicating better usability.

- Diabetes Management Experience Questionnaire (DME-Q)

Satisfaction with diabetes treatment will be measured using the Diabetes Management Experience Questionnaire (DME-Q).

- Confidence in Diabetes Scale (CIDS)

The Confidence in Diabetes Scale (CIDS) [24] is a self-reported questionnaire with 20 items each scored from 1 (No, I am sure I cannot) to 5 (Yes, I am sure I can). The overall score is the sum of the items, minus the lowest possible score (20), divided by the score range (80) and multiplied by 100. This results in a 0-100 scale where higher scores indicate higher self-efficacy.

- Hypoglycaemia Confidence Scale (HCS)

The Hypoglycaemia Confidence Scale (HCS) [25] consists of 9 questions (8 items for participants without a partner) each rated from 1 (Not confident at all) to 4 (Very confident). The total score is calculated as the sum of the items divided by the number of items completed and ranges between 1-4 with higher scores indicating more confidence.

- Beliefs about Consequences

The Beliefs about Consequences questionnaire contains 6 items scored from 1 (not at all helpful) to 5 (extremely helpful), the items are summed to obtain the total score which ranges from 6-30.

- Self-Regulation Questionnaire (SRQ-T1D)

The SRQ-T1D questionnaire is an adaptation of the Self-Regulation Questionnaire [26], individual items are scored from 1 (strongly disagree) to 5 (strongly agree) and some items are reverse scaled (i.e. 1=5, 2=4, 3=3, 4=2, 5=1).

5.2 Analysis of Primary Outcome

The primary analysis will use the primary outcome of HbA1c at 12-months, using the primary ITT analysis population which is all consenting participants that have a baseline HbA1c > 7.5%. Descriptive statistics for baseline, 6- and 12-month HbA1c will be summarised and presented as mean, standard deviation (SD), median, min and max.

The treatment groups will be compared using a multiple linear regression model with coefficients estimated using GEE. The advantage of using GEE is that it is possible to calculate robust standard errors which are consistent even if the correlation structure is specified incorrectly [27], [28]. In this

For peer review only - http://bmjopen.bmj.com/site/about/guidelines.xhtml

For peer review only

model, an exchangeable correlation will be used to account for the clustering. In the event of any baseline differences in patient demographic characteristics, these covariates will be included in the model along with course. Adjustments for the stratification variables has not been included as they are at the centre level and therefore will be highly correlated with the course variable included in the model. The adjusted and unadjusted mean difference (MD) between DAFNE and DAFNE*plus* with associated 95% CI and p-value will be reported as well as the ICC from the model.

5.2.1 Model Checking

Given that correlation can lead to loss of information, ignoring the correlation structure can waste information and decrease standard errors when using an inappropriate analytical method. Model assumptions will be assessed graphically using the following methods:

- The linearity of the response variable will be assessed by a plot of the residuals against each explanatory variable in the model (curvilinear relationships). In cases of non-linearity, a transformation of the response variable could be performed, e.g log transformation or for particular fixed effect, a non-linear transformation of the particular fixed effect could be undertaken and included in the model,
- Constant variance will be assessed by plotting the residuals against the fitted values (errors have constant variance),
- Normality checks will be performed using a normal probability plot of the residuals (standardised) or histogram of the residuals,
- Partial residual plot will be used in identifying if quadratic or higher order terms are needed for any of the explanatory variables,
- Cook's distance can be used to indicate those observations that may be having an undue influence on the estimates. In cases of influential points, a sensitivity analyses with and without those points to assess the effects these points have on the regression coefficients will be undertaken.

5.3 Sensitivity Analyses of the Primary Outcome

Matching analyses will be undertaken on the primary outcome at 12-months using the full ITT and PP analysis sets as defined in table 4. These analyses will be completed using the primary analysis model with the only alternation the population set included within the analysis.

Additionally, a multi-level model will be completed using the primary ITT population on the primary outcome. This will contain the variables as mentioned in the original analysis but use course as a random effect to take into account the clustering in this model. These analyses will help to assess the robustness of the main trial result. The results in each case will be presented as adjusted MD of HbA1c at 12 months with associated 95% CI and p-value.

5.4 Subgroup Analyses

The following sub-group analysis will be completed on an ITT basis (Full ITT analysis population). The analysis will be the same model as the primary analysis with the addition of an interaction term between the treatment and subgroup to assess the stability of the result in different populations.

For peer review only - http://bmjopen.bmj.com/site/about/guidelines.xhtml

Treatment effect estimates with 95% confidence intervals will be calculated for each sub-group and a test for the overall interaction effect to reduce p-value use within each subgroup.

a) Previously attended a DAFNE course (Yes, No)
b) Use CGM or flash glucose monitoring between baseline and 12-month follow up (Yes, No)
c) Baseline HbA1c (≤7.5%, 7.5% < 8.5%, ≥8.5%)
d) Duration of diabetes (<15 years, ≥15 years)
e) Blood glucose level that symptoms of hypoglycaemia occur (do not feel symptoms, < 3mmol/l, ≥ 3mmol/l)
f) Self-reported use of the bolus advisor over the study duration (never or rarely, sometimes, often or always)
g) Age (≤34, 35-49, ≥50 years)
h) Sex (Male, Female)
i) BMI (Normal (<25), Overweight (25 < 30), Obese (≥30))
j) Socio-economic status (SES) as defined by the ONS Index of Multiple Deprivation (4 groups: above/below median in England, and above/below median in Scotland)
k) Total daily dose of insulin at baseline
l) Experience of lead course educator (Less experienced (6 courses or less within previous 3 years OR completed the DAFNE educator programme within previous year), More experienced (7+ courses within previous 3 years OR had continuous educator status for over 6 years))
m) Pregnant during the trial (Yes, No)
n) Type of basal insulin: (Human, Levemir, Lantus, Degludec, Toujeo)

5.5 Handling Missing Data

Missing observations can occur for numerous reasons (e.g. attrition) which can shrink the sample size, affects the precision of confidence intervals, reduce statistical power and biases parameter estimates [29]. Appropriately dealing with missing observations requires careful examination of data to identify the type and pattern of missingness.

In DAFNE*plus*, we anticipate that missing observations on the primary outcome (HbA1c) at 6 and 12months will occur amongst ITT participants. HbA1c results will be considered missing if the measure is outside +/- six weeks of the expected follow-up date. For the primary endpoint of HbA1c at 12 months, participant characteristics will be compared for those with and without the outcome. The aim of this is to explore any possible predictors of the missing outcome and evaluate the missing at random assumption. Multiple imputation strategies using a sequence of regression models [30] on the primary endpoint will be used where the missing values are filled ten times to generate ten complete data sets while utilising all variables that were included in the primary outcome analysis (section 5.4) as predictors. Any additional variables associated with the missing data will be included in the imputation model. This model will use a conservative approach by excluding treatment allocation.

If the data results in being missing not at random then a sensitivity analysis will be completed to assess the difference this makes on the results.

If weight is not recorded, other time points can be used as follows:

For peer review only - http://bmjopen.bmj.com/site/about/guidelines.xhtml

- Baseline weight missing - use 6-month weight,
- 6-month weight missing - if both baseline and 12-month weight are available, use the mean of these two. If only one of baseline or 12-month data are available, the recorded value will be used,
- All other circumstances - weight defined as missing.

5.6 Analysis of Secondary Outcomes - Biomedical

5.6.1 HbA1c

Additional secondary analyses on the primary outcome using the same model described in section 5.4 will be undertaken using HbA1c data collected at 6 months. This will be undertaken for all participants with HbA1C > 7.5% on an ITT basis. The adjusted and unadjusted mean difference (MD) between DAFNE and DAFNE*plus* with associated 95% CI and p-value will be reported as shown in table 7.11. Multiple imputations will be undertaken for missing HbA1c at 6 months using model described in 5.7.

The proportion of patients that have achieved improved glycaemic control will also be assessed. The patient is deemed to have improved glycaemic control if they achieved either: HbA1c $\leq$7.5% (58 mmol/mol) OR decrease in HbA1c ≥0.5% (≥5.5 mmol/mol). To test this, a GLM using a logit link function with treatment group, course and centre as random effects, and any baseline characteristics as used within the primary analysis model will be used (Logistic regression). Summary statistics for counts and percentages at 6 and 12 months follow up will be reported for DAFNE and DAFNE*plus* and overall. Treatment effect will be reported as both unadjusted or adjusted Odds Ratio (OR) with its associated 95% CI and p-value. This analysis will be undertaken using data collected at 6- and 12-month follow-up.

Additionally, it is important to assess the HbA1c trend over time.

5.6.2 Weight, BMI, Blood Pressure and Lipids

Summary statistics will be presented for each of these variables at baseline, and 12-months follow-up and all are treated as continuous variables. Summaries of weight and BMI at 6-months will be presented in the same way. Results will be presented by treatment group and overall and it will include the number, range and either the mean and SD or median and interquartile range depending on the distribution.

5.6.3 Severe Hypoglycaemia and DKA

The total number of episodes of hypoglycaemia will be treated as continuous and summary statistics of episodes since their last visit prior to the 12-month follow-up will be reported for DAFNE, DAFNE*plus* and overall.

To test for the difference between the two groups, the number of episodes of severe hypoglycaemia will be modelled using a negative binomial regression model with treatment group, baseline HbA1c and course (random effect). Treatment effect will be reported as Incidence Rate Ratios (IRR) with its associated 95% CI and p-value.

For peer review only - http://bmjopen.bmj.com/site/about/guidelines.xhtml

An additional analysis will be complete for the proportion of participants who experienced at least one episode of severe hypoglycaemia since their last visit prior to the 12 months follow-up. This will be modelled using a random effect logistic regression model. The model will include treatment group, baseline HbA1c and centre. Treatment effect will be reported and presented as Odds Ratio (OR) with its associated 95% CI and p-value.

5.6.4 Model Checking

Frequency graphs and the ratio of the variance to the mean will be used to assess the distribution of severe hypoglycaemia episodes. Failure to properly address existing over dispersion leads to serious underestimation of standard errors and misleading inference for the treatment effect. The Deviance and Likelihood Ratio Test (LRT) will be employed to assess goodness of fit of the Poisson linear regression model against two specific alternatives: a) a zero-inflated Poisson GLM (in the case of overdispersion due to excess zeros, or participants who experienced no episodes), and b) negative binomial regression for more general overdispersion. Further model diagnostics including measures of influence such as Cook's Distance will be undertaken for sensitivity analysis.

Unlike linear regression where graphical diagnostic displays can be very useful, for logistic regression models, the discreteness of binary data makes it difficult to interpret such displays. Three methods will be used for diagnostic checking of logistic regression models. Local mean deviance plots for detecting overall lack of fit, empirical probability plots to point out isolated departures from the fitted model and partial residual plots (smoothed) to identify specific causes of lack of fit.

5.7 Analysis of Secondary Outcomes - Psychological

All psychological outcomes will be analysed by the team at the University of Surrey, led by Debbie Cooke. The planned analysis will be defined by the team in a separate document and will be discussed with the statistical team to ensure consistency between methods. This will be signed off prior to data analysis.

5.8 Safety Outcomes

Adverse Events (AE) will be recorded throughout the trial and are defined as any unwanted medical occurrences which includes any episodes of diabetic ketoacidosis and any increase in frequency of severe hypoglycaemia. Serious Adverse Event (SAE) will also be recorded throughout the trial and are defined as AEs which result in hospitalisation or have a risk to life. A detailed description of AEs and SAEs can be found in the protocol.

Summary measures will be presented by treatment group as the number and percentage of participants reporting an AE/SAE as well as the total number of AE/SAEs reported and will be on an ITT basis. No formal statistical testing will be undertaken.

5.9 Statistical Software

This analysis will be carried out using any suitable packages such as R or STATA.

For peer review only - http://bmjopen.bmj.com/site/about/guidelines.xhtml

6 REFERENCES

6.1 Documents

DAFNE*plus* Protocol V2.0, 4th July 2018
Data Management Plan
ST001 The Statistical Analysis Plan
ST006 Undertaking a Statistical Analysis

6.2 Publications

[1] Diabetes UK, "Diabetes UK: Facts and Stats, October 2016," 2016. .

[2] The Diabetes Control and Complications Trial Research Group, "The effect of intensive treatment of diabetes on the development and progression of long-term complications in insulin-dependent diabetes mellitus," *N. Engl. J. Med.*, 1993.

[3] S. Heller, "Hypoglycaemia and diabetes," in *Textbook of Diabetes*, G. Pickup, J, WIlliams, Ed. Oxford: Blackwell Science, 2003, pp. 33.1-33.19.

[4] M. J. Campbell and S. J. Walters, *How to Design, Analyse and Report Cluster Randomised Trials in Medicine and Health Related Research*. Chichester, UK: John Wiley & Sons, Ltd, 2014.

[5] F. Li, Y. Lokhnygina, D. M. Murray, P. J. Heagerty, and E. R. DeLong, "An evaluation of constrained randomization for the design and analysis of group-randomized trials," *Stat. Methods Med. Res.*, vol. 35, no. 10, pp. 1565–1579, 2016.

[6] StataCorp, "Stata Statistical Software: Release 15," *2017*. 2017.

[7] ICH, "ICH Harmonised Tripartite Guideline. Statistical Principles for Clinical Trials. E9.," 1998. [Online]. Available: http://www.ich.org/fileadmin/Public_Web_Site/ICH_Products/Guidelines/Efficacy/E9/Step4/E9_Guideline.pdf.

[8] K. F. Schulz, D. G. Altman, D. Moher, and CONSORT Group, "CONSORT 2010 statement: updated guidelines for reporting parallel group randomised trials.," *BMJ*, vol. 340, no. mar23 1, p. c332, 2010.

[9] International Hypoglycaemia Study Group, "Glucose Concentrations of Less Than 3.0 mmol/L (54 mg/dL) Should Be Reported in Clinical Trials: A Joint Position Statement of the American Diabetes Association and the European Association for the Study of Diabetes: Table 1," *Diabetes Care*, vol. 40, no. 1, pp. 155–157, Jan. 2017.

[10] C. Bradley, C. Todd, T. Gorton, E. Symonds, A. Martin, and R. Plowright, "The development of an individualized questionnaire measure of perceived impact of diabetes on quality of life: the ADDQoL," *Qual. Life Res.*, vol. 8, no. 1/2, pp. 79–91, 1999.

[11] M. C. Rosal, B. Olendzki, G. W. Reed, O. Gumieniak, J. Scavron, and I. Ockene, "Diabetes self-management among low-income Spanish-speaking patients: A pilot study," *Ann. Behav. Med.*, vol. 29, no. 3, pp. 225–235, 2005.

[12] M. Peyrot, R. R. Rubin, T. Lauritzen, F. J. Snoek, D. R. Matthews, and S. E. Skovlund, "Psychosocial problems and barriers to improved diabetes management: results of the Cross-National Diabetes Attitudes, Wishes and Needs (DAWN) Study," *Diabet. Med.*, vol. 22, no. 10, pp. 1379–1385, Oct. 2005.

[13] W. H. Polonsky *et al.*, "Assessment of diabetes-related distress," *Diabetes Care*, 1995.

[14] D. A. W. and N. (DAWN), "Problem Area In Diabetes Questionnaire (PAID)," 1999. [Online]. Available: http://www.dawnstudy.com/content/dam/Dawnstudy/AFFILIATE/www-dawnstudy-com/Home/TOOLSANDRESOURCES/Documents/PAID_problem_areas_in_diabetes_questionnaire.pdf.

For peer review only - http://bmjopen.bmj.com/site/about/guidelines.xhtml

[15] C. Speight, J, Barendse, S, Bradley, "The W-BQ28: further development of the well-being Questionnaire to include diabetes-specific as well as generic subscales and new stress subscales," in *British Psychological Society, Division of Health Psychology*, 1999.

[16] J. Grabman *et al.*, "An empirically derived short form of the Hypoglycaemia Fear Survey II," *Diabet. Med.*, vol. 34, no. 4, pp. 500–504, Apr. 2017.

[17] M. Herdman *et al.*, "Development and preliminary testing of the new five-level version of EQ-5D (EQ-5D-5L)," *Qual. Life Res.*, vol. 20, no. 10, pp. 1727–1736, Dec. 2011.

[18] N. J. Devlin, K. K. Shah, Y. Feng, B. Mulhern, and B. van Hout, "Valuing health-related quality of life: An EQ-5D-5L value set for England," *Health Econ.*, Aug. 2017.

[19] J. Carlton *et al.*, "Developing a questionnaire to determine the impact of self-management in diabetes: giving people with diabetes a voice," *Health Qual. Life Outcomes*, vol. 15, no. 1, p. 146, Dec. 2017.

[20] A. E. Gold, K. M. MacLeod, and B. M. Frier, "Frequency of severe hypoglycemia in patients with type I diabetes with impaired awareness of hypoglycemia.," *Diabetes Care*, vol. 17, no. 7, pp. 697–703, Jul. 1994.

[21] M. E. Hilliard, E. Iturralde, J. Weissberg-Benchell, and K. K. Hood, "The Diabetes Strengths and Resilience Measure for Adolescents With Type 1 Diabetes (DSTAR-Teen): Validation of a New, Brief Self-Report Measure," *J. Pediatr. Psychol.*, vol. 42, no. 9, pp. 995–1005, Oct. 2017.

[22] C. D. Mansell P, Grant L, Bond R, Pouwer F, Page N, Davies A, Stenner K, Elliott J, Hopkins D, Jones A, Vallis M, Speight J, Lawton J, Clark M, Heller S, "Assessment of Self-Care Behaviours focused on Glycemic Control in Type 1 Diabetes: Development and Validation of the SCB-T1D Questionnaire." .

[23] J. Brooke, "SUS-A quick and dirty usability scale," *Usability Eval. Ind.*, vol. 189, no. 194, pp. 4–7, 1996.

[24] N. C. W. Van Der Ven *et al.*, "The confidence in diabetes self-care scale: Psychometric properties of a new measure of diabetes-specific self-efficacy in Dutch and U.S. patients with type 1 diabetes," *Diabetes Care*, vol. 26, no. 3, pp. 713–718, Mar. 2003.

[25] W. H. Polonsky, L. Fisher, D. Hessler, and S. V. Edelman, "Investigating Hypoglycemic Confidence in Type 1 and Type 2 Diabetes," *Diabetes Technol. Ther.*, vol. 19, no. 2, pp. 131–136, Feb. 2017.

[26] J. M. Brown, W. R. Miller, and L. A. Lawendowski, "The Self-Regulation Questionnaire.," in *Vandecreek, L. and Jackson, T.L., Eds., Innovations in Clinical Practice: A Sourcebook*, Vol. 17., Professional Resource Press/Professional Resource Exchange, Sarasota, FL, 1999, pp. 281–292.

[27] K.-Y. Liang and S. L. Zeger, "Longitudinal Data Analysis Using Generalized Linear Models," *Biometrika*, 1986.

[28] E. Demidenko and T. A. Stukel, "Influence analysis for linear mixed-effects models," *Stat. Med.*, 2005.

[29] P. D. Allison, "Missing Data," *Quant. Appl. Soc. Sci.*, 2001.

[30] T. E. Raghunathan, J. M. Lepkowski, J. Van Hoewyk, and P. Solenberger, "A multivariate technique for multiply imputing missing values using a sequence of regression models," *Surv. Methodol.*, 2001.

For peer review only - http://bmjopen.bmj.com/site/about/guidelines.xhtml

SPIRIT

STANDARD PROTOCOL ITEMS: RECOMMENDATIONS FOR INTERVENTIONAL TRIALS

SPIRIT 2013 checklist: Recommended items to address in a clinical trial protocol and related documents

Completed for DAFNE*plus* protocol submitted to BMJ Open

**Section/item**	**ItemNo**	**Description**	**Included? (page number)**
**Administrative information**			
Title	1	Descriptive title identifying the study design, population, interventions, and, if applicable, trial acronym	Yes<br>All elements included (p1)
Trial registration	2a	Trial identifier and registry name. If not yet registered, name of intended registry	Yes<br>Registered with ISRCTN42908016 (p3)
	2b	All items from the World Health Organization Trial Registration Data Set	Yes<br>See supplementary material 1
Protocol version	3	Date and version identifier	Yes (p2)
Funding	4	Sources and types of financial, material, and other support	Yes<br>Funded by the NIHR (p4)
Roles and responsibilities	5a	Names, affiliations, and roles of protocol contributors	Yes<br>Protocol contributors to be listed under Corporate 'DAFNEplus Group' Authorship are provided (p1)
	5b	Name and contact information for the trial sponsor	Yes<br>Sponsored by STH NHS FT (p4)
	5c	Role of study sponsor and funders, if any, in study design; collection, management, analysis, and interpretation of data; writing of the report; and the decision to submit the report for publication, including whether they will have ultimate authority over any of these activities	Yes (p4)

For peer review only

	5d	Composition, roles, and responsibilities of the coordinating centre, steering committee, endpoint adjudication committee, data management team, and other individuals or groups overseeing the trial, if applicable (see Item 21a for data monitoring committee)	Yes<br>Membership of trial co-ordinating centre, Trial Management Group and Trial Steering Committee listed (p5)
**Introduction**			
Background and rationale	6a	Description of research question and justification for undertaking the trial, including summary of relevant studies (published and unpublished) examining benefits and harms for each intervention	Yes<br>See 'Background and rationale' section (pp6-7)
	6b	Explanation for choice of comparators	Yes<br>See 'Standard DAFNE (control arm)' section, paragraph 3 (pp9-10)
Objectives	7	Specific objectives or hypotheses	Yes<br>See 'Aims and objectives' section (p7)
Trial design	8	Description of trial design including type of trial (eg, parallel group, crossover, factorial, single group), allocation ratio, and framework (eg, superiority, equivalence, noninferiority, exploratory)	Yes<br>See 'Trial design' section (p7-8)
**Methods: Participants, interventions, and outcomes**			
Study setting	9	Description of study settings (eg, community clinic, academic hospital) and list of countries where data will be collected. Reference to where list of study sites can be obtained	Yes<br>See 'Study setting' section (p8)
Eligibility criteria	10	Inclusion and exclusion criteria for participants. If applicable, eligibility criteria for study centres and individuals who will perform the interventions (eg, surgeons, psychotherapists)	Yes<br>See 'Eligibility criteria' section (p8)
Interventions	11a	Interventions for each group with sufficient detail to allow replication, including how and when they will be administered	Yes<br>See 'Interventions' section (pp9-11)

For peer review only

	11b	Criteria for discontinuing or modifying allocated interventions for a given trial participant (eg, drug dose change in response to harms, participant request, or improving/worsening disease)	Yes<br>See 'Criteria for withdrawal' section (p12)
	11c	Strategies to improve adherence to intervention protocols, and any procedures for monitoring adherence (eg, drug tablet return, laboratory tests)	Yes<br>Supervision of interventionists (p11) and fidelity assessment (pp14)
	11d	Relevant concomitant care and interventions that are permitted or prohibited during the trial	No<br>No restrictions on concomitant care other than use of CSII pump therapy (exclusion criteria 1)
Outcomes	12	Primary, secondary, and other outcomes, including the specific measurement variable (eg, systolic blood pressure), analysis metric (eg, change from baseline, final value, time to event), method of aggregation (eg, median, proportion), and time point for each outcome. Explanation of the clinical relevance of chosen efficacy and harm outcomes is strongly recommended	Yes<br>Outcomes listed in table 1 (pp16-17) and described in 'Outcomes' section (pp12-15)
Participant timeline	13	Time schedule of enrolment, interventions (including any run-ins and washouts), assessments, and visits for participants. A schematic diagram is highly recommended (see Figure)	Yes<br>Participant timeline shown in figure 1
Sample size	14	Estimated number of participants needed to achieve study objectives and how it was determined, including clinical and statistical assumptions supporting any sample size calculations	Yes<br>See 'sample size' section (p15)
Recruitment	15	Strategies for achieving adequate participant enrolment to reach target sample size	Yes<br>See 'recruitment' section (p9)
**Methods: Assignment of interventions (for controlled trials)**			
Allocation:			

For peer review only

Sequence generation	16a	Method of generating the allocation sequence (eg, computer-generated random numbers), and list of any factors for stratification. To reduce predictability of a random sequence, details of any planned restriction (eg, blocking) should be provided in a separate document that is unavailable to those who enrol participants or assign interventions	Yes<br>See 'random allocation' section (p12)
Allocation concealment mechanism	16b	Mechanism of implementing the allocation sequence (eg, central telephone; sequentially numbered, opaque, sealed envelopes), describing any steps to conceal the sequence until interventions are assigned	N/A – cluster RCT whereby trial centres randomised post ethical approval (p12)
Implementation	16c	Who will generate the allocation sequence, who will enrol participants, and who will assign participants to interventions	Yes<br>See 'random allocation' (p12)
Blinding (masking)	17a	Who will be blinded after assignment to interventions (eg, trial participants, care providers, outcome assessors, data analysts), and how	Yes<br>Blinding not possible during this trial – see 'blinding' section (p12)
	17b	If blinded, circumstances under which unblinding is permissible, and procedure for revealing a participant's allocated intervention during the trial	N/A
**Methods: Data collection, management, and analysis**			
Data collection methods	18a	Plans for assessment and collection of outcome, baseline, and other trial data, including any related processes to promote data quality (eg, duplicate measurements, training of assessors) and a description of study instruments (eg, questionnaires, laboratory tests) along with their reliability and validity, if known. Reference to where data collection forms can be found, if not in the protocol	Yes<br>Collection of outcome data described in 'Outcomes' section (pp12-15) and paragraph 1 of 'Data collection and management' (pp18-19)
	18b	Plans to promote participant retention and complete follow-up, including list of any outcome data to be collected for participants who discontinue or deviate from intervention protocols	Yes<br>See 'Data collection and management' section (pp18-19)

For peer review only

Data management	19	Plans for data entry, coding, security, and storage, including any related processes to promote data quality (eg, double data entry; range checks for data values). Reference to where details of data management procedures can be found, if not in the protocol	Yes<br>See 'Data collection and management' section (p18-19)
Statistical methods	20a	Statistical methods for analysing primary and secondary outcomes. Reference to where other details of the statistical analysis plan can be found, if not in the protocol	Yes<br>See 'Statistical analysis' section (p22) and SAP is provided in supplementary material
	20b	Methods for any additional analyses (eg, subgroup and adjusted analyses)	Yes<br>See 'Statistical analysis' section (p18) and SAP is provided in supplementary material
	20c	Definition of analysis population relating to protocol non-adherence (eg, as randomised analysis), and any statistical methods to handle missing data (eg, multiple imputation)	Yes<br>See 'Statistical analysis' section (p18) and SAP is provided in supplementary material
**Methods: Monitoring**			
Data monitoring	21a	Composition of data monitoring committee (DMC); summary of its role and reporting structure; statement of whether it is independent from the sponsor and competing interests; and reference to where further details about its charter can be found, if not in the protocol. Alternatively, an explanation of why a DMC is not needed	N/A<br>As described in 'Trial oversight committees', a DMEC has not been convened for this trial on the grounds that this is low risk. This has been approved by both Sponsor and TSC.
	21b	Description of any interim analyses and stopping guidelines, including who will have access to these interim results and make the final decision to terminate the trial	N/A
Harms	22	Plans for collecting, assessing, reporting, and managing solicited and spontaneously reported adverse events and other unintended effects of trial interventions or trial conduct	Yes<br>See 'Safety outcomes' (p15)

For peer review only

Auditing	23	Frequency and procedures for auditing trial conduct, if any, and whether the process will be independent from investigators and the sponsor	Yes<br>See final paragraph of 'Data collection and management' (p19) section for summary of plans for monitoring.
**Ethics and dissemination**			
Research ethics approval	24	Plans for seeking research ethics committee/institutional review board (REC/IRB) approval	Yes<br>See 'Research ethics approval section' (p19)
Protocol amendments	25	Plans for communicating important protocol modifications (eg, changes to eligibility criteria, outcomes, analyses) to relevant parties (eg, investigators, REC/IRBs, trial participants, trial registries, journals, regulators)	No<br>This has not been included due to word count restrictions.
Consent or assent	26a	Who will obtain informed consent or assent from potential trial participants or authorised surrogates, and how (see Item 32)	Yes<br>See paragraph 3 of 'Recruitment' section (p9)
	26b	Additional consent provisions for collection and use of participant data and biological specimens in ancillary studies, if applicable	N/A
Confidentiality	27	How personal information about potential and enrolled participants will be collected, shared, and maintained in order to protect confidentiality before, during, and after the trial	Yes<br>See 'Data collection and management' (pp18-19)
Declaration of interests	28	Financial and other competing interests for principal investigators for the overall trial and each study site	Yes<br>See declarations of interest submitted to journal
Access to data	29	Statement of who will have access to the final trial dataset, and disclosure of contractual agreements that limit such access for investigators	No but documented in Data Management Plan for trial
Ancillary and post-trial care	30	Provisions, if any, for ancillary and post-trial care, and for compensation to those who suffer harm from trial participation	No but provisions addressed in sponsor contracts
Dissemination policy	31a	Plans for investigators and sponsor to communicate trial results to participants, healthcare professionals, the	Yes

For peer review only

		public, and other relevant groups (eg, via publication, reporting in results databases, or other data sharing arrangements), including any publication restrictions	See 'Dissemination Policy' section (p19)
	31b	Authorship eligibility guidelines and any intended use of professional writers	No<br>This is not documented in protocol due to word count restrictions but addressed in dissemination strategy for trial. ICJME criteria used to assess authorship eligibility.
	31c	Plans, if any, for granting public access to the full protocol, participant-level dataset, and statistical code	No but documented in Data Management Plan for trial
**Appendices**			
Informed consent materials	32	Model consent form and other related documentation given to participants and authorised surrogates	No<br>Available if required for supplementary
Biological specimens	33	Plans for collection, laboratory evaluation, and storage of biological specimens for genetic or molecular analysis in the current trial and for future use in ancillary studies, if applicable	N/A

For peer review only
